# Supplementary material for: Organocatalytic asymmetric synthesis of Si-stereogenic silacycles
Source: Nat Commun. 2024 Jul 11;15:5846. doi: 10.1038/s41467-024-49988-2 (PMC11239892; doi:10.1038/s41467-024-49988-2)
Supplement: Supplementary file 1 — Supplementary Information [file 41467_2024_49988_MOESM1_ESM.pdf]

Supplementary Information

Organocatalytic asymmetric synthesis of *Si*-stereogenic silacycles

Jung Tae Han[1,2], Nobuya Tsuji[3], Hui Zhou[1], Markus Leutzsch[1] & Benjamin List[1,3]*

[1]Max-Planck-Institut für Kohlenforschung, Kaiser-Wilhelm-Platz 1, D-45470 Mülheim an der Ruhr, Germany

[2]Korea Institute of Science and Technology (KIST), Seoul 02792, Republic of Korea

[3]Institute for Chemical Reaction Design and Discovery (WPI-ICReDD), Hokkaido University, Sapporo 001-0021, Japan

*Email: list@kofo.mpg.de

Content

1. General considerations S2

2. Preparation and characterization of the silane starting materials S3

3. Substrate scope for the cyclization of bis(methallyl)silanes S7

4. Analytic data of products S7

5. Solvent effect S12

6. Control experiments S13

7. NMR experiments S15

8. ESI-MS experiments S17

9. Determination of the absolute configuration of silacycle **2a** S18

10. Limitations S38

11. DFT calculations S38

12. Copies of NMR spectra S42

13. Copies of HPLC and GC traces S76

14. References S95

1. General considerations

Chemicals

Unless otherwise noted, starting materials were obtained from Sigma-Aldrich, ABCR-GmbH, TCI, or Acros Co. Ltd. Moreover, commercially available reagents were used without additional purification. The bis(methallyl)silanes **1a**–**1q**[1,2] and chiral imidodiphosphorimidate acids (IDPis) **6a**–**4d**[3–5] were synthesized according to literature procedures.

Solvents

Solvents (tetrahydrofuran, toluene and benzene) were dried by distillation from an appropriate drying agent in the technical department of the Max-Planck-Institut für Kohlenforschung and received in Schlenk flasks under argon.

Inert Gas

Dry argon was purchased from Air Liquide with >99.5% purity.

Thin Layer Chromatography

Thin-layer chromatography (TLC) was performed using silica gel pre-coated plastic sheets (Polygram SIL G/UV_{254}, 0.2 mm, with fluorescent indicator; Macherey-Nagel) which was visualized with a UV lamp (254 nm) and/or basic $KMnO_4$.

Column Chromatography

Column chromatography (CC) was carried out using Merck silica gel (60 Å, 230−400 mesh, particle size 0.040−0.063 mm) using technical grade solvents. Elution was accelerated using compressed argon. All reported yields, unless otherwise noted, refer to spectroscopically and chromatographically pure compounds.

Nomenclature

Nomenclature follows the suggestions proposed by the computer program ChemBioDraw (12.0.3.1216) of CBD/cambridgesoft.

Nuclear Magnetic Resonance Spectroscopy

^{1}H, ^{13}C, ^{19}F, ^{31}P Nuclear magnetic resonance (NMR) spectra for compound characterization were recorded on Bruker AVIII-500 MHz, NMR spectrometer in a suitable deuterated solvent. The solvent employed and the respective measuring frequency are indicated for each experiment. Chemical shifts are reported with tetramethylsilane (TMS) serving as a universal reference of all nuclides. The resonance multiplicity is described as s (singlet), d (doublet), t (triplet), q (quadruplet), m (multiplet), and b (broad). All spectra were recorded at 298 K, processed with MestReNova 10.0.2 suits of program, and coupling constants are reported as observed. The residual deuterated solvent signal relative to tetramethylsilane was used as the internal reference in ^{1}H NMR spectra (e.g. $CDCl_3$ = 7.26 ppm, CD_2Cl_2 = 5.32 ppm). Signals are reported as follows: chemical shift δ in ppm (multiplicity, coupling constant *J* in Hz, number of protons). All X-nuclei spectra were acquired proton decoupled unless otherwise noted.

Mass Spectrometry

Electrospray ionization (ESI) mass spectrometry was conducted on a Bruker ESQ 3000 spectrometer. High resolution mass spectra were determined on a Bruker APEX III FTMS (7 T magnet). The ionization method and mode of detection employed is indicated for the

respective experiment and all masses are reported in atomic units per elementary charge (*m/z*) with an intensity normalized to the most intense peak.

Specific Rotations

Specific rotations ($[\alpha]_D^T$) were measured with a Rudolph RA Autopol IV Automatic Polarimeter at the indicated temperature with a sodium lamp (sodium D line, λ = 589 nm). Measurements were performed in an acid resistant 1 mL cell (50 mm length) with concentrations (g/(100 mL)) reported in the corresponding solvent.

High Performance Liquid Chromatography

High performance liquid chromatography (HPLC) was performed on a Shimadzu LC-20AD liquid chromatograph SIL-20AC auto sampler, CMB-20A using Daicel columns with a chiral stationary phase. All solvents used were HPLC-grade solvents purchased from Sigma-Aldrich. The column employed and the respective solvent mixture are indicated for each experiment.

Gas Chromatography

Gas chromatography (GC) analyses on a chiral stationary phase were performed on HP 6890 and 5890 series instruments (split-mode capillary injection system, flame ionization detector (FID), hydrogen carrier gas). The conditions employed are described in detail for the individual experiments.

Abbreviations

er = enantiomeric ratio, dr = diastereomeric ratio, TLC = thin layer chromatography, THF = tetrahydrofuran, Tf = SO_2CF_3, TBAB = tetrabutylammonium bromide, DMAP = 4-(dimethylamino)pyridine, MTPA = α-methoxy-α-trifluoromethylphenylacetic acid

2. Preparation and characterization of the silane starting materials

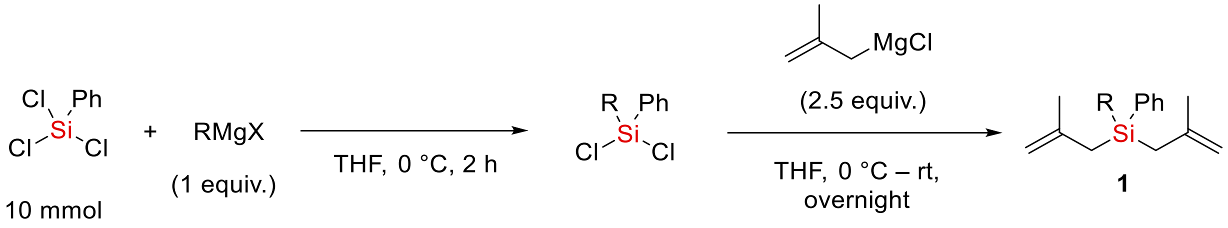

Method A: To a solution of phenyl trichlorosilane (10 mmol) in THF (20 mL), Grignard reagent (1 equiv.) was added at 0 °C under argon atmosphere. After the resulting mixture was stirred at 0 °C for 2 h, a solution of methallylmagnesium chloride (2.5 equiv.) was added at 0 °C. The mixture was then stirred at room temperature for overnight. After complete conversion indicated by TLC, the reaction was quenched with sat. NH_4Cl and extracted with pentanes. The combined organic phase was dried over Na_2SO_4, filtrated and dried under reduced pressure. Purification of the mixture by silica gel chromatography with hexanes afforded the desired silane **1**.

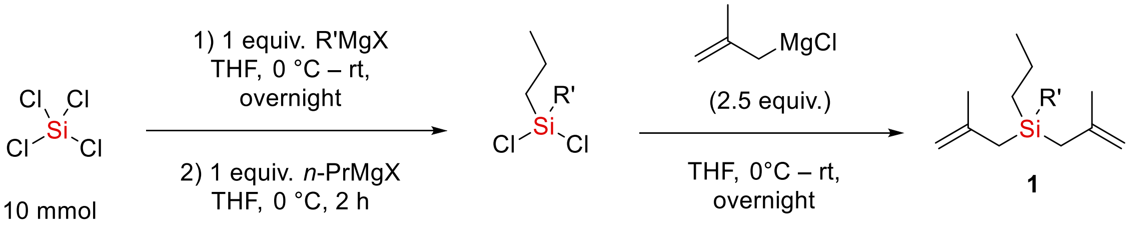

Method B: To a solution of silicon tetrachloride (10 mmol) in THF (20 mL), Grignard reagent (1 equiv.) was added at 0 °C under argon atmosphere and the resulting mixture was stirred at room temperature for overnight. Then, *n*-Propyl magnesium chloride (1 equiv.) was added at 0 °C. After the mixture was stirred at 0 °C for 2 h, a solution of methallylmagnesium chloride (2.5 equiv.) was added at 0 °C. The mixture was stirred at room temperature for overnight. After complete conversion indicated by TLC, the reaction was quenched with sat. NH_4Cl and extracted with pentanes. The combined organic phase was dried over Na_2SO_4, filtrated and dried under reduced pressure. Purification of the mixture by silica gel chromatography with hexanes afforded the desired silane **1**.

bis(2-methylallyl)(phenyl)(propyl)silane (1a)

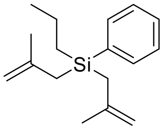

^{1}H NMR (501 MHz, CD_2Cl_2) δ 7.57–7.48 (m, 2H), 7.41–7.26 (m, 3H), 4.58 (dt, *J* = 2.8, 1.4 Hz, 2H), 4.50 (dd, *J* = 2.4, 1.2 Hz, 2H), 1.85 (s, 4H), 1.55 (s, 6H), 1.45–1.37 (m, 2H), 0.98 (t, *J* = 7.2 Hz, 3H), 0.93–0.88 (m, 2H).

^{13}C NMR (126 MHz, CD_2Cl_2) δ 143.5, 137.1, 134.5, 129.2, 127.8, 109.5, 25.5, 24.5, 18.6, 17.5, 14.8.

EI-HRMS (*m/z*): calculated for $C_{17}H_{26}Si_1$ [$M^{+\bullet}$]: 258.1798, found: 258.1798.

ethylbis(2-methylallyl)(phenyl)silane (1b)

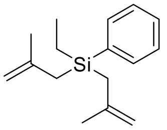

^{1}H NMR (501 MHz, CD_2Cl_2) δ 7.58–7.48 (m, 2H), 7.39–7.28 (m, 3H), 4.58 (dq, *J* = 2.9, 1.4 Hz, 2H), 4.50 (dq, *J* = 2.1, 1.0 Hz, 2H), 1.86 (s, 4H), 1.56 (s, 6H), 1.06–0.98 (m, 3H), 0.95–0.87 (m, 2H).

^{13}C NMR (126 MHz, CD_2Cl_2) δ 143.5, 136.9, 134.6, 129.3, 127.8, 109.5, 25.4, 24.1, 7.4, 3.8.

EI-HRMS (*m/z*): calculated for $C_{16}H_{24}Si_1$ [$M^{+\bullet}$]: 244.1639, found: 244.1642.

butylbis(2-methylallyl)(phenyl)silane (1c)

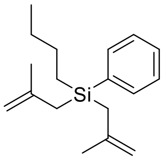

^{1}H NMR (501 MHz, CD_2Cl_2) δ 7.55–7.48 (m, 2H), 7.38–7.30 (m, 3H), 4.58 (dd, *J* = 2.5, 1.4 Hz, 2H), 4.52–4.47 (m, 2H), 1.85 (s, 4H), 1.55 (s, 6H), 1.38–1.32 (m, 4H), 0.93–0.86 (m, 5H).

^{13}C NMR (126 MHz, CD_2Cl_2) δ 143.5, 137.2 134.6 129.2, 127.8, 109.5, 27.1, 26.0, 25.5, 24.4, 13.8, 11.8.

EI-HRMS (*m/z*): calculated for $C_{18}H_{28}Si_1$ [$M^{+\bullet}$]: 272.1955, found: 272.1955.

bis(2-methylallyl)(pentyl)(phenyl)silane (1d)

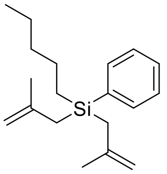

^{1}H NMR (501 MHz, CD_2Cl_2) δ 7.56–7.47 (m, 2H), 7.40–7.29 (m, 3H), 4.58 (dq, *J* = 2.9, 1.5 Hz, 2H), 4.49 (dd, *J* = 2.6, 1.3 Hz, 2H), 1.85 (s, 4H), 1.55 (s, 6H), 1.40–1.27 (m, 6H), 0.91–0.84 (m, 5H).

^{13}C NMR (126 MHz, CD_2Cl_2) δ 143.5, 137.2, 134.6, 129.2, 127.8, 109.5, 36.3, 25.5, 24.5, 23.4, 22.5, 14.2, 12.0.

EI-HRMS (*m/z*): calculated for $C_{19}H_{30}Si_1$ [$M^{+\bullet}$]: 286.2110, found: 286.2111.

hexylbis(2-methylallyl)(phenyl)silane (1e)

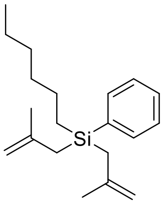

^{1}H NMR (501 MHz, CD_2Cl_2) δ 7.55–7.50 (m, 2H), 7.39–7.30 (m, 3H), 4.58 (dq, *J* = 3.0, 1.5 Hz, 2H), 4.49 (dd, *J* = 2.3, 1.2 Hz, 2H), 1.85 (s, 4H), 1.55 (s, 6H), 1.39–1.24 (m, 8H), 0.92–0.84 (m, 5H).

^{13}C NMR (126 MHz, CD_2Cl_2) δ 143.5, 137.2, 134.5, 129.2, 127.8, 109.5, 33.8, 31.7, 25.5, 24.5, 23.7, 22.9, 14.3, 12.1.

EI-HRMS (*m/z*): calculated for $C_{20}H_{32}Si_1$ [$M^{+\bullet}$]: 300.2266, found: 300.2268.

isopentylbis(2-methylallyl)(phenyl)silane (1f)

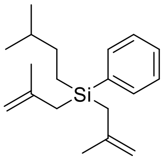

^{1}H NMR (501 MHz, CD_2Cl_2) δ 7.55–7.50 (m, 2H), 7.37–7.29 (m, 3H), 4.57 (dq, *J* = 2.6, 1.5 Hz, 2H), 4.49 (dd, *J* = 2.4, 1.2 Hz, 2H), 1.84 (s, 4H), 1.55 (s, 6H), 1.47 (dq, *J* = 13.2, 6.6 Hz, 1H), 1.29–1.23 (m, 2H), 0.92–0.86 (m, 8H).

^{13}C NMR (126 MHz, CD_2Cl_2) δ 143.5, 137.1, 134.6, 129.2, 127.8, 109.5, 32.6, 31.5, 25.5, 24.4, 22.1, 9.4.

EI-HRMS (*m/z*): calculated for $C_{19}H_{30}Si_1$ [$M^{+\bullet}$]: 286.2111, found: 286.2111.

isobutylbis(2-methylallyl)(phenyl)silane (1g)

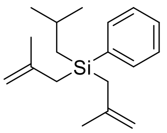

^{1}H NMR (501 MHz, CD_2Cl_2) δ 7.61–7.53 (m, 2H), 7.41–7.28 (m, 3H), 4.68–4.59 (m, 2H), 4.54 (d, *J* = 2.1 Hz, 2H), 1.93–1.83 (m, 5H), 1.59 (s, 6H), 0.94 (d, *J* = 6.9 Hz, 2H), 0.91 (d, *J* = 6.6 Hz, 6H).

^{13}C NMR (126 MHz, CD_2Cl_2) δ 143.6, 137.9, 134.8, 129.3, 127.9, 110.1, 26.7, 25.8, 25.2, 25.0, 23.1.

CI-HRMS (*m/z*): calculated for $C_{18}H_{29}Si_1$ ($[M+H]^+$): 273.2030, found: 273.2033.

bis(2-methylallyl)(naphthalen-2-yl)(propyl)silane (1h)

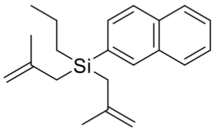

^{1}H NMR (501 MHz, CD_2Cl_2) δ 8.03 (s, 1H), 7.87–7.79 (m, 3H), 7.62 (dd, *J* = 8.2, 1.2 Hz, 1H), 7.52–7.45 (m, 2H), 4.59 (dq, *J* = 2.8, 1.4 Hz, 2H), 4.53 (dd, *J* = 2.4, 1.2 Hz, 2H), 1.94 (s, 4H), 1.56 (s, 6H), 1.50–1.42 (m, 2H), 1.05–0.98 (m, 5H).

^{13}C NMR (126 MHz, CD_2Cl_2) δ 143.5, 135.2, 134.9, 133.9, 133.0, 130.9, 128.3, 127.9, 126.8, 126.7, 126.1, 109.6, 25.5, 24.5, 18.7, 17.5, 14.8.

EI-HRMS (*m/z*): calculated for $C_{21}H_{28}Si_1$ [$M^{+\bullet}$]: 308.1955, found: 308.1955.

bis(2-methylallyl)(propyl)(*o*-tolyl)silane (1i)

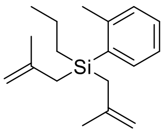

^{1}H NMR (501 MHz, CD_2Cl_2) δ 7.43 (dd, *J* = 7.4, 1.5 Hz, 1H), 7.25 (td, *J* = 7.5, 1.5 Hz, 1H), 7.18–7.10 (m, 2H), 4.58 (dq, *J* = 2.9, 1.5 Hz, 2H), 4.52 (dt, *J* = 2.3, 1.1 Hz, 2H), 2.49 (s, 3H), 1.93 (s, 4H), 1.58 (s, 6H), 1.46–1.39 (m, 2H), 1.01–0.95 (m, 5H).

^{13}C NMR (126 MHz, CD_2Cl_2) δ 144.2, 143.8, 135.5, 135.4, 130.1, 129.5, 125.0, 109.6, 25.2, 24.7, 23.4, 18.6, 17.6, 15.5.

EI-HRMS (*m/z*): calculated for $C_{18}H_{28}Si_1$ [$M^{+\bullet}$]: 272.1953, found: 272.1955.

bis(2-methylallyl)(propyl)(*m*-tolyl)silane (1j)

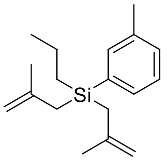

^{1}H NMR (501 MHz, CD_2Cl_2) 7.36–7.29 (m, 2H), 7.22 (t, *J* = 7.4 Hz, 1H), 7.17 (d, *J* = 7.6 Hz, 1H), 4.58 (dt, *J* = 2.6, 1.5 Hz, 2H), 4.50 (d, *J* = 2.5 Hz, 2H), 2.33 (s, 3H), 1.84 (s, 4H), 1.56 (s, 6H), 1.43–1.37 (m, 2H), 0.98 (t, *J* = 7.3 Hz, 3H), 0.91–0.87 (m, 2H).

^{13}C NMR (126 MHz, CD_2Cl_2) δ 143.6, 137.2, 136.9, 135.1, 131.6, 130.0, 127.7, 109.4, 25.5, 24.5, 21.6, 18.7, 17.5, 14.8.

EI-HRMS (*m/z*): calculated for $C_{18}H_{28}Si_1$ [$M^{+\bullet}$]: 272.1954, found: 272.1955.

bis(2-methylallyl)(propyl)(*p*-tolyl)silane (1k)

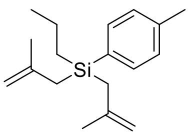

^{1}H NMR (501 MHz, CD_2Cl_2) δ 7.46–7.37 (m, 2H), 7.16 (d, *J* = 7.5 Hz, 2H), 4.57 (dq, *J* = 2.6, 1.3 Hz, 2H), 4.48 (dt, *J* = 2.1, 1.0 Hz, 2H), 2.32 (s, 3H), 1.82 (s, 4H), 1.55 (s, 6H), 1.43–1.35 (m, 2H), 0.97 (t, *J* = 7.2 Hz, 3H), 0.89–0.85 (m, 2H).

^{13}C NMR (126 MHz, CD_2Cl_2) δ 143.7, 139.2, 134.5, 133.3, 128.6, 109.4, 25.5, 24.6, 21.5, 18.7, 17.5, 14.9.

EI-HRMS (*m/z*): calculated for $C_{18}H_{28}Si_1$ [$M^{+\bullet}$]: 272.1954, found: 272.1955.

(4-methoxyphenyl)bis(2-methylallyl)(propyl)silane (1l)

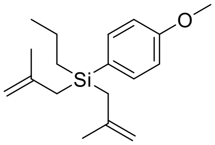

^{1}H NMR (501 MHz, CD_2Cl_2) δ 7.46–7.42 (m, 2H), 6.90–6.86 (m, 2H), 4.57 (dq, *J* = 2.9, 1.5 Hz, 2H), 4.49 (dd, *J* = 2.6, 1.1 Hz, 2H), 3.78 (s, 3H), 1.82 (s, 4H), 1.55 (s, 6H), 1.43–1.37 (m, 2H), 0.97 (t, *J* = 7.2 Hz, 3H), 0.90–0.86 (m, 2H).

^{13}C NMR (126 MHz, CD_2Cl_2) δ 160.6, 143.7, 136.0, 127.6, 113.5, 109.4, 55.2, 25.5, 24.7, 18.7, 17.5, 15.0.

EI-HRMS (*m/z*): calculated for $C_{18}H_{28}O_1Si_1$ [$M^{+\bullet}$]: 288.1902, found: 288.1904.

(4-fluorophenyl)bis(2-methylallyl)(propyl)silane (1m)

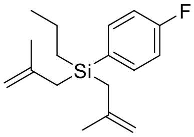

^{1}H NMR (501 MHz, CD_2Cl_2) δ 7.59–7.43 (m, 2H), 7.12–6.98 (m, 2H), 4.58 (dt, *J* = 2.8, 1.4 Hz, 2H), 4.49 (dq, *J* = 2.0, 0.9 Hz, 2H), 1.83 (s, 4H), 1.55 (s, 6H), 1.44–1.36 (m, 2H), 0.98 (t, *J* = 7.2 Hz, 3H), 0.92–0.87 (m, 2H).

^{13}C NMR (126 MHz, CD_2Cl_2) δ 163.9 (d, *J* = 247.0 Hz), 143.0, 136.5 (d, *J* = 7.6 Hz), 132.7 (d, *J* = 3.8 Hz), 114.9 (d, *J* = 20.2 Hz), 109.7, 25.4, 24.5, 18.6, 17.4, 14.9.

^{19}F NMR (471 MHz, CD_2Cl_2) δ -112.7.

EI-HRMS (*m/z*): calculated for $C_{17}H_{25}Si_1F_1$ [$M^{+\bullet}$]: 276.1700, found: 276.1704.

allylbis(2-methylallyl)(phenyl)silane (1n)

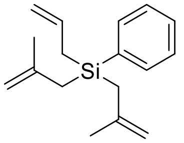

^{1}H NMR (501 MHz, CD_2Cl_2) δ 7.64–7.51 (m, 2H), 7.43–7.29 (m, 3H), 5.94–5.79 (m, 1H), 5.01–4.85 (m, 2H), 4.63 (dp, *J* = 2.6, 1.3 Hz, 2H), 4.56 (dt, *J* = 2.2, 1.0 Hz, 2H), 1.96 (dq, *J* = 7.9, 1.1 Hz, 2H), 1.90 (s, 4H), 1.59 (s, 6H).

^{13}C NMR (126 MHz, CD_2Cl_2) δ 143.3, 136.6, 134.8, 134.6, 129.6, 128.0, 114.7, 110.2, 25.7, 24.3, 20.4.

CI-HRMS (*m/z*): calculated for $C_{17}H_{25}Si_1$ ($[M+H]^+$): 257.1717, found: 257.1720.

tris(2-methylallyl)(phenyl)silane (1o)

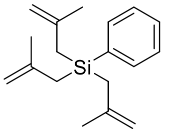

^{1}H NMR (501 MHz, CD_2Cl_2) δ 7.62–7.54 (m, 2H), 7.40–7.29 (m, 3H), 4.64 (dd, *J* = 2.6, 1.4 Hz, 3H), 4.56 (dt, *J* = 2.3, 1.1 Hz, 3H), 1.92 (s, 6H), 1.59 (s, 9H).

^{13}C NMR (126 MHz, CD_2Cl_2) δ 143.2, 136.8, 134.7, 129.4, 127.8, 110.4, 25.7, 24.1.

CI-HRMS (*m/z*): calculated for $C_{18}H_{27}Si_1$ ($[M+H]^+$): 271.1875, found: 271.1877.

cyclohexylbis(2-methylallyl)(propyl)silane (1p)

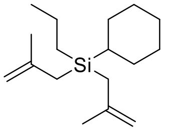

^{1}H NMR (501 MHz, CD_2Cl_2) δ 4.60 (dq, *J* = 2.8, 1.5 Hz, 2H), 4.54 (td, *J* = 2.0, 0.9 Hz, 2H), 1.76–1.69 (m, 11H), 1.63–1.57 (m, 4H), 1.43–1.35 (m, 2H), 1.25 – 1.17 (m, 5H), 0.95 (t, *J* = 7.2 Hz, 3H), 0.83–0.75 (m, 1H), 0.64–0.59 (m, 2H).

^{13}C NMR (126 MHz, CD_2Cl_2) δ 144.4, 109.2, 28.7, 28.0, 27.5, 25.7, 25.1, 23.1, 18.9, 17.8, 15.0.

ESI-HRMS (*m/z*): calculated for $C_{17}H_{32}Si_1Ag_1$ ($[M+Ag]^+$): 371.1320, found: 371.1319.

3. Substrate scope for the cyclization of bis(methallyl)silanes

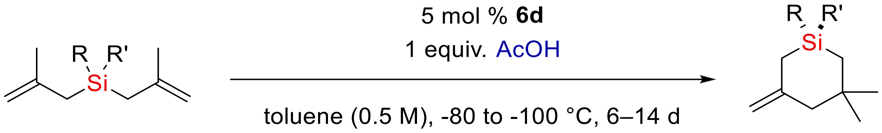

1 **2**

General procedure for the cyclization of bis(methallyl)silanes

Unless otherwise noted, a GC vial was charged with catalyst **6d** (5 mol %), toluene and acetic acid (1 equiv.), and the resulting mixture was cooled to -100 °C in a cryostat. After 10 min, bis(methallyl)silane **1** (0.1 or 0.2 mmol) was slowly added and the GC vial was stored for the given reaction time at the same temperature. After complete conversion indicated by TLC, the reaction was quenched with trimethylamine. The solvent was removed *in vacuo* and the mixture was purified by column chromatography on silica gel to afford the desired silacycle **2**.

4. Analytic data of products

(*S*)-3,3-dimethyl-5-methylene-1-phenyl-1-propylsilinane (2a)

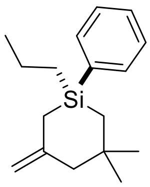

The reaction was conducted on a 0.2 mmol scale. The titled product was purified by column chromatography with hexanes as eluent to afford **2a** as a colorless oil (33.6 mg, 65% yield)

^{1}H NMR (501 MHz, CD_2Cl_2) δ 7.56–7.45 (m, 2H), 7.40–7.24 (m, 3H), 4.75 (dt, *J* = 2.6, 1.3 Hz, 1H), 4.56 (d, *J* = 2.7 Hz, 1H), 2.02–1.95 (m, 3H), 1.78 (d, *J* = 13.4 Hz, 1H), 1.35–1.26 (m, 2H), 1.03 (s, 3H), 0.97–0.85 (m, 5H), 0.82 (s, 3H), 0.77 (t, *J* = 8.3 Hz, 2H).

^{13}C NMR (126 MHz, CD_2Cl_2) δ 146.5, 138.6, 134.2, 129.2, 128.1, 109.8, 52.3, 34.6, 32.9, 32.4, 25.5, 23.7, 18.4, 17.8, 17.7.

CI-HRMS (*m/z*): calculated for $C_{17}H_{27}Si_1$ ($[M+H]^+$): 259.1875, found: 259.1877.

HPLC (IG-3R, Acetonitrile:Water = 70:30, 1.0 mL/min, 298 K, 220 nm): t_{R1} = 6.2 min, t_{R2} = 7.4 min, er = 95:5.

$[\alpha]_D^{25}$ = –19.9 (*c* 0.92, $CHCl_3$).

(*S*)-1-ethyl-3,3-dimethyl-5-methylene-1-phenylsilinane (2b)

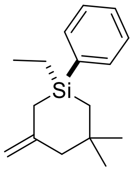

The reaction was conducted on a 0.2 mmol scale. The titled product was purified by column chromatography with hexanes as eluent to afford **2b** as a colorless oil (29.3 mg, 60% yield)

^{1}H NMR (501 MHz, CD_2Cl_2) δ δ 7.55–7.49 (m, 2H), 7.36–7.29 (m, 3H), 4.74 (dt, *J* = 2.7, 1.3 Hz, 1H), 4.55 (d, *J* = 2.7 Hz, 1H), 2.02–1.93 (m, 3H), 1.78 (d, *J* = 12.9 Hz, 1H), 1.02 (s, 3H), 0.94–0.85 (m, 5H), 0.83 (s, 3H), 0.78–0.72 (m, 2H).
^{13}C NMR (126 MHz, CD_2Cl_2) δ 146.5, 138.3, 134.2, 129.2, 128.1, 109.8, 52.3, 34.5, 32.7, 32.5, 25.0, 23.1, 7.5, 6.8.

EI-HRMS (*m/z*): calculated for $C_{16}H_{24}Si_1$ $[M^{+\bullet}]$: 244.1639, found: 244.1642.

HPLC (IG-3R, Acetonitrile:Water = 70:30, 1.0 mL/min, 298 K, 220 nm): t_{R1} = 5.7 min, t_{R2} = 7.5 min, er = 95:5.

$[\alpha]_D^{25}$ = –10.2 (*c* 1.10, $CHCl_3$).

(*S*)-1-butyl-3,3-dimethyl-5-methylene-1-phenylsilinane (2c)

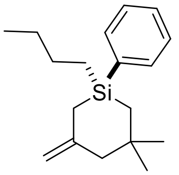

The reaction was conducted on a 0.2 mmol scale. The titled product was purified by column chromatography with hexanes as eluent to afford **2c** as a colorless oil (36.5 mg, 67% yield)

^{1}H NMR (501 MHz, CD_2Cl_2) δ 7.58–7.45 (m, 2H), 7.37–7.30 (m, 3H), 4.74 (dt, *J* = 2.7, 1.3 Hz, 1H), 4.55 (d, *J* = 2.7 Hz, 1H), 2.03–1.93 (m, 3H), 1.77 (d, *J* = 13.7 Hz, 1H), 1.32–1.21 (m, 4H), 1.02 (s, 3H), 0.96–0.81 (m, 8H), 0.80–0.74 (m, 2H).

^{13}C NMR (126 MHz, CD_2Cl_2) δ 146.5, 138.6, 134.2, 129.2, 128.0, 109.8, 52.3, 34.6, 32.8, 32.4, 26.9, 26.4, 25.5, 23.6, 14.9, 13.9.

EI-HRMS (*m/z*): calculated for $C_{18}H_{28}Si_1$ [$M^{+\bullet}$]: 272.1951, found: 272.1955.

HPLC (OJ-3R, Acetonitrile:Water = 55:45, 1.0 mL/min, 298 K, 220 nm): t_{R1} = 44.6 min, t_{R2} = 48.3 min, er = 95:5.

$[\alpha]_D^{25}$ = –12.3 (*c* 0.80, $CHCl_3$).

(*S*)-3,3-dimethyl-5-methylene-1-pentyl-1-phenylsilinane (2d)

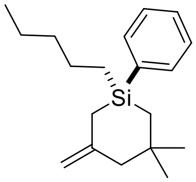

The reaction was conducted on a 0.2 mmol scale. The titled product was purified by column chromatography with hexanes as eluent to afford **2d** as a colorless oil (39.0 mg, 68% yield)

^{1}H NMR (501 MHz, CD_2Cl_2) δ 7.57–7.48 (m, 2H), 7.39–7.30 (m, 3H), 4.74 (dt, *J* = 2.7, 1.3 Hz, 1H), 4.55 (d, *J* = 2.7 Hz, 1H), 2.02–1.94 (m, 3H), 1.78 (d, *J* = 13.5 Hz, 1H), 1.30–1.22 (m, 6H), 1.02 (s, 3H), 0.96–0.81 (m, 8H), 0.79–0.73 (m, 2H).

^{13}C NMR (126 MHz, CD_2Cl_2) δ 146.5, 138.6, 134.2, 129.2, 128.1, 109.8, 52.3, 36.1, 34.6, 32.8, 32.5, 25.5, 23.8, 23.6, 22.6, 15.1, 14.1.

CI-HRMS (*m/z*): calculated for $C_{19}H_{31}Si_1$ ($[M+H]^+$): 287.2187, found: 287.2190.

HPLC (IG-3R, Acetonitrile:Water = 70:30, 1.0 mL/min, 298 K, 220 nm): t_{R1} = 8.1 min, t_{R2} = 8.7 min, er = 95:5.

$[\alpha]_D^{25}$ = –12.0 (*c* 1.02, $CHCl_3$).

(*S*)-1-hexyl-3,3-dimethyl-5-methylene-1-phenylsilinane (2e)

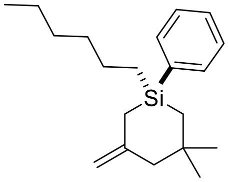

The reaction was conducted on a 0.2 mmol scale. The titled product was purified by column chromatography with hexanes as eluent to afford **2e** as a colorless oil (37.9 mg, 63% yield)

^{1}H NMR (501 MHz, CD_2Cl_2) δ 7.56–7.48 (m, 2H), 7.39–7.28 (m, 3H), 4.74 (dt, *J* = 2.7, 1.3 Hz, 1H), 4.55 (d, *J* = 2.7 Hz, 1H), 2.01–1.94 (m, 3H), 1.77 (d, *J* = 13.3 Hz, 1H), 1.30–1.18 (m, 8H), 1.02 (s, 3H), 0.95–0.81 (m, 8H), 0.79–0.72 (m, 2H).

^{13}C NMR (126 MHz, CD_2Cl_2) δ 146.5, 138.6, 134.2, 129.1, 128.0, 109.8, 52.3, 34.6, 33.6, 32.8, 32.5, 31.9, 25.5, 24.1, 23.6, 23.0, 15.1, 14.3.

EI-HRMS (*m/z*): calculated for $C_{20}H_{32}Si_1$ [$M^{+\bullet}$]: 300.2266, found: 300.2268.

HPLC (IG-3R, Acetonitrile:Water = 60:40, 1.0 mL/min, 298 K, 220 nm): t_{R1} = 26.0 min, t_{R2} = 27.2 min, er = 93:7.

$[\alpha]_D^{25}$ = –10.3 (*c* 1.01, $CHCl_3$).

(*S*)-1-isopentyl-3,3-dimethyl-5-methylene-1-phenylsilinane (2f)

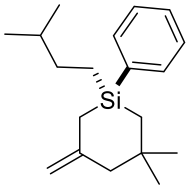

The reaction was conducted on a 0.1 mmol scale. The titled product was purified by column chromatography with hexanes as eluent to afford **2f** as a colorless oil (18.3 mg, 64% yield)

^{1}H NMR (501 MHz, CD_2Cl_2) δ 7.58–7.47 (m, 2H), 7.37–7.30 (m, 3H), 4.75 (dt, *J* = 2.6, 1.3 Hz, 1H), 4.56 (d, *J* = 2.7 Hz, 1H), 2.04–1.93 (m, 3H), 1.78 (d, *J* = 13.4 Hz, 1H), 1.49–1.34 (m, 1H), 1.21–1.08 (m, 2H), 1.03 (s, 3H), 0.95–0.82 (m, 11H), 0.79–0.72 (m, 2H).

^{13}C NMR (126 MHz, CD_2Cl_2) δ 146.5, 138.6, 134.2, 129.2, 128.1, 109.8, 52.3, 34.6, 33.1, 32.8, 32.5, 31.3, 25.4, 23.6, 22.3, 22.2, 12.5.

EI-HRMS (*m/z*): calculated for $C_{19}H_{30}Si_1$ [$M^{+\bullet}$]: 286.2109, found: 286.2111.

HPLC (IG-3R, Acetonitrile:Water = 60:40, 1.0 mL/min, 298 K, 220 nm): t_{R1} = 16.8 min, t_{R2} = 17.6 min, er = 92:8.

$[\alpha]_D^{25}$ = –10.3 (*c* 0.80, $CHCl_3$).

(*S*)-1-isobutyl-3,3-dimethyl-5-methylene-1-phenylsilinane (2g)

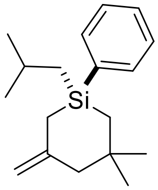

The reaction was conducted on a 0.1 mmol scale. The titled product was purified by column chromatography with hexanes as eluent to afford **2g** as a colorless oil (19.1 mg, 70% yield)

^{1}H NMR (501 MHz, CD_2Cl_2) δ 7.58–7.51 (m, 2H), 7.39–7.30 (m, 3H), 4.74 (dt, *J* = 2.7, 1.3 Hz, 1H), 4.54 (d, *J* = 2.7 Hz, 1H), 2.05–1.93 (m, 3H), 1.77 (d, *J* = 13.2 Hz, 1H), 1.63 (ddd, *J* = 12.8, 7.3, 6.5 Hz, 1H), 1.03 (s, 3H), 0.99 (d, *J* = 14.7 Hz, 1H), 0.87–0.74 (m, 12H).

^{13}C NMR (126 MHz, CD_2Cl_2) δ 146.5, 138.9, 134.3, 129.1, 128.0, 109.8, 52.3, 34.6, 33.5, 31.9, 26.6, 26.4, 26.2, 26.1, 25.2, 24.6.

CI-HRMS (*m/z*): calculated for $C_{18}H_{29}Si_1$ [$M^{+\bullet}$]: 273.2031, found: 273.2033.

HPLC (IG-3R, Acetonitrile:Water = 70:30, 1.0 mL/min, 298 K, 220 nm): t_{R1} = 6.3 min, t_{R2} = 6.9 min, er = 91:9.

$[\alpha]_D^{25}$ = –16.0 (*c* 0.73, $CHCl_3$).

(*S*)-3,3-dimethyl-5-methylene-1-(naphthalen-2-yl)-1-propylsilinane (2h)

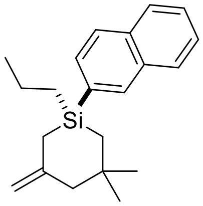

The reaction was conducted on a 0.1 mmol scale. The titled product was purified by column chromatography with hexanes as eluent to afford **2h** as a colorless oil (22.2 mg, 72% yield)

^{1}H NMR (501 MHz, CD_2Cl_2) 7.90–7.76 (m, 3H), 7.61 (dd, *J* = 8.1, 1.2 Hz, 1H), 7.49 (dt, *J* = 6.2, 3.4 Hz, 2H), 4.81 (dt, *J* = 2.6, 1.3 Hz, 1H), 4.58 (d, *J* = 2.7 Hz, 1H), 2.10 (d, *J* = 13.4 Hz, 1H), 2.05–1.98 (m, 2H), 1.85 (d, *J* = 13.3 Hz, 1H), 1.38–1.28 (m, 2H), 1.08–1.03 (m, 4H), 0.96–0.90 (m, 4H), 0.86–0.80 (m, 5H).

^{13}C NMR (126 MHz, CD_2Cl_2) δ 146.4, 136.2, 134.9, 134.0, 133.4, 130.6, 128.3, 128.0, 127.1, 126.7, 126.2, 109.9, 52.3, 34.6, 33.0, 32.3, 25.6, 23.7, 18.4, 17.9, 17.8.

CI-HRMS (*m/z*): calculated for $C_{21}H_{29}Si_1$ ($[M+H]^+$): 309.2031, found: 309.2033.

HPLC (OJ-3R, Acetonitrile:Water = 55:45, 1.0 mL/min, 298 K, 254 nm): t_{R1} = 76.0 min, t_{R2} = 81.3 min, er = 95:5.

$[\alpha]_D^{25}$ = –12.9 (*c* 0.81, $CHCl_3$).

(*S*)-3,3-dimethyl-5-methylene-1-propyl-1-(*o*-tolyl)silinane (2i)

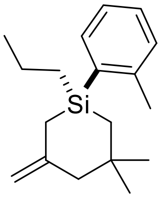

The reaction was conducted on a 0.1 mmol scale. The titled product was purified by column chromatography with hexanes as eluent to afford **2i** as a colorless oil (18.5 mg, 68% yield)

^{1}H NMR (501 MHz, CD_2Cl_2) δ 7.42 (dd, *J* = 7.7, 1.5 Hz, 1H), 7.23 (td, *J* = 7.4, 1.5 Hz, 1H), 7.12 (dt, *J* = 6.9, 3.3 Hz, 2H), 4.76 (dt, *J* = 2.7, 1.3 Hz, 1H), 4.57 (d, *J* = 2.7 Hz, 1H), 2.40 (s, 3H), 1.97 (dt, *J* = 25.3, 12.6 Hz, 3H), 1.87 (d, *J* = 13.2 Hz, 1H), 1.24–1.11 (m, 2H), 0.99 (s, 3H), 0.97 (s, 2H), 0.87–0.77 (m, 8H).

^{13}C NMR (126 MHz, CD_2Cl_2) δ 146.6, 143.7, 136.7, 135.0, 129.8, 129.3, 125.0, 109.6, 52.1, 34.5, 32.8, 31.9, 26.6, 24.6, 23.3, 18.3, 17.9, 17.5.

EI-HRMS (*m/z*): calculated for $C_{18}H_{28}Si_1$ [$M^{+\bullet}$]: 272.1953, found: 272.1955.

HPLC (OJ-3R, Acetonitrile:Water = 55:45, 1.0 mL/min, 298 K, 220 nm): t_{R1} = 36.6 min, t_{R2} = 39.2 min, er = 63:37.

$[\alpha]_D^{25}$ = –4.1 (*c* 0.29, $CHCl_3$).

(*S*)-3,3-dimethyl-5-methylene-1-propyl-1-(*m*-tolyl)silinane (2j)

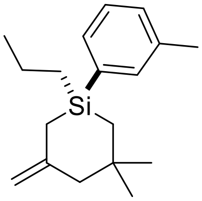

The reaction was conducted on a 0.1 mmol scale. The titled product was purified by column chromatography with hexanes as eluent to afford **2j** as a colorless oil (20.2 mg, 74% yield)

^{1}H NMR (501 MHz, CD_2Cl_2) δ 7.34–7.28 (m, 2H), 7.22 (t, *J* = 7.4 Hz, 1H), 7.19–7.10 (m, 1H), 4.74 (dt, *J* = 2.8, 1.3 Hz, 1H), 4.55 (d, *J* = 2.7 Hz, 1H), 2.34 (s, 3H), 2.03–1.91 (m, 3H), 1.77 (d, *J* = 13.5 Hz, 1H), 1.35–1.23 (m, 2H), 1.02 (s, 3H), 0.95–0.82 (m, 8H), 0.78–0.73 (m, 2H).

^{13}C NMR (126 MHz, CD_2Cl_2) δ 146.6, 138.4, 137.4, 134.8, 131.2, 129.9, 127.9, 109.7, 52.3, 34.6, 32.7, 32.6, 25.5, 23.7, 21.6, 18.4, 17.8, 17.8.

EI-HRMS (*m/z*): calculated for $C_{18}H_{28}Si_1$ [$M^{+\bullet}$]: 272.1953, found: 272.1955.

HPLC (IG-3R, Acetonitrile:Water = 70:30, 1.0 mL/min, 298 K, 220 nm): t_{R1} = 6.0 min, t_{R2} = 6.4 min, er = 96:4.

$[\alpha]_D^{25}$ = –12.5 (*c* 0.80, $CHCl_3$).

(*S*)-3,3-dimethyl-5-methylene-1-propyl-1-(*p*-tolyl)silinane (2k)

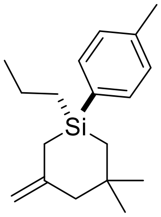

The reaction was conducted on a 0.1 mmol scale. The titled product was purified by column chromatography with hexanes as eluent to afford **2k** as a colorless oil (19.1 mg, 70% yield)

^{1}H NMR (501 MHz, CD_2Cl_2) δ 7.44–7.38 (m, 2H), 7.16 (d, *J* = 7.5 Hz, 2H), 4.72 (dt, *J* = 2.9, 1.4 Hz, 1H), 4.54 (d, *J* = 2.7 Hz, 1H), 2.33 (s, 3H), 2.01–1.93 (m, 3H), 1.75 (d, *J* = 13.3 Hz, 1H), 1.34–1.24 (m, 2H), 1.02 (s, 3H), 0.94–0.81 (m, 8H), 0.77–0.72 (m, 2H).

^{13}C NMR (126 MHz, CD_2Cl_2) δ 146.6, 139.1, 134.8, 134.2, 128.9, 109.6, 52.3, 34.6, 32.9, 32.4, 25.6, 23.8, 21.6, 18.4, 17.9, 17.8.

EI-HRMS (*m/z*): calculated for $C_{18}H_{28}Si_1$ [$M^{+\bullet}$]: 272.1953, found: 272.1955.

HPLC (IG-3R, Acetonitrile:Water = 70:30, 1.0 mL/min, 298 K, 220 nm): t_{R1} = 7.3 min, t_{R2} = 10.4 min, er = 95:5.

$[\alpha]_D^{25}$ = –9.1 (*c* 0.80, $CHCl_3$).

(*S*)-1-(4-methoxyphenyl)-3,3-dimethyl-5-methylene-1-propylsilinane (2l)

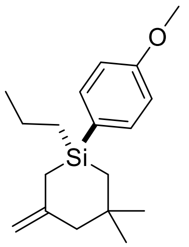

The reaction was conducted on a 0.1 mmol scale. The titled product was purified by column chromatography with hexanes as eluent to afford **2l** as a colorless oil (15.9 mg, 55% yield)

^{1}H NMR (501 MHz, CD_2Cl_2) δ 7.47–7.41 (m, 2H), 6.92–6.85 (m, 2H), 4.72 (dt, *J* = 2.7, 1.3 Hz, 1H), 4.54 (d, *J* = 2.7 Hz, 1H), 3.79 (s, 3H), 2.00–1.91 (m, 3H), 1.74 (d, *J* = 13.3 Hz, 1H), 1.33–1.25 (m, 2H), 1.02 (s, 3H), 0.92–0.88 (m, 4H), 0.85–0.81 (m, 4H), 0.76–0.72 (m, 2H).

^{13}C NMR (126 MHz, CD_2Cl_2) δ 160.8, 146.7, 135.6, 129.2, 113.8, 109.6, 55.3, 52.3, 34.6, 32.9, 32.3, 25.7, 23.9, 18.4, 18.0, 17.8.

EI-HRMS (*m/z*): calculated for $C_{18}H_{28}O_1Si_1$ [$M^{+\bullet}$]: 288.1902, found: 288.1904.

HPLC (IG-3R, Acetonitrile:Water = 60:40, 1.0 mL/min, 298 K, 220 nm): t_{R1} = 15.7 min, t_{R2} = 27.8 min, er = 91:9.

$[\alpha]_D^{25}$ = –5.2 (*c* 0.84, $CHCl_3$).

(*S*)-1-(4-fluorophenyl)-3,3-dimethyl-5-methylene-1-propylsilinane (2m)

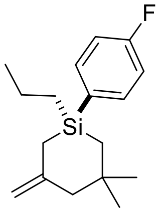

The reaction was conducted on a 0.1 mmol scale. The titled product was purified by column chromatography with hexanes as eluent to afford **2m** as a colorless oil (16.6 mg, 60% yield)

^{1}H NMR (501 MHz, CD_2Cl_2) δ 7.54–7.46 (m, 2H), 7.09–6.99 (m, 2H), 4.74 (dt, *J* = 2.6, 1.2 Hz, 1H), 4.55 (dt, *J* = 2.7, 0.8 Hz, 1H), 2.02–1.92 (m, 3H), 1.76 (d, *J* = 12.6 Hz, 1H), 1.31–1.25 (m, 2H), 1.02 (s, 3H), 0.95–0.86 (m, 5H), 0.79 (s, 3H), 0.77–0.72 (m, 2H).

^{13}C NMR (126 MHz, CD_2Cl_2) δ 164.0 (d, *J* = 247.2 Hz), 146.3, 136.2 (d, *J* = 7.2 Hz), 134.16 (d, *J* = 3.7 Hz), 115.1 (d, *J* = 19.6 Hz), 109.9, 52.2, 33.0, 32.1, 31.0, 25.6, 23.7, 18.3, 17.9, 17.7.

^{19}F NMR (471 MHz, CD_2Cl_2) δ -113.2

EI-HRMS (*m/z*): calculated for $C_{17}H_{25}F_1Si_1$ [$M^{+\bullet}$]: 276.1703, found: 276.1704.

HPLC (IG-3R, Acetonitrile:Water = 70:30, 1.0 mL/min, 298 K, 220 nm): t_{R1} = 6.2 min, t_{R2} = 7.2 min, er = 86.5:13.5.

$[\alpha]_D^{25}$ = –14.2 (*c* 0.30, $CHCl_3$).

(*S*)-1-allyl-3,3-dimethyl-5-methylene-1-phenylsilinane (2n)

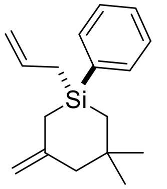

The reaction was conducted on a 0.1 mmol scale. The titled product was purified by column chromatography with hexanes as eluent to afford **2n** as a colorless oil (13.1 mg, 51% yield)

^{1}H NMR (501 MHz, CD_2Cl_2) δ 7.56–7.50 (m, 2H), 7.38–7.30 (m, 3H), 5.76–5.64 (m, 1H), 4.85–4.82 (m, 1H), 4.80 (dp, *J* = 3.6, 1.3 Hz, 1H), 4.77 (dt, *J* = 2.6, 1.3 Hz, 1H), 4.58 (d, *J* = 2.6 Hz, 1H), 2.04–1.95 (m, 3H), 1.84–1.75 (m, 3H), 1.05 (s, 3H), 0.96–0.88 (m, 2H), 0.82 (s, 3H).

^{13}C NMR (126 MHz, CD_2Cl_2) δ 146.0, 137.6, 134.6, 134.3, 129.4, 128.1, 113.9, 110.2, 52.2, 34.5, 33.0, 32.2, 24.9, 22.9, 22.9.

EI-HRMS (*m/z*): calculated for $C_{17}H_{24}Si_1$ [$M^{+\bullet}$]: 256.1640, found: 256.1642.

HPLC (IG-3R, Acetonitrile:Water = 60:40, 1.0 mL/min, 298 K, 220 nm): t_{R1} = 11.2 min, t_{R2} = 14.5 min, er = 96.5:3.5.

$[\alpha]_D^{25}$ = –21.5 (*c* 0.55, $CHCl_3$).

(*S*)-3,3-dimethyl-1-(2-methylallyl)-5-methylene-1-phenylsilinane (2o)

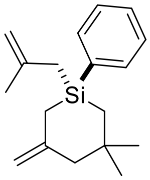

The reaction was conducted on a 0.2 mmol scale and pivalic acid (1 equiv.) was used as an additive. The titled product was purified by column chromatography with hexanes as eluent to afford **2o** as a colorless oil (28.1 mg, 52% yield)

^{1}H NMR (501 MHz, CD_2Cl_2) δ 7.57–7.48 (m, 2H), 7.38–7.30 (m, 3H), 4.76 (dt, J = 2.7, 1.3 Hz, 1H), 4.57–4.55 (m, 1H), 4.54 (dq, J = 2.8, 1.5 Hz, 1H), 4.40 (dq, J = 2.0, 0.9 Hz, 1H), 2.06 (d, J = 13.9 Hz, 1H), 2.01–1.93 (m, 2H), 1.80 (d, J = 13.4 Hz, 1H), 1.78–1.71 (m, 2H), 1.50 (s, 3H), 1.05–0.88 (m, 5H), 0.75 (s, 3H).

^{13}C NMR (126 MHz, CD_2Cl_2) δ 146.1, 143.2, 138.0, 134.3, 129.3, 128.0, 110.2, 109.3, 52.2, 34.6, 34.0, 31.4, 27.4, 25.6, 25.4, 23.3.

EI-HRMS (*m/z*): calculated for $C_{18}H_{26}Si_1$ [$M^{+\bullet}$]: 270.1798, found: 258.1798.

HPLC (IG-3R, Acetonitrile:Water = 70:30, 1.0 mL/min, 298 K, 220 nm): t_{R1} = 5.6 min, t_{R2} = 6.9 min, er = 96.5:3.5.

$[\alpha]_D^{25}$ = –29.0 (*c* 0.95, $CHCl_3$).

(*S*)-1-cyclohexyl-3,3-dimethyl-5-methylene-1-propylsilinane (2p)

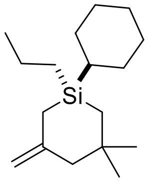

The reaction was conducted on a 0.2 mmol scale. The titled product was purified by column chromatography with hexanes as eluent to afford **2p** as a colorless oil (34.4 mg, 65% yield)

^{1}H NMR (501 MHz, CD_2Cl_2) δ 4.62 (dt, J = 2.8, 1.3 Hz, 1H), 4.49 (d, J = 2.9 Hz, 1H), 1.94–1.85 (m, 2H), 1.74–1.65 (m, 5H), 1.52–1.49 (m, 2H), 1.39–1.32 (m, 2H), 1.24–1.09 (m, 5H), 0.99–0.93 (m, 9H), 0.72 (tt, J = 12.4, 3.0 Hz, 1H), 0.60–0.53 (m, 4H).

^{13}C NMR (126 MHz, CD_2Cl_2) δ 147.3, 108.9, 52.5, 34.4, 34.2, 31.3, 28.7, 28.0, 28.0, 27.5, 25.0, 23.2, 22.4, 18.8, 18.1, 14.9.

EI-HRMS (*m/z*): calculated for $C_{17}H_{32}Si_1$ [$M^{+\bullet}$]: 264.2264, found: 264.2268.

GC (24.0 m Cyclodextrin-H 176, injection temperature: 220 °C, 70 °C iso 350 min, 0.5 bar H_2): t_{R1} = 337.2 min, t_{R2} = 355.3 min, er = 57.6:42.4.

$[\alpha]_D^{25}$ = –1.6 (*c* 1.03, $CHCl_3$).

5. Solvent effect

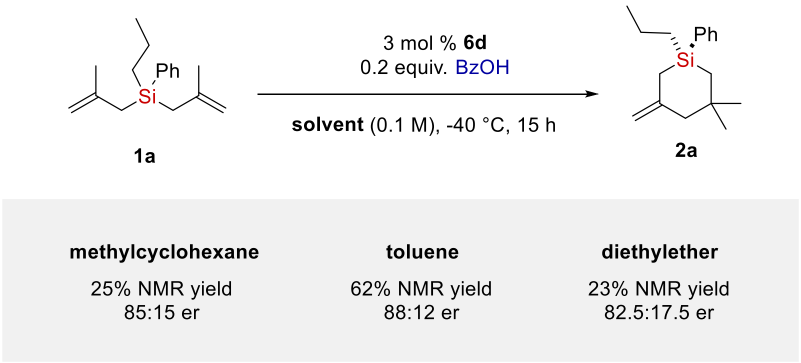

Supplementary Figure 1. Solvent effect. NMR yield was determined by ^{1}H NMR analysis with dibromomethane as an internal standard.

6. Control experiments

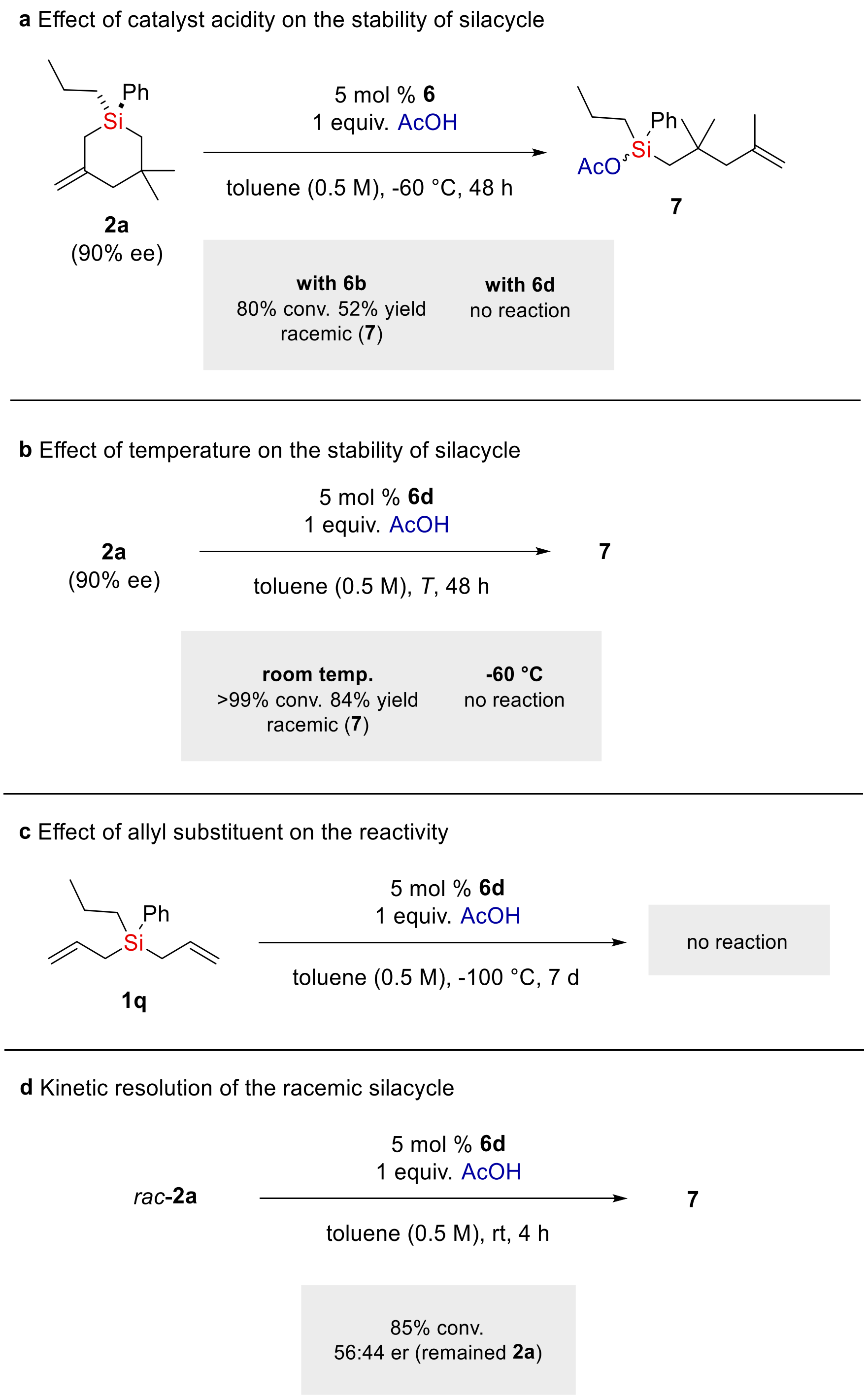

Supplementary Figure 2. Control experiments. a effect of catalyst acidity on the stability of silacycle. **b** effect of temperature on the stability of silacylce. **c** effect of allyl substituent on the reactivity. **d** kinetic resolution of the racemic silacycle.

Procedure for the experiment **A**: A GC vial was charged with catalyst **6b** (5 mol %), toluene and acetic acid (1 equiv.), and the resulting mixture was cooled to -60 °C in a cryostat. After 10 min, enantioenriched silacycle **2a** (0.1 mmol) was slowly added and the GC vial was stored for 48 h at the same temperature. The reaction was quenched with trimethylamine. The solvent was removed *in vacuo* and

the mixture was purified by preparative TLC on silica gel. During the purification, silyl ester **7** was partially converted to the silanol **7’** (see ^{1}H NMR spectrum below). *Preparative TLC on silica gel was imperative because the silyl ester* ***7*** *was completely converted to the silanol 7’ when it was purified by column chromatography on silica gel.* When catalyst **6d** was used instead of **6b**, the reaction did not take place and starting material **2a** remained intact.

^{1}H NMR (after purification by preparative TLC)

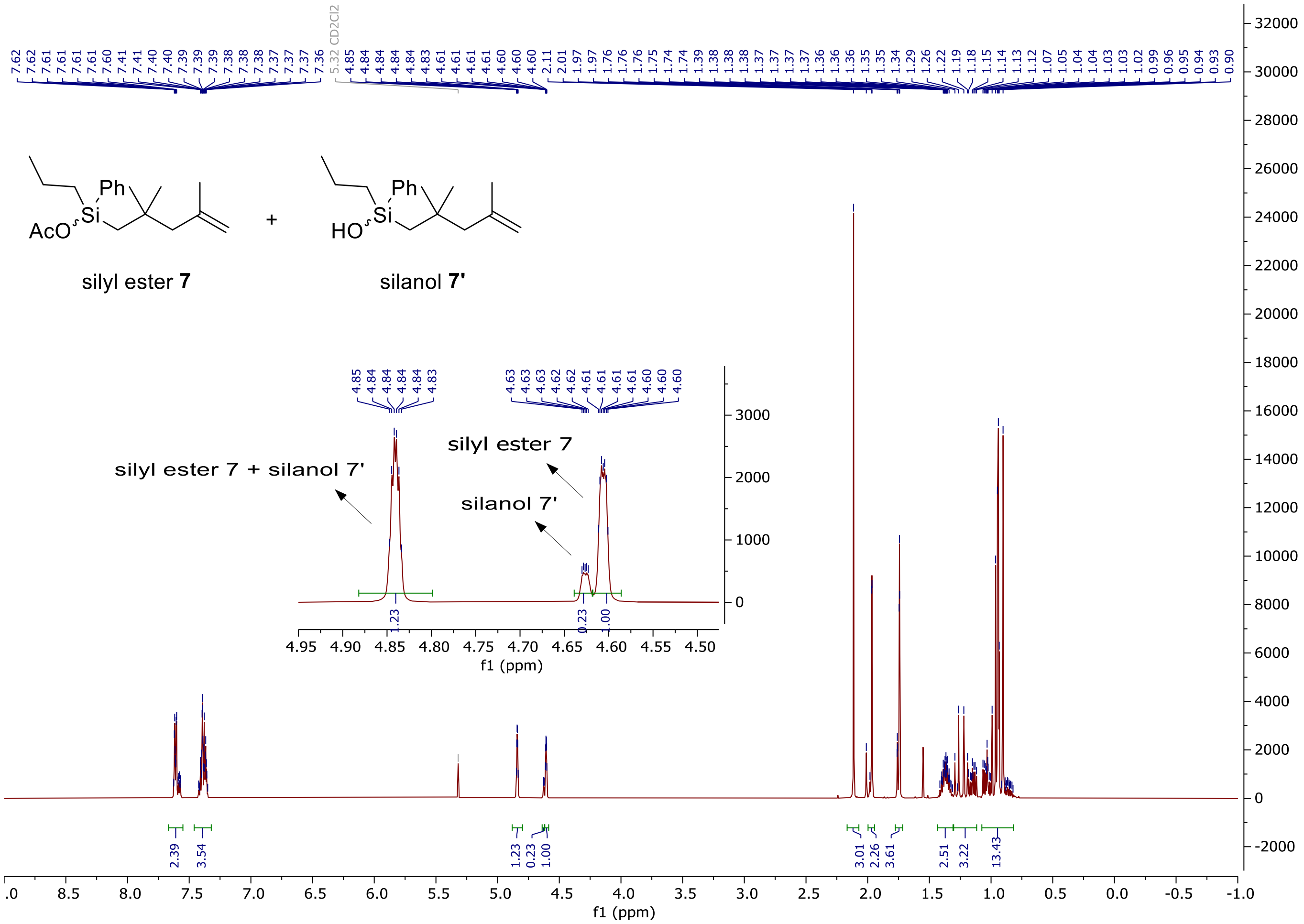

phenyl(propyl)(2,2,4-trimethylpent-4-en-1-yl)silanol (7’)

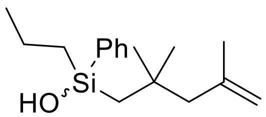

^{1}H NMR (501 MHz, CD_2Cl_2) δ 7.63–7.52 (m, 2H), 7.36 (dd, *J* = 4.9, 1.9 Hz, 3H), 4.84 (dq, *J* = 3.0, 1.5 Hz, 1H), 4.62 (d, *J* = 2.6 Hz, 1H), 2.01 (s, 2H), 1.95 (brs, 1H), 1.76 (s, 3H), 1.43–1.30 (m, 2H), 1.03 (d, *J* = 2.6 Hz, 2H), 0.99 (s, 3H), 0.97–0.92 (m, 6H), 0.90–0.80 (m, 2H).

^{13}C NMR (126 MHz, CD_2Cl_2) δ 144.6, 139.8, 133.8, 129.5, 128.1, 114.3, 53.9, 34.5, 31.3, 30.8, 25.6, 20.5, 18.4, 17.2.

ESI-HRMS (*m/z*): calculated for $C_{17}H_{28}O_1Si_1Na_1$ ($[M+Na]^+$): 299.1801, found: 299.1802.

Procedure for the experiment **B**: A GC vial was charged with catalyst **6d** (5 mol %), toluene and acetic acid (1 equiv.). After 10 min, enantioenriched silacycle **2a** (0.1 mmol) was slowly added and the GC vial was stored for 48 h at room temperature. The reaction was

quenched with trimethylamine. The solvent was removed *in vacuo* and the mixture was purified by preparative TLC on silica gel. During the purification, silyl ester **7** was partially converted to the silanol **7'**. *Preparative TLC on silica gel was imperative because the silyl ester **7** was completely converted to the silanol 7' when it was purified by column chromatography on silica gel*. When the reaction was performed at -60 °C, the reaction did not take place and starting material **2a** remained intact.

Procedure for the experiment **C**: A GC vial was charged with catalyst **6d** (5 mol %), toluene and acetic acid (1 equiv.), and the resulting mixture was cooled to -100 °C in a cryostat. After 10 min, bis(allyl)silane **1p** (0.025 mmol) was slowly added and the GC vial was stored for 7 days at the same temperature. The reaction did not take place and starting material **1p** remained intact.

Procedure for the experiment **D**: A GC vial was charged with catalyst **6d** (5 mol %), toluene and acetic acid (1 equiv.). After 10 min, racemic silacycle **2a** (0.1 mmol) was added and the GC vial was stored for 4 h at room temperature. The reaction was quenched with trimethylamine. The solvent was removed *in vacuo* and the mixture was purified by preparative TLC on silica gel.

7. NMR experiments

For the NMR experiment shown in Scheme 4:

Catalyst **6c** (13 mg, 0.0078 mmol) and dry toluene-d_8 (0.5 mL) were added to NMR tube (5 mm diameter) at room temperature and the NMR tube was then inserted into the NMR spectrometer (step 1). After the first ^{31}P-NMR measurement, silane **1a** (0.078 mmol, 10 equiv.) was added at room temperature. The second ^{31}P-NMR measurement was then promptly performed (step 2). Subsequently, acetic acid (0.078 mol, 10 equiv.) was added at room temperature. Then, the third ^{31}P-NMR measurement was conducted immediately (step 3).

For the additional NMR experiments:

Catalyst **6c** (13 mg, 0.0078 mmol) and dry toluene-d_8 (0.5 mL) were added to NMR tube (5 mm diameter) at room temperature and the NMR tube was then inserted into the NMR spectrometer (step 1). After the first ^{31}P-NMR measurement, acetic acid (0.078 mmol, 10 equiv.) was added at room temperature. Then, the second ^{31}P-NMR measurement was conducted immediately. Although the resulting mixture was monitored by ^{31}P-NMR for 24 h, a complexation between catalyst **6c** and acetic acid was not observed.

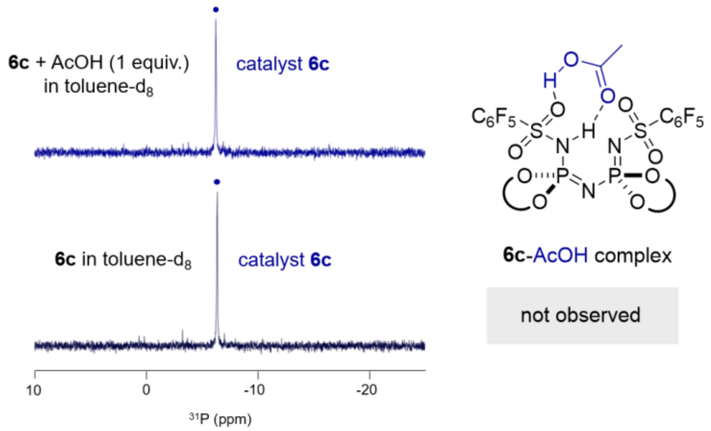

Supplementary Figure 3. ^{31}P NMR experiment with catalyst **6c** and AcOH.

Catalyst **6c** (10 mg, 0.0062 mmol), acetic acid (40 equiv. 0.25 mmol) and dry toluene-*d*$_8$ (0.5 mL) were added to NMR tube (5 mm diameter) which was treated with dry-ice bath in ethanol (-78 °C). After 10 min, bis(methallyl)silane **1a** (40 equiv. 0.25 mmol) was added and the NMR tube was then inserted into the NMR spectrometer. The NMR tube was kept at -60 °C for 24 h, in which ^{1}H-NMR spectra were taken at different time points.

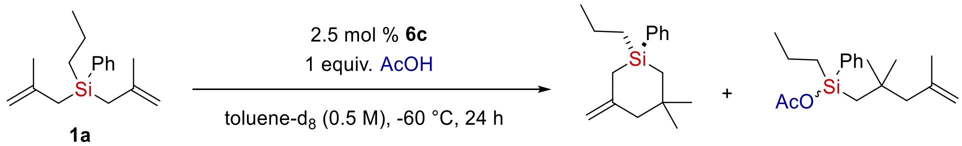

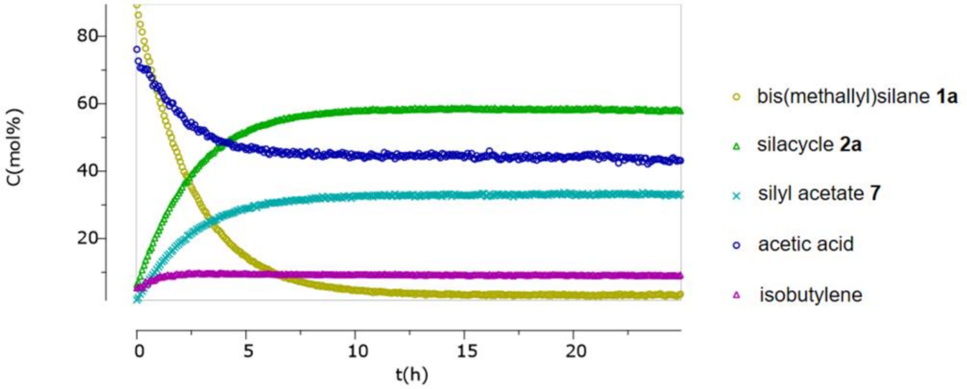

Supplementary Figure 4. Reaction progress monitored by in situ ^{1}H NMR (with AcOH).

Catalyst **6c** (10 mg, 0.0062 mmol) and dry toluene-*d*$_8$ (0.5 mL) were added to NMR tube (5 mm diameter) which was treated with dry-ice bath in ethanol (-78 °C). After 10 min, bis(methallyl)silane **1a** (40 equiv. 0.25 mmol) was added and the NMR tube was then inserted into the NMR spectrometer. The NMR tube was kept at -60 °C for 24 h, in which ^{1}H-NMR spectra were taken at different time points.

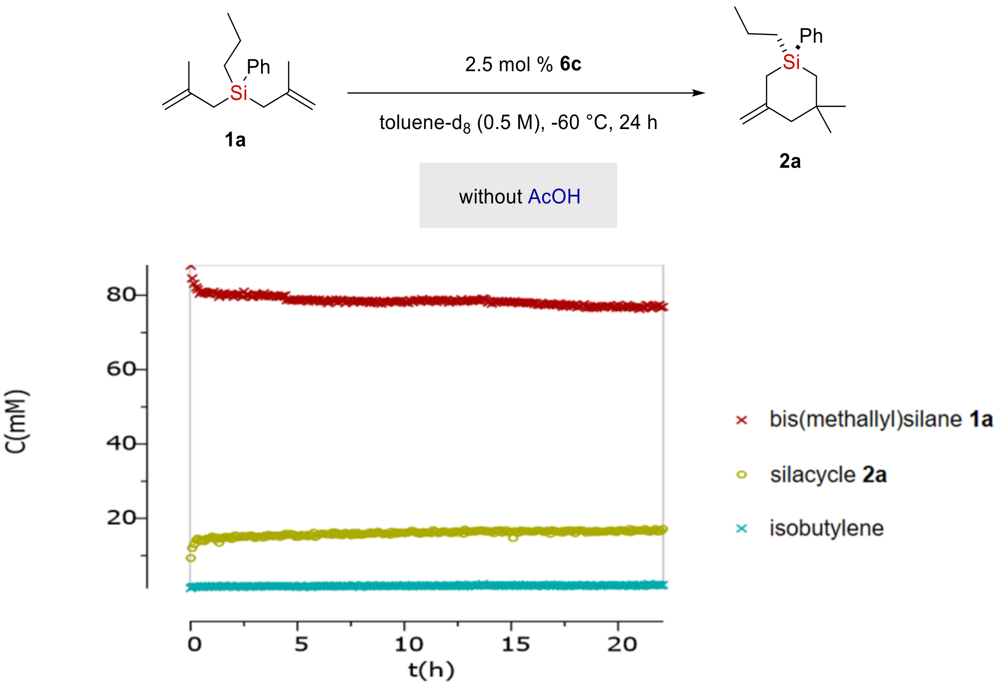

Supplementary Figure 5. Reaction progress monitored by in situ ^{1}H NMR (without AcOH).

8. ESI-MS experiments

Catalyst **6c** (13 mg, 0.0078 mmol) and dry toluene-d_8 (0.5 mL) were added to a GC vial at room temperature. Subsequently, silane **1a** (0.078 mmol, 10 equiv.) was added. The reaction mixture was then injected to electrospray ionization spectrometry (ESI-MS).

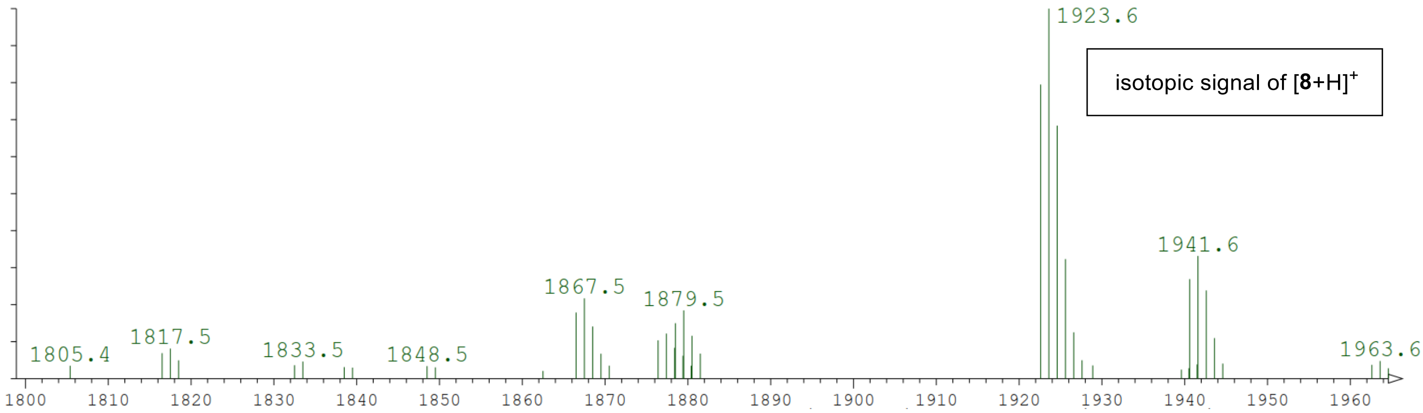


```
Mass to be matched (m/z): 1922.60247  charge: 1

Mass tolerance: ±0.005
restriction of atom numbers:
      C       H       N       O       S       P       F      Si
   1-150   1-100    1-3    0-10     2-2     2-2   10-10     1-1
Number of calculated formulas: 3
Formula                          Diff. (ppm)  theor. m/z
C109 H100 F10 N3 O8 P2 S2 Si1          0.34   1922.60312
C121 H96 F10 N1 O1 P2 S2 Si1          -0.62   1922.60127
C114 H100 F10 N1 O6 P2 S2 Si1          2.43   1922.60714
```

```
Suggestion:
C109H99F10N3O8P2S2Si1
MW 1921

characteristical ion
1922 = [1921 + H]+
```

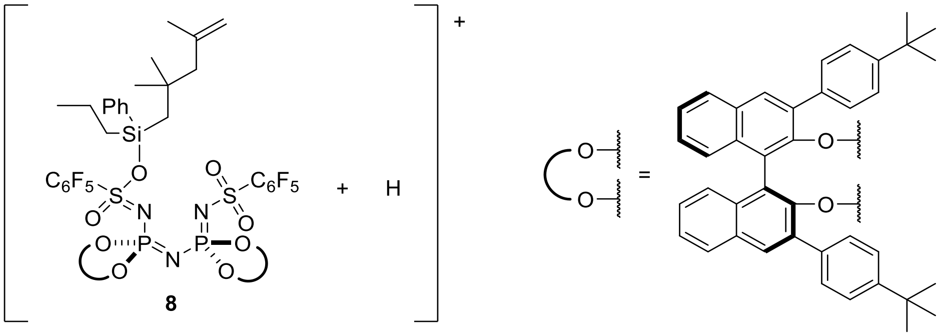

[8+H]+
formula: $C_{109}H_{100}F_{10}N_3O_8P_2S_2Si_1$

9. Determination of the absolute configuration of silacycle 2a

9.1. Mosher ester analysis

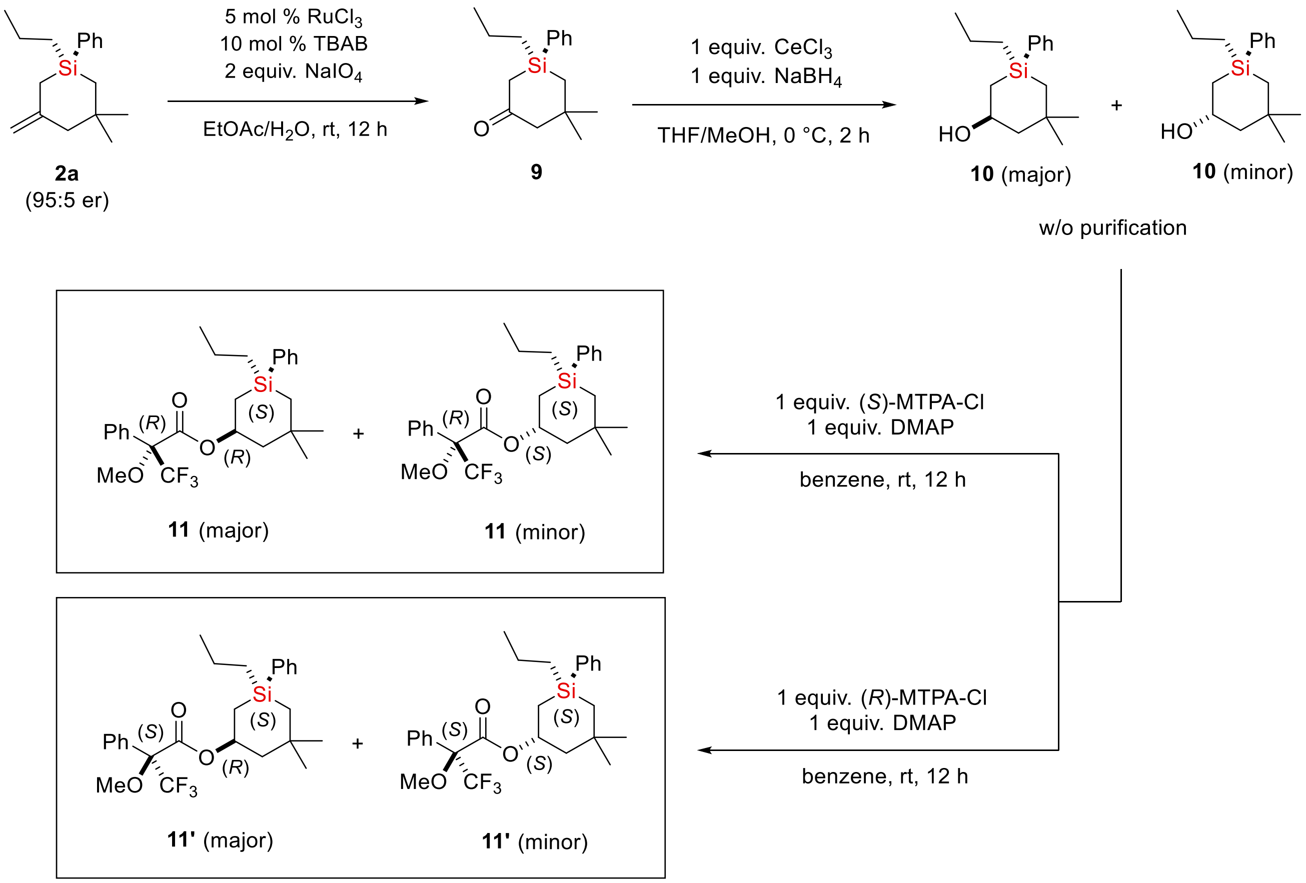

Supplementary Figure 6. Synthesis of Mosher esters from enantioenriched silacycle **2a**.

Procedure for the oxidation of **2a**: To a solution of **2a** (0.67 mmol) in H_2O (2 mL) and EtOAc (2 mL), $RuCl_3$ (5 mol %), $NaIO_4$ (2 equiv.) and TBAB (10 mol %) were added at room temperature. The resulting mixture was stirred for 12 h at the same temperature. To the reaction mixture, H_2O was added and extracted with EtOAc. The combined organic phase were dried over Na_2SO_4, filtrated and dried under reduced pressure. Purification of the mixture by preparative TLC on silica gel afforded the desired ketone **9** as a colorless oil (40.0

mg, 23% yield). *Preparative TLC on silica gel was imperative because the ketone **9** was completely decomposed when it was purified by column chromatography on silica gel.*

Procedure for the reduction of **9**: To a solution of **9** (0.15 mmol) in THF (1 mL) and MeOH (0.5 mL), $CeCl_3$ (1 equiv.) and $NaBH_4$ were added were added subsequently at 0 °C. The resulting mixture was stirred for 2 h at the same temperature. After complete conversion indicated by TLC, sat. NH_4Cl was added to the mixture and extracted with EtOAc. The combined organic phase were dried over Na_2SO_4, filtrated and dried under reduced pressure. The crude mixture was used for the next step without further purification.

Procedure for the esterification of **10**: **9** (0.075 mmol) and benzene (0.2 mL) were placed in a flame-dried Schlenk flask equipped with a Teflon-coated magnetic stirring bar. DMAP (1 equiv.) and (*S*)-MTPA-Cl (1 equiv.) were added and the resulting mixture was stirred 12 h. After complete conversion indicated by TLC, the solvent was removed under reduced pressure. Purification of the mixture by preparative TLC on silica gel (25 mm) afforded the desired (*R*)-Mosher ester **11** as a colorless oil (17.9 mg, 50% yield, 1.25:1 dr). With the same procedure, the (*S*)-Mosher ester **11'** was prepared as a colorless oil (16.5 mg, 46% yield, 1.35:1 dr).

Based on the observed couplings (2 large ^3J~12.5 Hz (*trans*) and 2 smaller ones ^3J~3.8 Hz (*cis*)) H-2 is mostly in the axial position in the ring and the OR group in the equatorial position. This enabled the simple differentiation of the two diastereomers based on NOE from H-2 and H-6 to H-8 (major) and a NOE from H-2 and H-6 to H-12 (minor). Mosher ester analysis (see page S29 for details) lead to the conclusion that the stereoconfiguration is (*R*) at C-2 for the major and (*S*) for the minor diastereomer. Based on the NOE analysis for the determination of of the diastereomers, this suggests that the stereoconfiguration is (*S*) at Si-100 in both cases.

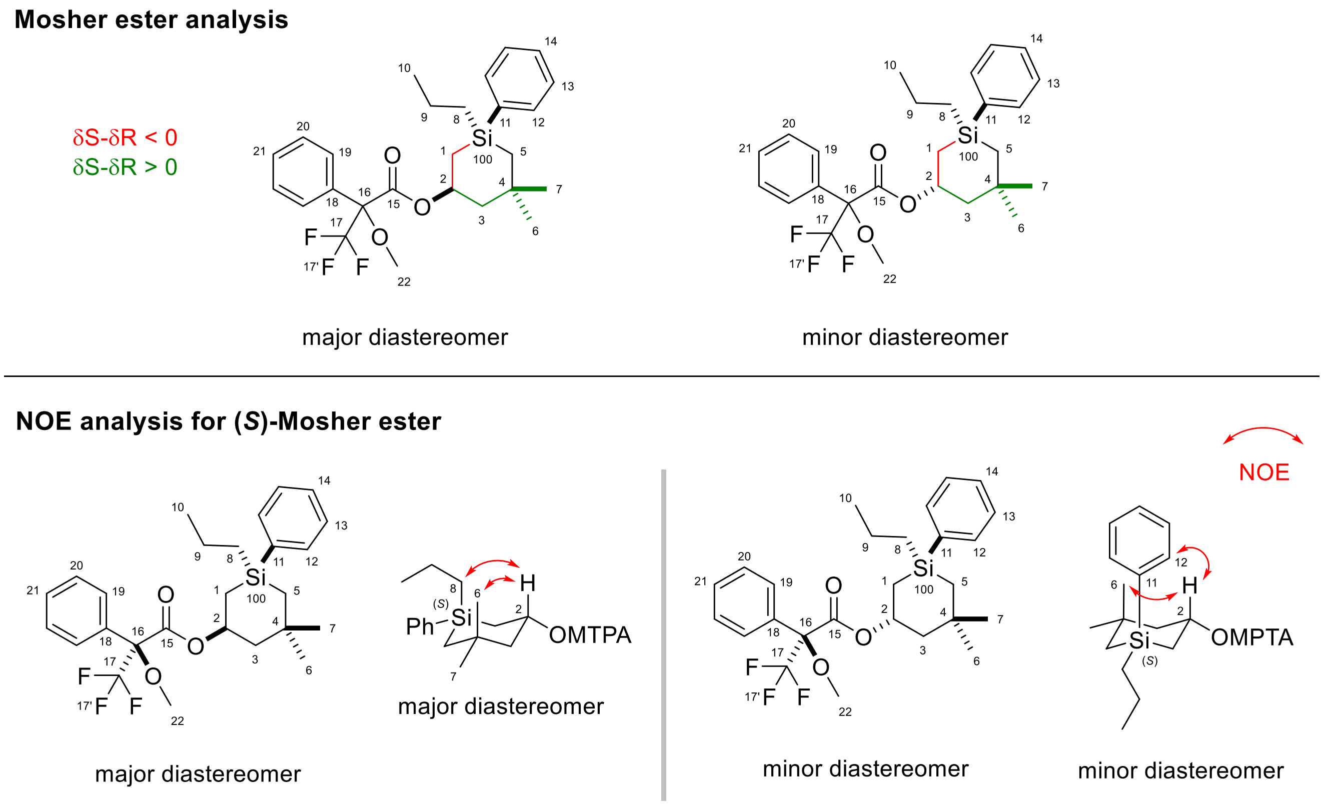

Supplementary Figure 7. Determination of the absolute configuration by Mosher ester and NOE analysis.

(1*S*,3*R*)-5,5-dimethyl-1-phenyl-1-propylsilinan-3-yl (*R*)-3,3,3-trifluoro-2-methoxy-2-phenylpropanoate (11)

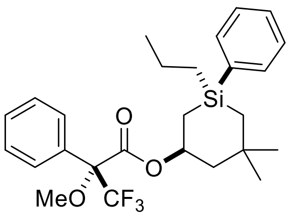

^{1}H NMR (501 MHz, $CDCl_3$) Major diastereomer δ 7.62–7.53 (m, 3H), 7.46–7.4 (m, 7H), 5.46 (dddd, *J* = 12.8, 11.9, 4.1, 3.2 Hz, 1H), 3.58 (d, *J* = 1.1 Hz, 3H), 1.72–1.65 (m, 2H), 1.44–1.36 (m, 3H), 1.14–0.86 (m, 12H), 0.71–0.69 (m, 2H). Minor diastereomer δ 7.62–7.53 (m, 3H), 7.46–7.4 (m, 7H), 5.39–5.33 (m, 1H), 3.59–3.59 (m, 3H), 1.91–1.89 (m, 1H), 1.72–1.65 (m, 1H), 1.49 (t, *J* = 12.3 Hz, 1H), 1.30–1.20 (m, 4H), 1.14–0.86 (m, 8H), 0.71–0.69 (m, 1H), 0.68–0.64 (m, 2H), 0.61 (d, *J* = 14.9 Hz, 1H).

^{13}C NMR (126 MHz, $CDCl_3$) Major diastereomer: δ 166.1, 137.1, 133.7, 132.7, 129.6, 129.4, 128.5, 128.1, 127.5 (q, *J* = 1.2 Hz), 123.5 (q, *J* = 288.4 Hz), 84.6 (q, *J* = 27.4 Hz), 75.3, 55.5, 47.3, 37.6, 33.3, 27.9, 24.3, 19.6, 18.4, 17.7, 17.4. Minor diastereomer: δ 166.1, 136.9, 133.8, 132.7, 129.6, 129.4, 128.5, 128.2, 127.5 (q, *J* = 1.2 Hz), 123.6 (q, *J* = 288.4 Hz), 84.6, 75.4, 55.5, 47.6, 37.5, 33.5, 28.1, 24.4, 18.5, 18.2, 17.7, 17.2.

^{19}F NMR (471 MHz, $CDCl_3$) δ -71.6.

ESI-HRMS (*m/z*): calculated for $C_{26}H_{33}F_3O_3Si_1Na_1$ $[M+Na]^+$: 501.2041, found: 501.2043.

(1*S*,3*R*)-5,5-dimethyl-1-phenyl-1-propylsilinan-3-yl (*S*)-3,3,3-trifluoro-2-methoxy-2-phenylpropanoate (11')

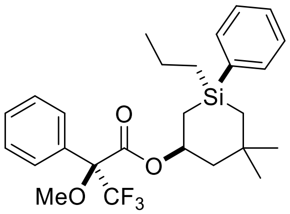

^{1}H NMR (501 MHz, $CDCl_3$) Major diastereomer δ 7.61–7.55 (m, 3H), 7.44–7.32 (m, 7H), 5.45 (tt, *J* = 12.3, 3.7 Hz, 1H), 3.58–3.58 (m, 3H), 1.80 (dq, *J* = 12.8, 2.4 Hz, 1H), 1.63–1.59 (m, 1H), 1.50 (t, *J* = 12.4 Hz, 1H), 1.41–1.35 (m, 2H), 1.30–1.22 (m, 3H), 1.15 (s, 3H), 1.11 (s, 3H), 0.98–0.95 (m, 3H), 0.71–0.66 (m, 2H). Minor diastereomer δ 7.61–7.55 (m, 3H), 7.44–7.32 (m, 7H), 5.35 (tt, *J* = 12.2, 3.7 Hz, 1H), 3.60–3.60 (m, 3H), 1.98–1.92 (m, 1H), 1.63–1.59 (m, 1H), 1.44–1.40 (m, 1H), 1.30–1.22 (m, 2H), 1.06–1.03 (m, 3H), 0.98–0.95 (m, 3H), 0.98–0.95 (m, 3H), 0.88 (t, *J* = 7.3 Hz, 2H), 0.71–0.66 (m, 2H), 0.61 (d, *J* = 14.9 Hz, 1H).

^{13}C NMR (126 MHz, $CDCl_3$) Major diastereomer δ 166.1, 137.1, 133.7, 132.7, 129.6, 129.4, 128.5, 128.1, 127.5 (q, *J* = 1.2 Hz), 123.5 (q, *J* = 288.4 Hz), 84.6 (q, *J* = 27.4 Hz), 75.3, 55.5, 47.6, 37.6, 33.4, 28.0, 24.4, 19.2, 18.4, 17.7, 17.4. Minor diastereomer δ 166.1, 136.9, 133.9, 132.8, 129.6, 129.4, 128.5, 128.2, 127.6 (q, *J* = 1.2 Hz), 123.6 (q, *J* = 288.4 Hz), 84.6 (q, *J* = 27.4 Hz), 75.3, 55.5, 47.3, 37.5, 33.5, 28.0, 24.3, 18.6, 18.2, 18.1, 17.2.

^{19}F NMR (471 MHz, $CDCl_3$) Major diastereomer δ -71.7. Minor diastereomer δ -71.6.

ESI-HRMS (*m/z*): calculated for $C_{26}H_{33}F_3O_3Si_1Na_1$ $[M+Na]^+$: 501.2041, found: 501.2043.

For each diastereomer of **11**, diastereomeric ratio (dr) was determined by ^{19}F NMR analysis; 13.3:1 dr for major diastereomer of **11**, 15.7:1 dr for minor diastereomer of **11**. These results indicate that enantiomeric excess (ee) of the *Si*-stereogenic silacycle was decreased by ~3% during the three step derivatizations (from **2a** to **11**).

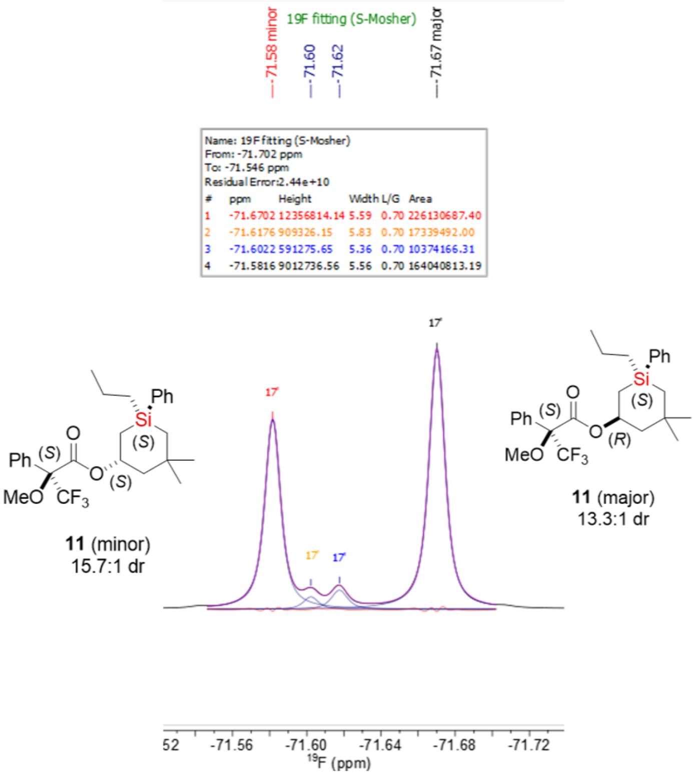

Supplementary Figure 8. Determination of diastereomeric ratio of Mosher ester **11** by ^{19}F NMR analysis.

NMR data of 11

User Report
HBD-JB-226-02
(*R*-Mosher-Ester)

The sample contains the following 2 diastereomers:

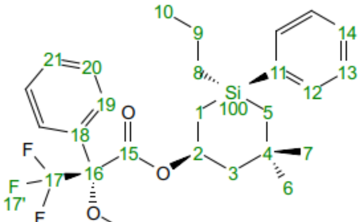

major

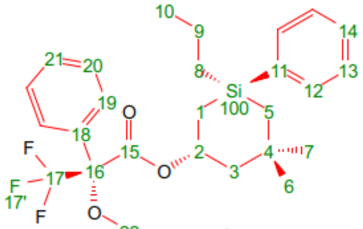

minor

Remarks:
These are the *R*-Mosher-ester derivatives.
Based on the observed couplings H-2 is always in axial position in the ring. This enabled the differentiation of the 2 diasteromers based on an NOE from H-2 to H-8 (major) and an NOE from H-2 to H-12 (minor)

For a more detailed discussion and the final Mosher analysis for the determination of the absolute stereochemistry, please see HBD-JB-230-01.

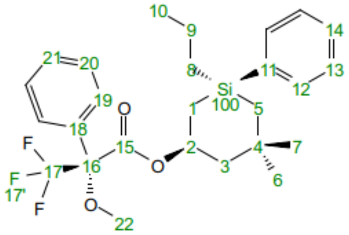

Atom	J	δ (ppm)	HSQC	COSY	HMBC	NOESY
1 C		19.559	1ax, 1eq		3ax, 3eq, 5eq	
Hax	12.90(1eq), 12.80(2)	1.058	1	1eq, 2	2, 3, 5, 100	1eq, 12
Heq	12.90(1ax)	1.667	1	1ax, 2, 3eq	2, 3, 5	1ax, 2, 12
2 C		75.251	2		1ax, 1eq, 3ax	
H	12.80(1ax), 11.90(3ax), 4.10(?), 3.20(?)	5.459	2	1ax, 1eq, 3ax, 3eq	3, 15	1eq, 3eq, 6, 8
3 C		47.313	3ax, 3eq		1ax, 1eq, 2, 5ax, 5eq, 6, 7	
Hax	12.80(3eq), 11.90(2)	1.421	3	2, 3eq	1, 2, 4, 5, 6, 7	3eq
Heq	12.80(3ax)	1.705	3	1eq, 2, 3ax, 5eq	1, 4, 5, 6	2, 3ax, 6, 7
4 C		33.285			3ax, 3eq, 5ax, 5eq, 6, 7	
5 C		24.299	5ax, 5eq		1ax, 1eq, 3ax, 3eq, 6, 7	
Hax	14.50(5eq)	0.702	5	5eq, 6	3, 4, 6, 7, 8, 100	5eq, 12
Heq	14.50(5ax)	0.987	5	3eq, 5ax	1, 3, 4, 6, 7, 8, 11	5ax, 12
6 C		27.941	6		3ax, 3eq, 5ax, 5eq, 7	
H3		1.138	6	5ax	3, 4, 5, 7	2, 3eq, 8
7 C		37.547	7		3ax, 5ax, 5eq, 6	
H3		1.088	7		3, 4, 5, 6, 100	3eq
8 C		17.368	8		5ax, 5eq, 9, 10	
H2		0.970	8	9	9, 10, 11, 100	2, 6, 12
9 C		17.704	9		8, 10	
H2	7.20(10)	1.388	9	8, 10	8, 10	12
10 C		18.418	10		8, 9	
H3	7.20(9)	0.969	10	9	8, 9	

Atom	J	δ (ppm)	HSQC	COSY	HMBC	NOESY
11 C		137.085			5eq, 8, 12, 13	
12 C		133.733	12		12, 14	
H		7.446	12	13	11, 12, 14, 100	1ax, 1eq, 5ax, 5eq, 8, 9
13 C		128.077	13		13	
H		7.354	13	12, 14	11, 13	
14 C		129.437	14		12	
H		7.364	14	13	12	
15 C		166.085			2	
16 C	27.40(17')	84.558			19, 22	
17 C	288.60(17')	123.538				
17' F	288.60(17), 1.10(22H), 1.40(22), 27.40(16), 1.20(19H)	-71.618				
18 C		132.698			20	
19 C		127.522	19		21	
H	1.20(17'F)	7.568	19	20	16, 21	
20 C		128.487	20		20	
H		7.406	20	19, 21	18, 20	
21 C		129.614	21		19	
H		7.406	21	20	19	
22 C	1.40(17')	55.500	22			
H3	1.10(17'F)	3.578	22		16	
100 Si		-4.970			1ax, 5ax, 7, 8, 12	

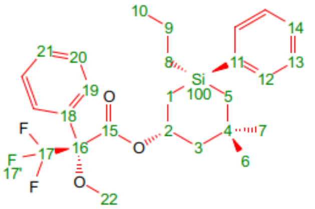

Atom	J	δ (ppm)	HSQC	COSY	HMBC	NOESY
1 C		17.698	1ax, 1eq		3ax, 5eq	
Hax	13.40(1eq), 12.50(2)	0.864	1	1eq, 2	2, 3, 5, 100	1eq
Heq	13.40(1ax)	1.898	1	1ax, 2, 3eq	2, 3, 5	1ax, 2, 12
2 C		75.357	2		1ax, 1eq, 3ax, 3eq	
H	12.50(1ax), 12.20(3ax)	5.359	2	1ax, 1eq, 3ax, 3eq	3, 15	1eq, 3eq, 6, 12
3 C		47.553	3ax, 3eq		1ax, 1eq, 2, 5ax, 5eq, 6, 7	
Hax	12.60(3eq), 12.20(2)	1.495	3	2, 3eq	1, 2, 4, 5, 6, 7	3eq
Heq	12.60(3ax)	1.700	3	1eq, 2, 3ax, 5eq	2, 4, 5, 6	2, 3ax, 6, 7
4 C		33.502			3ax, 3eq, 5ax, 5eq, 6, 7	
5 C		24.364	5ax, 5eq		1ax, 1eq, 3ax, 3eq, 6, 7	
Hax	14.90(5eq)	0.607	5	5eq, 6	3, 4, 6, 7, 100	5eq
Heq	14.90(5ax)	1.038	5	3eq, 5ax	1, 3, 4, 6, 7, 8, 11	5ax, 12
6 C		28.047	6		3ax, 3eq, 5ax, 5eq, 7	
H3		0.700	6	5ax	3, 4, 5, 7	2, 3eq, 8, 12
7 C		37.510	7		3ax, 5ax, 5eq, 6	
H3		1.063	7		3, 4, 5, 6, 100	3eq
8 C		18.521	8		5eq, 9, 10	
H2		0.660	8	9	10, 11, 100	6
9 C		17.166	9		10	
H2		1.251	9	8, 10	8, 10	
10 C		18.186	10		8, 9	
H3		0.870	10	9	8, 9	

Atom	J	δ (ppm)	HSQC	COSY	HMBC	NOESY
11 C		136.943			5eq, 8, 12, 13	
12 C		133.845	12		12, 14	
H		7.600	12	13	11, 12, 14, 100	1eq, 2, 5eq, 6
13 C		128.230	13		13	
H		7.385	13	12, 14	11, 13	
14 C		129.354	14		12	
H		7.385	14	13	12	
15 C		166.058			2	
16 C	27.40(17')	84.630			19, 22	
17 C	288.40(17')	123.530				
17' F	288.40(17), 1.10(22), 27.40(16), 1.20(19H)	-71.603				
18 C		132.698			20	
19 C		127.543	19		21	
H	1.20(17'F)	7.567	19	20	16, 21	
20 C		128.501	20		20	
H		7.420	20	19, 21	18, 20	
21 C		129.626	21		19	
H		7.420	21	20	19	
22 C	1.10(17')	55.540	22			
H3		3.588	22		16	
100 Si		-4.152			1ax, 5ax, 7, 8, 12	

^{1}H NMR spectrum of 11

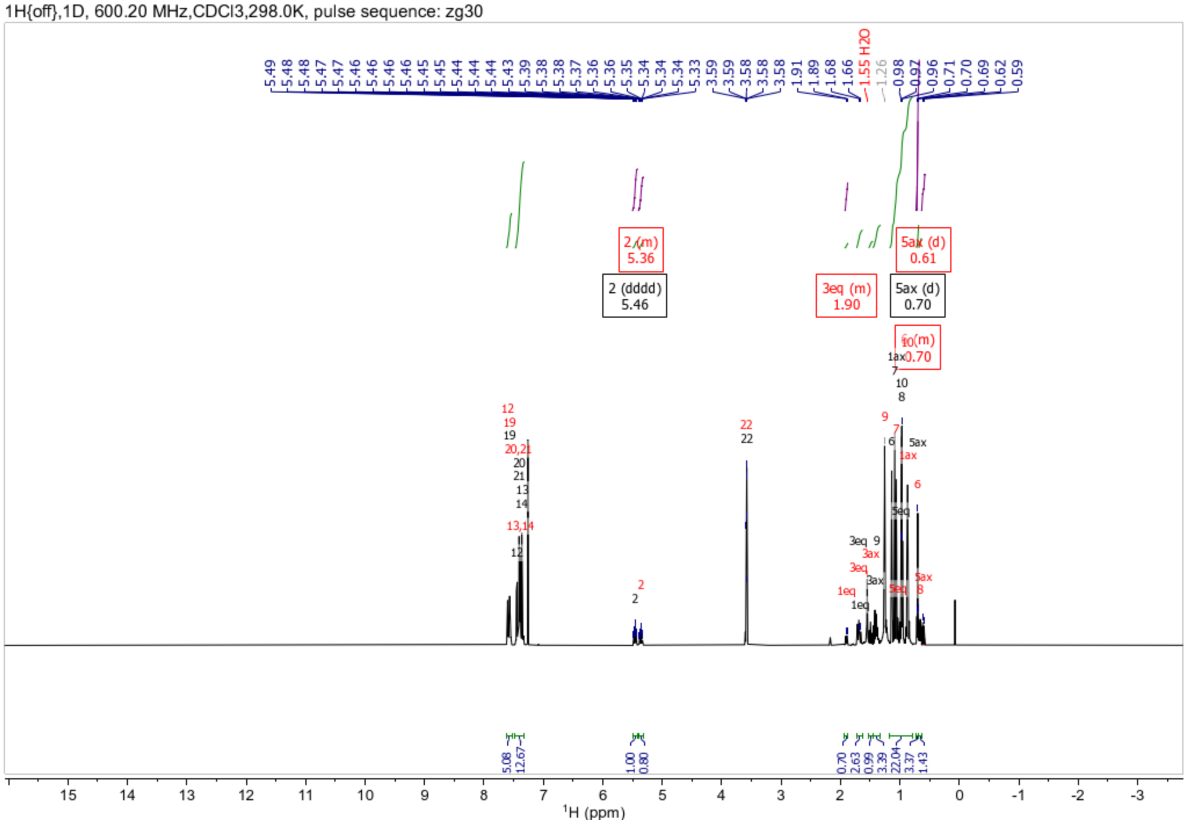

^{13}C NMR spectrum of 11

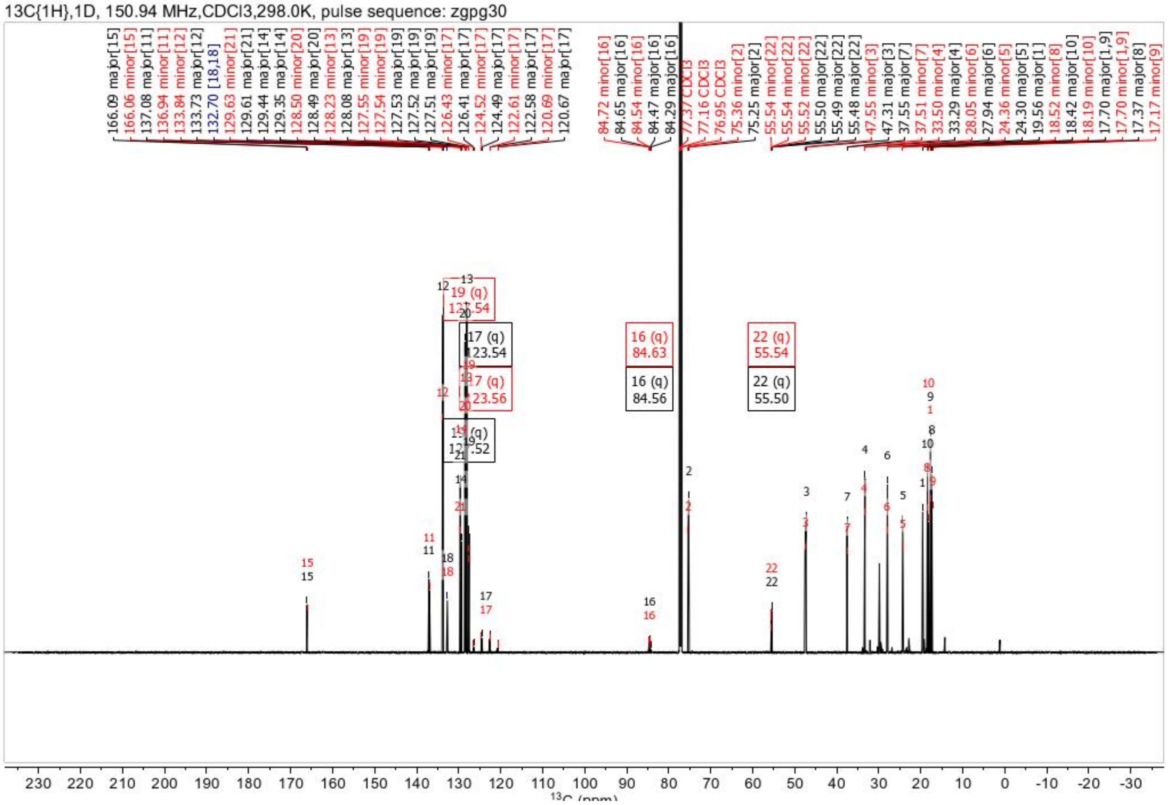

^{1}H-^{13}C HSQC NMR spectrum of 11

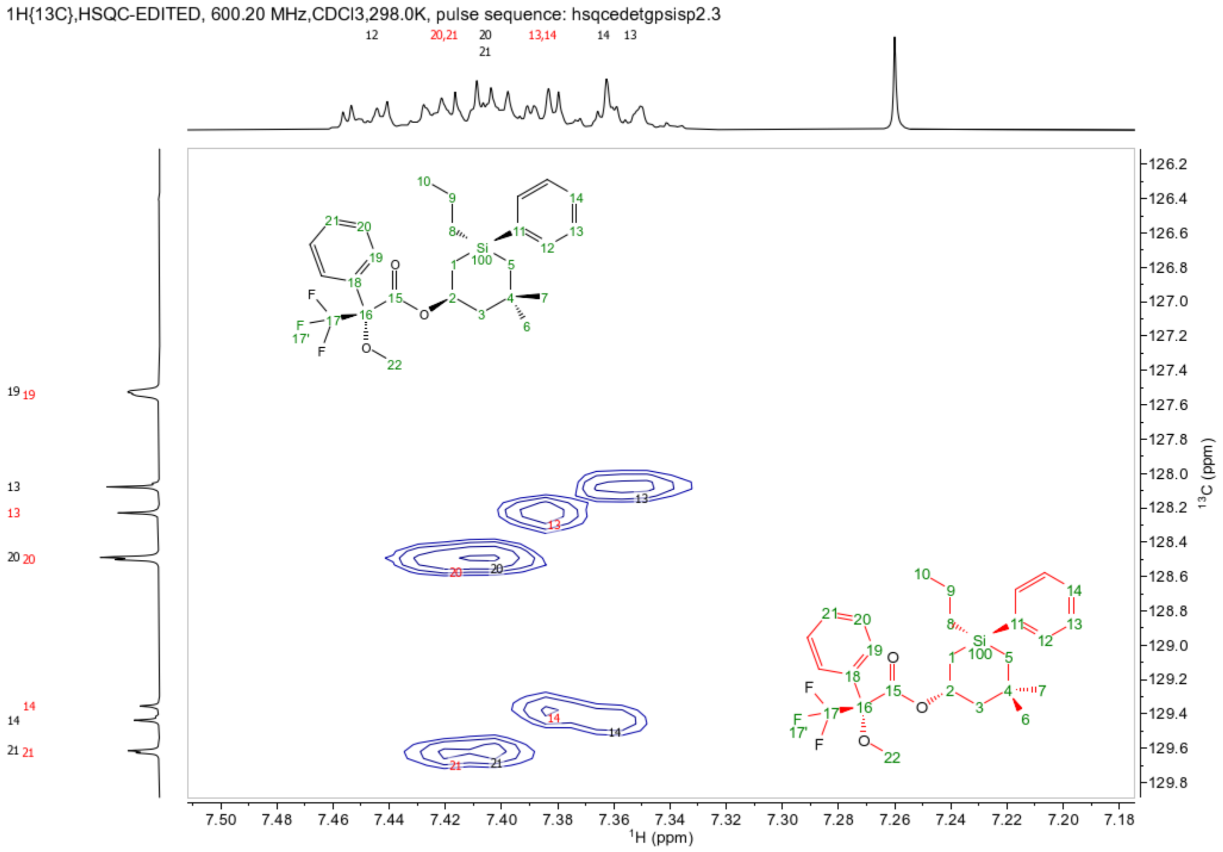

^{1}H-^{13}C HMBC NMR spectrum of 11

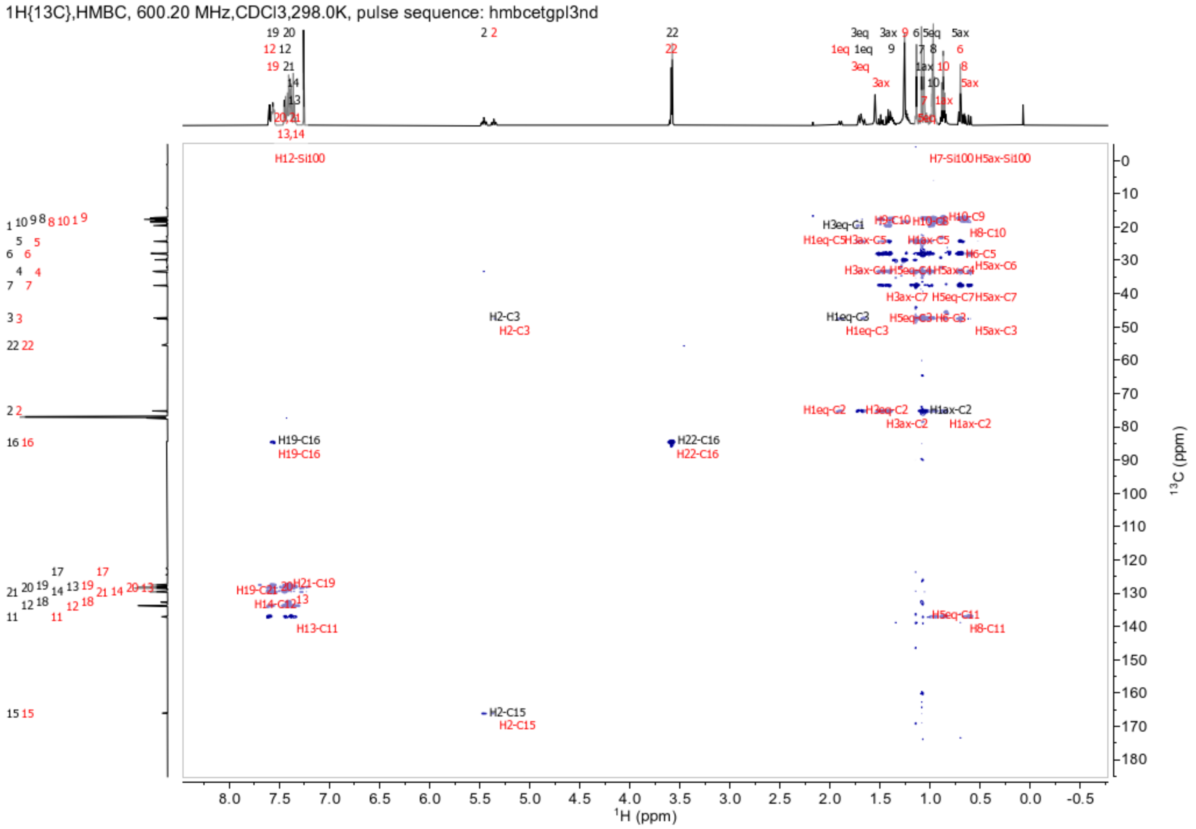

^{1}H COSY NMR spectrum of 11

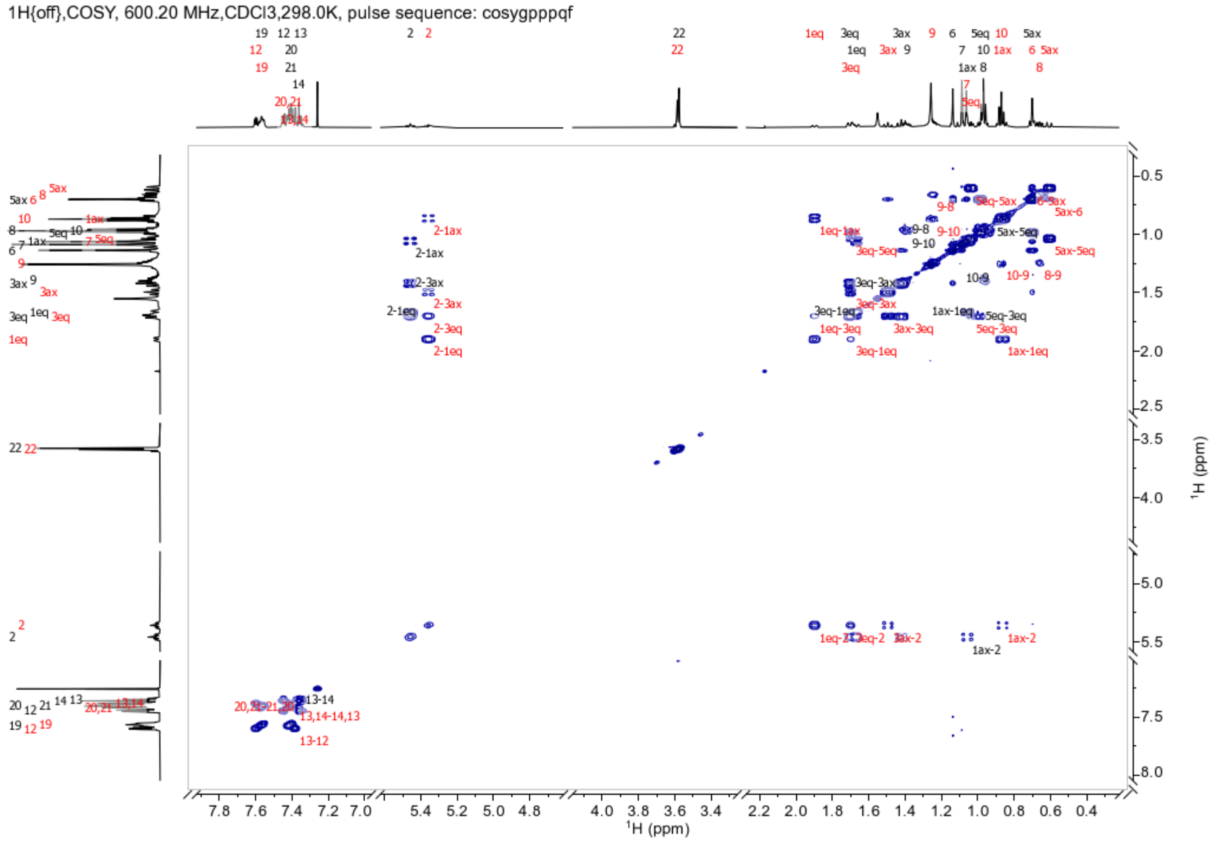

^{29}Si NMR and ^{1}H-^{29}Si HMBC NMR spectra of 11

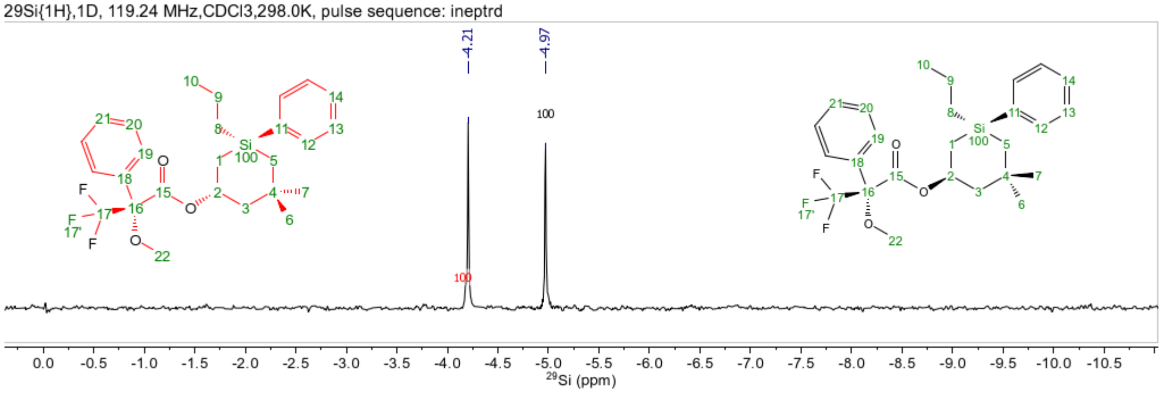

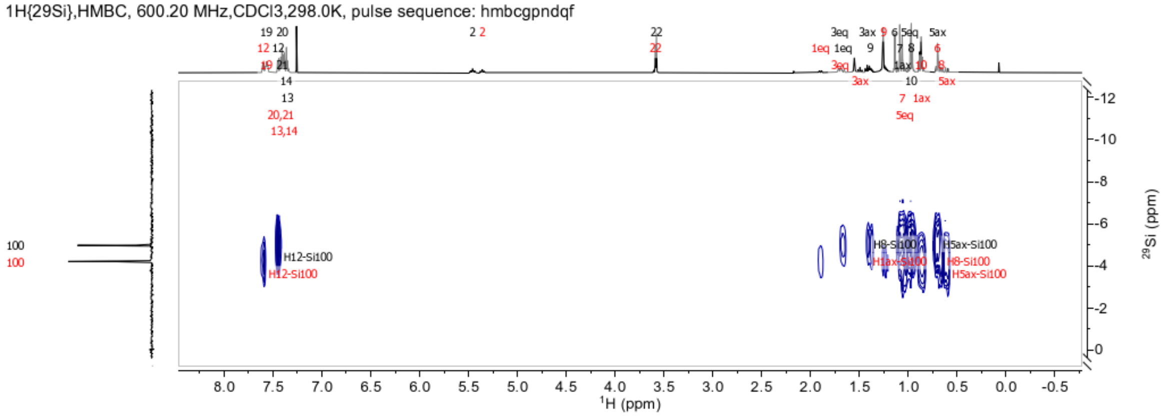

^{1}H NOESY NMR spectrum of 11

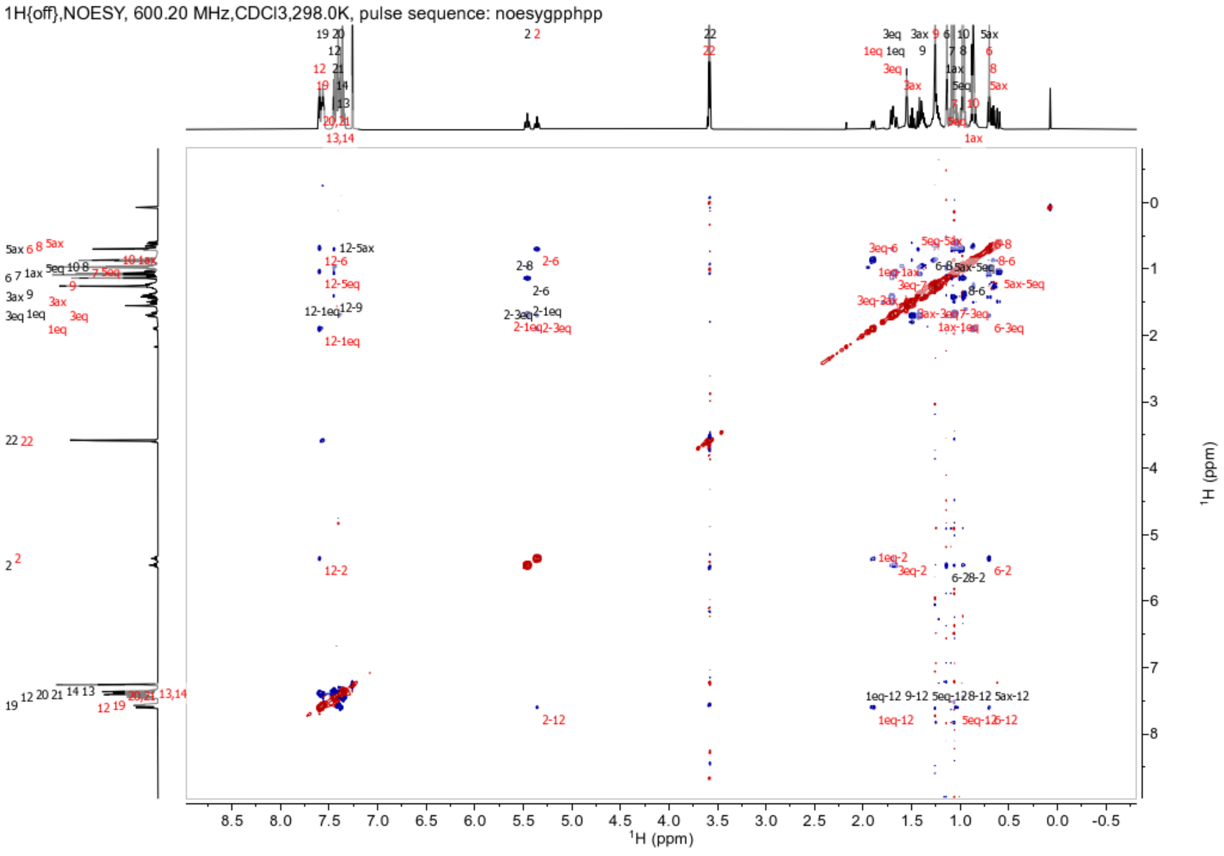

^{19}F NMR spectrum of 11

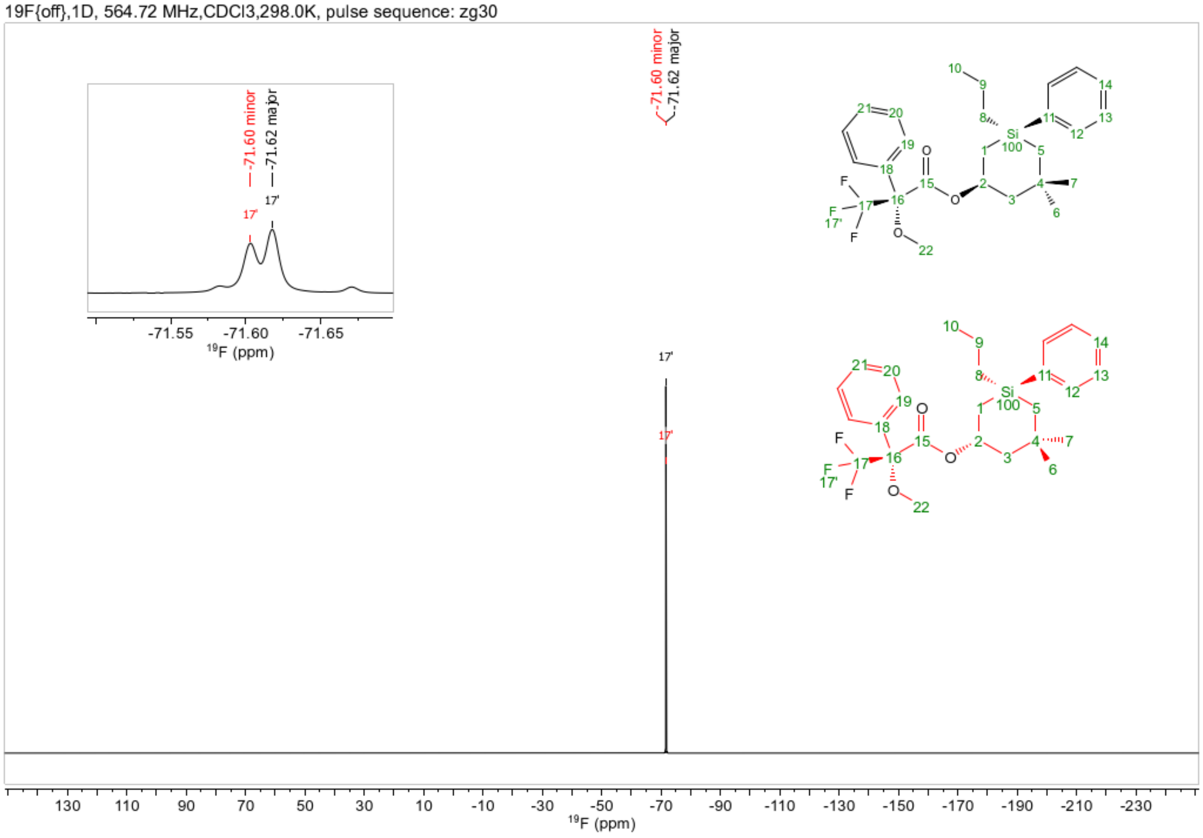

1D TOCSY NMR spectrum of 11

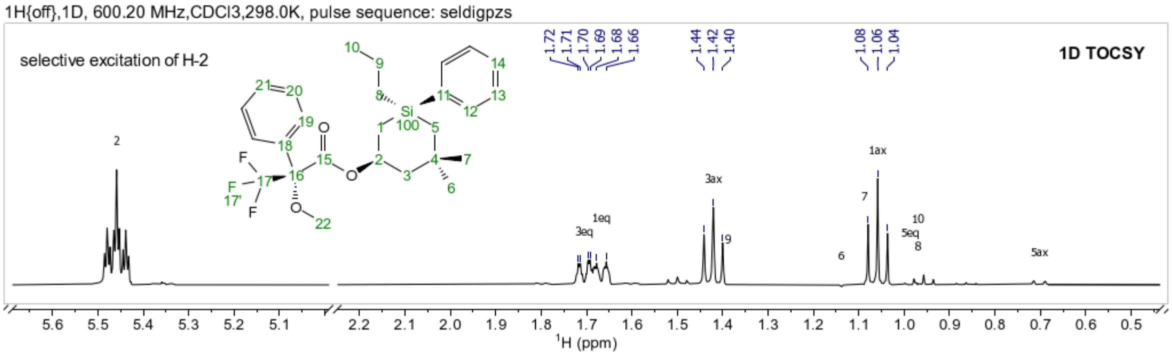

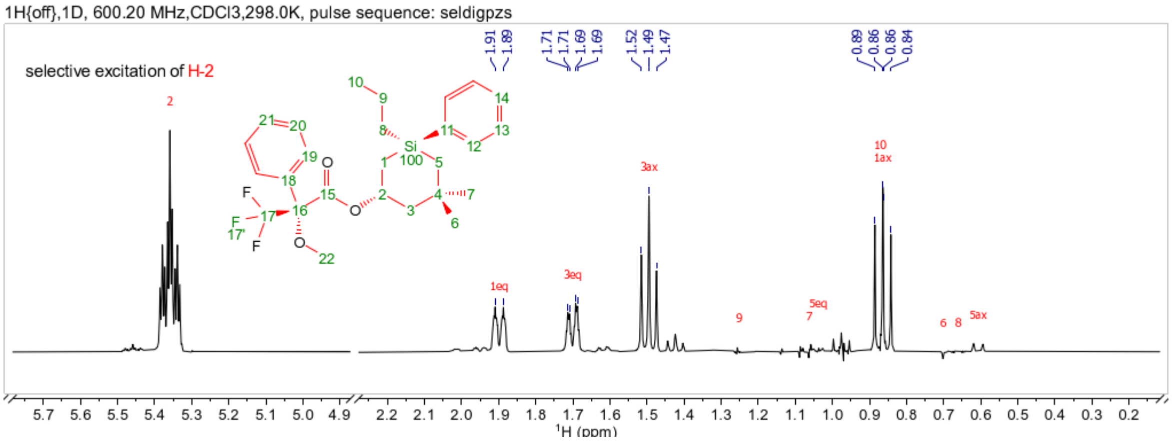

1D NOESY NMR spectrum of 11

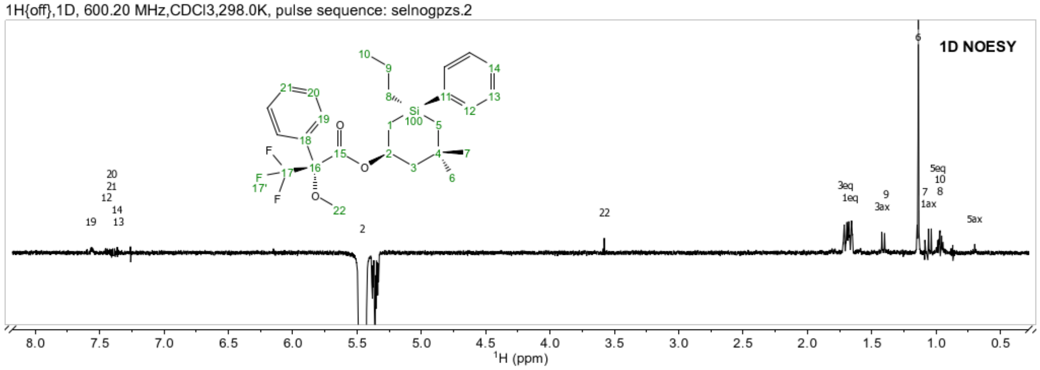

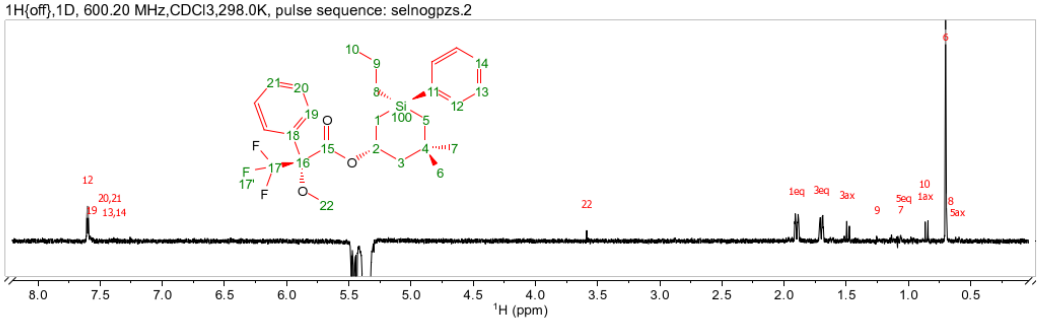

Δδ (=δ_S–δ_R) data for Mosher esters 11 and 11’

Major Diasteromer					Minor Diasteromer				
HBD-JB-226-01 *R*-Mosher Ester		HBD-JB-230-01 *S*-Mosher Ester		$\Delta\delta^{SR}$ (ppm)	HBD-JB-226-01 *R*-Mosher Ester		HBD-JB-230-01 *S*-Mosher Ester		$\Delta\delta^{SR}$ (ppm)
Atom	δ (ppm)	Atom	δ (ppm)		Atom	δ (ppm)	Atom	δ (ppm)	
1 C	19.559	1 C	19.179		1 C	17.698	1 C	18.082	
Hax	1.058	Hax	0.957	-0.10	Hax	0.864	Hax	0.975	0.11
Heq	1.667	Heq	1.604	-0.06	Heq	1.898	Heq	1.95	0.05
2 C	75.251	2 C	75.317		2 C	75.357	2 C	75.317	
H	5.459	H	5.453	-0.01	H	5.359	H	5.352	-0.01
3 C	47.313	3 C	47.593		3 C	47.553	3 C	47.289	
Hax	1.421	Hax	1.499	0.08	Hax	1.495	Hax	1.423	-0.07
Heq	1.705	Heq	1.797	0.09	Heq	1.7	Heq	1.617	-0.08
4 C	33.285	4 C	33.351		4 C	33.502	4 C	33.446	
5 C	24.299	5 C	24.357		5 C	24.364	5 C	24.334	
Hax	0.702	Hax	0.698	0.00	Hax	0.607	Hax	0.608	0.00
Heq	0.987	Heq	0.987	0.00	Heq	1.038	Heq	1.041	0.00
6 C	27.941	6 C	27.969		6 C	28.047	6 C	28.011	
H3	1.138	H3	1.149	0.01	H3	0.7	H3	0.694	-0.01
7 C	37.547	7 C	37.589		7 C	37.51	7 C	37.47	
H3	1.088	H3	1.111	0.02	H3	1.063	H3	1.039	-0.02
8 C	17.368	8 C	17.365		8 C	18.521	8 C	18.575	
H2	0.97	H2	0.96	-0.01	H2	0.66	H2	0.678	0.02
9 C	17.704	9 C	17.7		9 C	17.166	9 C	17.188	
H2	1.388	H2	1.393	0.01	H2	1.251	H2	1.264	0.01
10 C	18.418	10 C	18.42		10 C	18.186	10 C	18.206	
H3	0.969	H3	0.969	0.00	H3	0.87	H3	0.883	0.01
11 C	137.085	11 C	137.078		11 C	136.943	11 C	136.897	
12 C	133.733	12 C	133.724		12 C	133.845	12 C	133.845	
H	7.446	H	7.428	-0.02	H	7.6	H	7.6	0.00
13 C	128.077	13 C	128.057		13 C	128.23	13 C	128.231	
H	7.354	H	7.341	-0.01	H	7.385	H	7.341	-0.04
14 C	129.437	14 C	129.414		14 C	129.354	14 C	129.365	
H	7.364	H	7.354	-0.01	H	7.385	H	7.354	-0.03
15 C	166.085	15 C	166.053		15 C	166.058	15 C	166.062	
16 C	84.558	16 C	84.613		16 C	84.63	16 C	84.555	
17 C	123.538	17 C	123.53		17 C	123.53	17 C	123.57	
17' F	-71.618	17' F	-71.67		17' F	-71.603	17' F	-71.582	
18 C	132.698	18 C	132.65		18 C	132.698	18 C	132.746	
19 C	127.522	19 C	127.515		19 C	127.543	19 C	127.552	
H	7.568	H	7.555	-0.01	H	7.567	H	7.577	0.01
20 C	128.487	20 C	128.487		20 C	128.501	20 C	128.487	
H	7.406	H	7.406	0.00	H	7.42	H	7.406	-0.01
21 C	129.614	21 C	129.613		21 C	129.626	21 C	129.613	
H	7.406	H	7.407	0.00	H	7.42	H	7.407	-0.01
22 C	55.5	22 C	55.525		22 C	55.54	22 C	55.525	
H3	3.578	H3	3.577	0.00	H3	3.588	H3	3.601	0.01
100 Si	-4.97	100 Si	-4.948	0.02	100 Si	-4.152	100 Si	-4.152	0.00

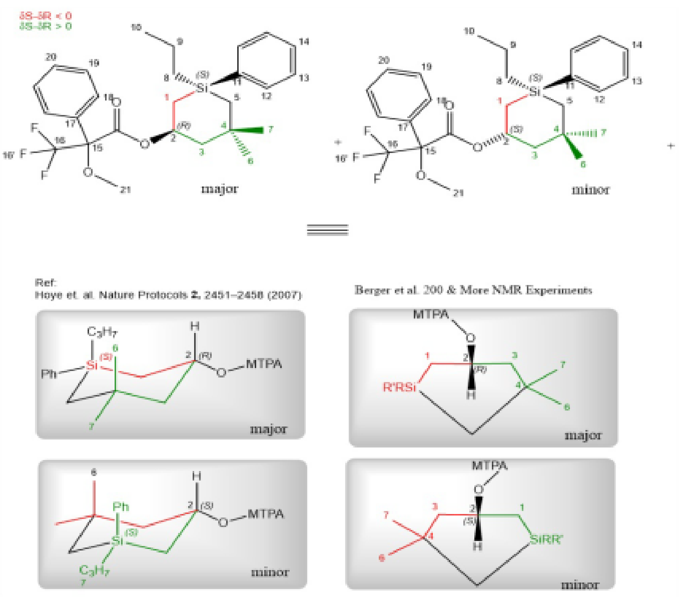

NMR data of 11’

User Report
HBD-JB-230-01
(*S*-Mosher ester)

The sample contains the following 2 diastereomers:

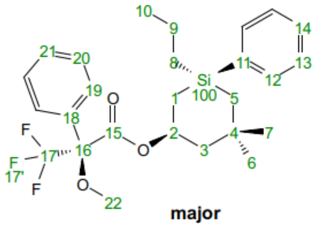

major

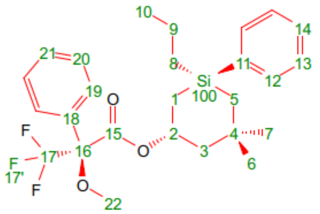

minor

Remarks:
These are the *S*-Mosher ester derivatives.
Based on the observed couplings (2 large ^3J~12.5 Hz (*trans*) and 2 smaller ones ^3J~3.8 Hz (cis)) H-2 is mostly in the axial position in the ring and the OR group in the equatorial position. This enabled the simple differentiation of the 2 diasteromers based on a NOE from H-2 and H-6 to H-8 (major) and a NOE from H-2 and H-6 to H-12 (minor).

An overview of all chemical shift assignments assignments can be found on page 3-4.

Mosher ester analysis (see next page) lead to the conclusion that the stereocentre is (*R*) configured at C-2 for the major and (*S*) for the minor diastereomer. Based on the NOE analysis for the determination of the diastereomers, this suggest that the stereoconfiguration is (S) at Si-100 in both cases.

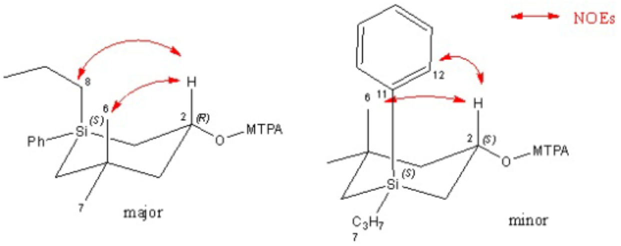

P-ID: ML00xxx

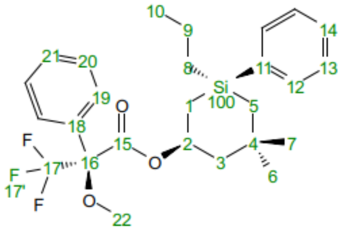

Atom	J	δ (ppm)	HSQC	COSY	HMBC	NOESY
1 C		19.179	1ax, 1eq		3ax, 3eq, 5eq	
Hax	12.90(1eq), 12.80(2)	0.957	1	1eq, 2	2, 3, 5, 100	1eq, 12
Heq	12.90(1ax)	1.604	1	1ax, 2, 3eq	2, 3, 5	1ax, 2, 12
2 C		75.317	2		1ax, 1eq, 3ax	
H	12.80(1ax), 11.90(3ax), 3.70(?), 3.70(?)	5.453	2	1ax, 1eq, 3ax, 3eq	3, 15	1eq, 3eq, 6, 8
3 C		47.593	3ax, 3eq		1ax, 1eq, 2, 5ax, 5eq, 6, 7	
Hax	12.80(3eq), 11.90(2)	1.499	3	2, 3eq	1, 2, 4, 5, 6, 7	3eq
Heq	12.80(3ax)	1.797	3	1eq, 2, 3ax, 5eq	1, 4, 5	2, 3ax, 6, 7
4 C		33.351			3ax, 3eq, 5ax, 5eq, 6, 7	
5 C		24.357	5ax, 5eq		1ax, 1eq, 3ax, 3eq, 6, 7	
Hax		0.698	5	5eq, 6	3, 4, 6, 7, 8, 100	5eq, 12
Heq		0.987	5	3eq, 5ax	1, 3, 4, 6, 7, 8, 11	5ax, 12
6 C		27.969	6		3ax, 5ax, 5eq, 7	
H3		1.149	6	5ax	3, 4, 5, 7	2, 3eq, 8
7 C		37.589	7		3ax, 5ax, 5eq, 6	
H3		1.111	7		3, 4, 5, 6, 100	3eq
8 C		17.364	8		5ax, 5eq, 9, 10	
H2		0.960	8	9	9, 10, 11, 100	2, 6, 12
9 C		17.699	9		8, 10	
H2	7.20(10)	1.393	9	8, 10	8, 10	12
10 C		18.420	10		8, 9	
H3	7.20(9)	0.969	10	9	8, 9	

Atom	J	δ (ppm)	HSQC	COSY	HMBC	NOESY
11 C		137.078			5eq, 8, 12, 13	
12 C		133.724	12		12, 14	
H		7.428	12	13	11, 12, 14, 100	1ax, 1eq, 5ax, 5eq, 8, 9
13 C		128.057	13		13	
H		7.341	13	12, 14	11, 13	
14 C		129.414	14		12	
H		7.354	14	13	12	
15 C		166.053			2	
16 C	27.40(17')	84.613			19, 22	
17 C	288.40(17')	123.530				
17' F	27.40(16), 1.40(22), 1.10(22H), 1.20(19H), 288.40(17)	-71.670				
18 C		132.650			20	
19 C		127.515	19		21	
H	1.20(17'F)	7.555	19	20	16, 21	
20 C		128.487	20		20	
H		7.406	20	19, 21	18, 20	
21 C		129.613	21		19	
H		7.407	21	20	19	
22 C	1.40(17')	55.525	22			
H3	1.10(17'F)	3.577	22		16	
100 Si		-4.948			1ax, 5ax, 7, 8, 12	

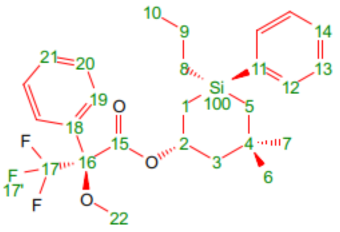

Atom	J	δ (ppm)	HSQC	COSY	HMBC	NOESY
1 C		18.082	1ax, 1eq		3ax, 5eq	
Hax	13.40(1eq), 12.50(2), 12.50(3ax)	0.975	1	1eq, 2	2, 3, 100	1eq
Heq	13.40(1ax)	1.950	1	1ax, 2, 3eq	2, 3	1ax, 2, 12
2 C		75.317	2		1ax, 1eq, 3ax, 3eq, 7	
H	12.50(1ax)	5.352	2	1ax, 1eq, 3ax, 3eq	3, 15	1eq, 3eq, 6, 12
3 C		47.289	3ax, 3eq		1ax, 1eq, 2, 5ax, 5eq, 6, 7	
Hax	12.50(1ax)	1.423	3	2, 3eq	1, 2, 4, 5, 6, 7	3eq
Heq		1.617	3	1eq, 2, 3ax, 5eq	2, 4, 5	2, 3ax, 6, 7
4 C		33.446			3ax, 3eq, 5ax, 5eq, 6, 7	
5 C		24.334	5ax, 5eq		3ax, 3eq, 6, 7	
Hax	14.90(5eq)	0.608	5	5eq, 6	3, 4, 6, 7, 100	5eq
Heq	14.90(5ax)	1.041	5	3eq, 5ax	1, 3, 4, 6, 7, 11	5ax, 12
6 C		28.011	6		3ax, 5ax, 5eq, 7	
H3		0.694	6	5ax	3, 4, 5, 7	2, 3eq, 8, 12
7 C		37.470	7		3ax, 5ax, 5eq, 6	
H3		1.039	7		2, 3, 4, 5, 6, 100	3eq
8 C		18.575	8		10	
H2		0.678	8	9	9, 10, 11, 100	6
9 C		17.188	9		8, 10	
H2		1.264	9	8, 10	10	
10 C		18.206	10		8, 9	
H3		0.883	10	9	8, 9	

Atom	J	δ (ppm)	HSQC	COSY	HMBC	NOESY
11 C		136.897			5eq, 8, 12, 13	
12 C		133.845	12		12, 14	
H		7.600	12	13	11, 12, 14, 100	1eq, 2, 5eq, 6
13 C		128.231	13		13	
H		7.341	13	12, 14	11, 13	
14 C		129.365	14		12	
H		7.354	14	13	12	
15 C		166.062			2	
16 C		84.555			19, 22	
17 C	288.40(17')	123.570				
17' F	288.40(17), 1.10(22), 1.20(19H)	-71.582				
18 C		132.746			20	
19 C		127.552	19		21	
H	1.20(17'F)	7.577	19	20	16, 21	
20 C		128.487	20		20	
H		7.406	20	19, 21	18, 20	
21 C		129.613	21		19	
H		7.407	21	20	19	
22 C	1.10(17')	55.525	22			
H3		3.601	22		16	
100 Si		-4.152			1ax, 5ax, 7, 8, 12	

^{1}H NMR spectrum of 11'

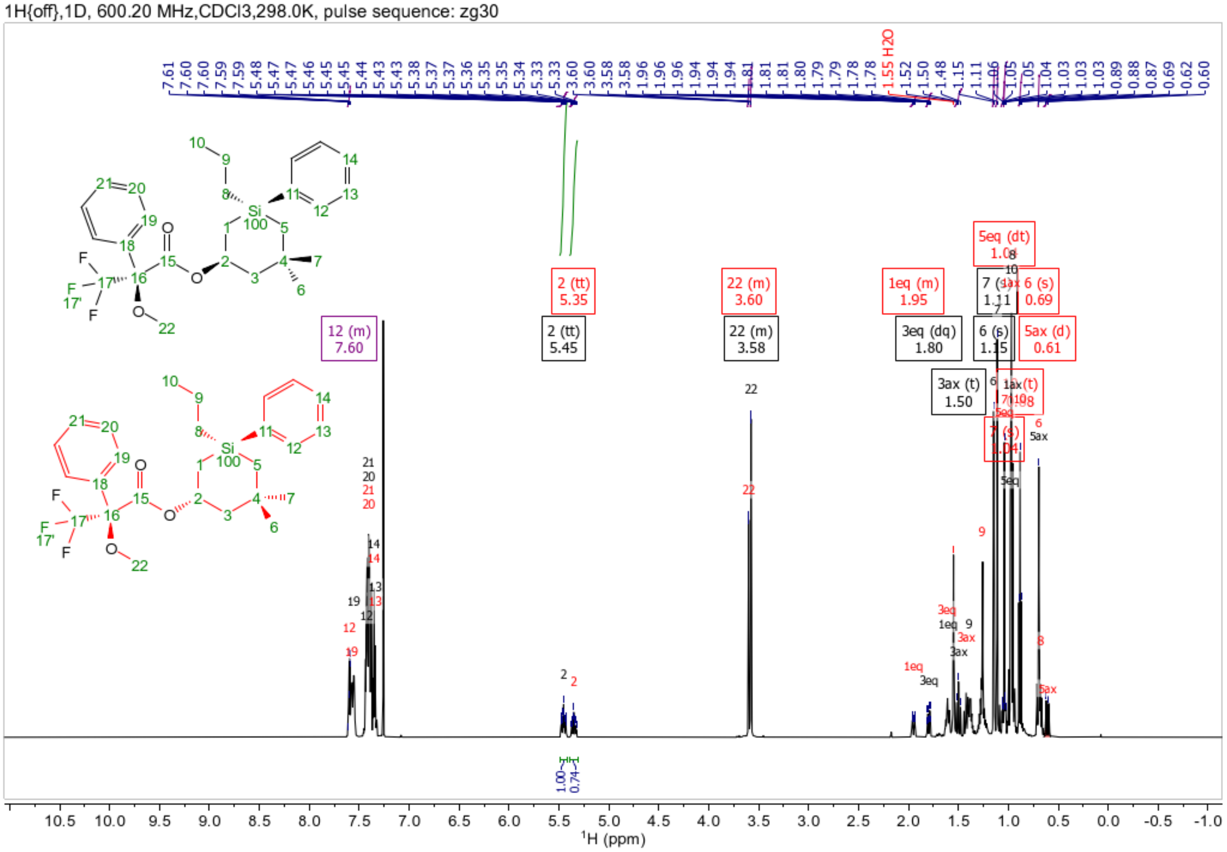

^{13}C NMR spectrum of 11'

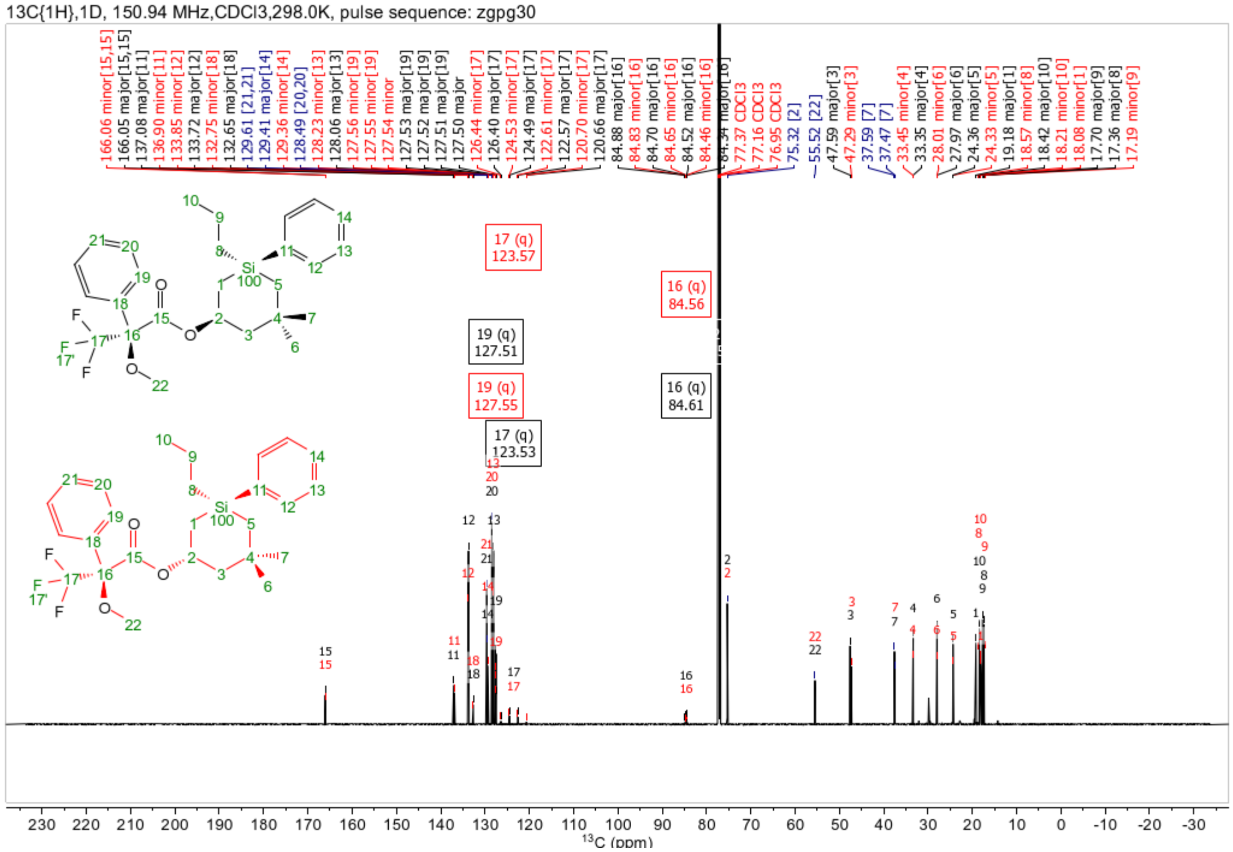

^{13}C NMR spectrum of 11'

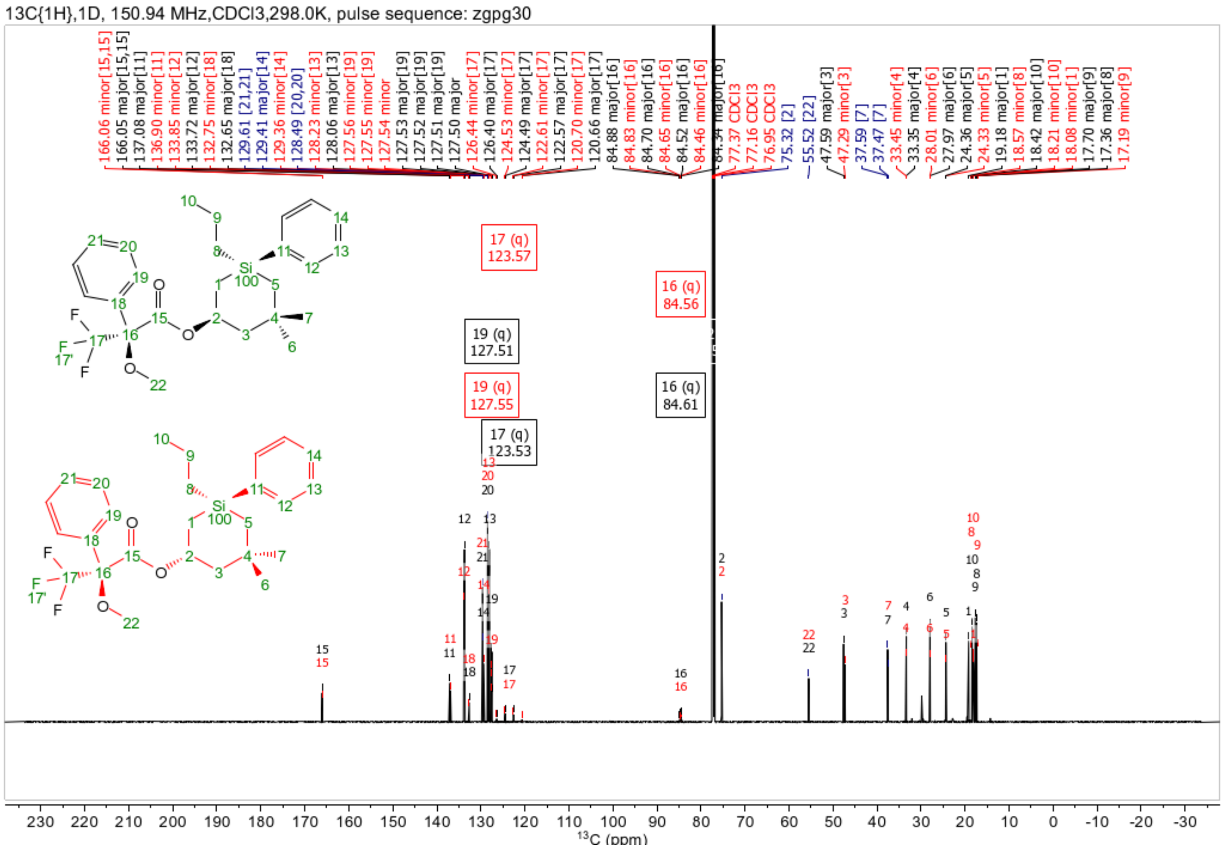

^{1}H-^{13}C HSQC spectrum of 11'

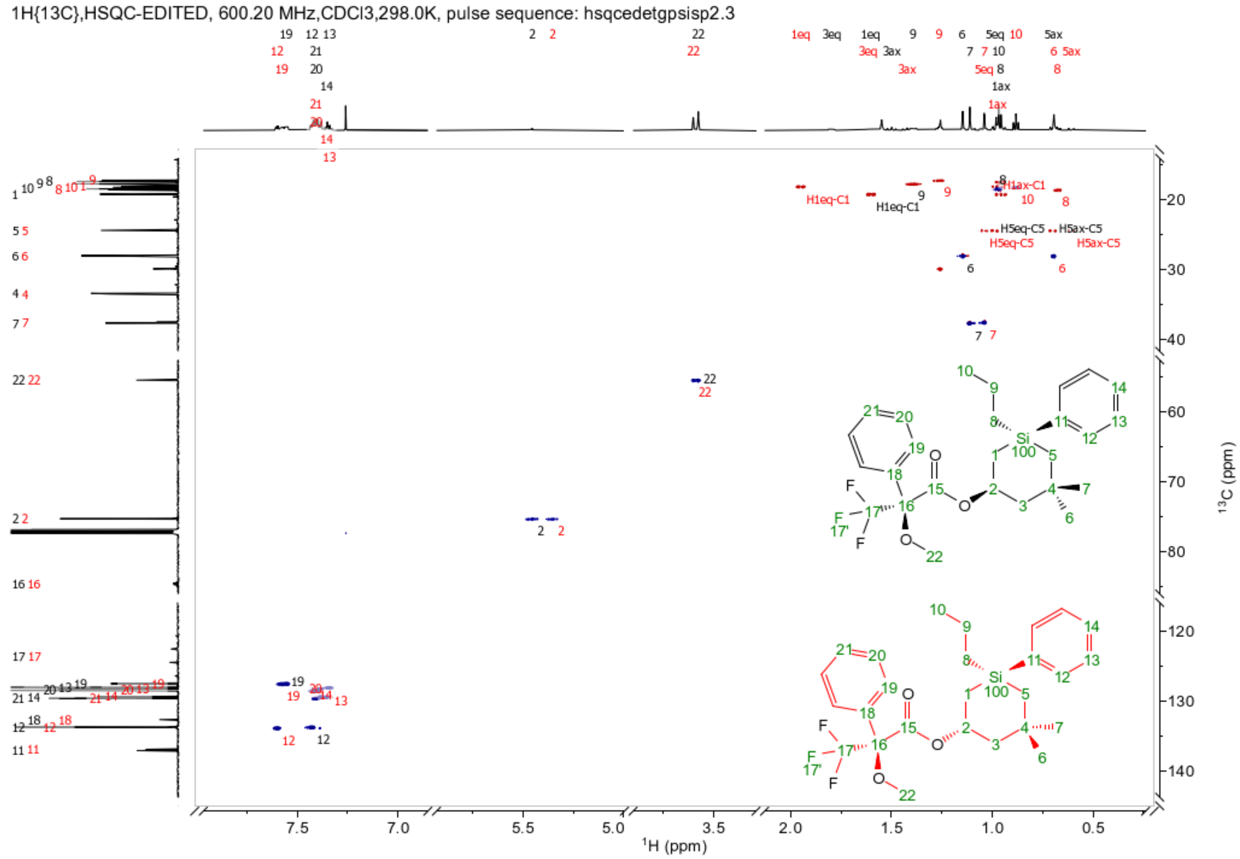

^{1}H-^{13}C HMBC spectrum of 11'

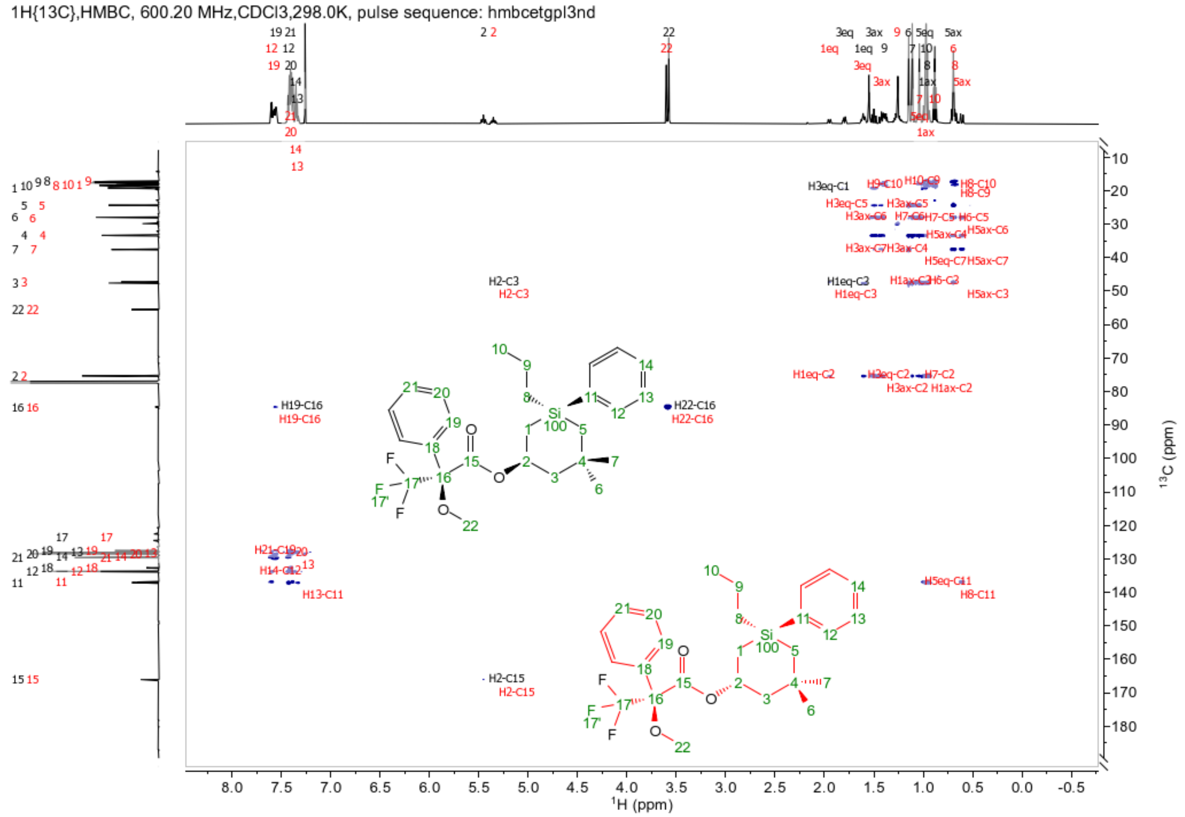

^{1}H COSY spectrum of 11'

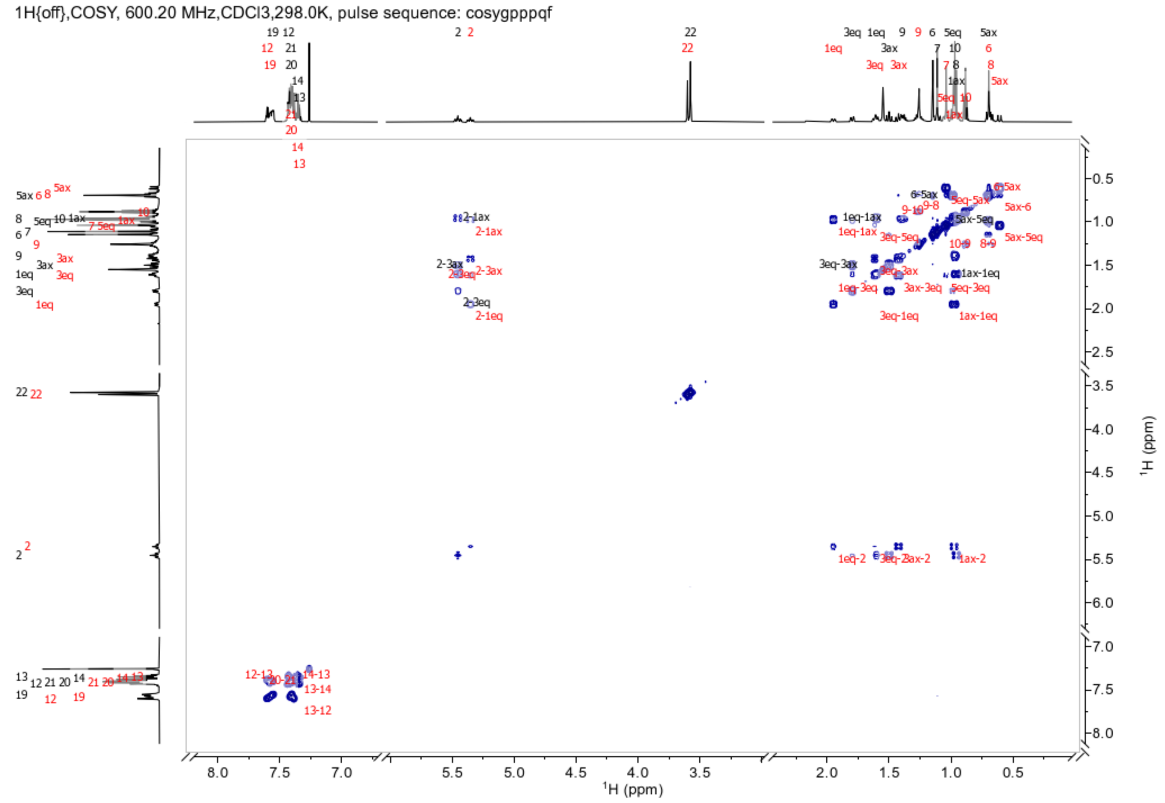

^{1}H NOESY spectrum of 11'

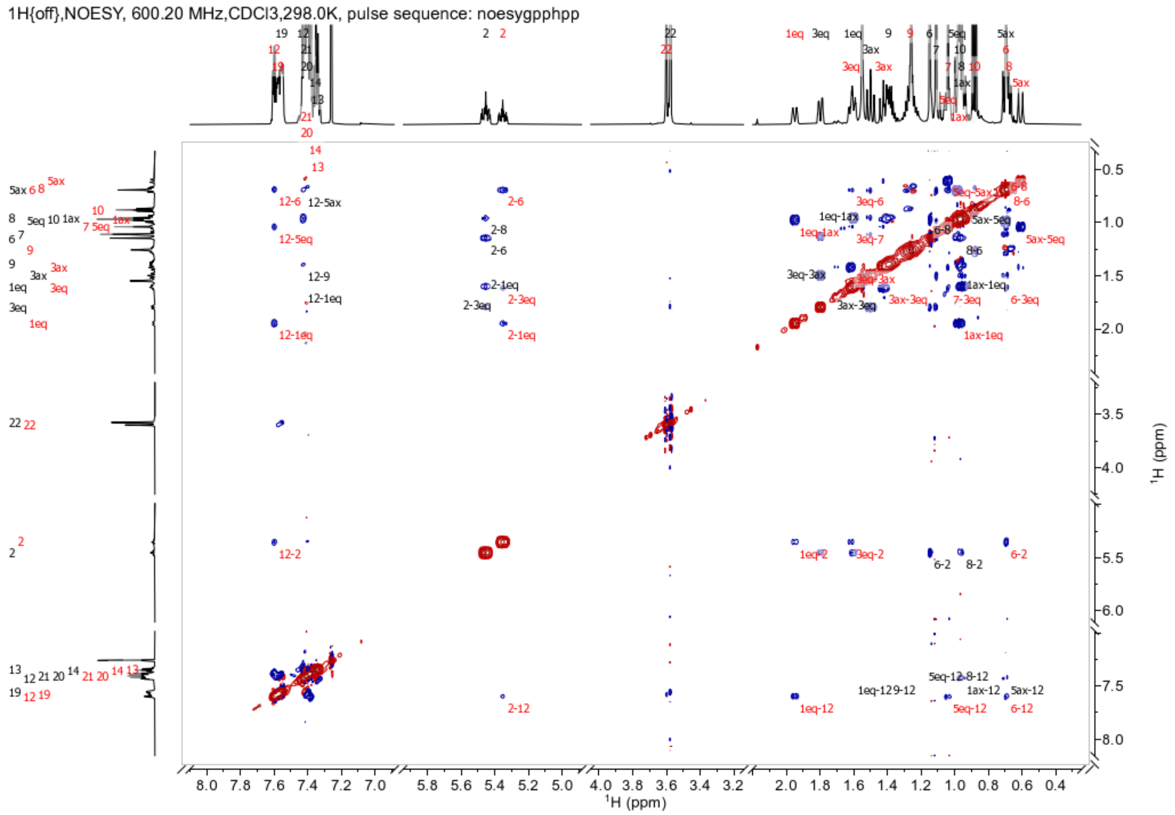

^{29}Si NMR and ^{1}H-^{29}Si HMBC NMR spectra of 11'

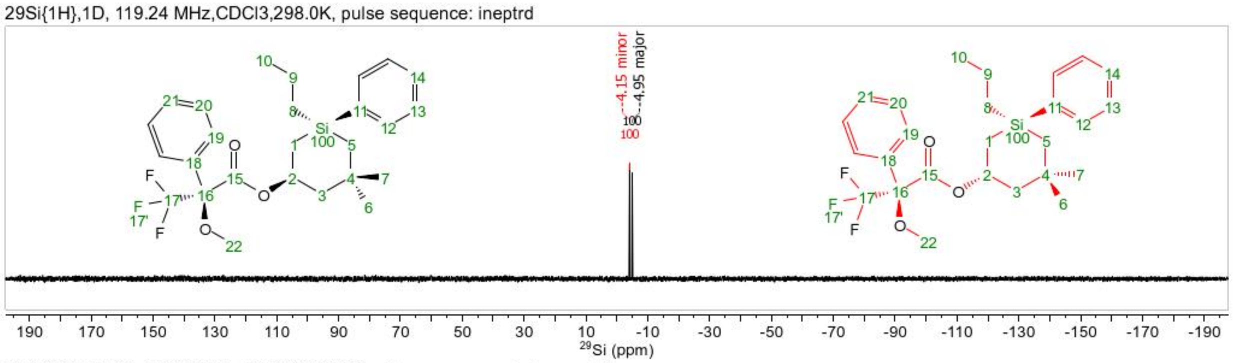

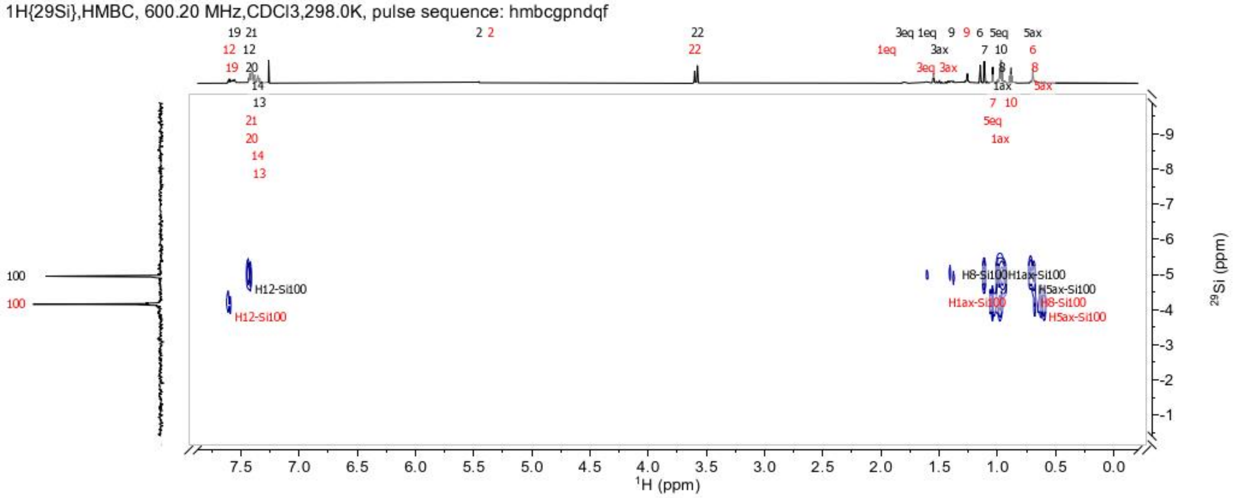

^{19}F NMR spectrum of 11'

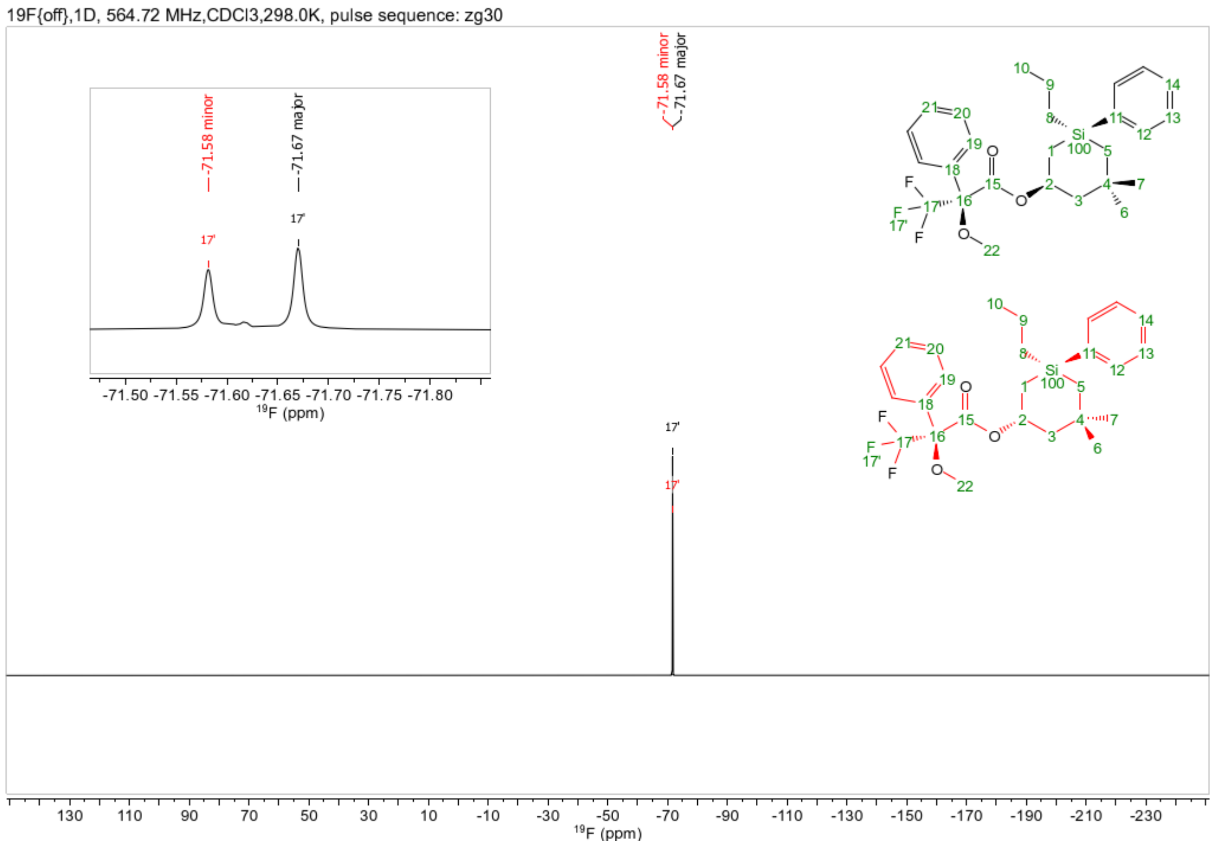

1D TOCSY NMR spectrum of 11'

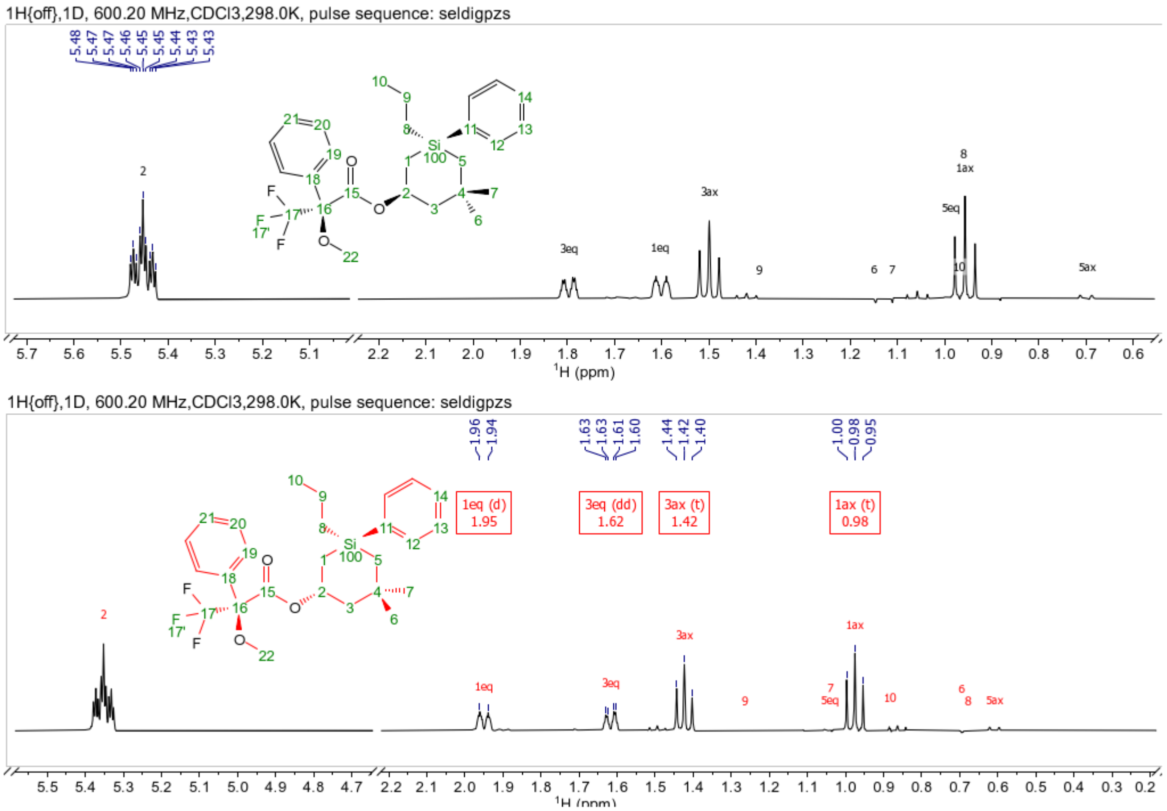

1D NOESY NMR spectrum of 11'

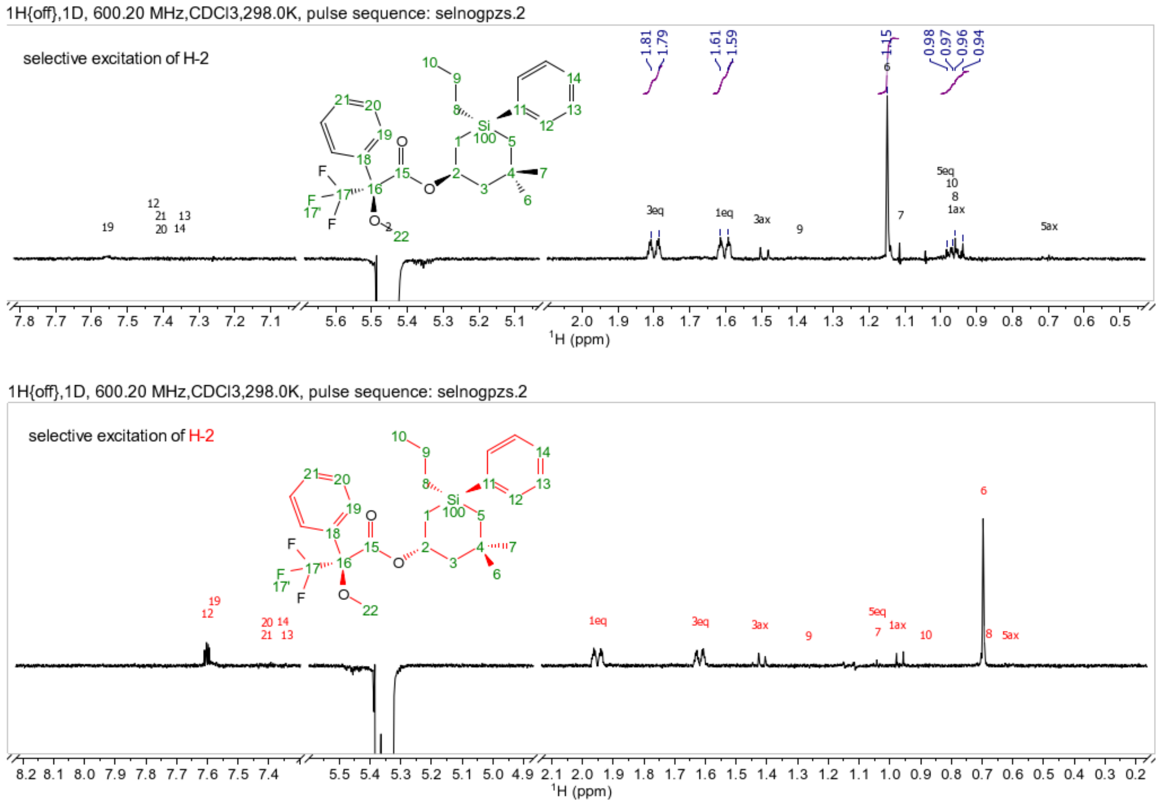

9.2. Electronic circular dichroism (ECD) analysis

The ECD-spectra of **2a** (1 mg/mL) was recorded in *n*-hexane (HPLC grade) at 20 °C and compared with the corresponding TD-DFT calculated ECD spectra of the possible conformers. After a correction of the σ-value of 0.33 eV, the CD characteristics of the calculated spectrum (blue curve) was in good agreement with the experimental spectrum (red curve), thus allowing the assignment of the absolute configuration of **2a**, and the absolute configuration of **2a** could be assigned to be (*S*) using catalyst **6d**.

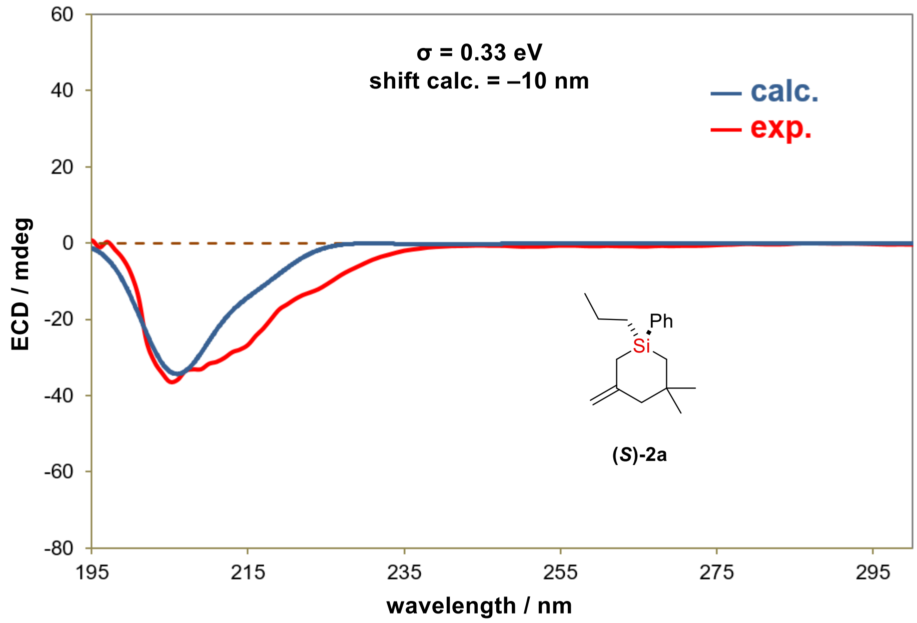

Supplementary Figure 9. Comparison between the experimental ECD-spectrum (red curve) and the calculated spectrum (blue curve).

9.3. ECD spectrum calculation

Density Functional Theory (DFT) calculations were performed on the Max-Planck-Institut für Kohlenforschung computer cluster using the ORCA program package (Version 5.0-Stable).[6] Structural optimizations and frequency calculations to identify all of the stationary points as minima (zero imaginary frequencies) and to obtain thermal and entropic correction were performed with the PBE functional[7,8] with D3 dispersion correction[9] and Becke-Johnson damping (BJ)[10] along with RI approximation, utilizing the def2/J auxiliary basis set[11] and the def2-SVP basis set[12] on all atoms. The libint2 library was used for the computation of 2-el integrals.[13] Tight SCF convergence and geometry optimization criterions were chosen.

Using the Gaussian 09 program, the CD spectrum was computed by time-dependent density functional theory (TD-DFT, NSTATES = 5) at m062x/def2tzvp level, solvent effects of *n*-hexane were taken into account on the basis of the conductor-like polarized continuum model using the integral equation formalism variant (IEFPCM).[14] The CD spectrum was created using Multiwfn[15] with a half-width at 1/6-height. Staring from the initial guess structures obtained in the first step, molecular dynamics (MD) simulations with xTB 6.3[16] employing GFN2-xTB method and the following data processing with Molclus[17] were performed to sample all the possible conformers of the compound.

10. Limitations

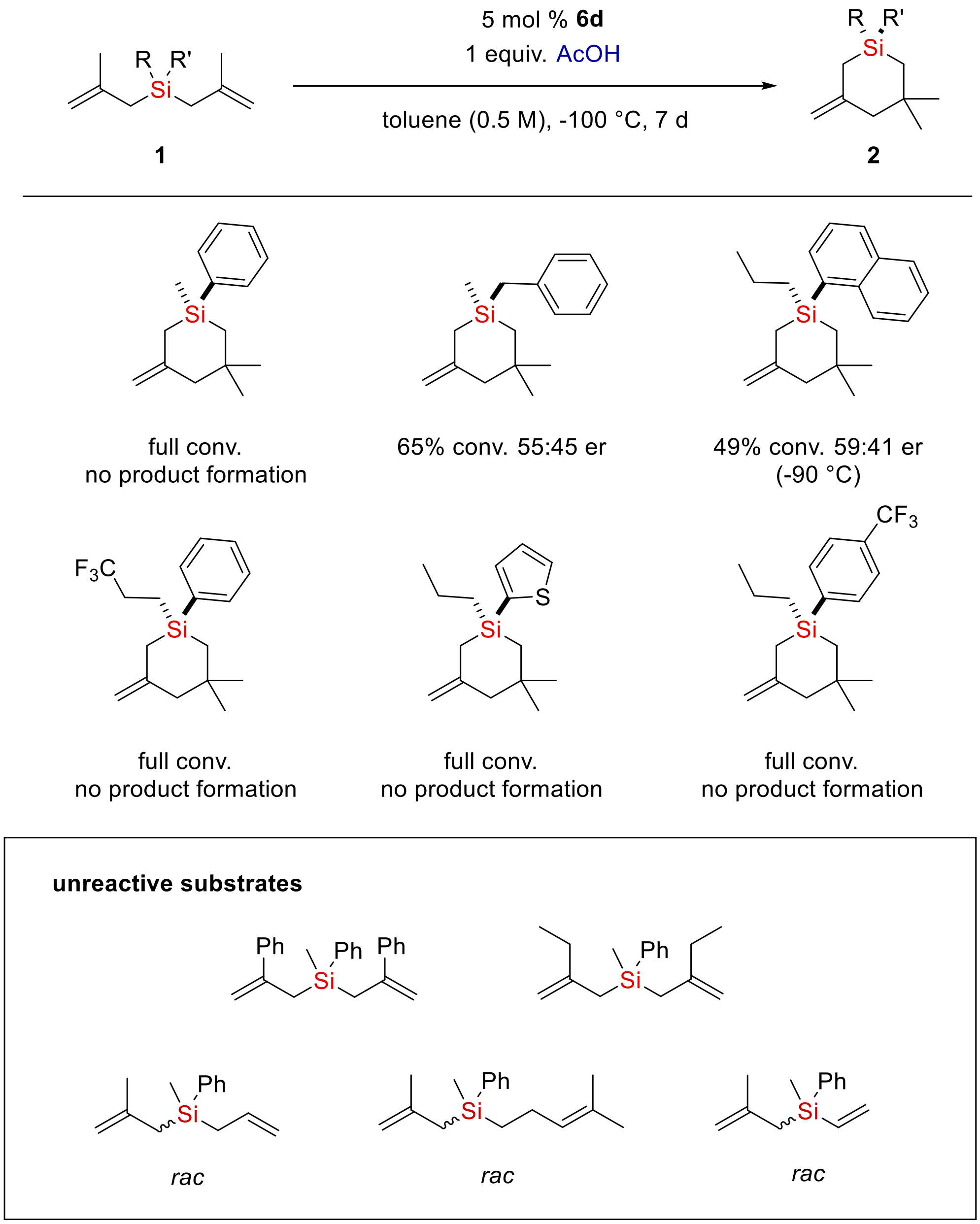

Supplementary Figure 10. Unsuccessful substrates.

11. DFT calculations

11.1. Method

Possible TS conformations were explored by the artificial force induced reaction (AFIR) method[18] implemented in the global route reaction mapping (GRRM) program.[19] An extensive conformational search has been performed on possible catalyst substrate orientations at GFN2-xTB level of theory[20] implemented in Orca 4.2.1,[21] using SC-AFIR with constraint. Molecular geometries were optimized using B3LYP-D3/6-31G(d)[22] implemented in Gaussian 16 program,[23] followed by further optimization at r^{2}SCAN-3c[24]

implemented in Orca 5.0.3 program.[25] In the case of transition state optimization, they were combined with GRRM program. Thermal free energy corrections have been performed at the same level of theory using Orca 5.0.3 program, and the temperature was set at 173.15K. Transition state structures were verified by the presence of a single imaginary vibrational frequency and the corresponding intrinsic reaction coordinates. Solvation effect has been accounted by using CPCM (toluene) solvation model[26] as implemented in Orca 5.0.3 program. All single point energy is calculated at CPCM(toluene)-wB97M-V/(ma)-def2-TZVPP level of theory.[27] The (ma)-def2-TZVPP basis set refers to ma-def2-TZVPP[28] on P, N, S, and O in the catalytic active site, and def2-TZVPP[29] on all others for a better evaluation of anionic species. RI approximation was used with AutoAux option implemented in Orca 5.0.3. The molecular structures were visualized with the Chimera program.[30] Conversion of enantiomeric ratio and DDG was performed based on the Boltzmann distribution as follows: DDG=RTln(pdt(*R*)/pdt(*S*)).[31] Visualization of NCIplot is performed using VMD program.[32]

11.2. Results and discussion

While overall energies are very similar between the major enantiomer and the minor enantiomer, the largest difference comes from **TS4** and **TS4'**. The major difference could be steric repulsion induced by the substrate, as indicated below. In the case of **TS4**, both *gem*-dimethyl group and the smaller substituent of silicon, in this case *n*-propyl group, seem to suffer from steric repulsion with the 3,3'-substituents of the catalyst, resulting in distorting the anion. On the other hand, for **TS4'**, the *n*-propyl group can be folded to minimize the steric repulsion. The superimposed image of the counter anion illustrates the difference of the distortion induced to the 3,3'-substituents.

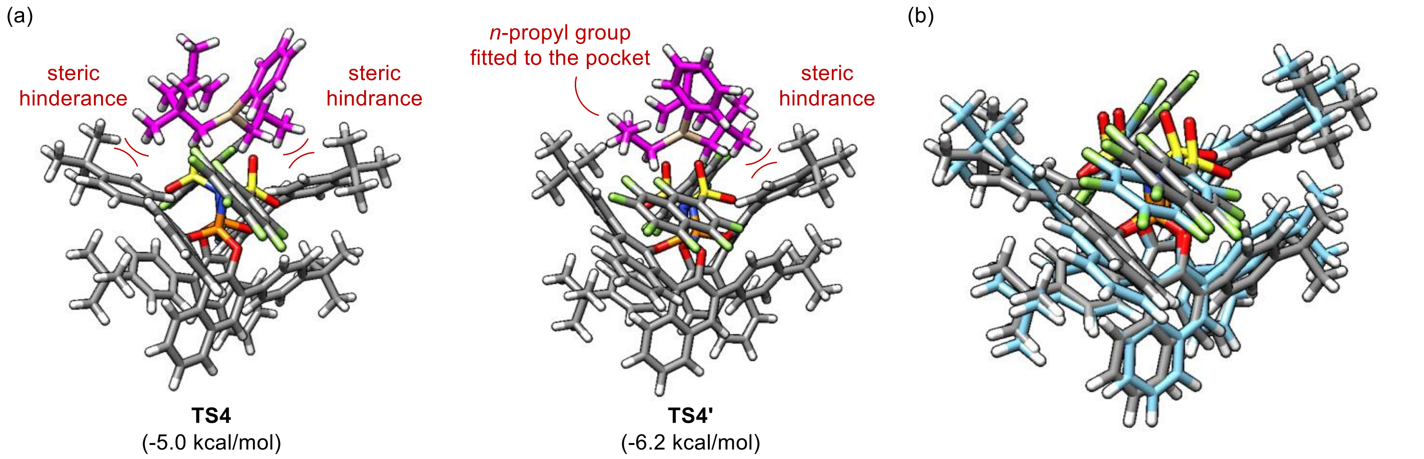

Supplemenatry Figure 11. a Visualization of **TS4** and **TS4'** for comparison. Substrate is depicted in magenta. **b** Visualization of superimposed structures of anions of **TS4** (gray) and **TS4'** (light blue).

The calculated yields and enantioselectivities are provided based on the premise that the reaction yields only the desired products and the adduct. The procedure is as follows:

1. In **TS1** and **TS1'**, distribution between **I** and **I'** is calculated; 0.22 kcal/mol corresponds to 65.6:34.4.

2. In **TS3** and **TS4**, the distribution between **[2a+6d]** and **8** is calculated; 0.62 kcal/mol corresponds to 85.7:14.3.

3. In **TS3'** and **TS4'**, the distribution between **[2a'+6d]** and **8'** is calculated; -0.83 kcal/mol corresponds to 8.1:91.9.

4. Calculate the ratio between enantiomers and the sum of the adduct: **[2a+6d]: [2a'+6d]:8+8'=** 56.2:2.8:41.0, which corresponds to 59% yield of the products with an enantiomeric ratio of 95.3:4.7.

Supplementary Table 1. Energy table of the optimized structures. Energies are given in Hartree. Computed single point energies (E), Gibbs free energy corrections (Gcorr), Gibbs free energies (G), and imaginary frequencies for transition states are provided.

Structures	E (solv) (in Hartree)	Gcorr (at 173.15 K)	G (solv) (in Hartree)	Imaginary Frequency
**1a**	-952.8759489	0.35888537	-952.5170636	-
**6d**	-7498.231783	1.45172758	-7496.780055	-
**[1a+6d]**	-8451.146374	1.82803224	-8449.318342	-
**TS1**	-8451.128828	1.82494577	-8449.303883	-1089.14418519
**TS1'**	-8451.128248	1.82471947	-8449.303528	-1086.24804528
**I**	- -8451.151331	1.82857293	-8449.322759	-
**I'**	-8451.148664	1.82762856	-8449.321035	-
**TS2**	-8451.141764	1.82844163	-8449.313322	-54.26921178
**TS2'**	-8451.139496	1.82777452	-8449.311721	-56.63791646
**II**	-8451.176813	1.83329962	-8449.343513	-
**II'**	-8451.176335	1.83331707	-8449.343018	-
**TS3**	-8451.156694	1.8293614	-8449.327332	-1089.54125286
**TS3'**	-8451.156641	1.82970974	-8449.326931	-922.17457674
**[2a+6d]**	-8451.17489	1.83305785	-8449.341833	-
**[2a'+6d]**	-8451.173424	1.8334287	-8449.339996	-
**TS4**	-8451.160293	1.83394411	-8449.326349	-10.98260413
**TS4'**	-8451.162716	1.83445466	-8449.328261	-57.67461799
**8**	-8451.184662	1.83215423	-8449.352508	
**8'**	-8451.184075	1.83237255	-8449.351703	-

**2a**	-952.907464	0.3623763	-952.5450877	-

Additionally, non-covalent interactions in **TS4** and **TS4’** are visualized using NCIplot.[33] While there is no significant stabilization induced by the phenyl group of the substrate in **TS4**, in **TS4’**, in contrast, the propyl group is folded and stabilized by both phenyl group of the substrate and one of the substituents of the catalyst. This could explain why the aryl group in the substrate is required to achieve high enantioselectivity in this transformation.

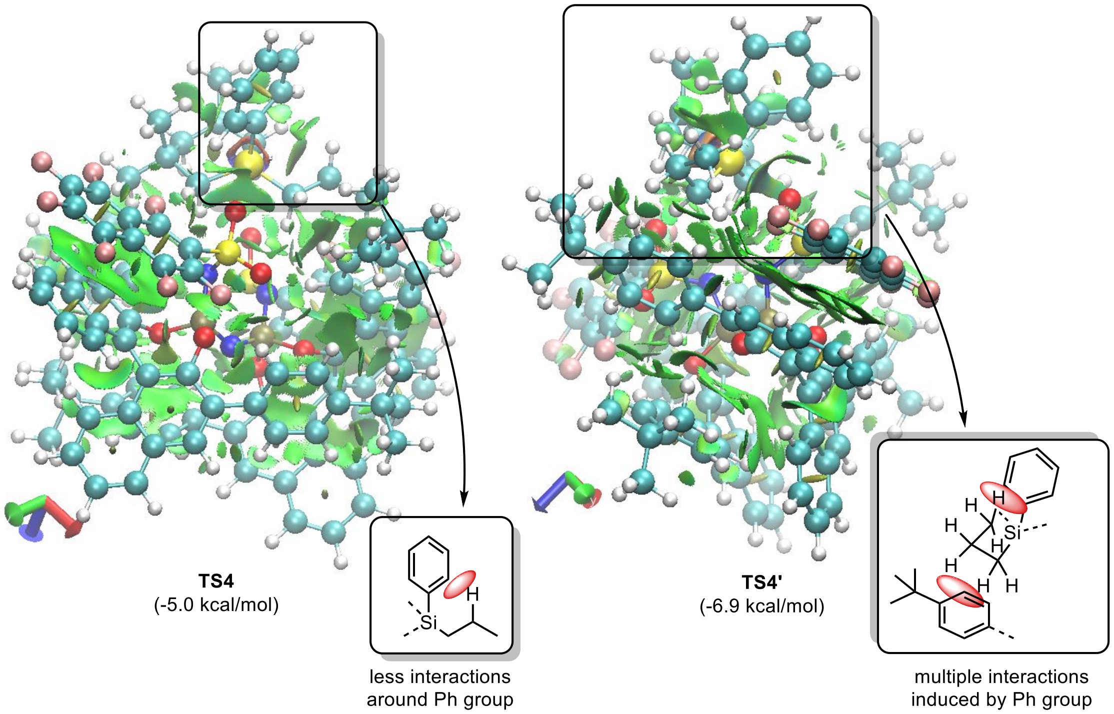

Supplementary Figure 12. Comparison of non-covalent interactions between **TS4** and **TS4’** using NCIplot. The isovalue is 0.4. The electron density was computed at the ωB97XD/def2TZVPP level of theory.

12. Copies of NMR spectra

bis(2-methylallyl)(phenyl)(propyl)silane 1a

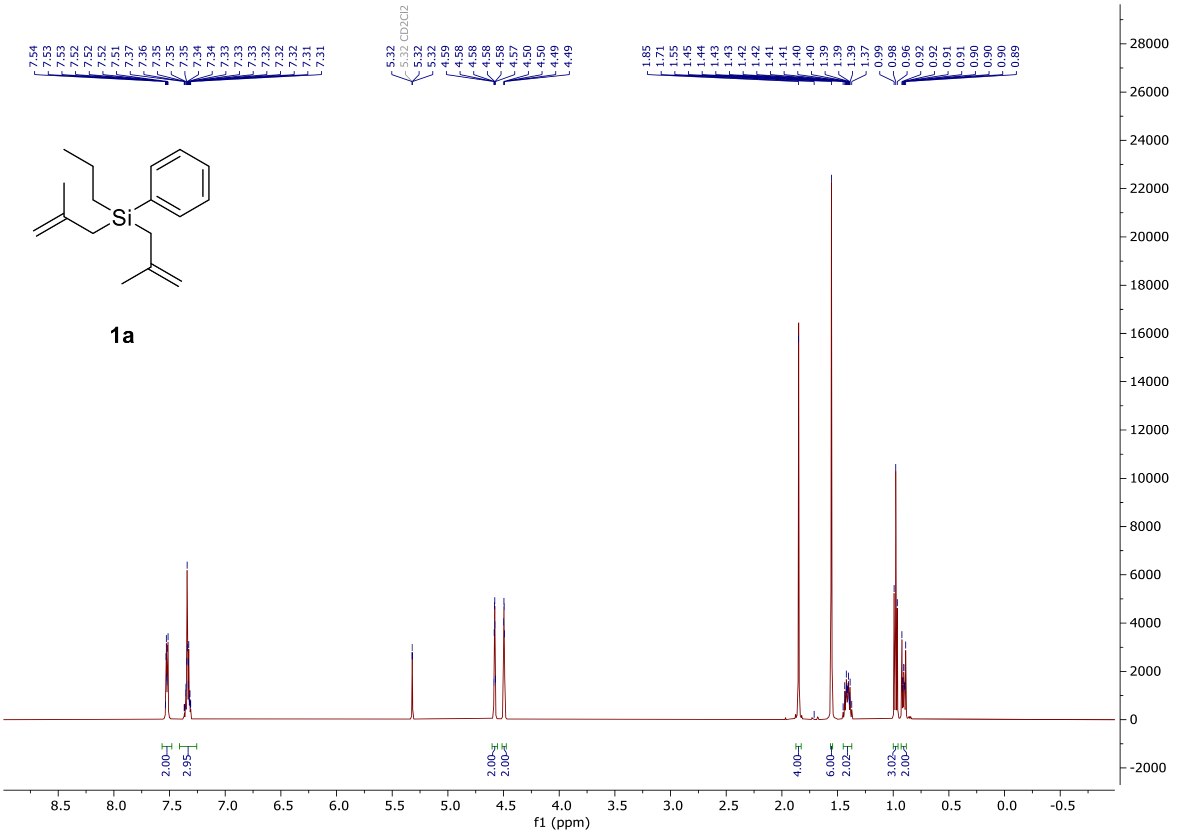

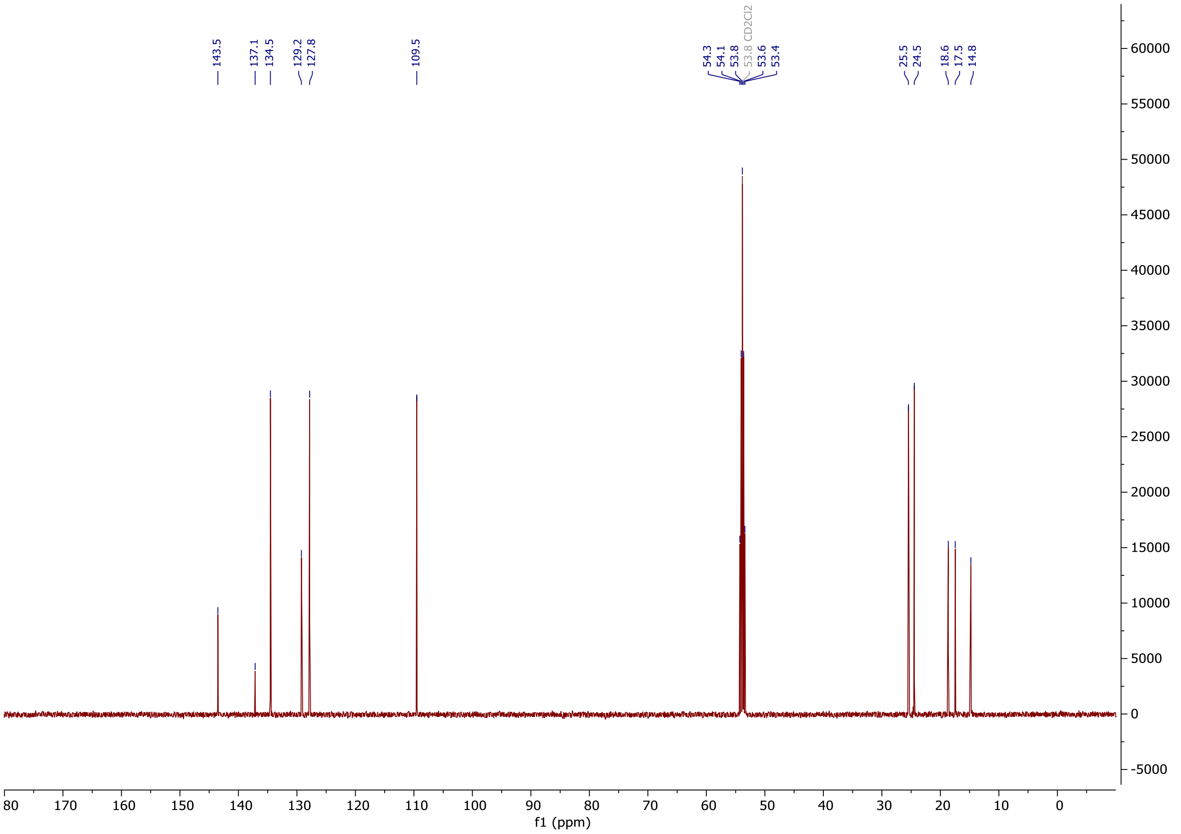

ethylbis(2-methylallyl)(phenyl)silane 1b

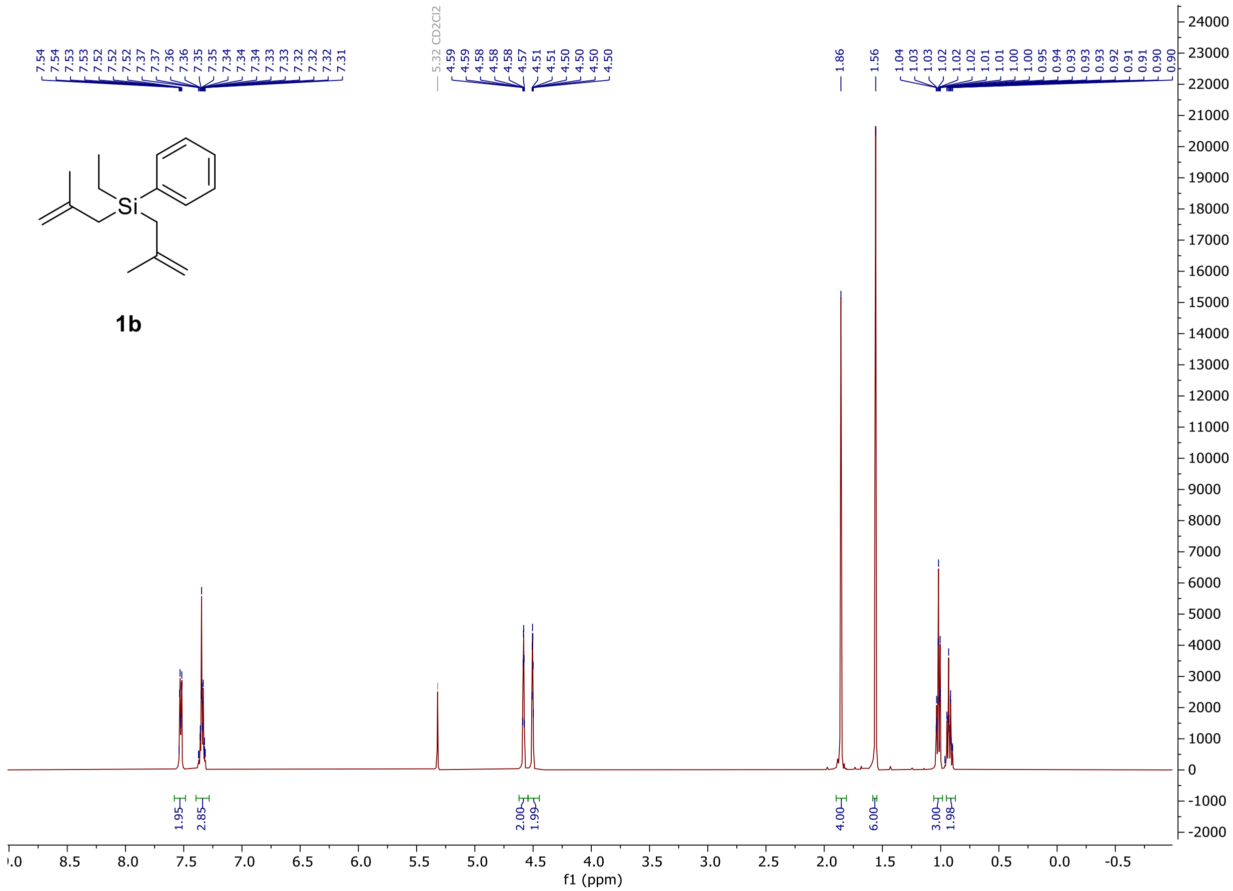

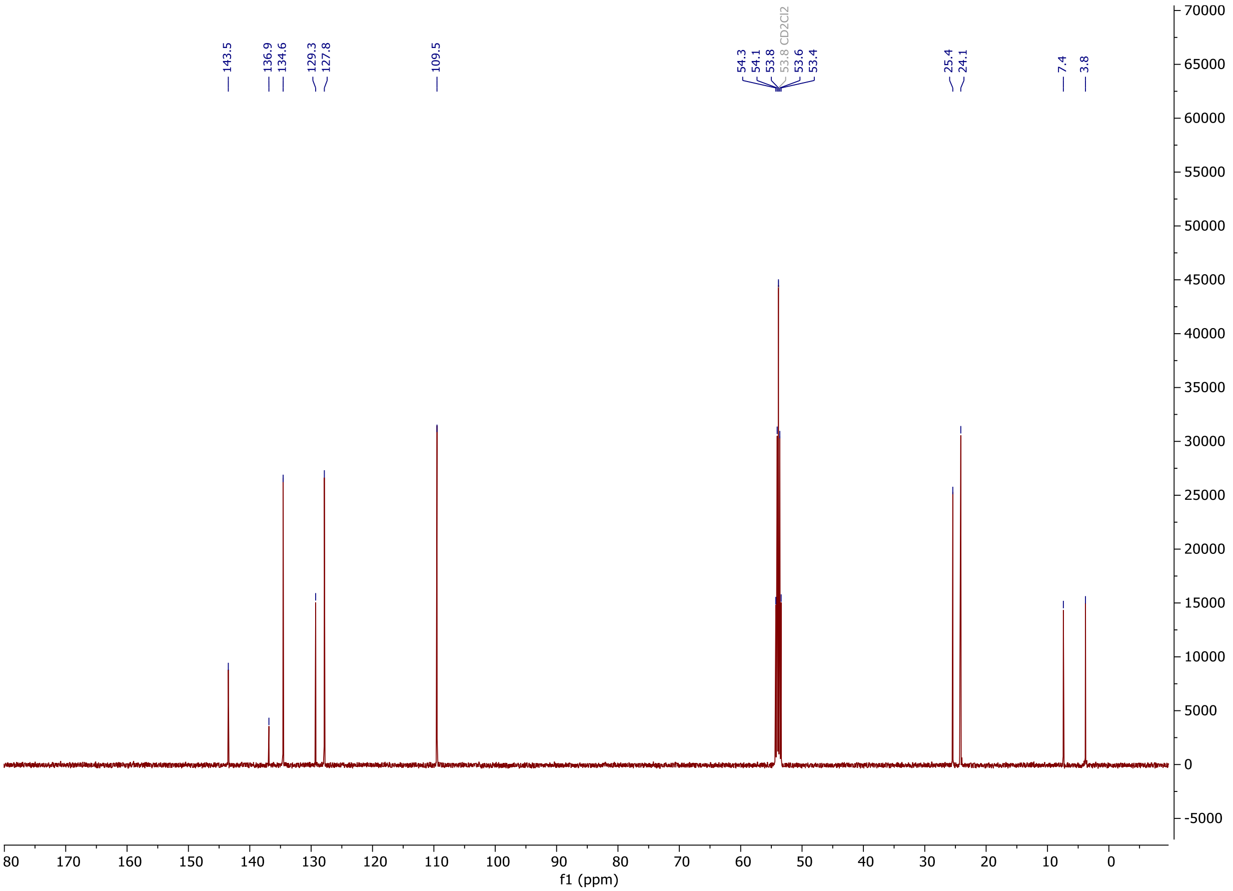

butylbis(2-methylallyl)(phenyl)silane 1c

1c

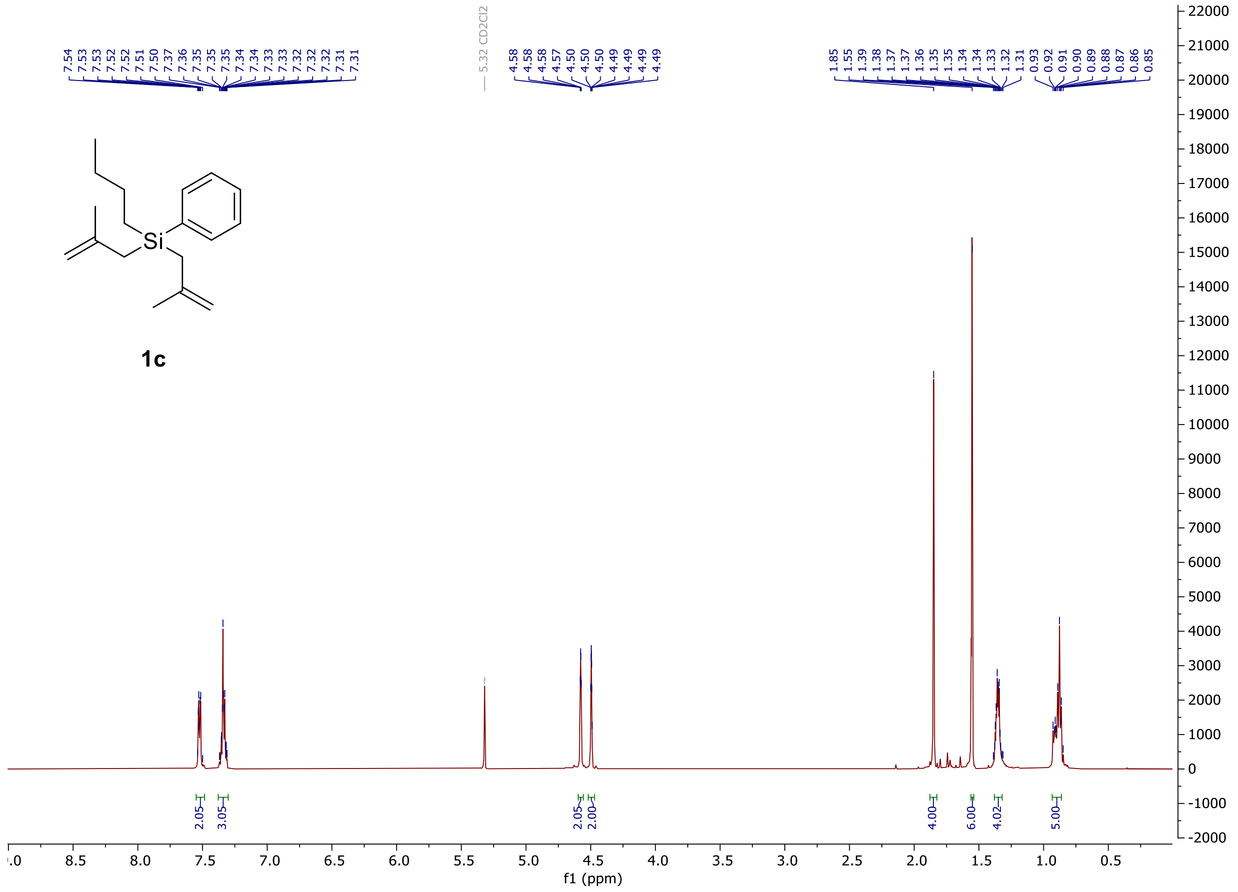

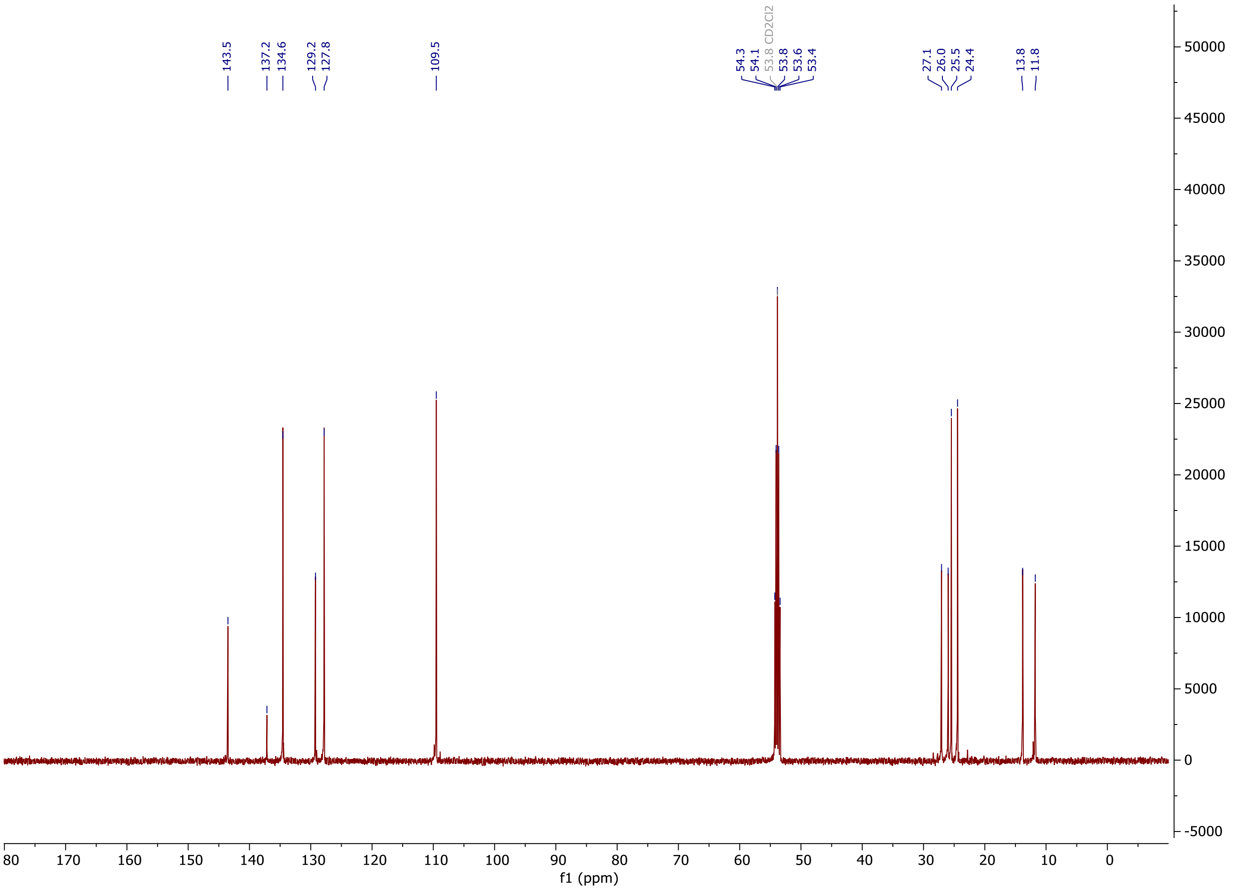

bis(2-methylallyl)(pentyl)(phenyl)silane 1d

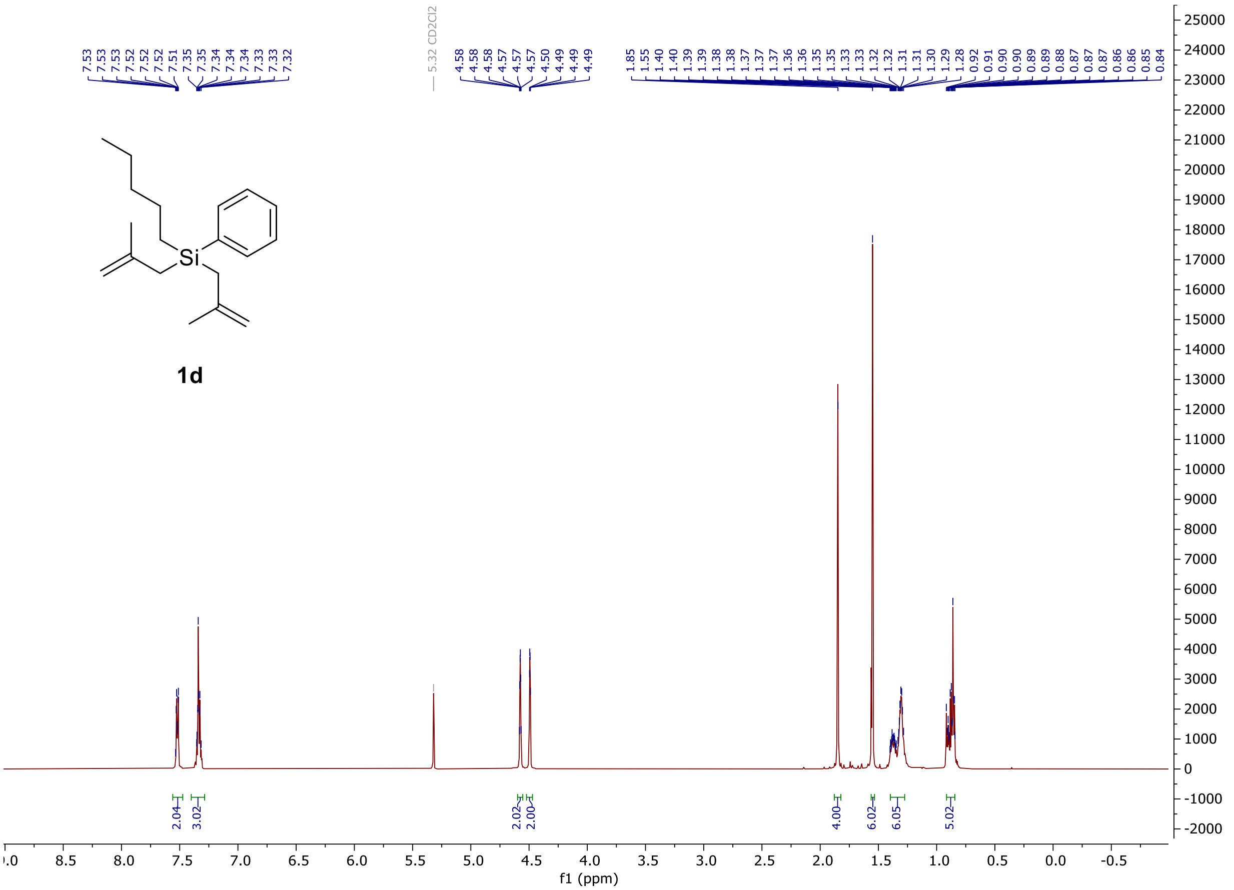

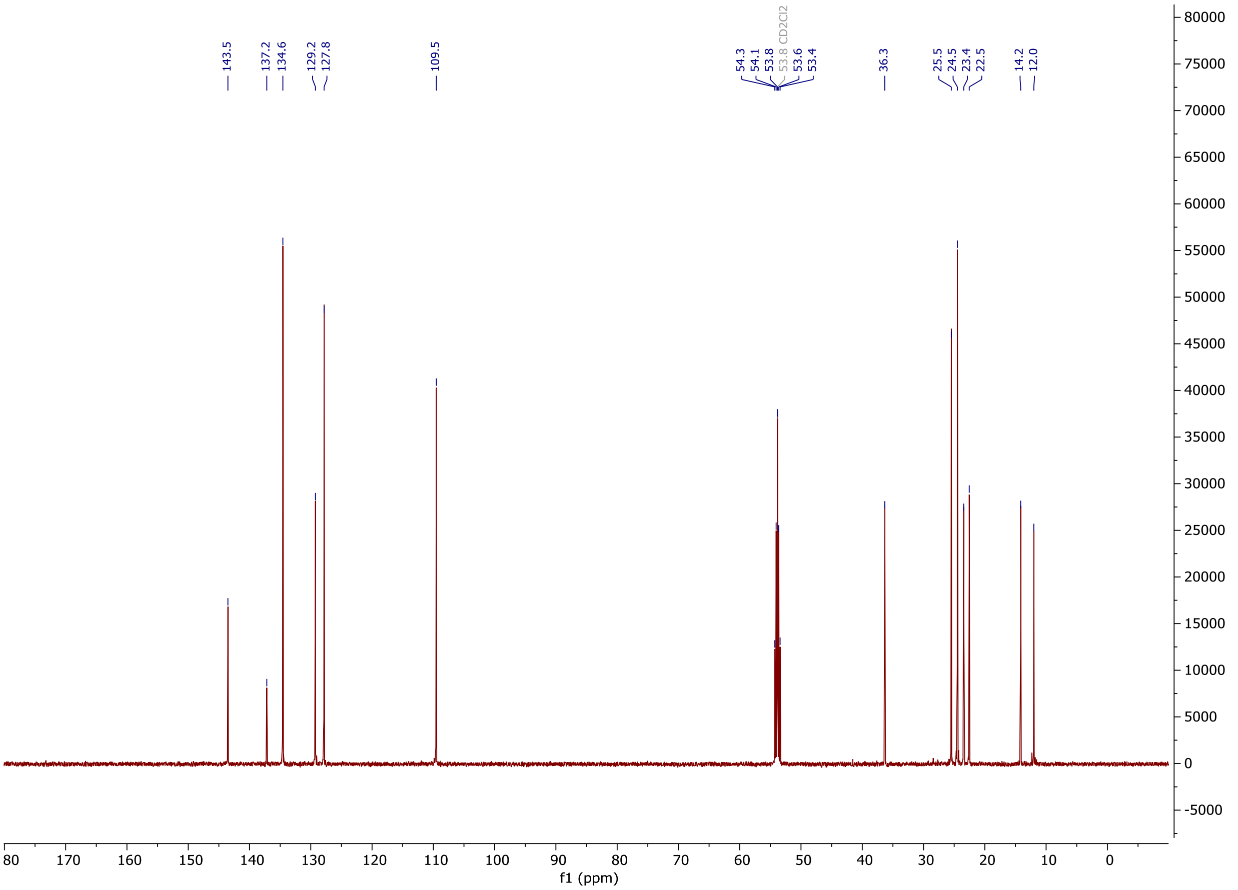

hexylbis(2-methylallyl)(phenyl)silane 1e

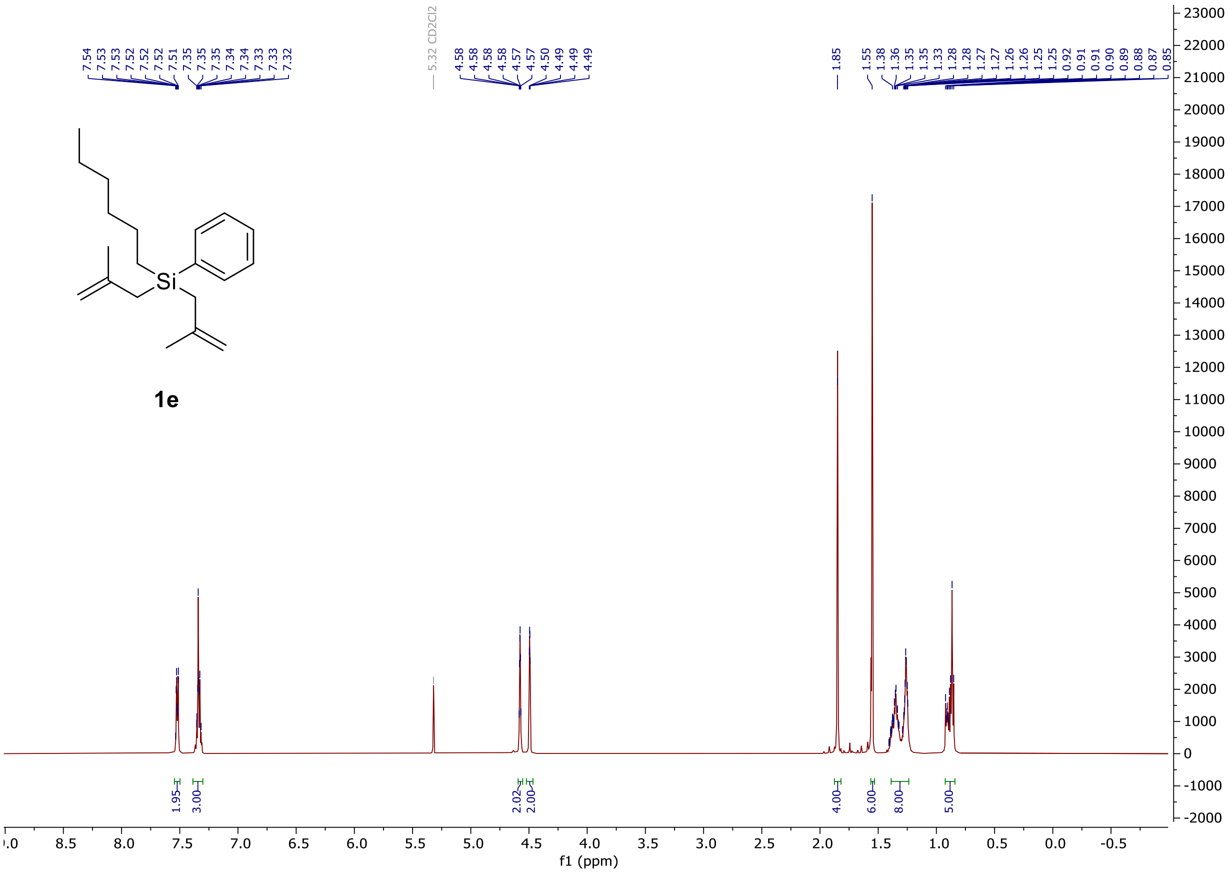

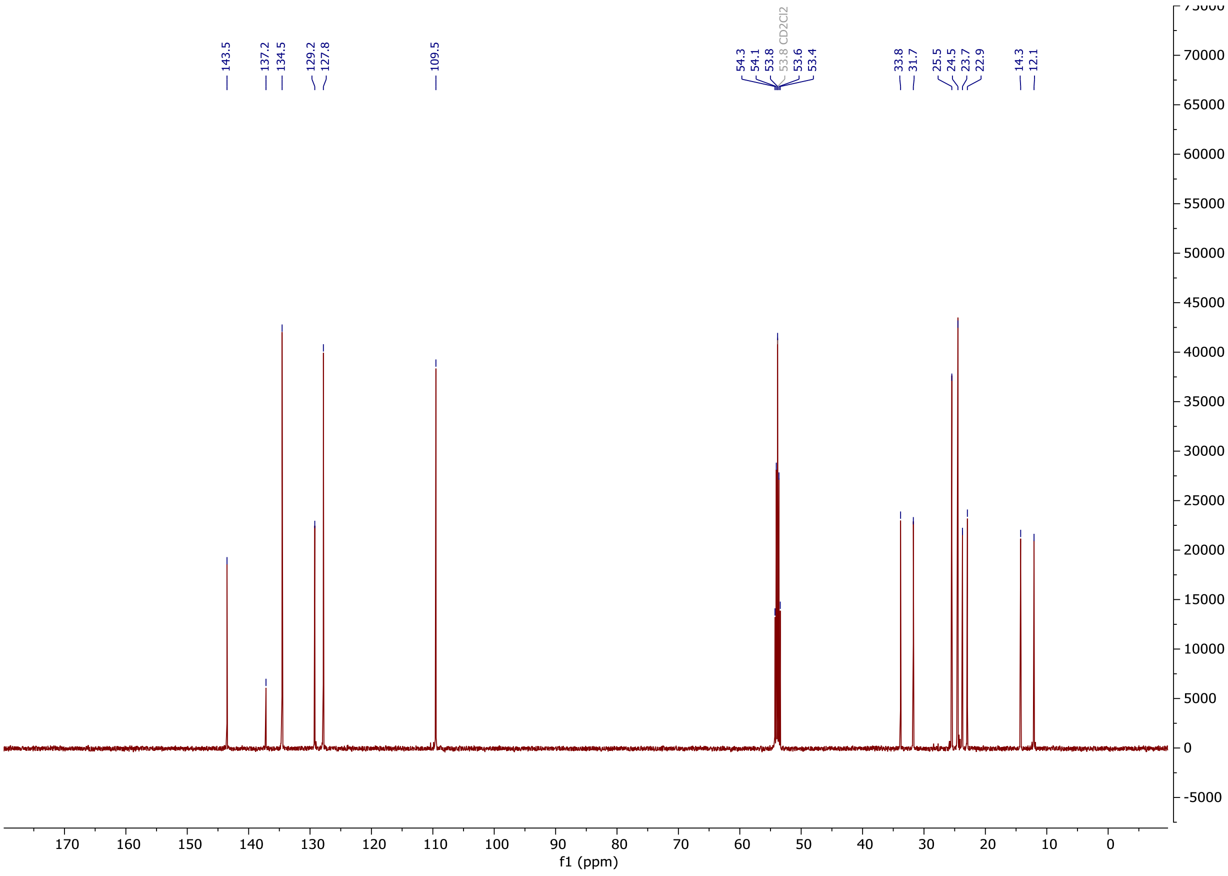

isopentylbis(2-methylallyl)(phenyl)silane 1f

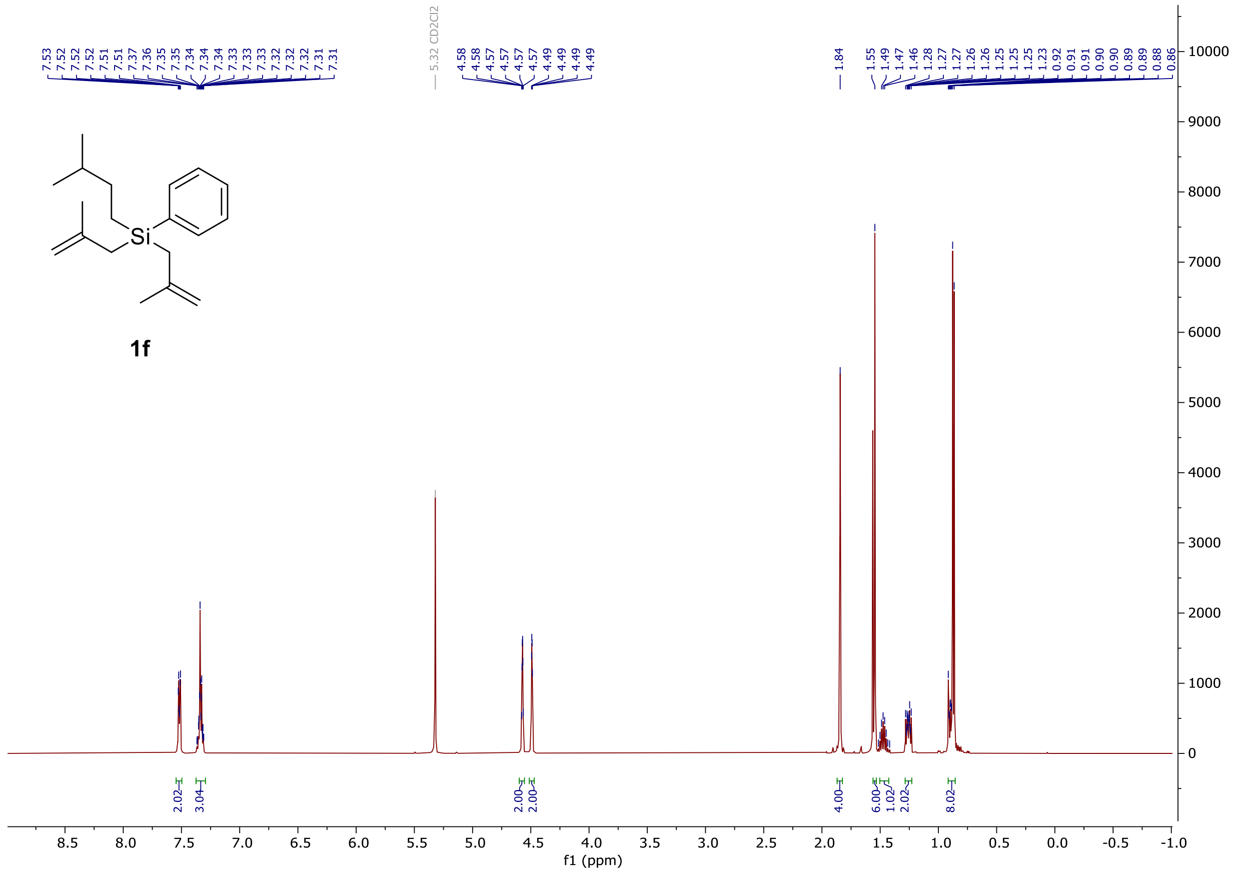

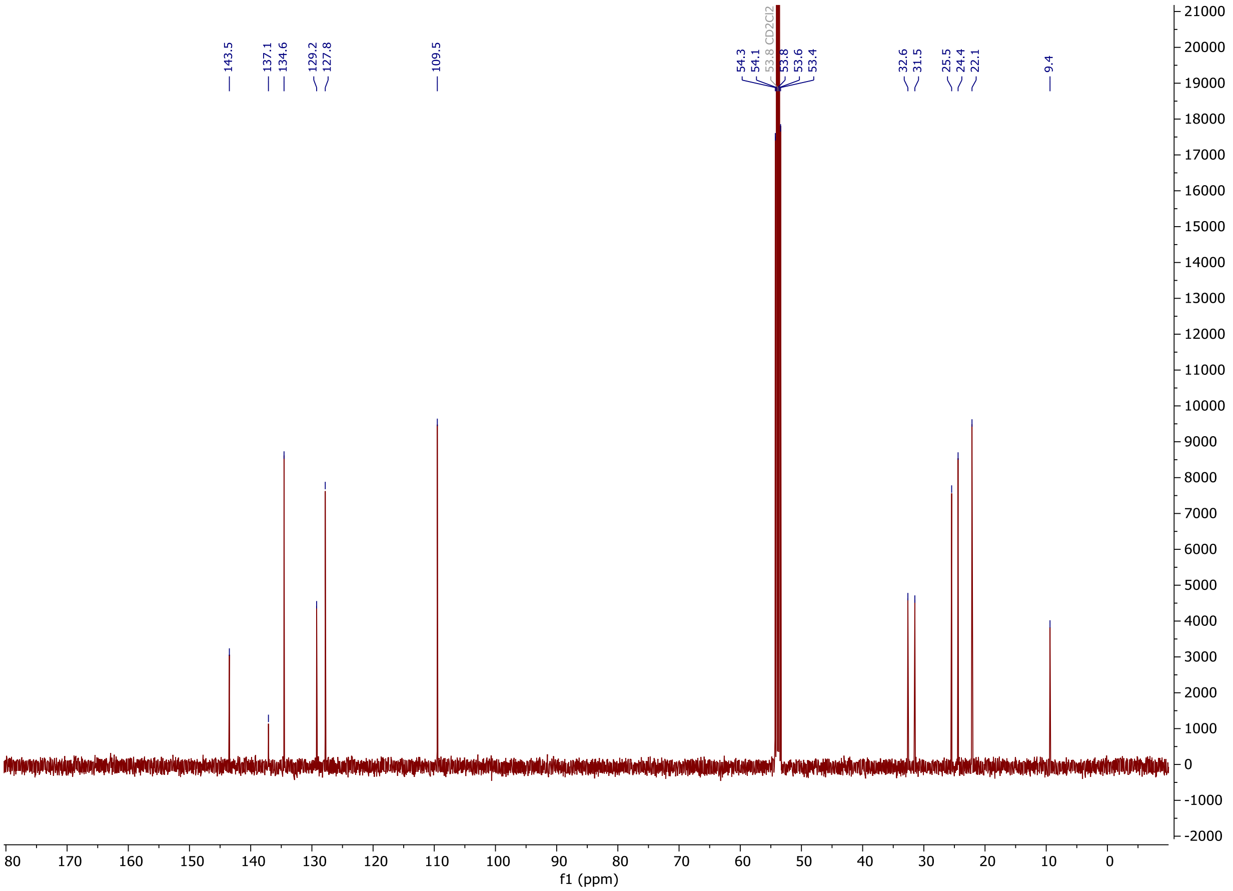

isobutylbis(2-methylallyl)(phenyl)silane 1g

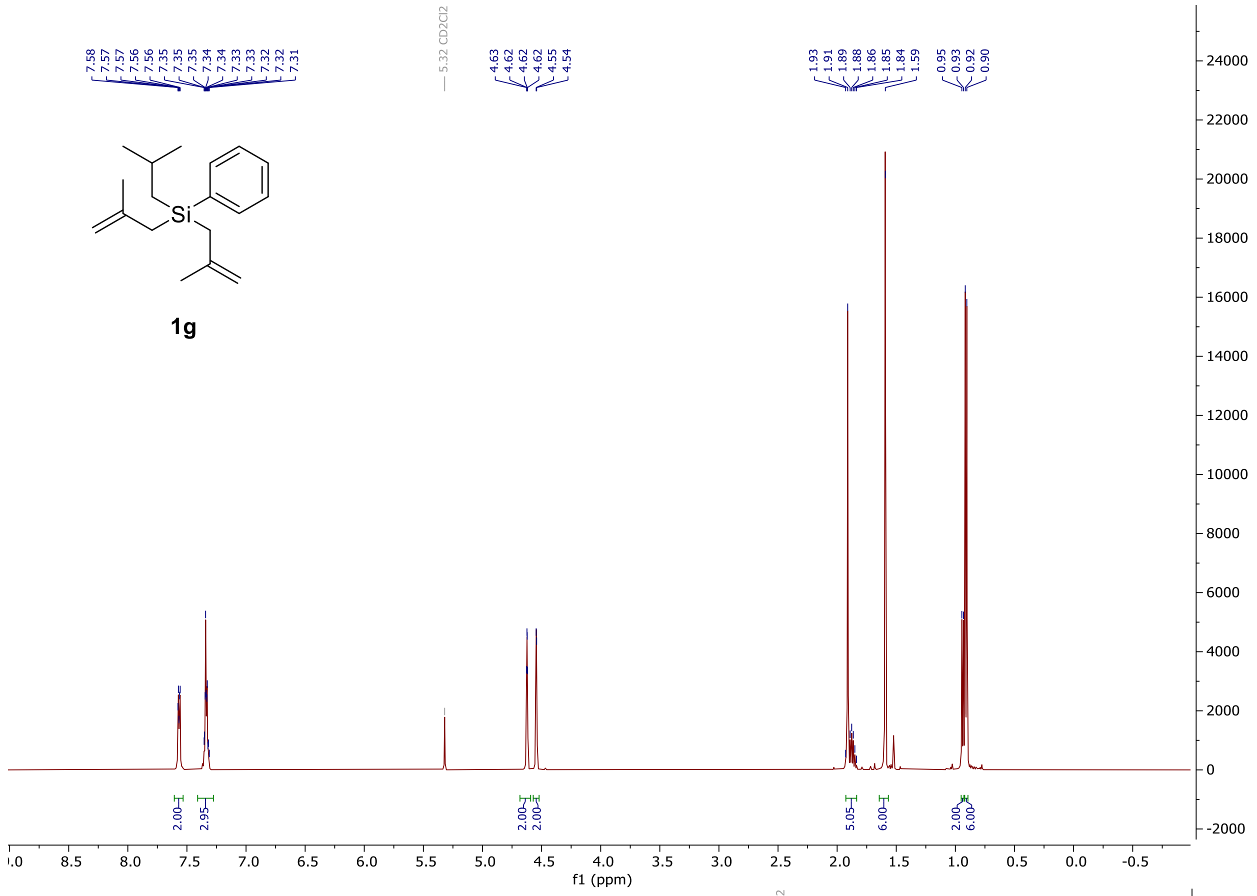

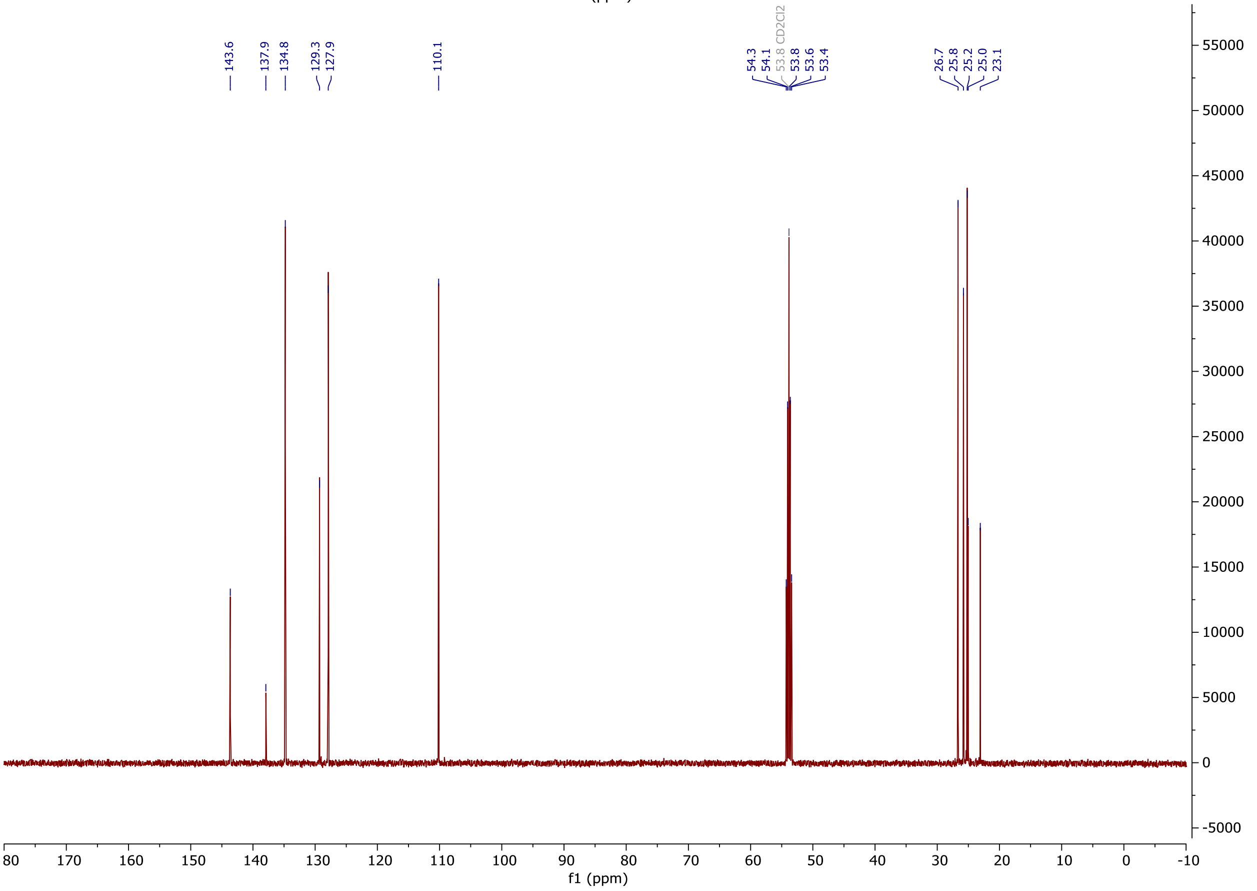

bis(2-methylallyl)(naphthalen-2-yl)(propyl)silane 1h

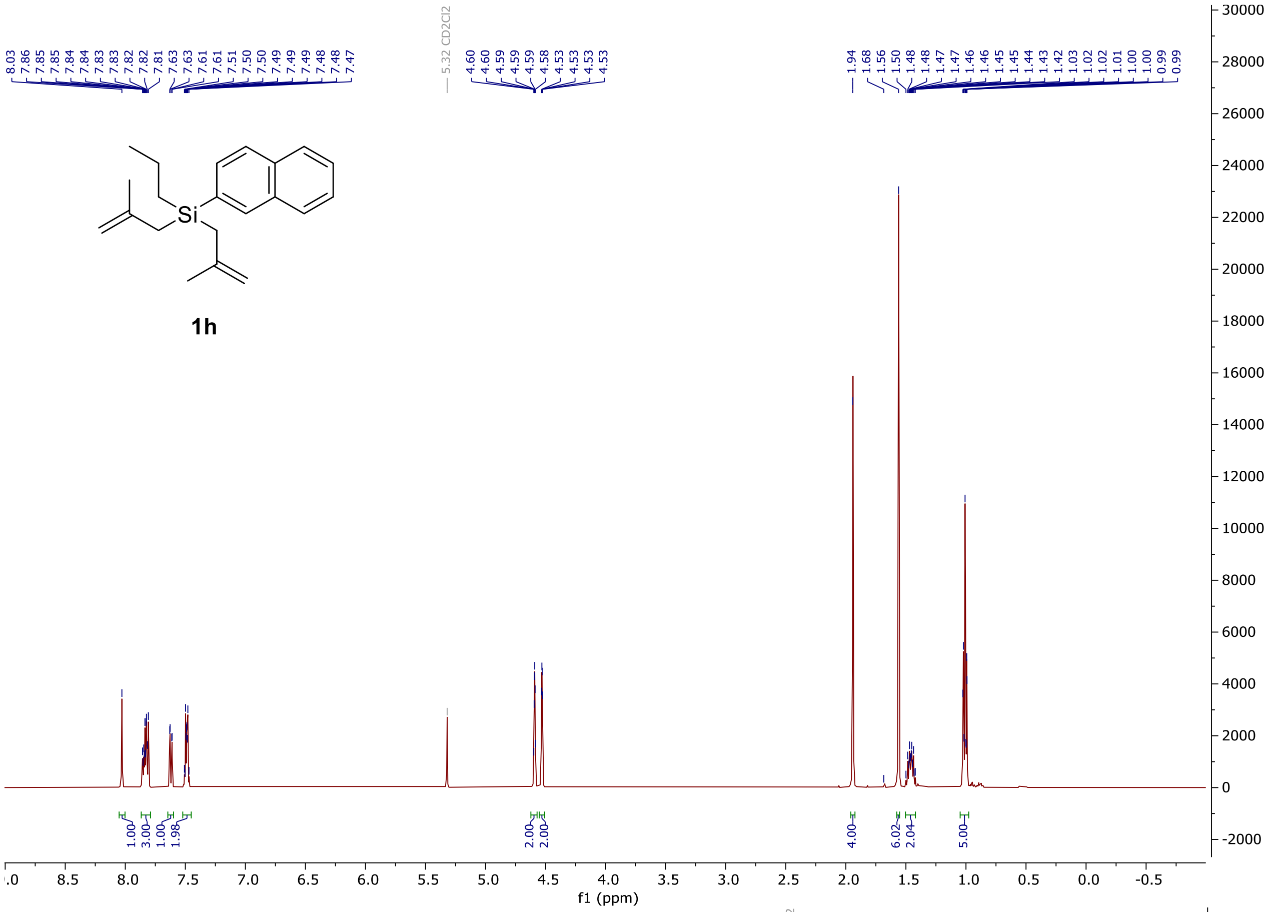

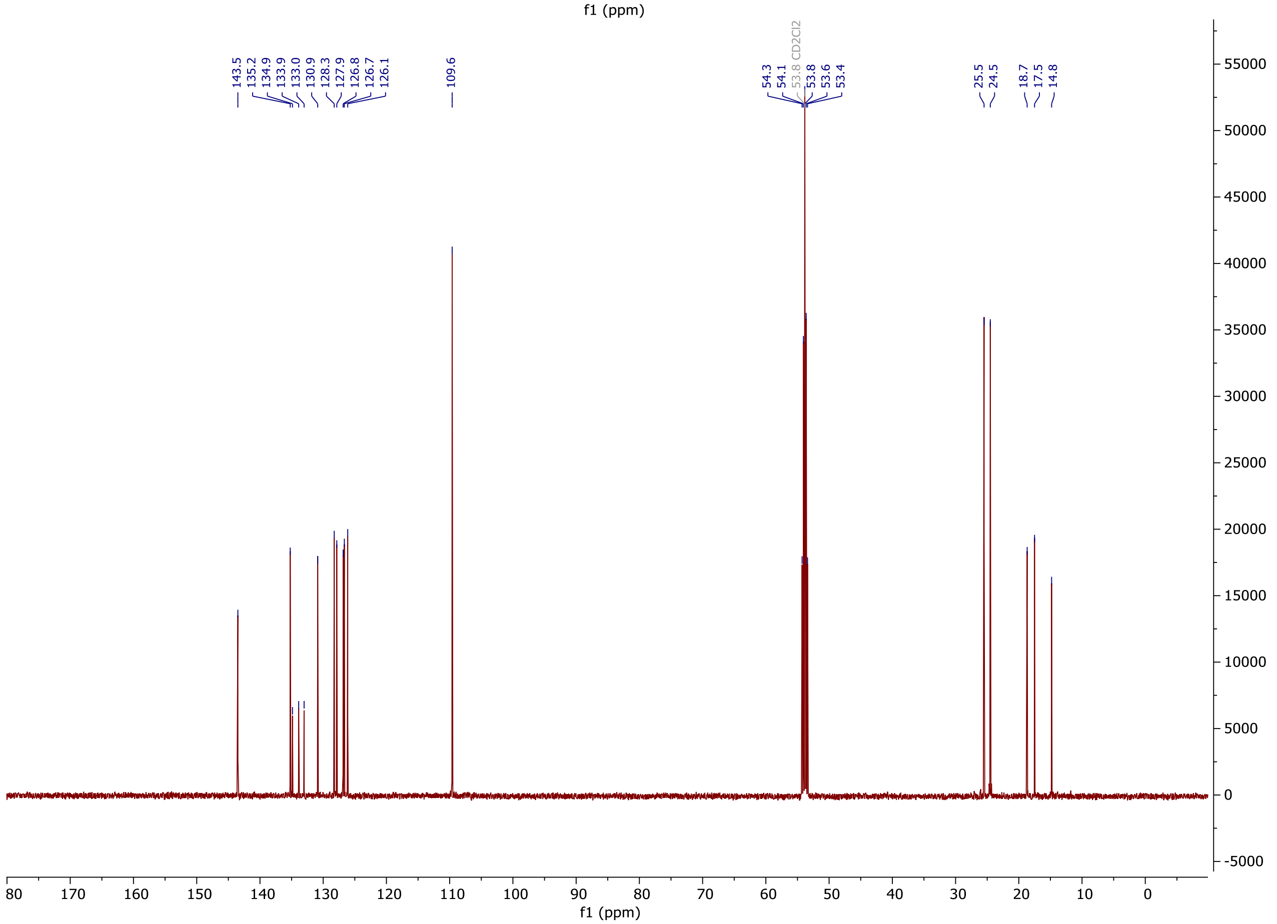

bis(2-methylallyl)(propyl)(*o*-tolyl)silane 1i

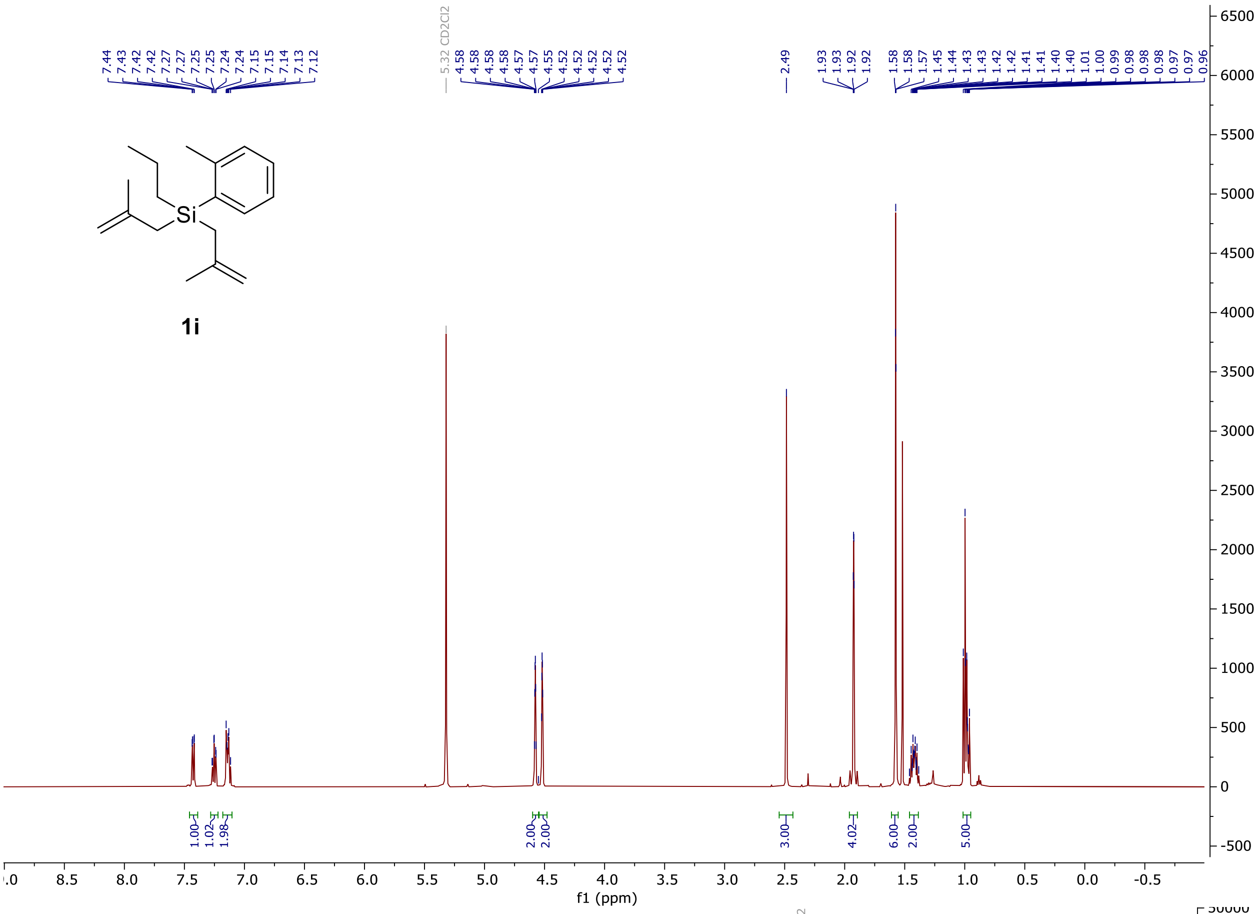

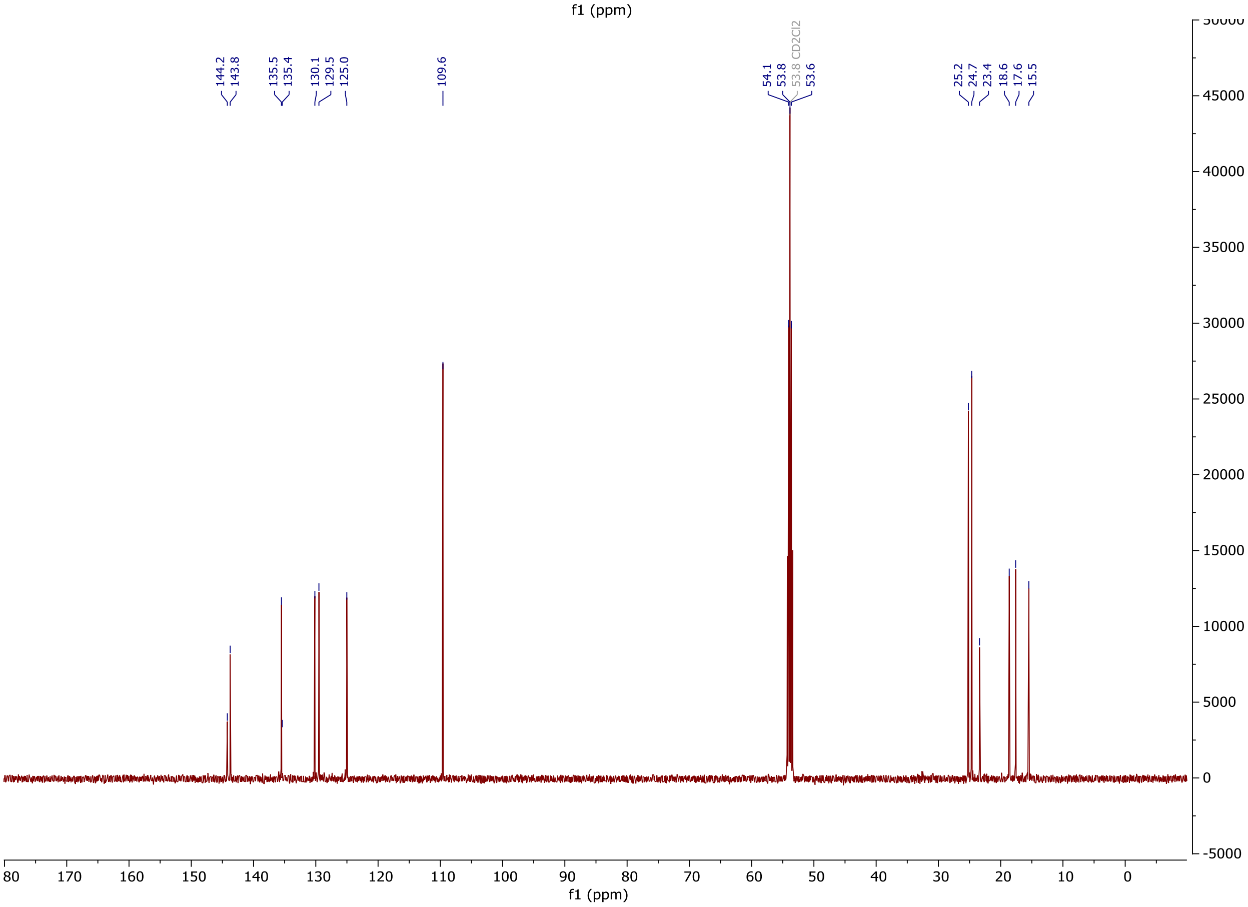

bis(2-methylallyl)(propyl)(*m*-tolyl)silane 1j

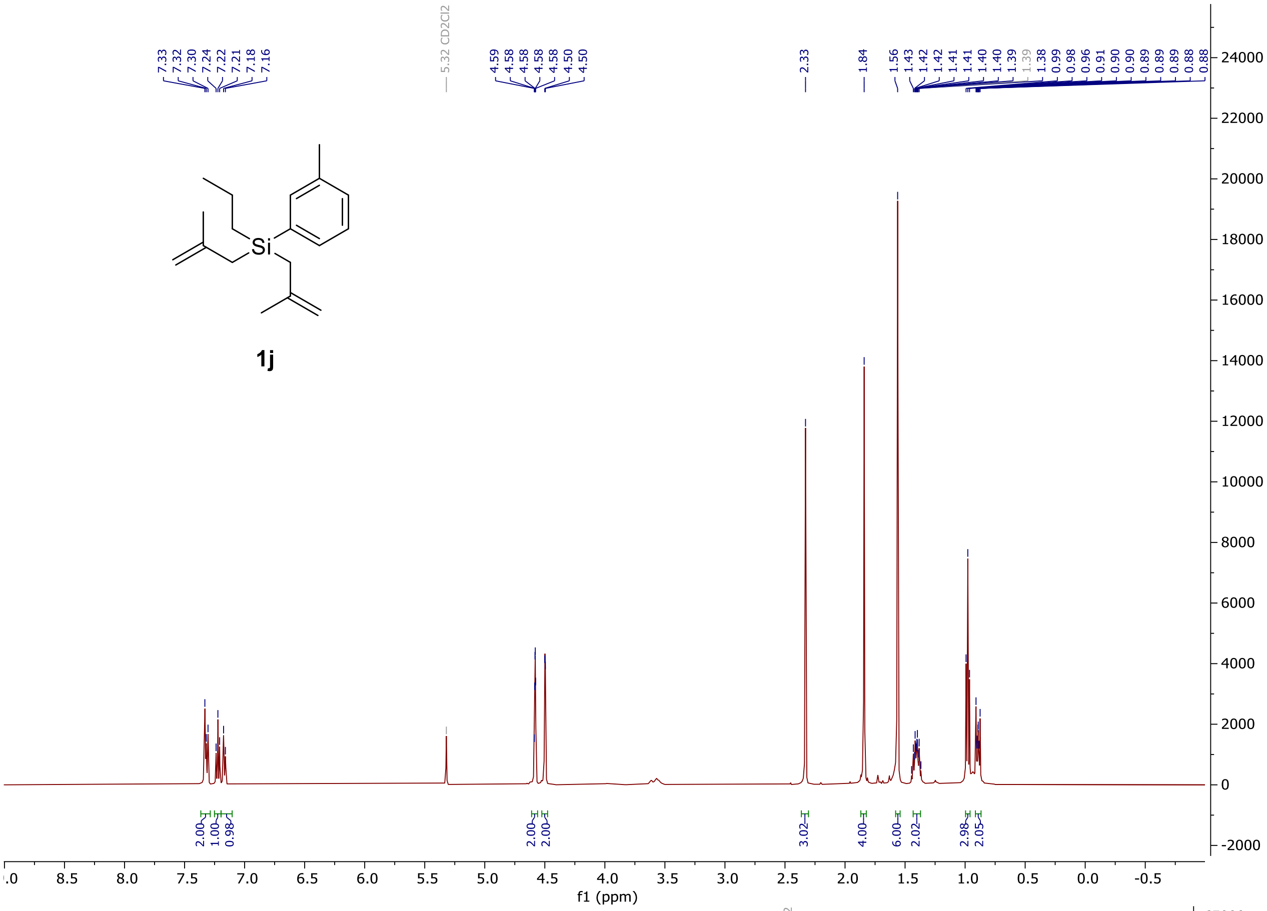

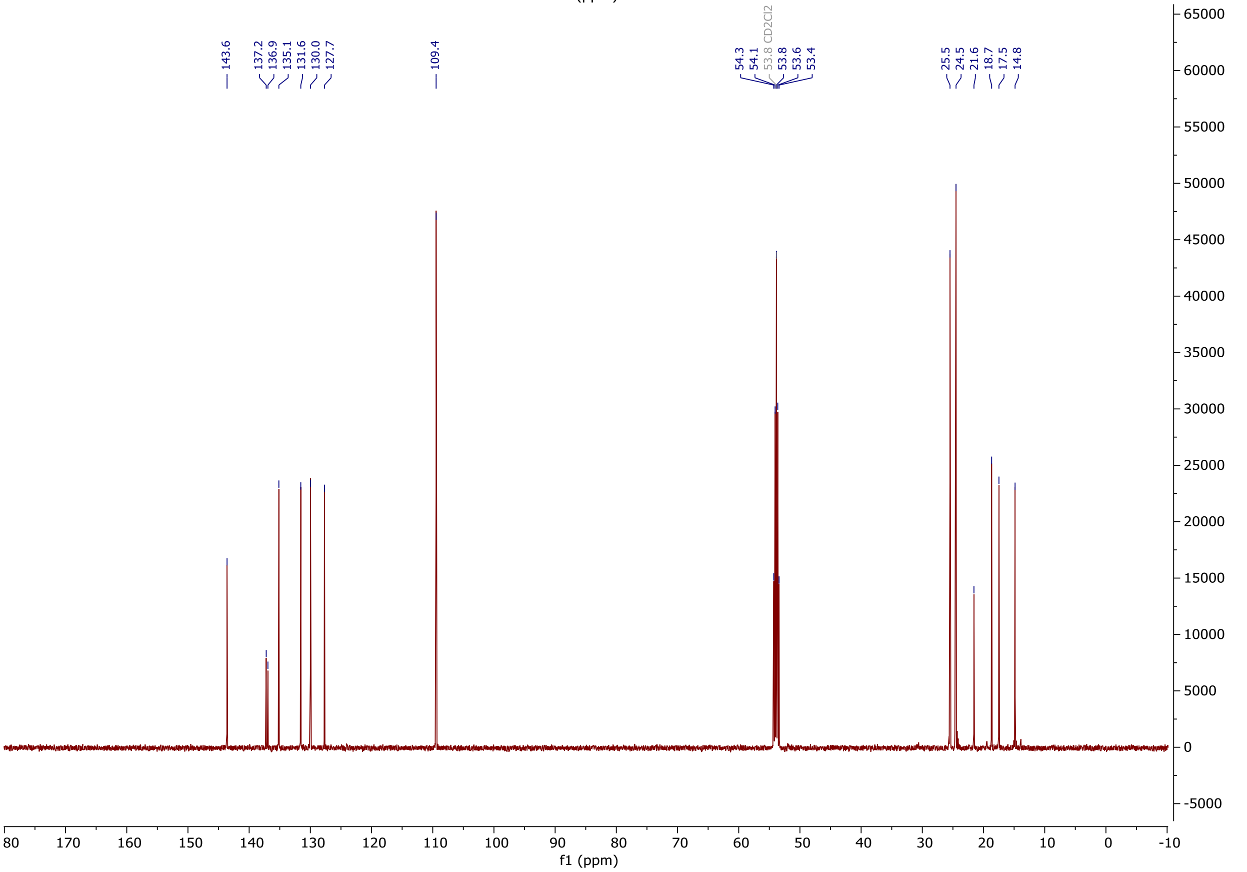

bis(2-methylallyl)(propyl)(*p*-tolyl)silane 1k

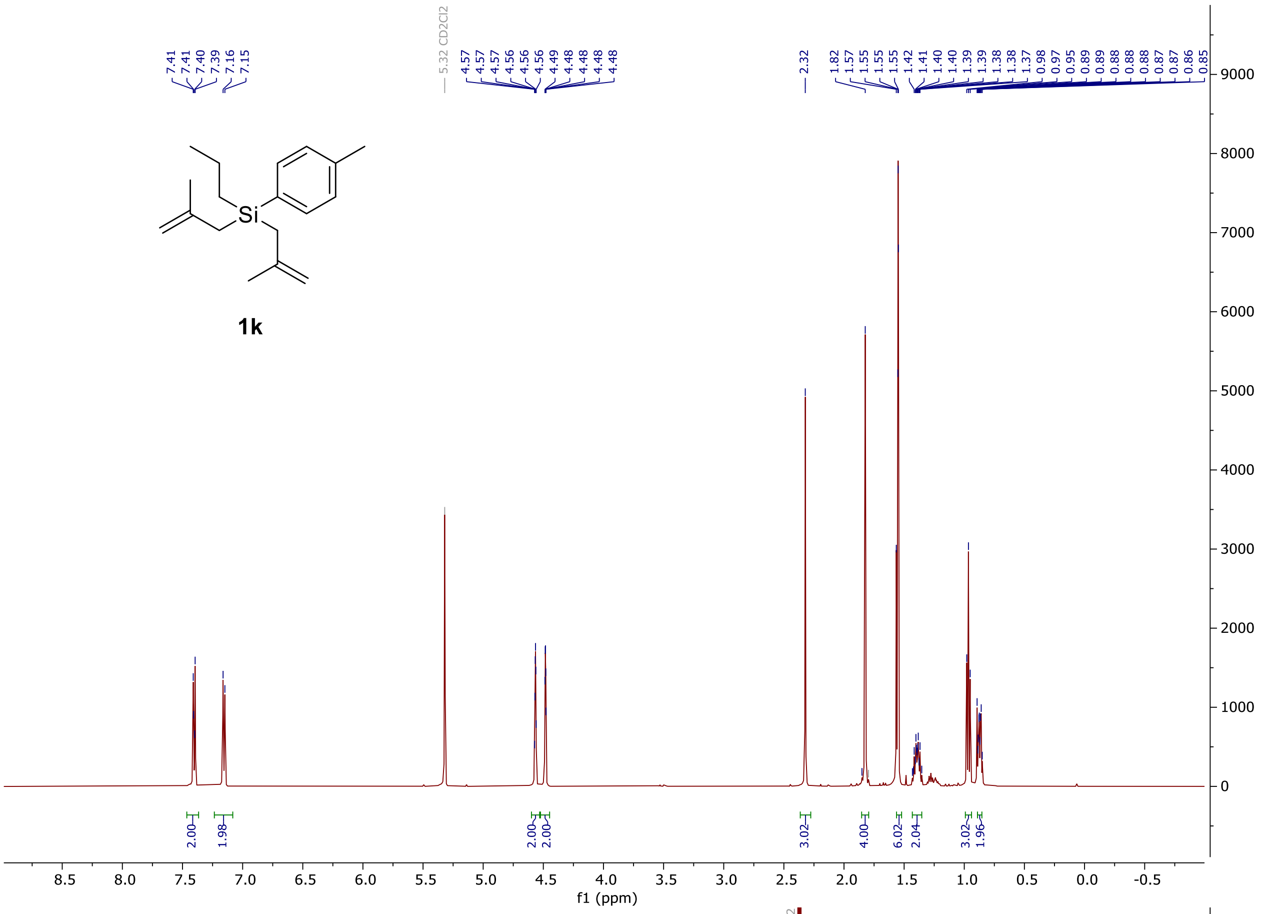

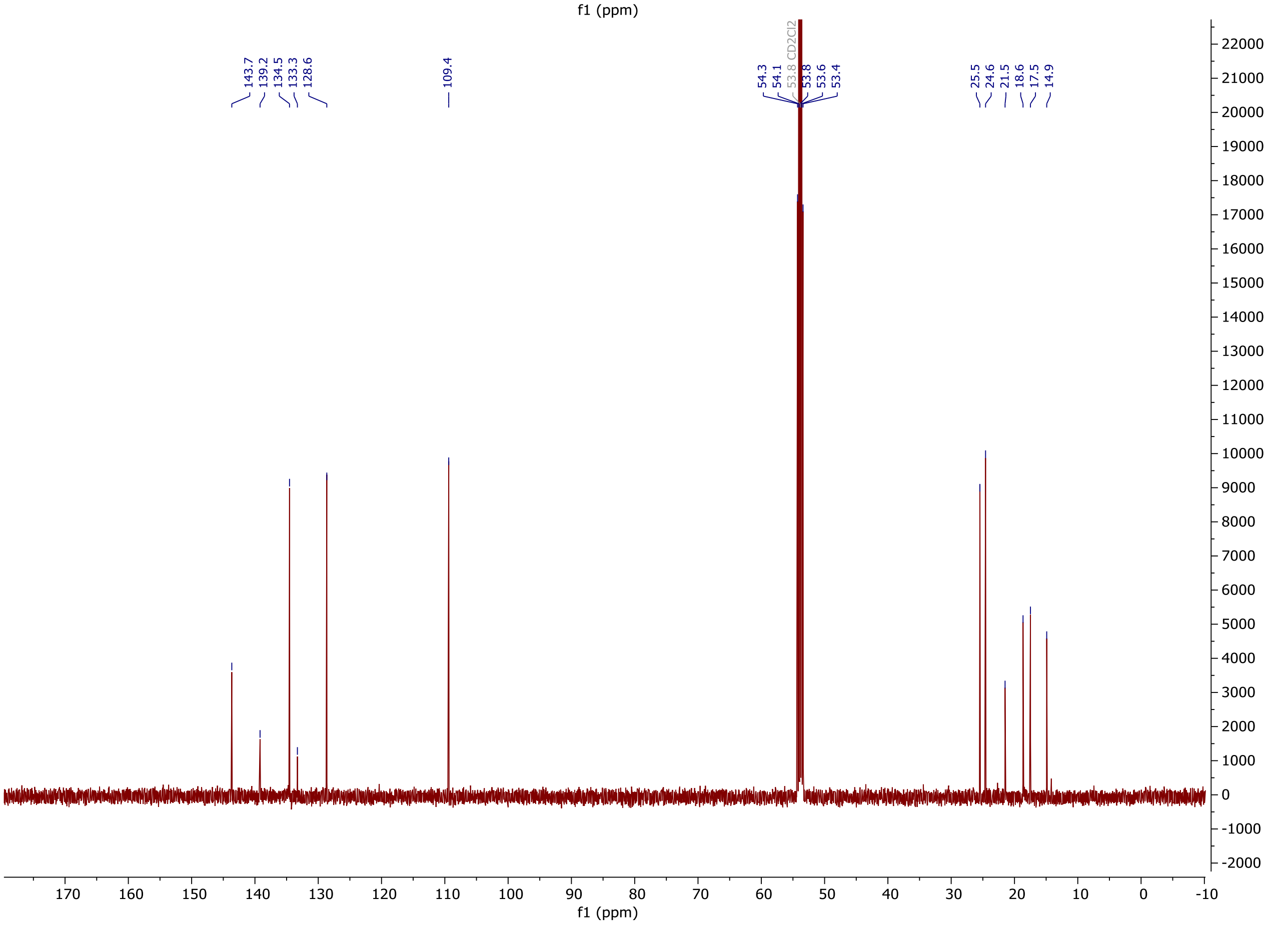

(4-methoxyphenyl)bis(2-methylallyl)(propyl)silane 1l

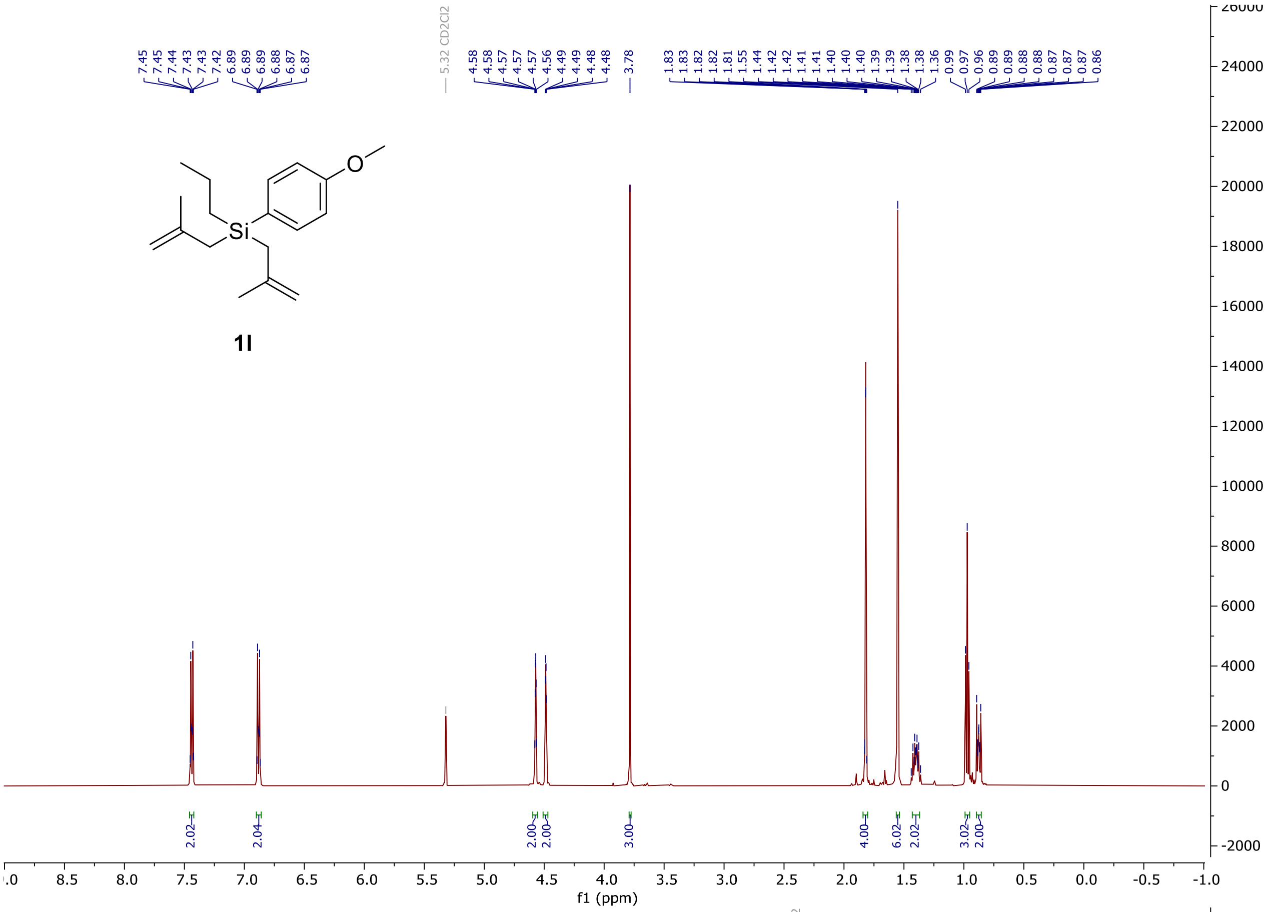

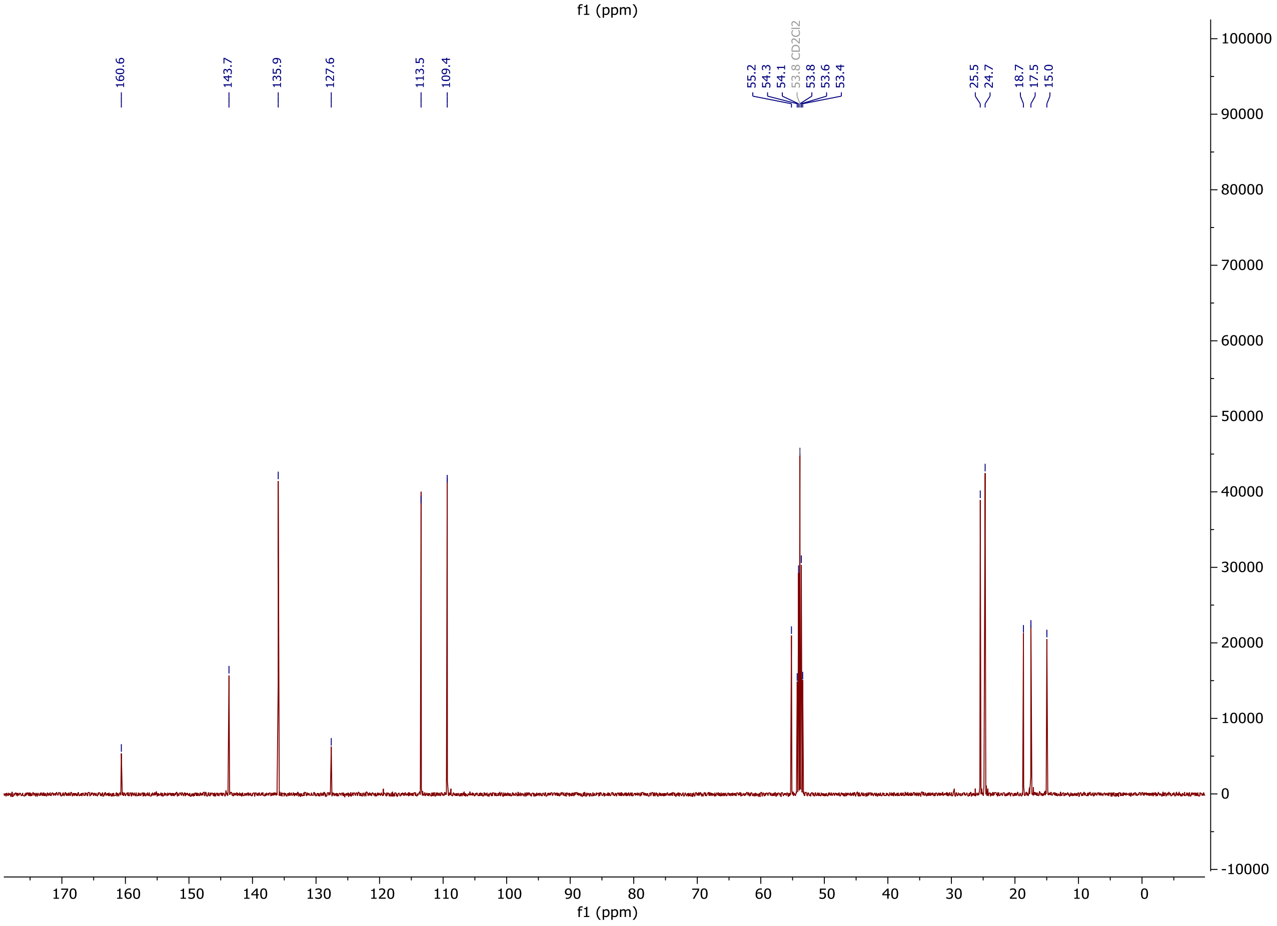

(*S*)-1-(4-fluorophenyl)-3,3-dimethyl-5-methylene-1-propylsilinane (1m)

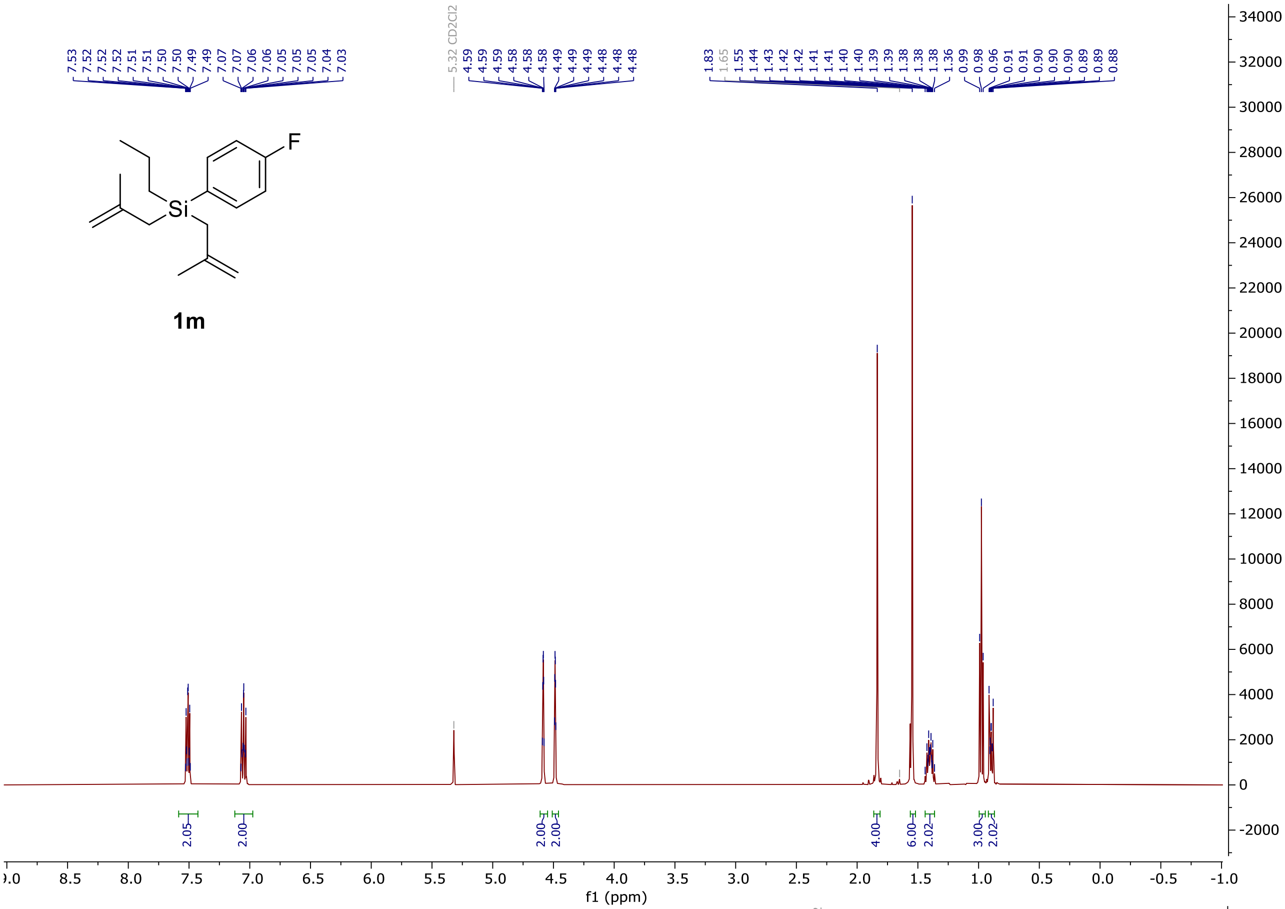

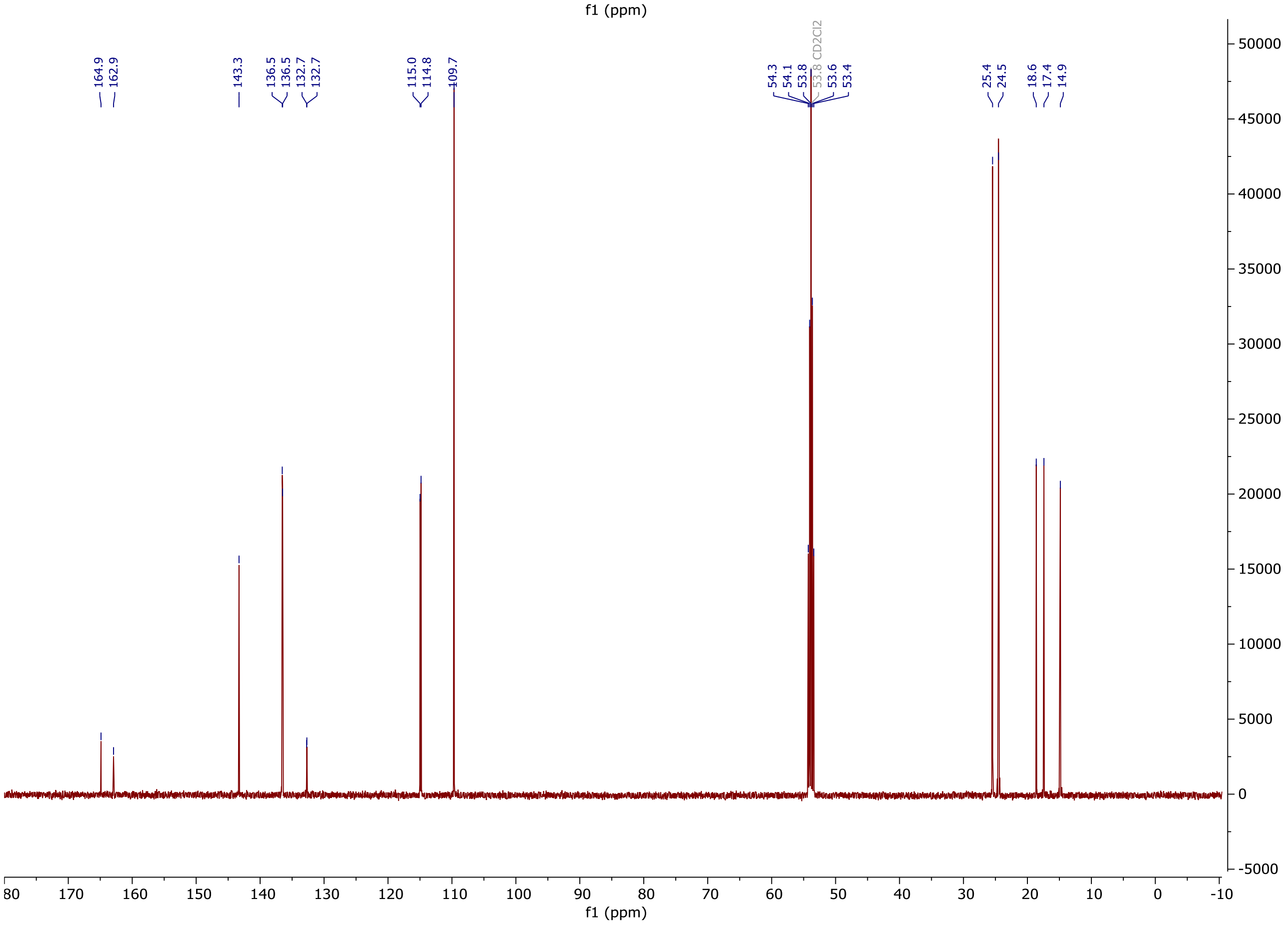

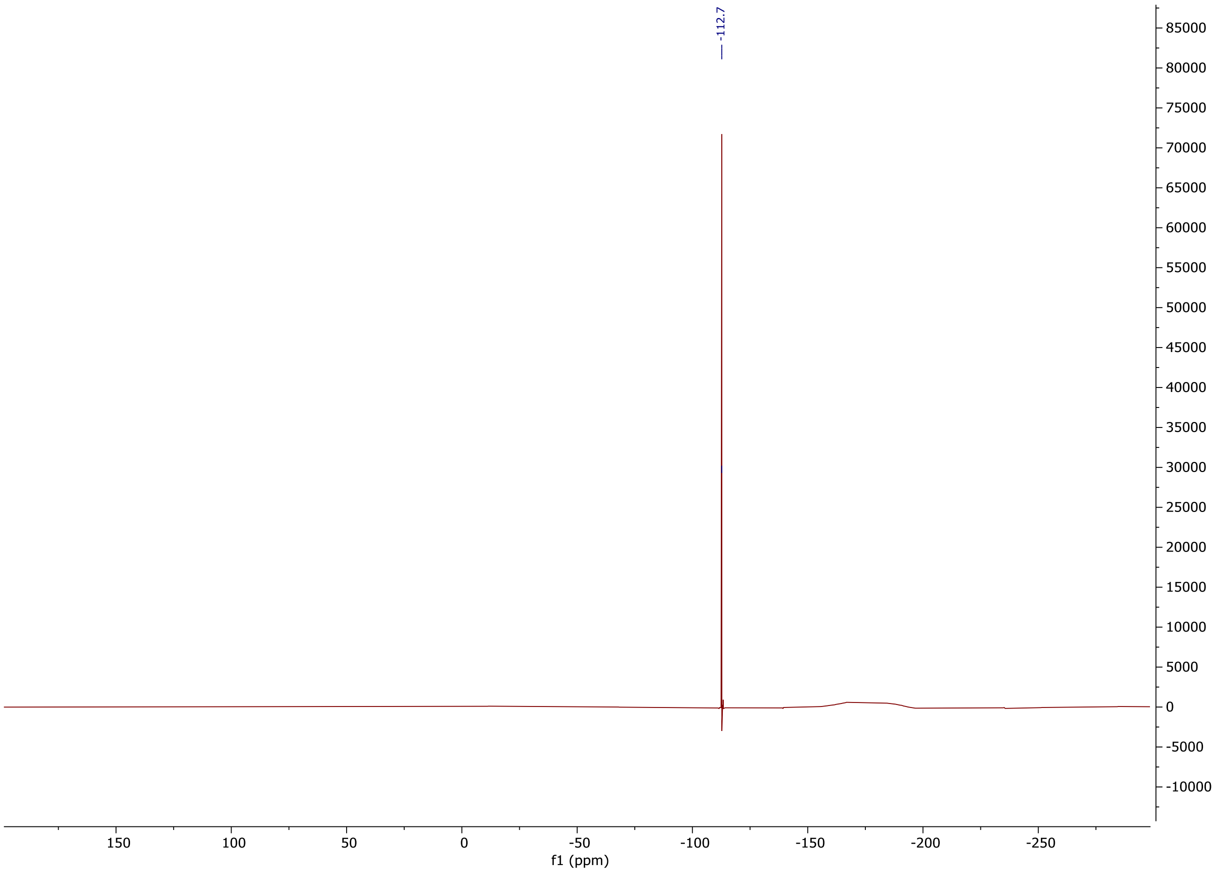

allylbis(2-methylallyl)(phenyl)silane 1n

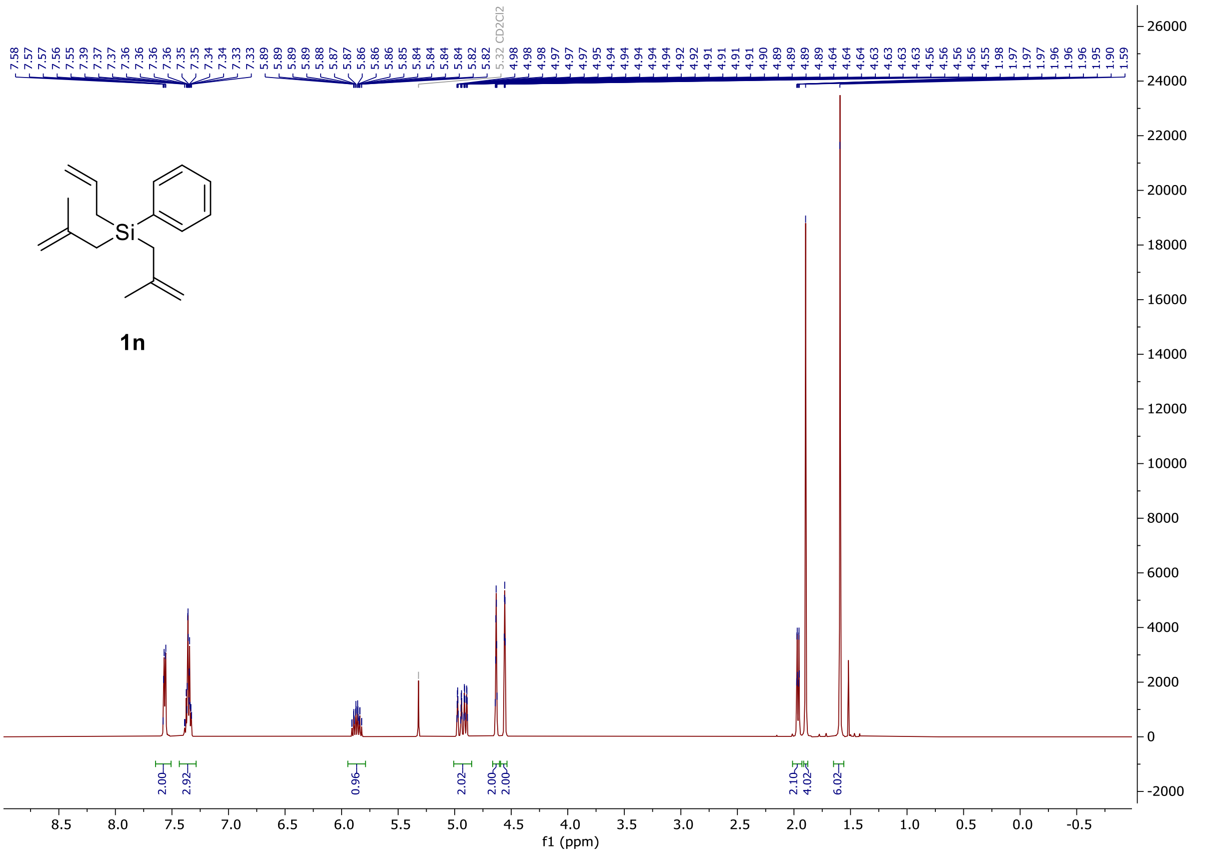

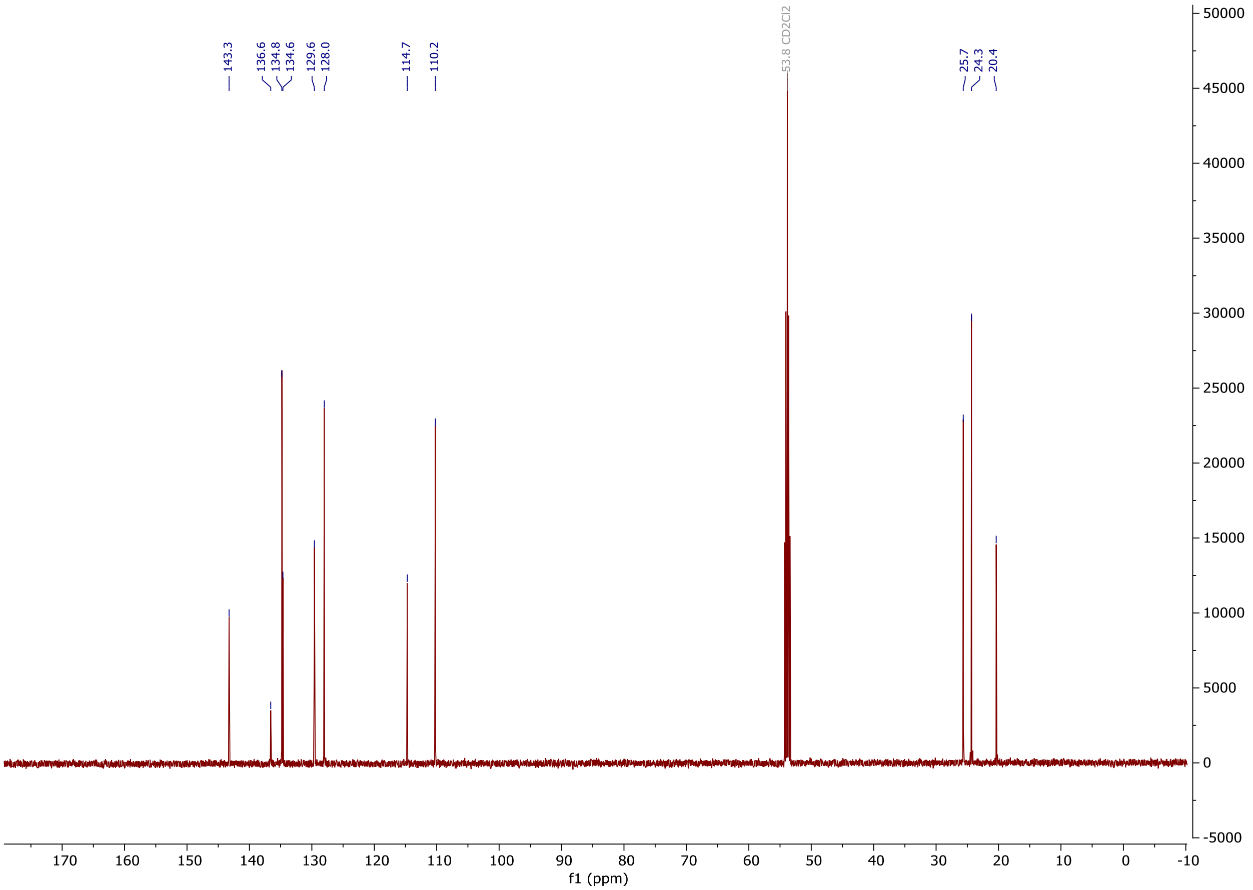

tris(2-methylallyl)(phenyl)silane 1o

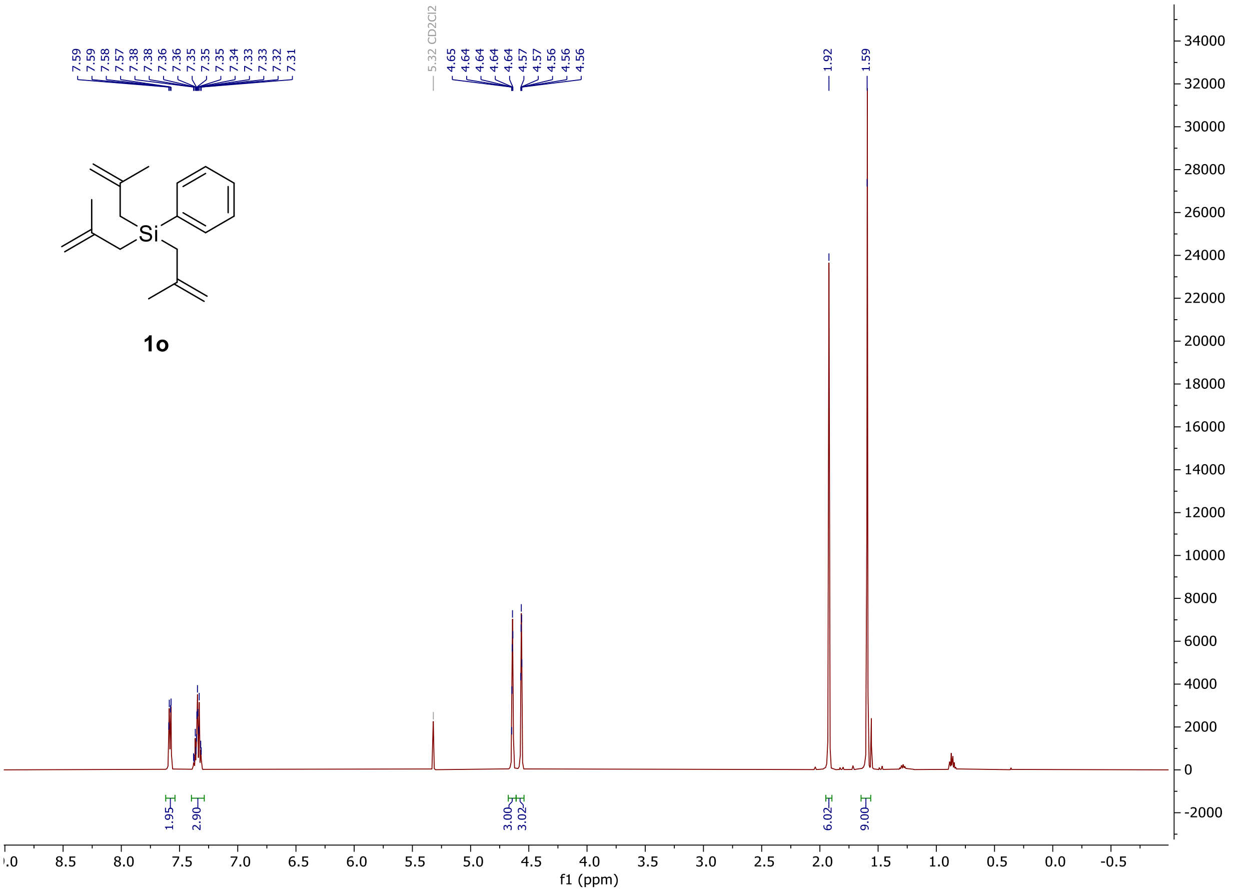

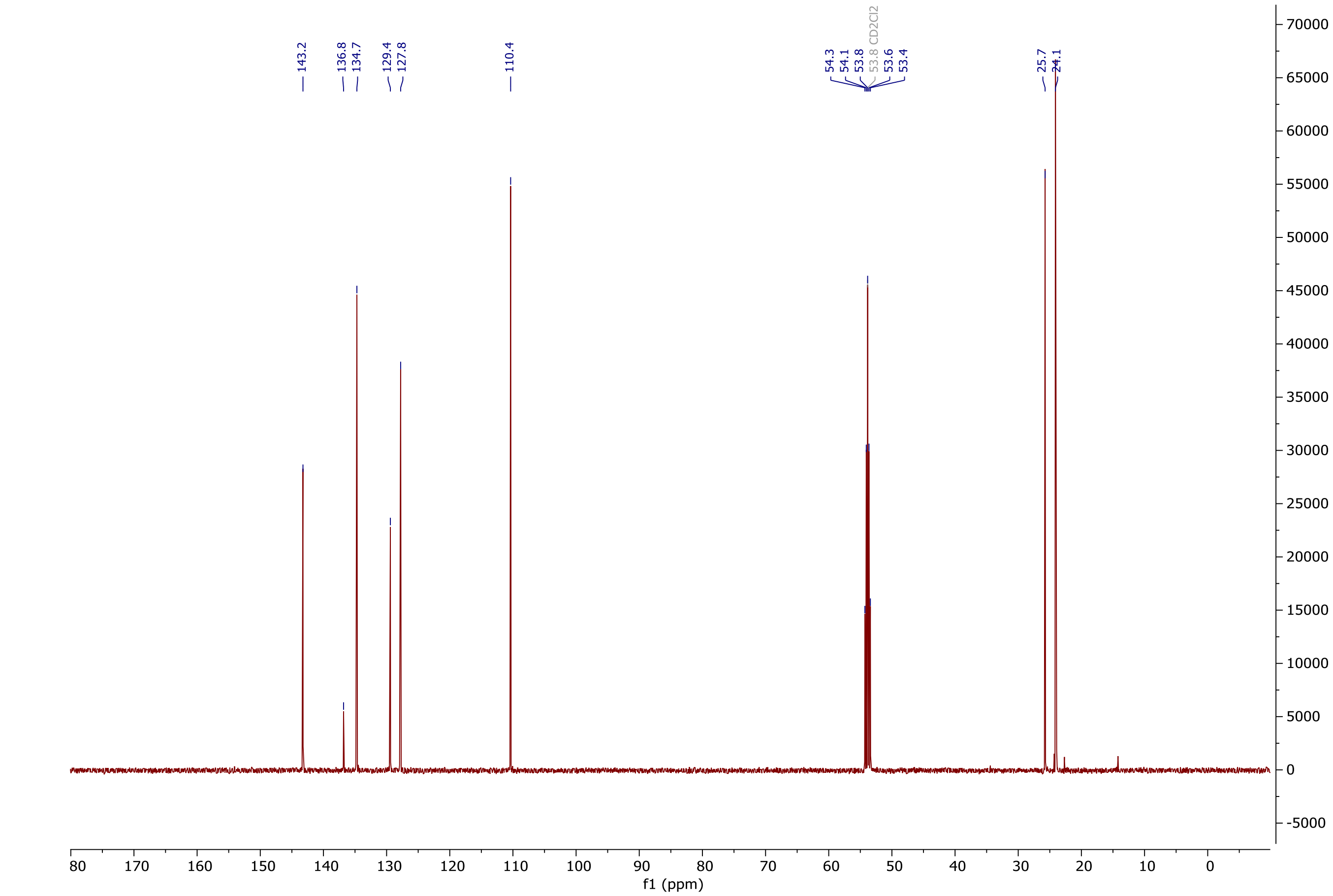

cyclohexylbis(2-methylallyl)(propyl)silane 1p

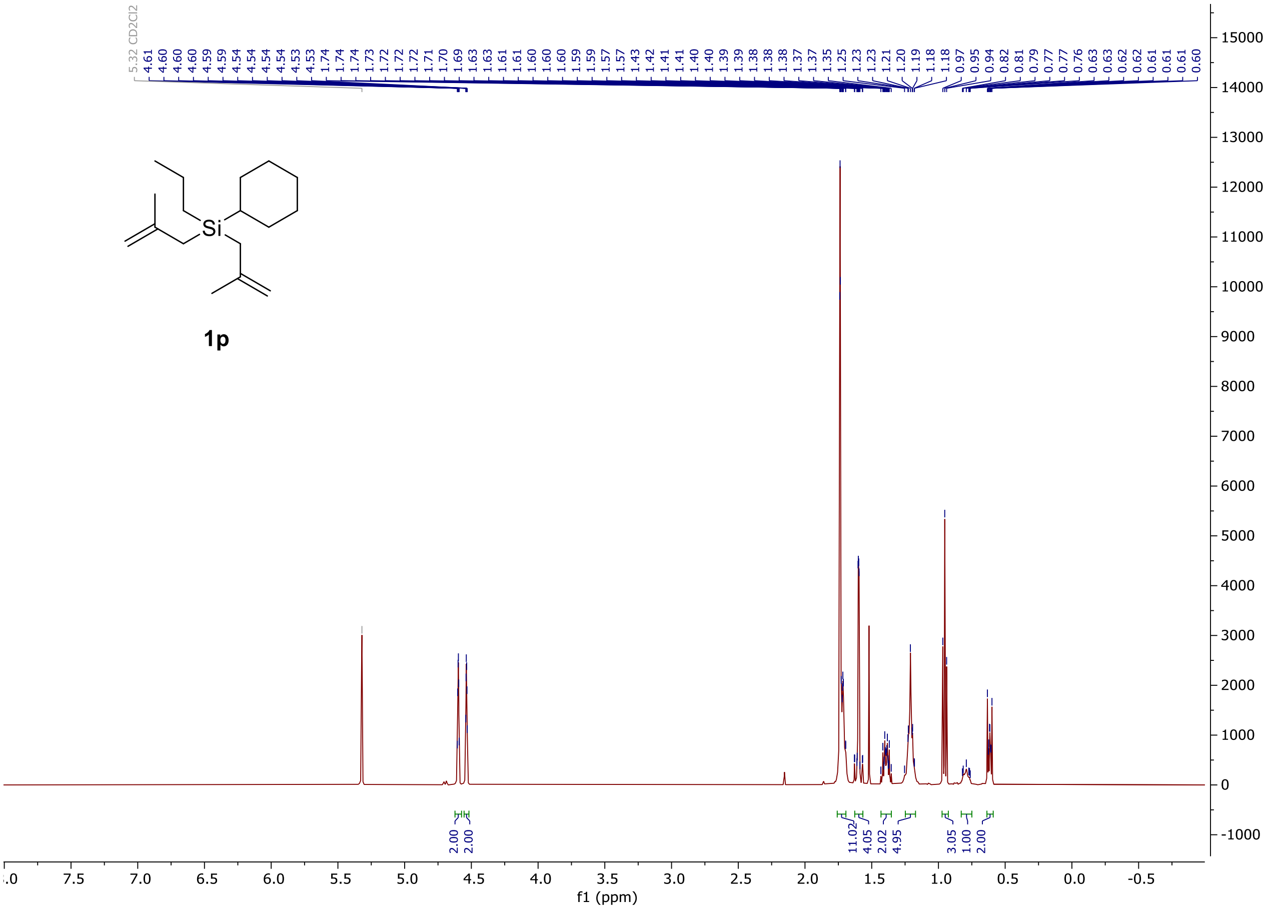

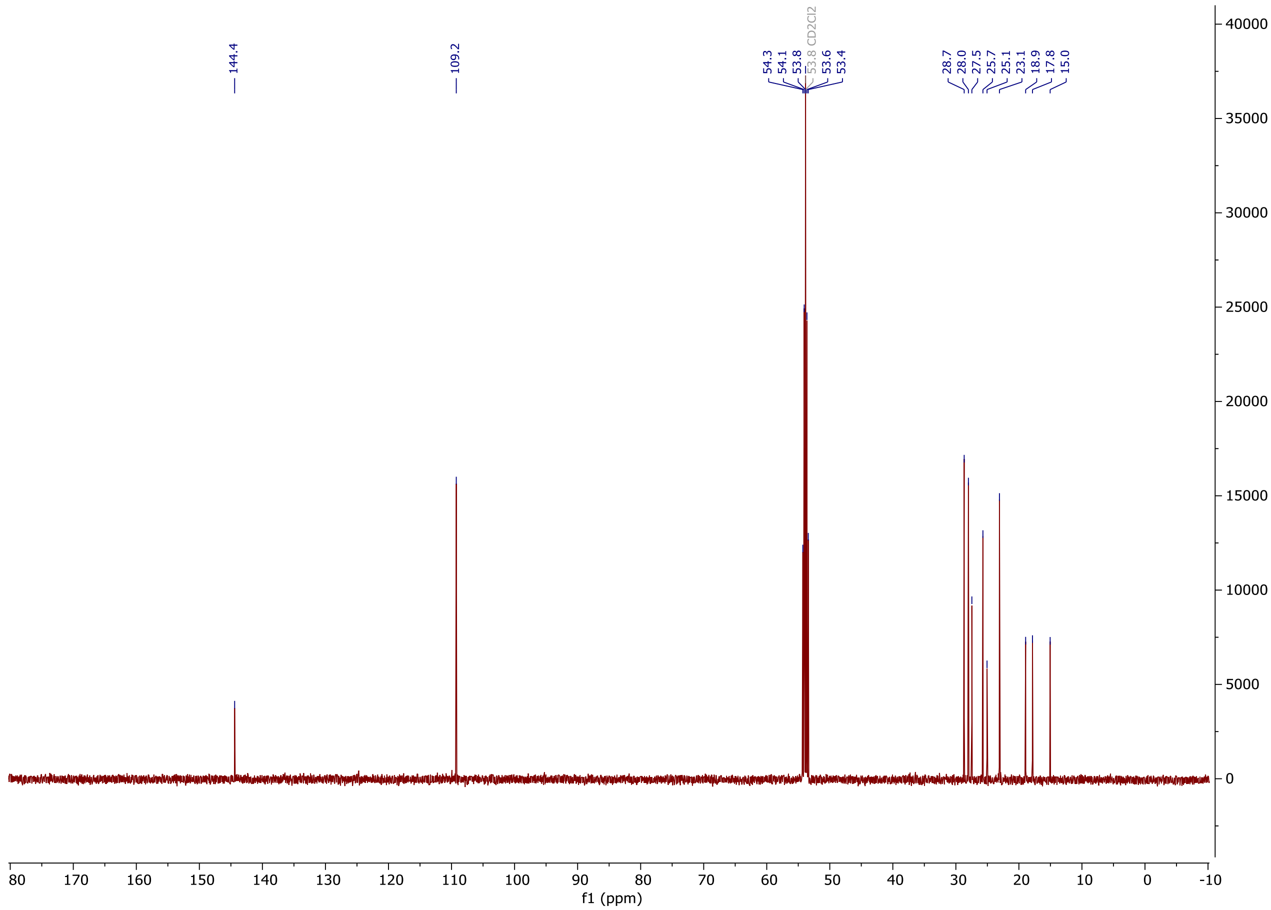

(*S*)-3,3-dimethyl-5-methylene-1-phenyl-1-propylsilinane 2a

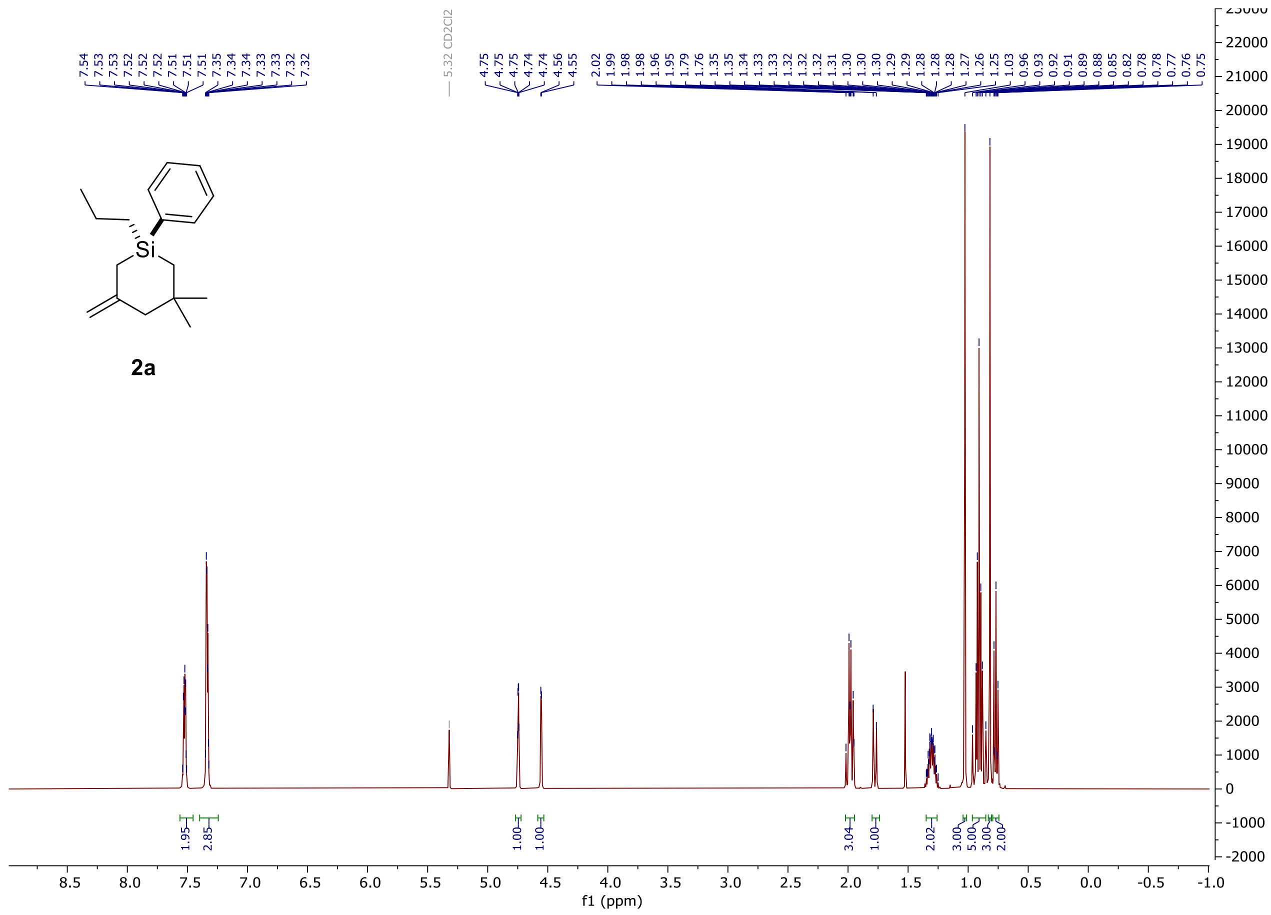

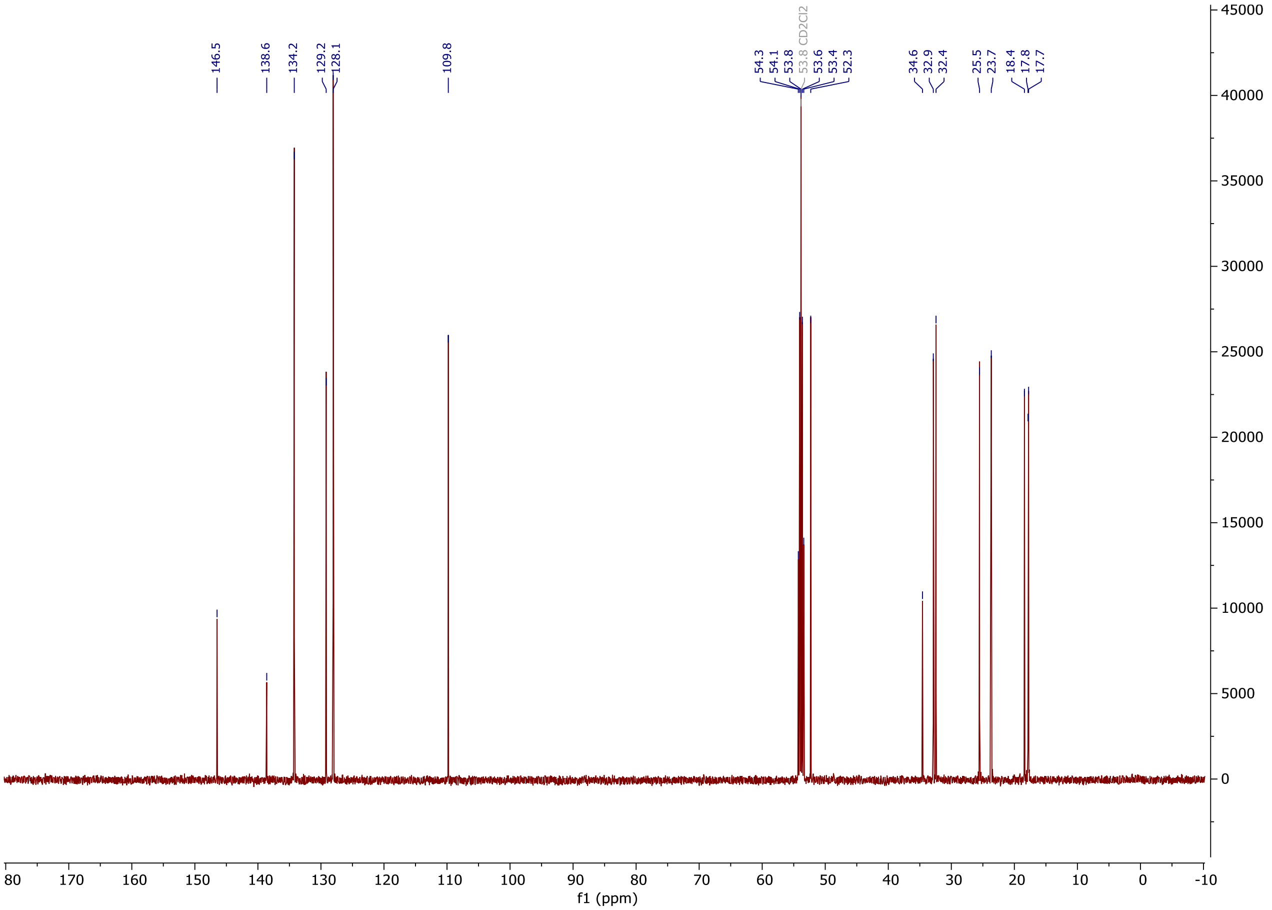

(*S*)-1-ethyl-3,3-dimethyl-5-methylene-1-phenylsilinane 2b

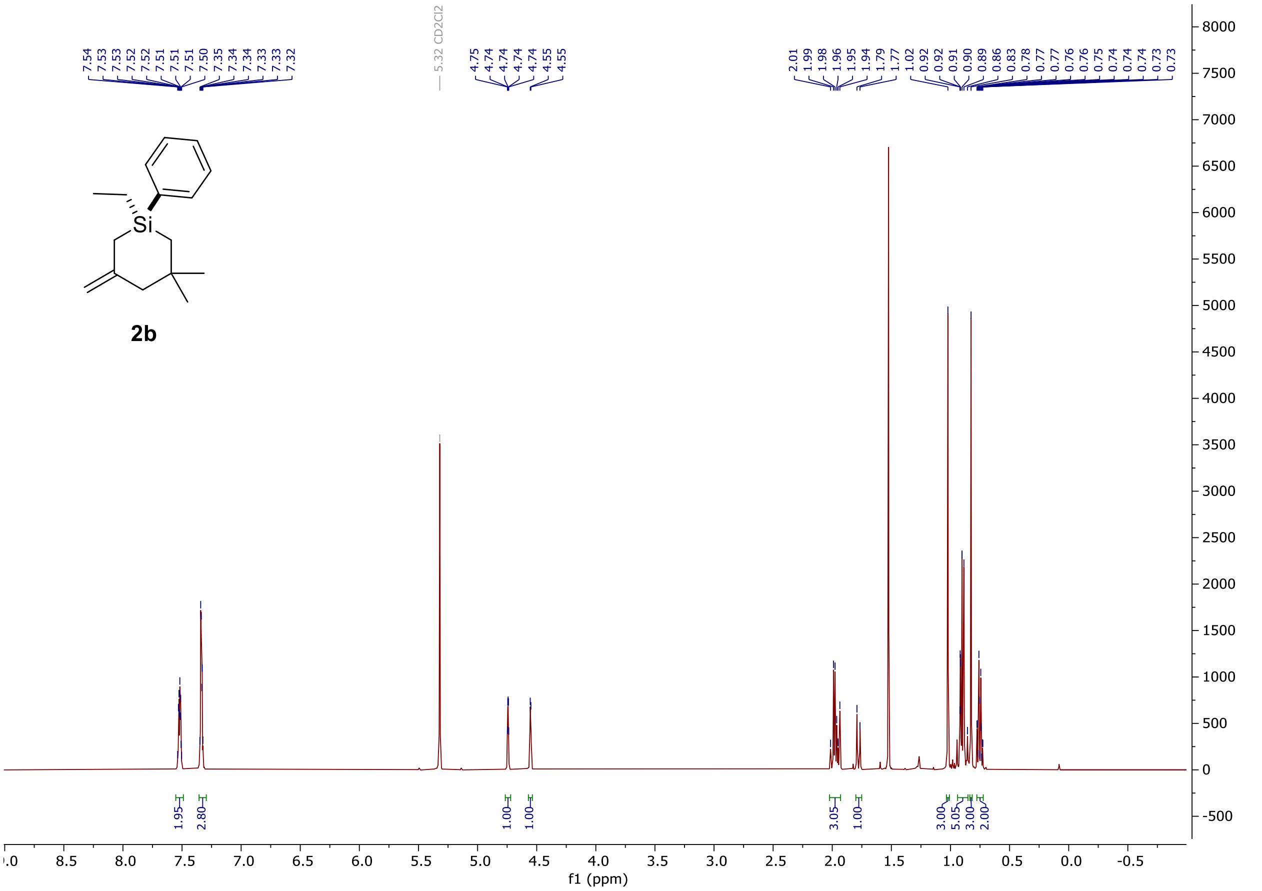

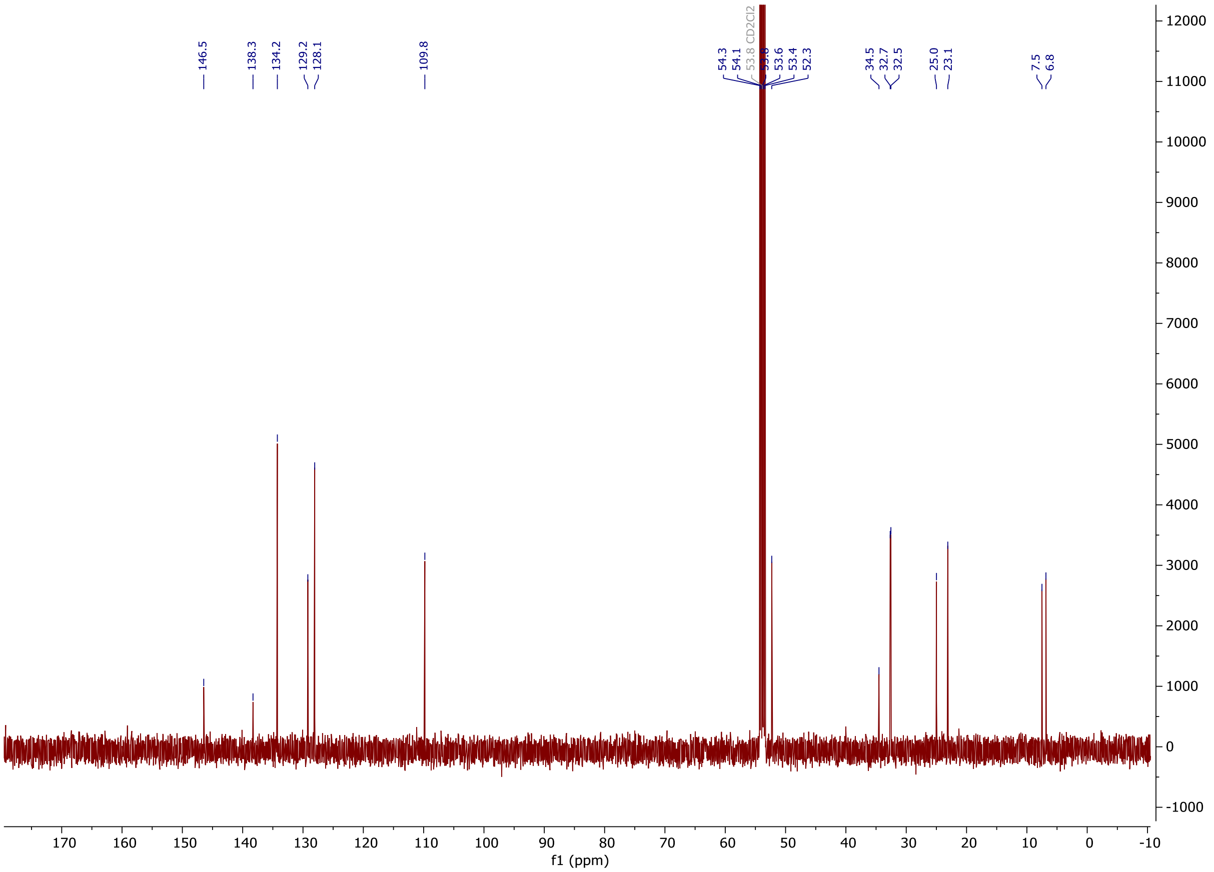

(*S*)-1-butyl-3,3-dimethyl-5-methylene-1-phenylsilinane 2c

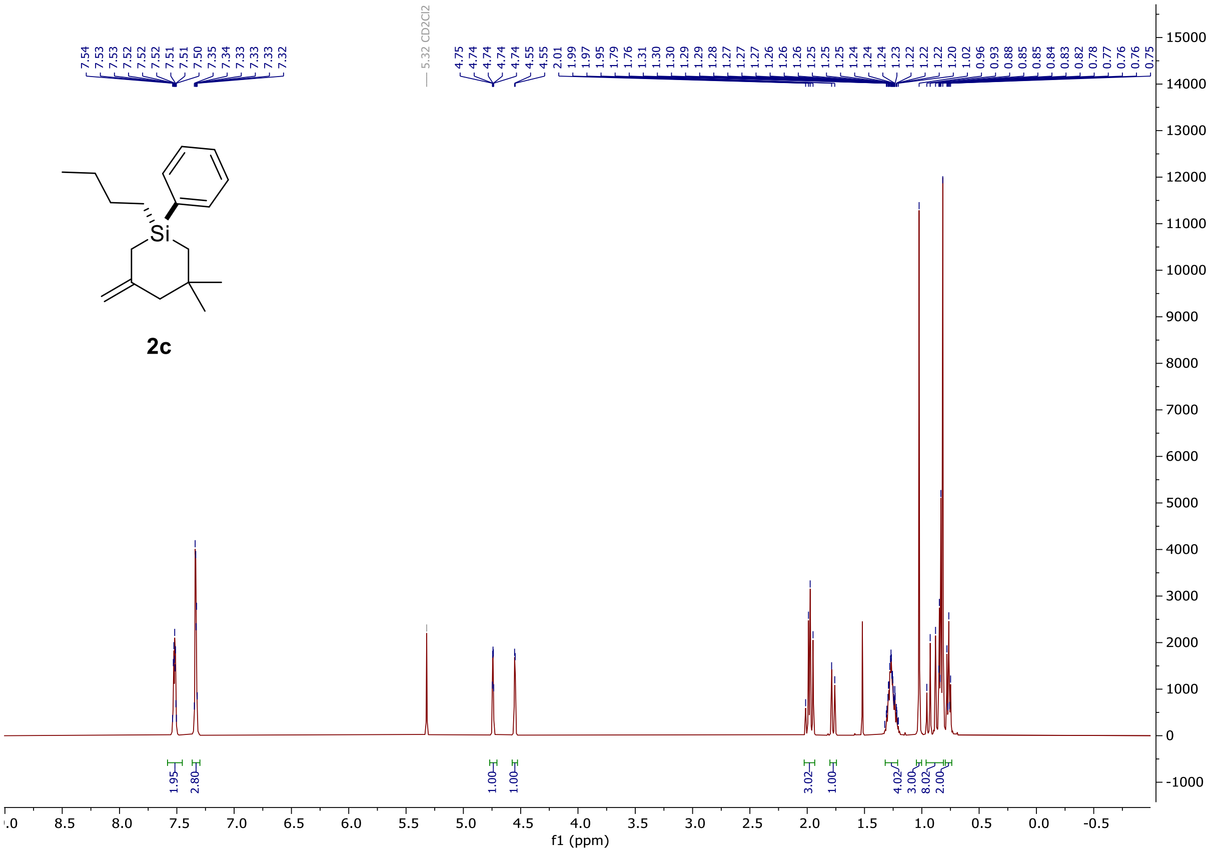

2c

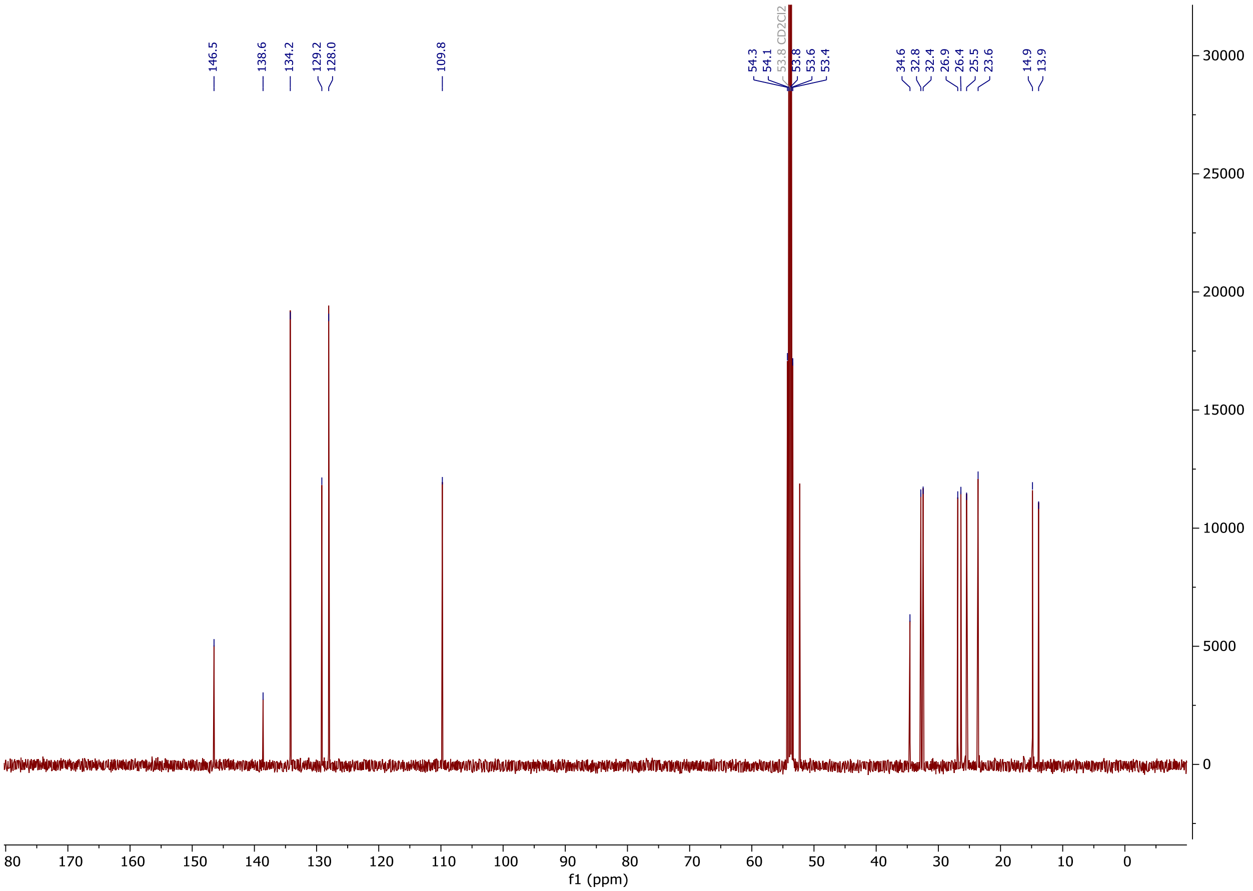

(*S*)-3,3-dimethyl-5-methylene-1-pentyl-1-phenylsilinane 2d

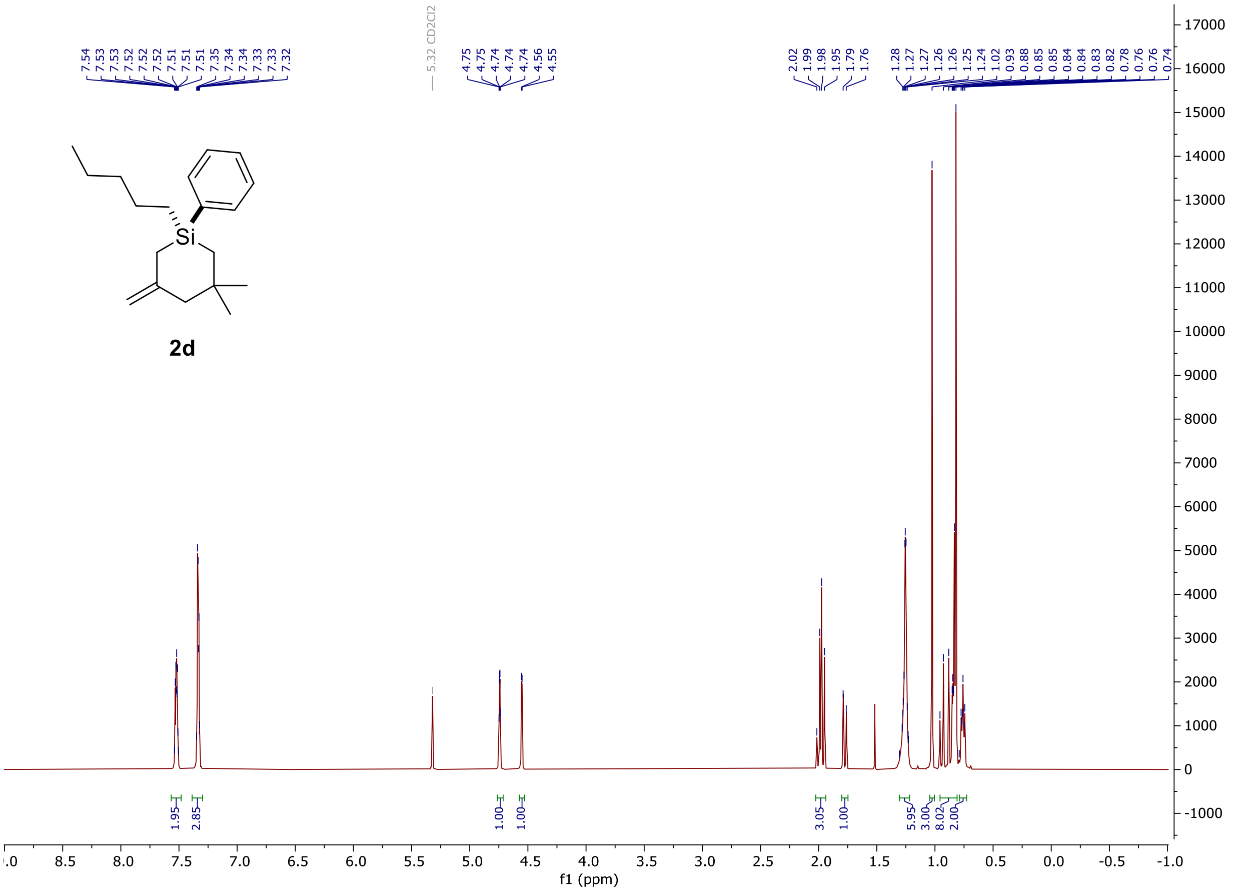

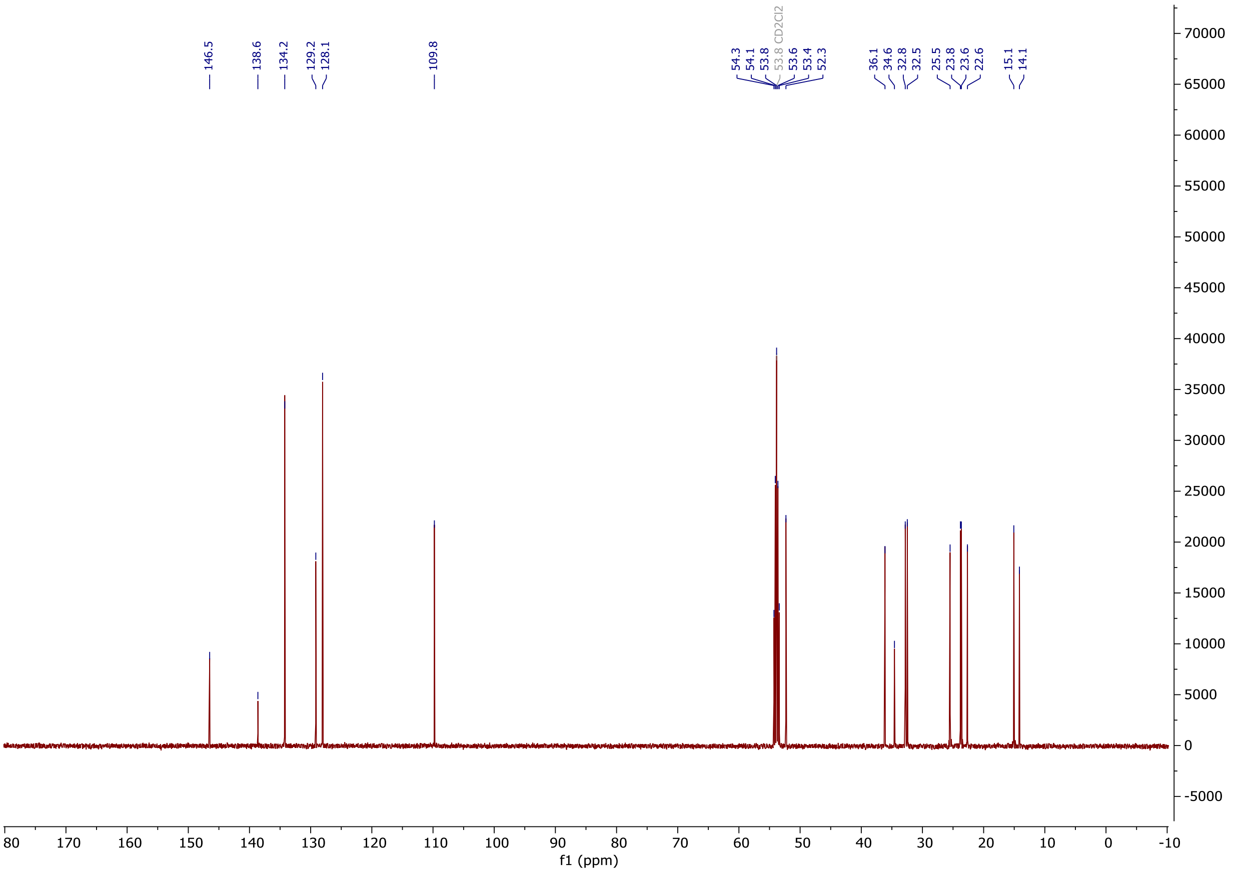

(*S*)-1-hexyl-3,3-dimethyl-5-methylene-1-phenylsilinane 2e

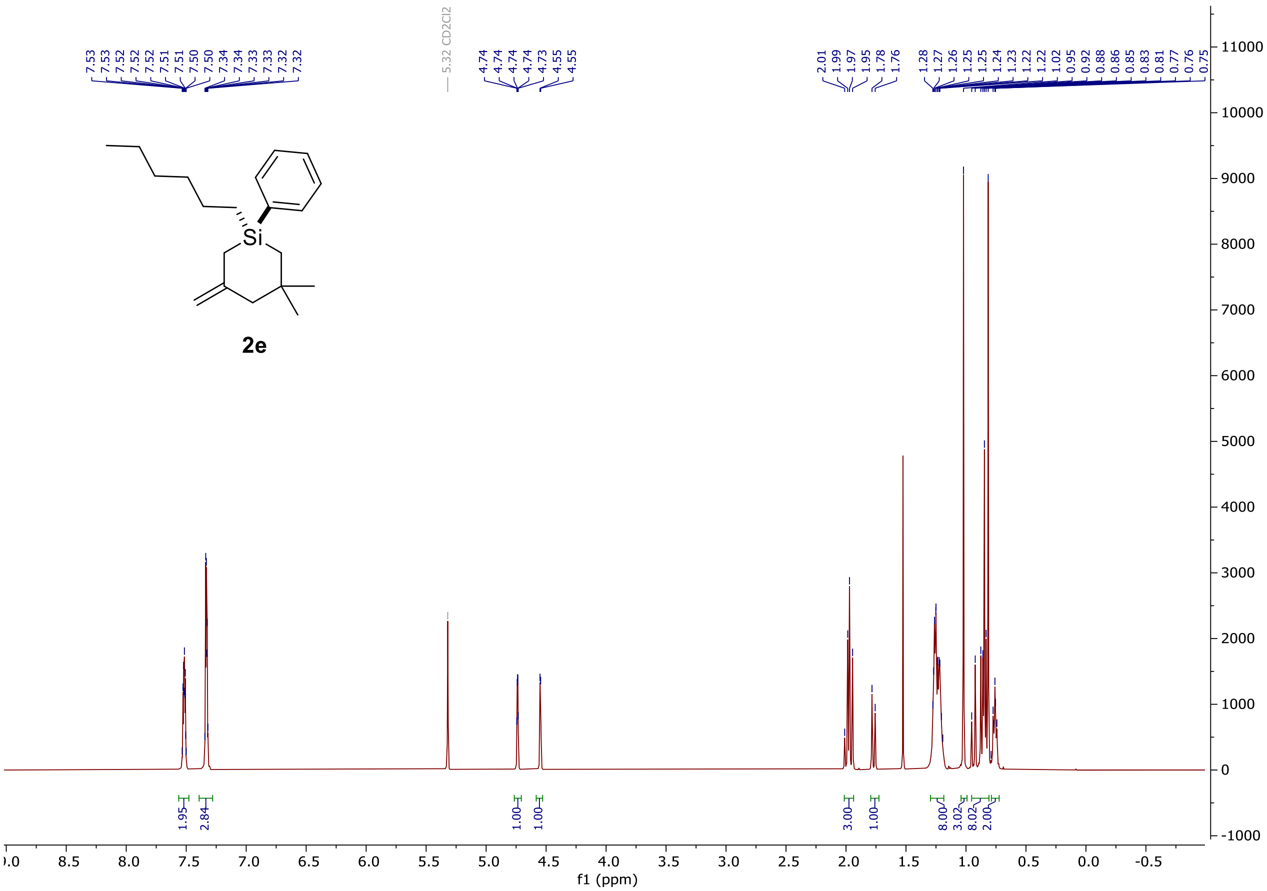

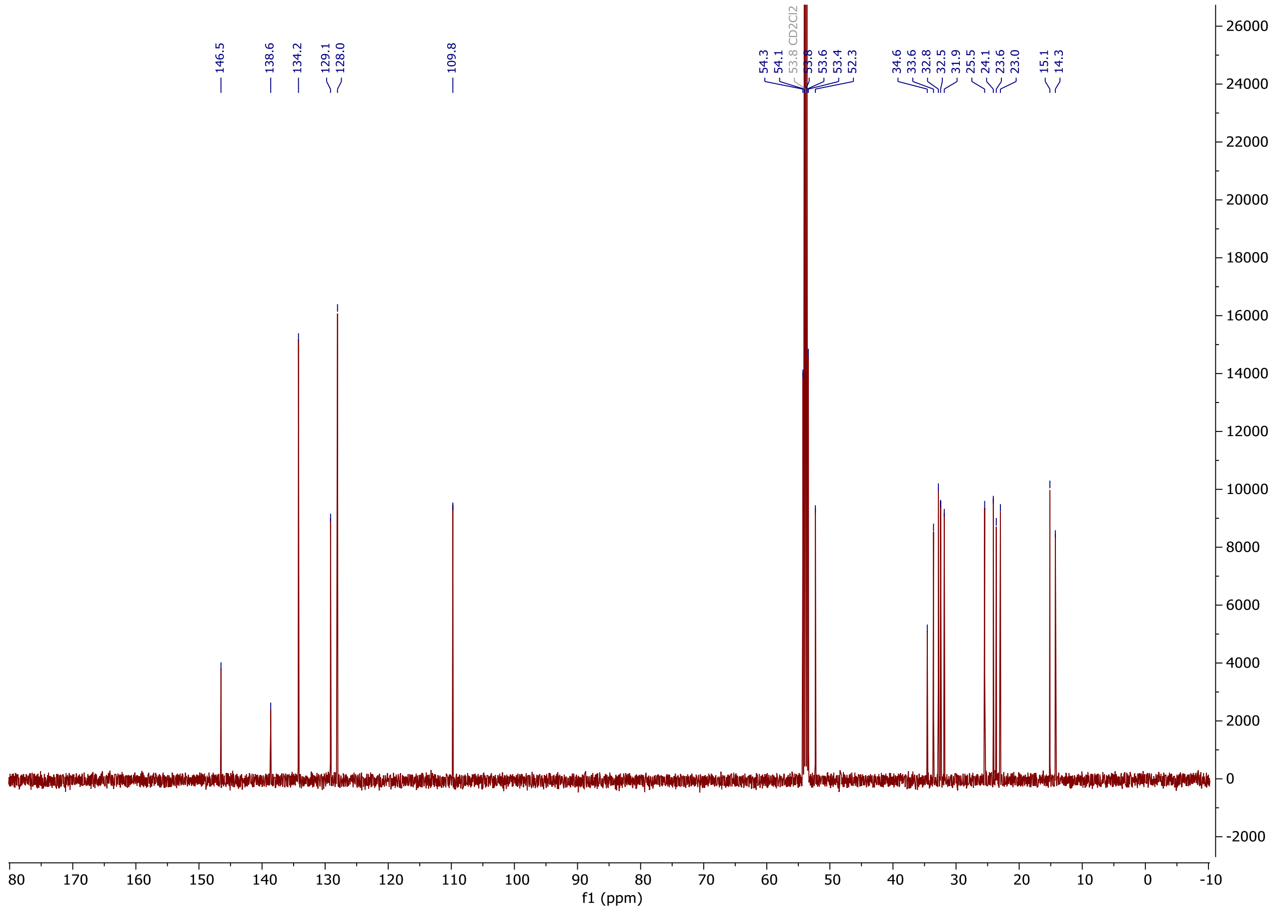

(*S*)-1-isopentyl-3,3-dimethyl-5-methylene-1-phenylsilinane 2f

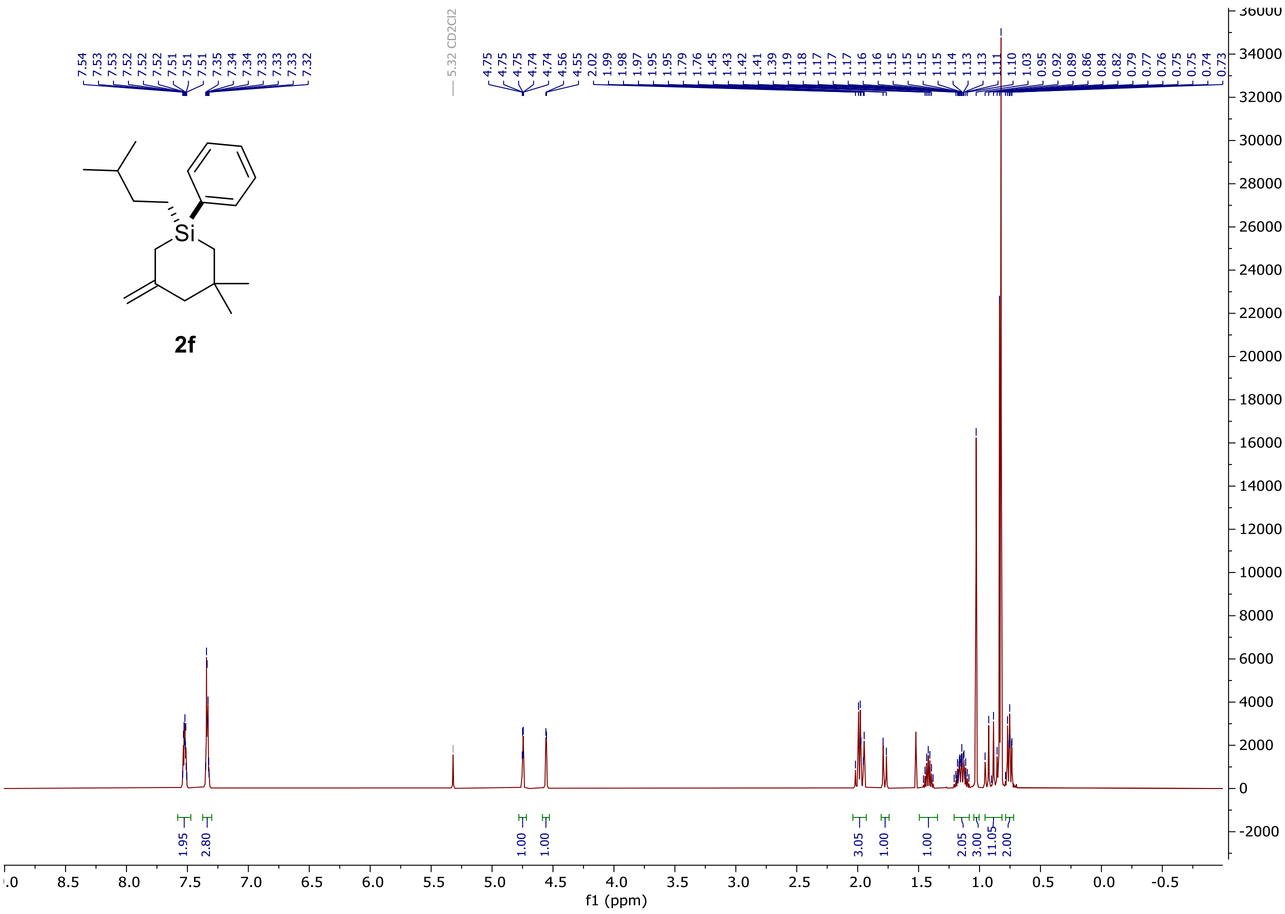

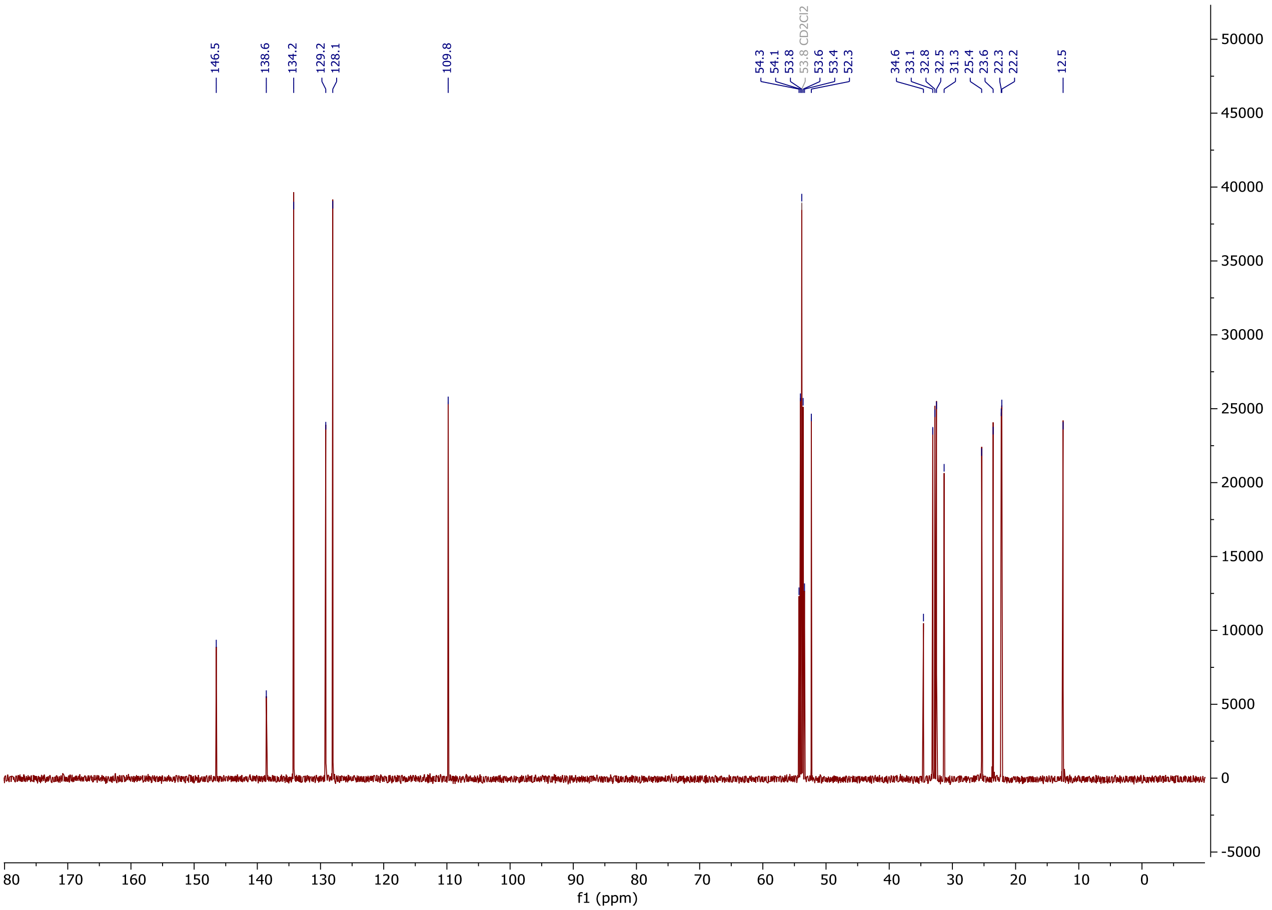

(*S*)-1-isobutyl-3,3-dimethyl-5-methylene-1-phenylsilinane 2g

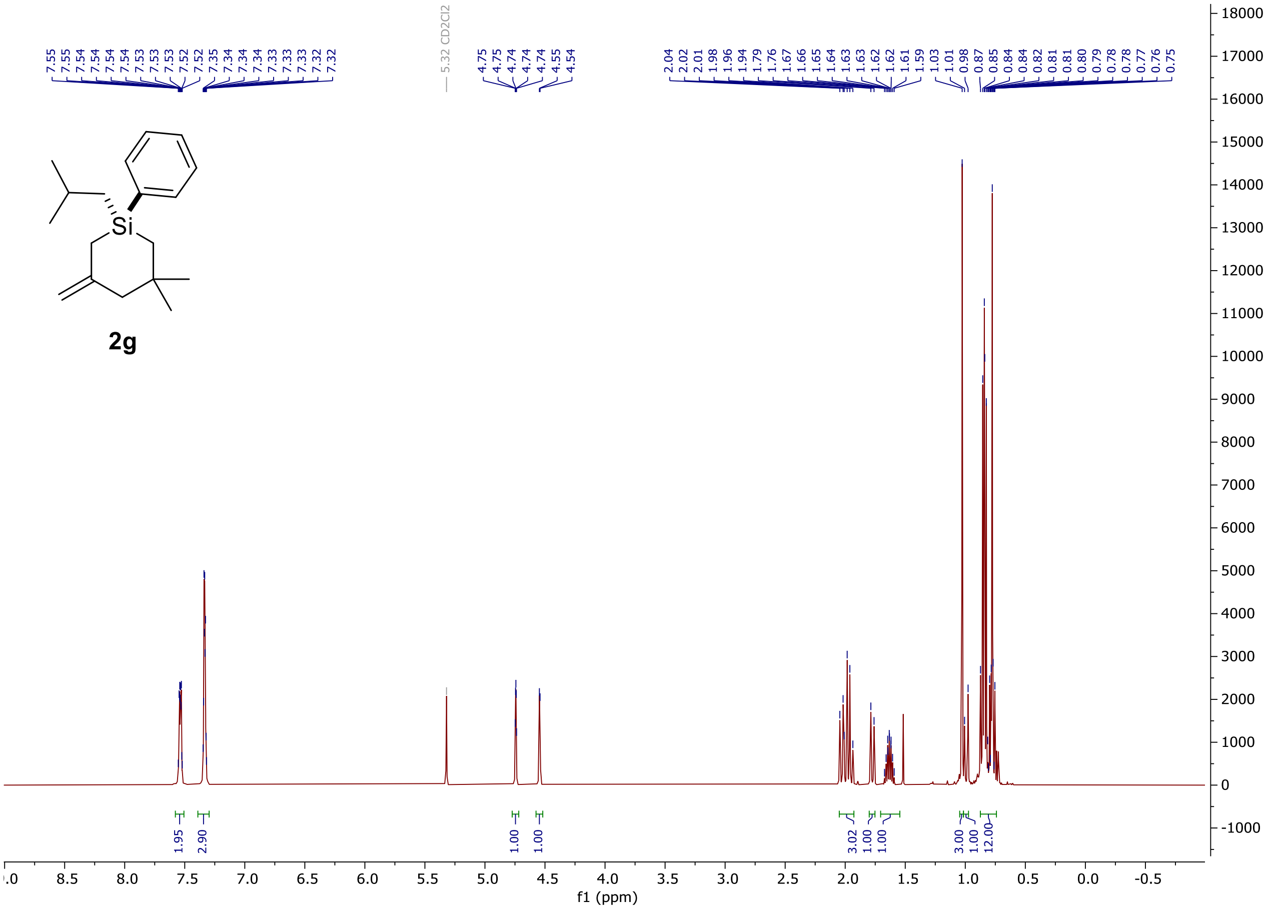

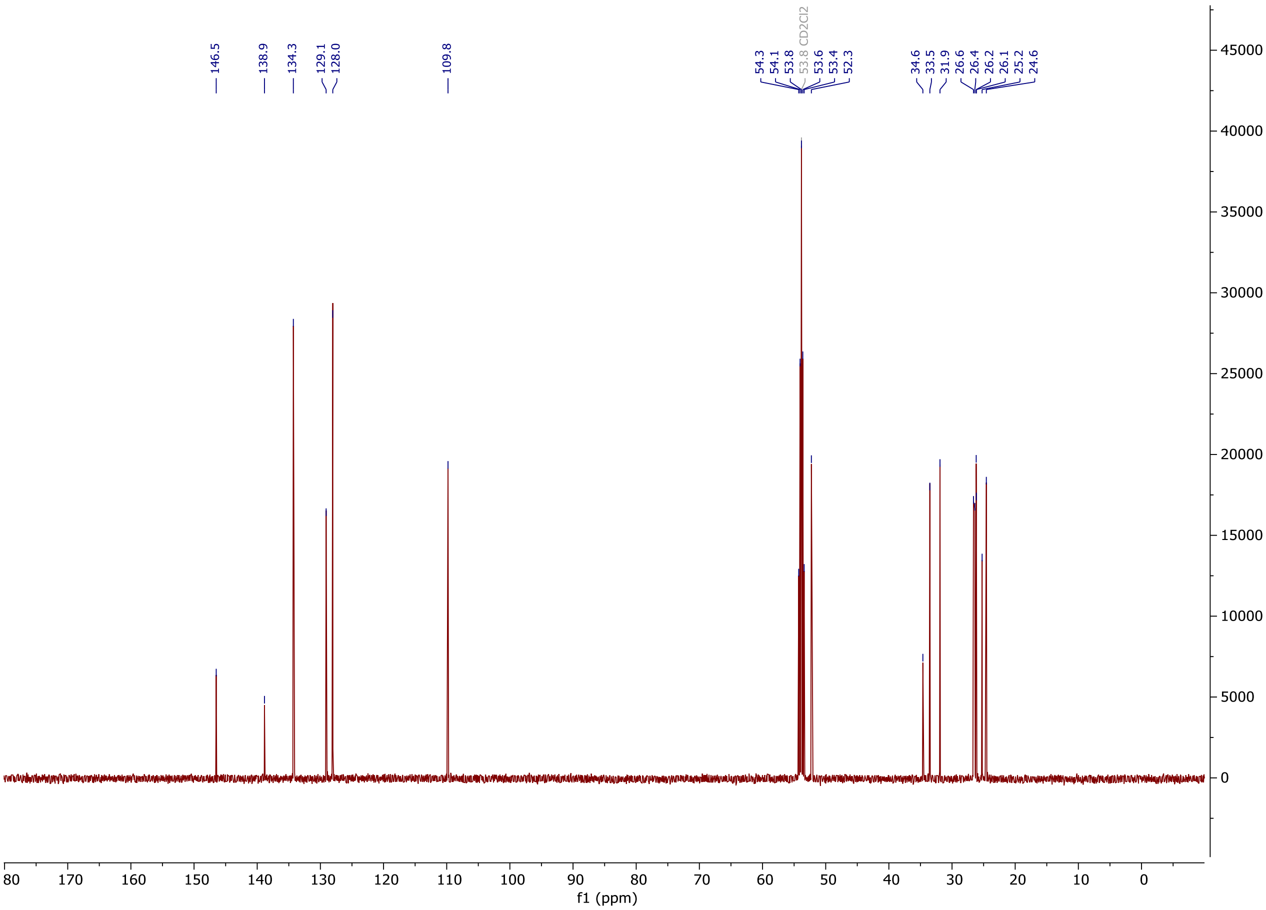

(*S*)-3,3-dimethyl-5-methylene-1-(naphthalen-2-yl)-1-propylsilinane 2h

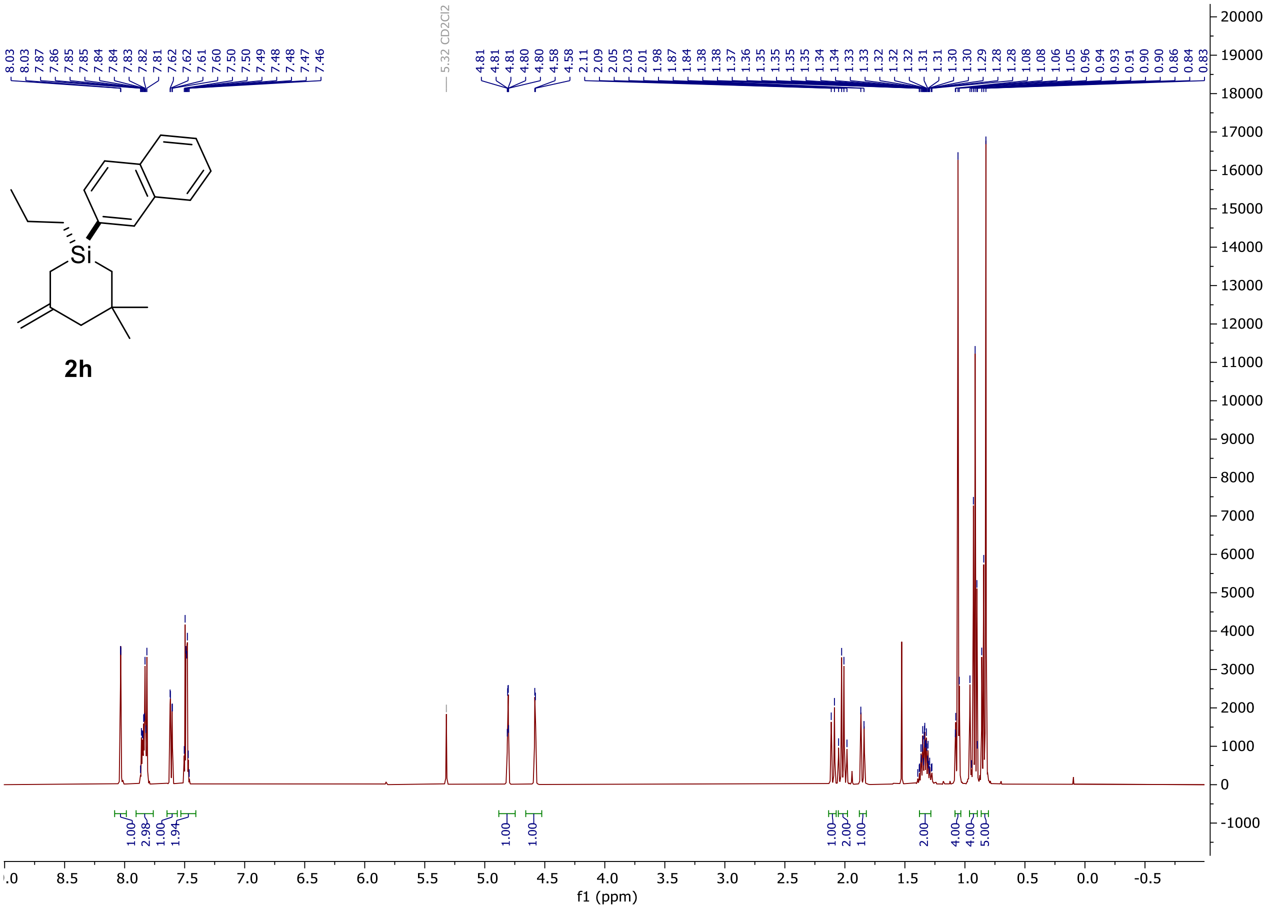

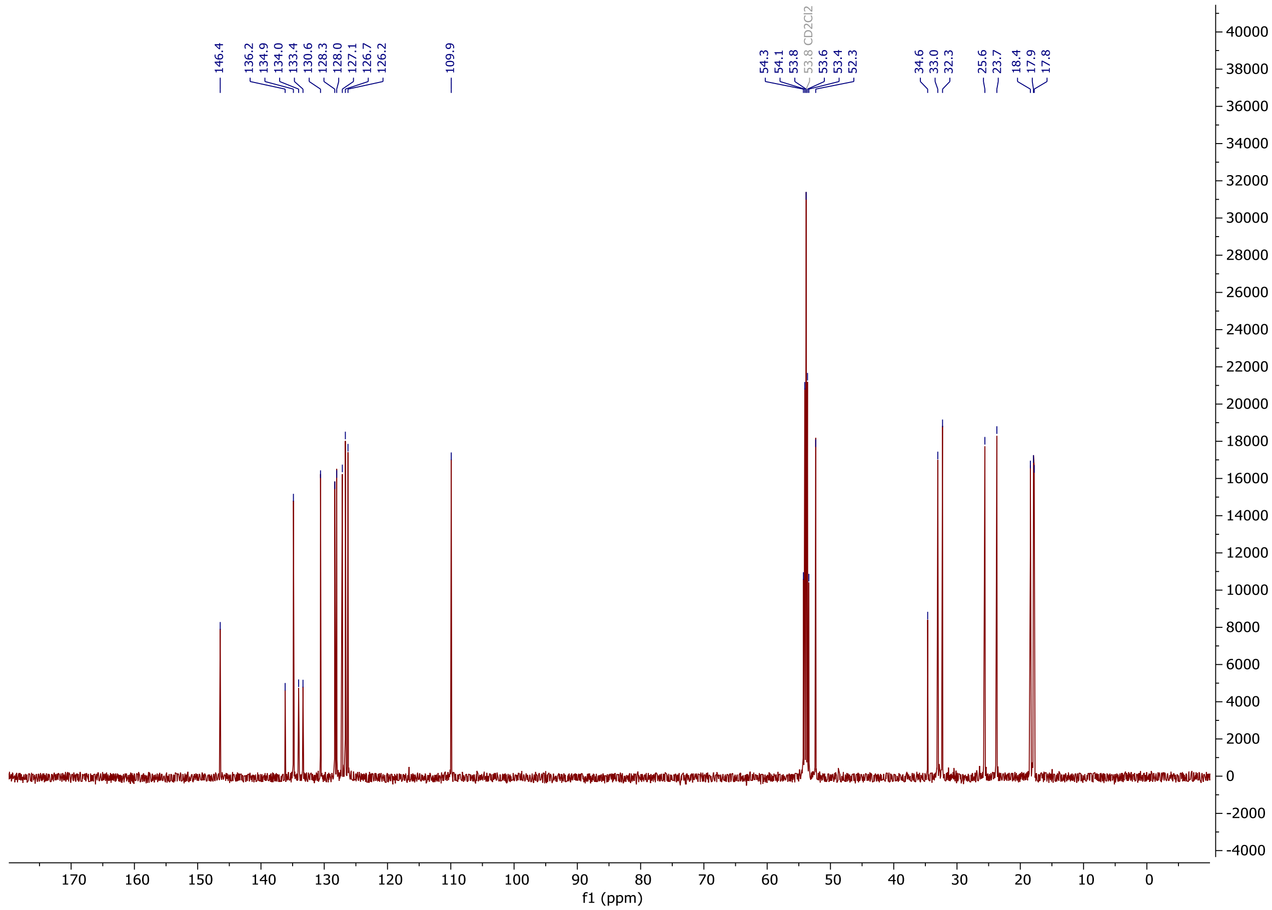

(*S*)-3,3-dimethyl-5-methylene-1-propyl-1-(*o*-tolyl)silinane 2i

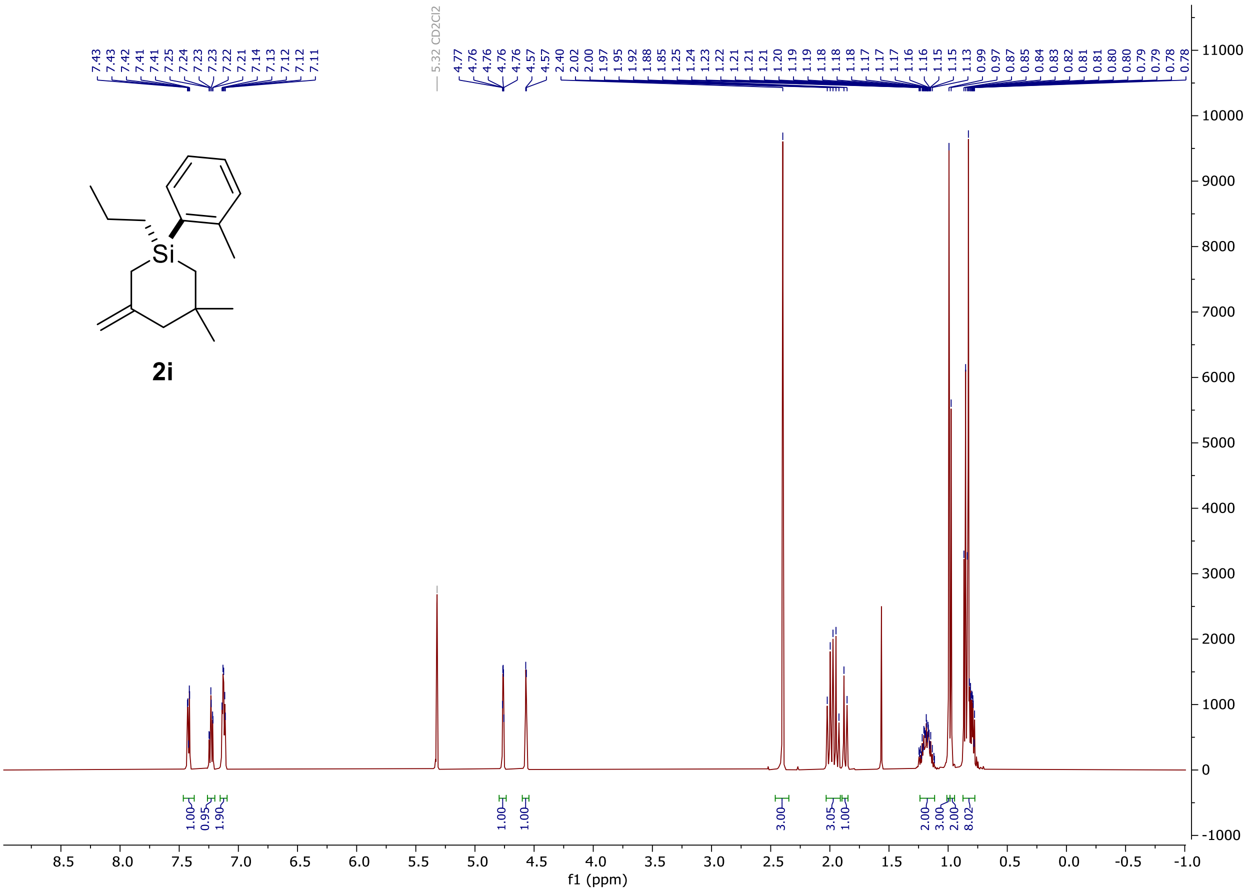

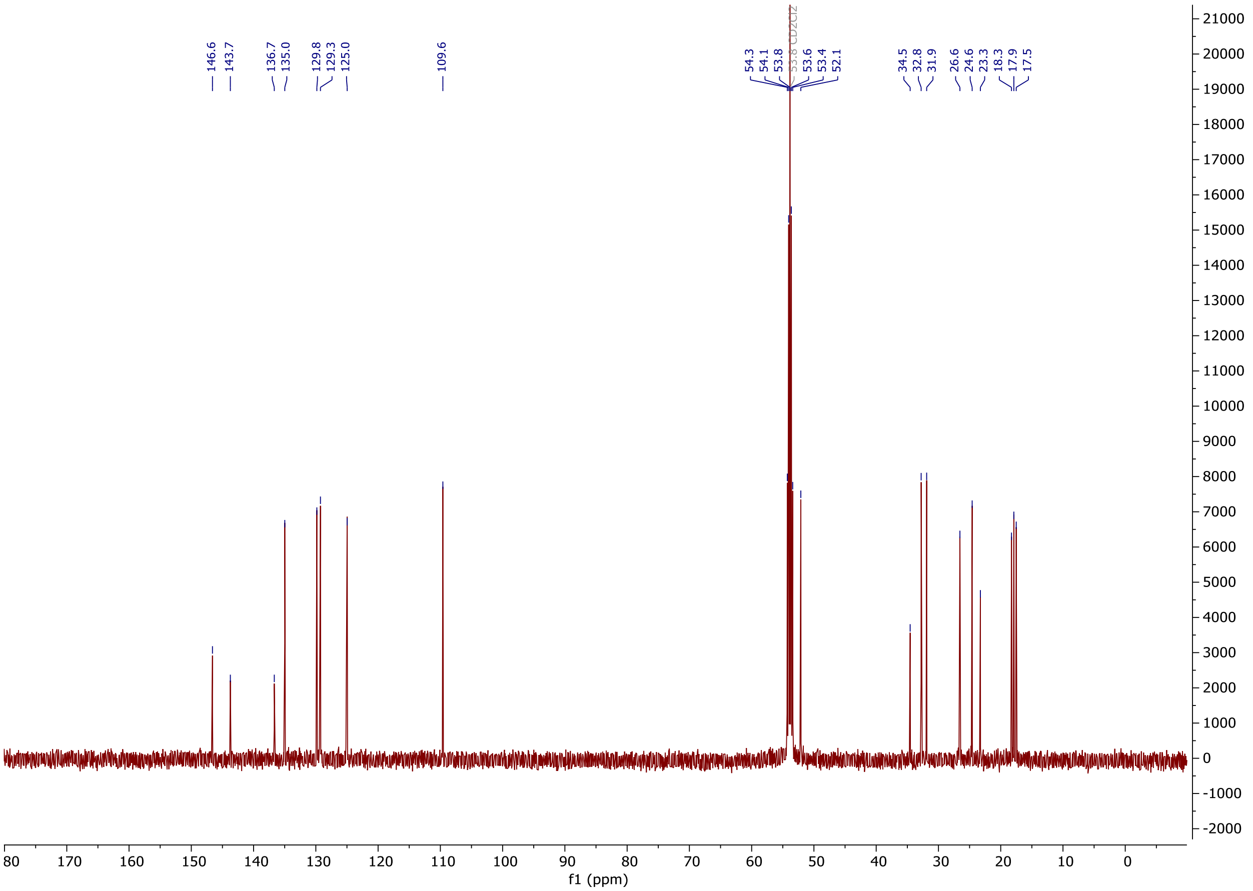

(*S*)-3,3-dimethyl-5-methylene-1-propyl-1-(*m*-tolyl)silinane 2j

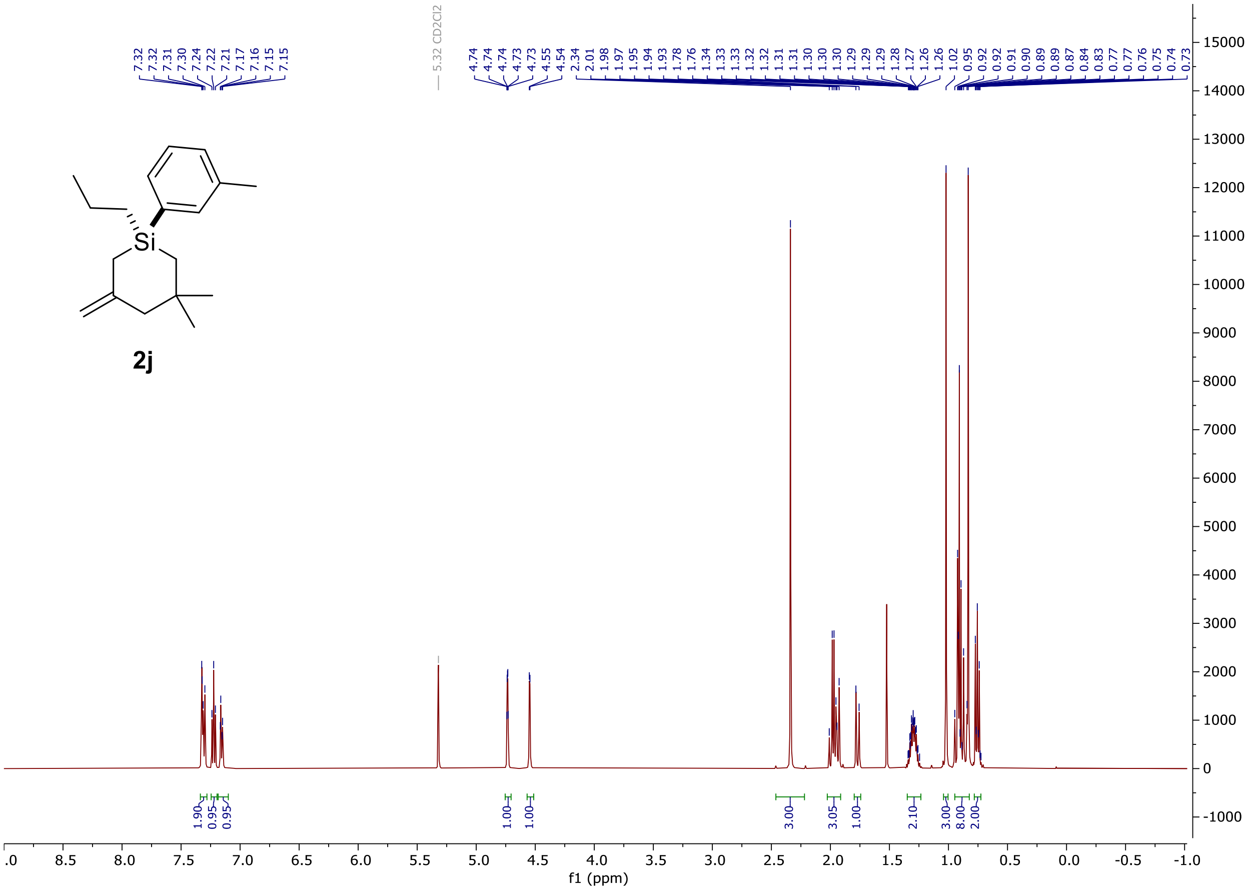

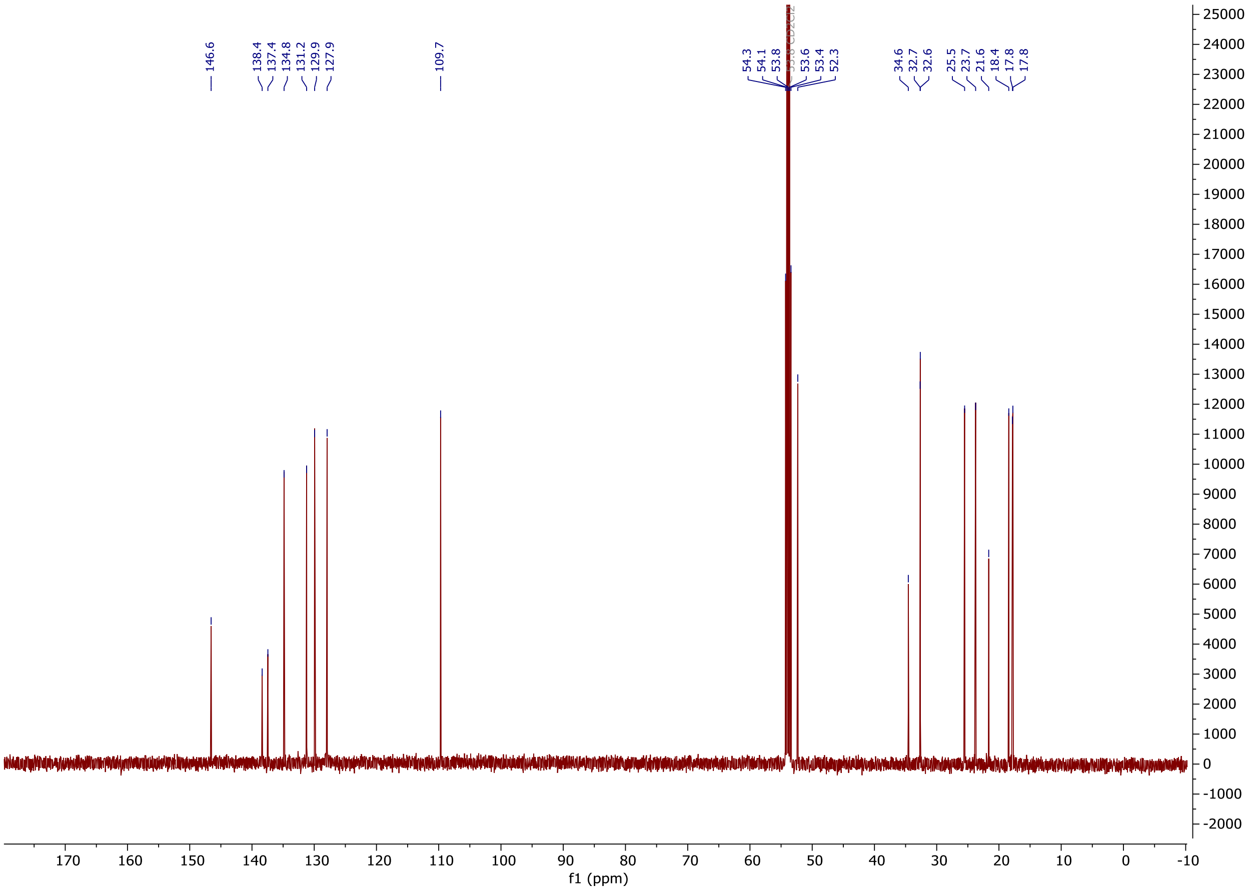

(*S*)-3,3-dimethyl-5-methylene-1-propyl-1-(*p*-tolyl)silinane 2k

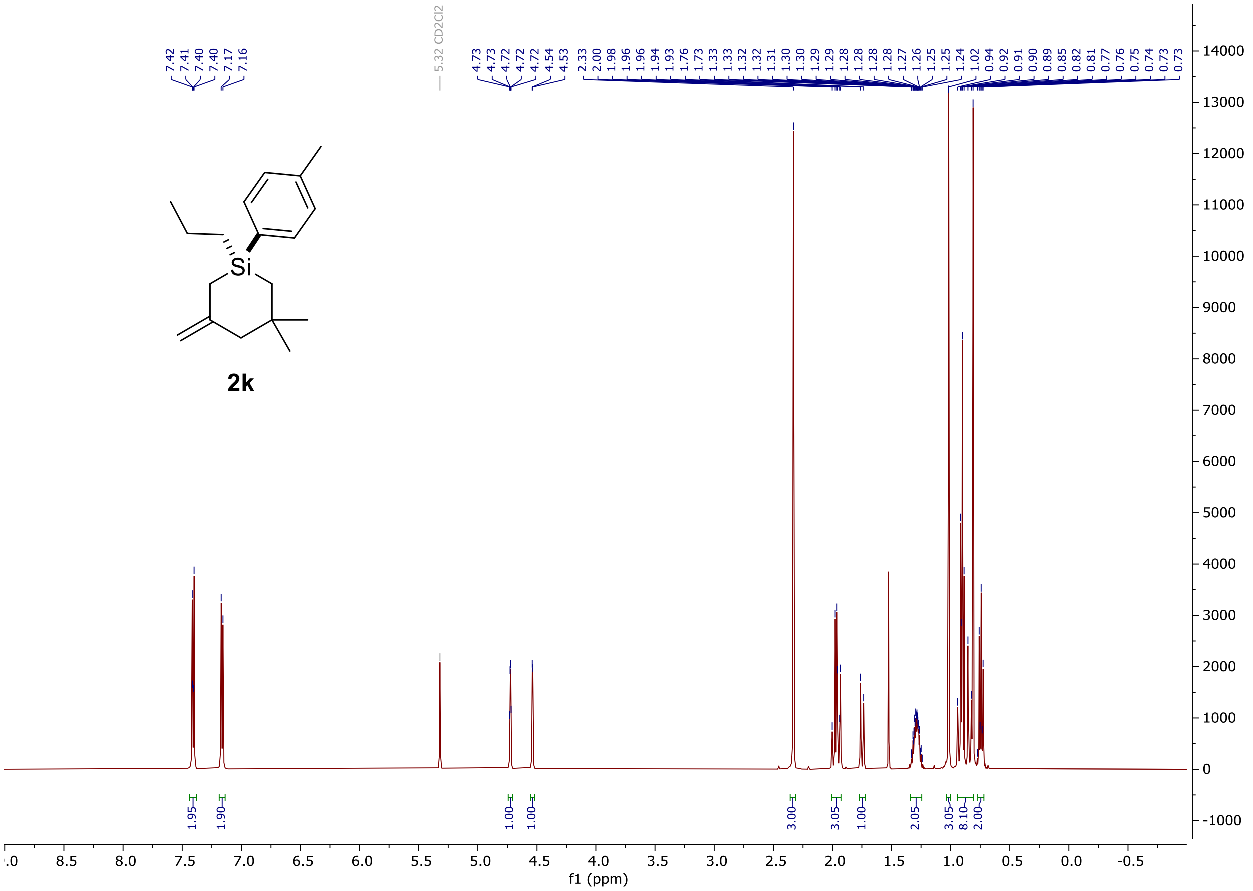

2k

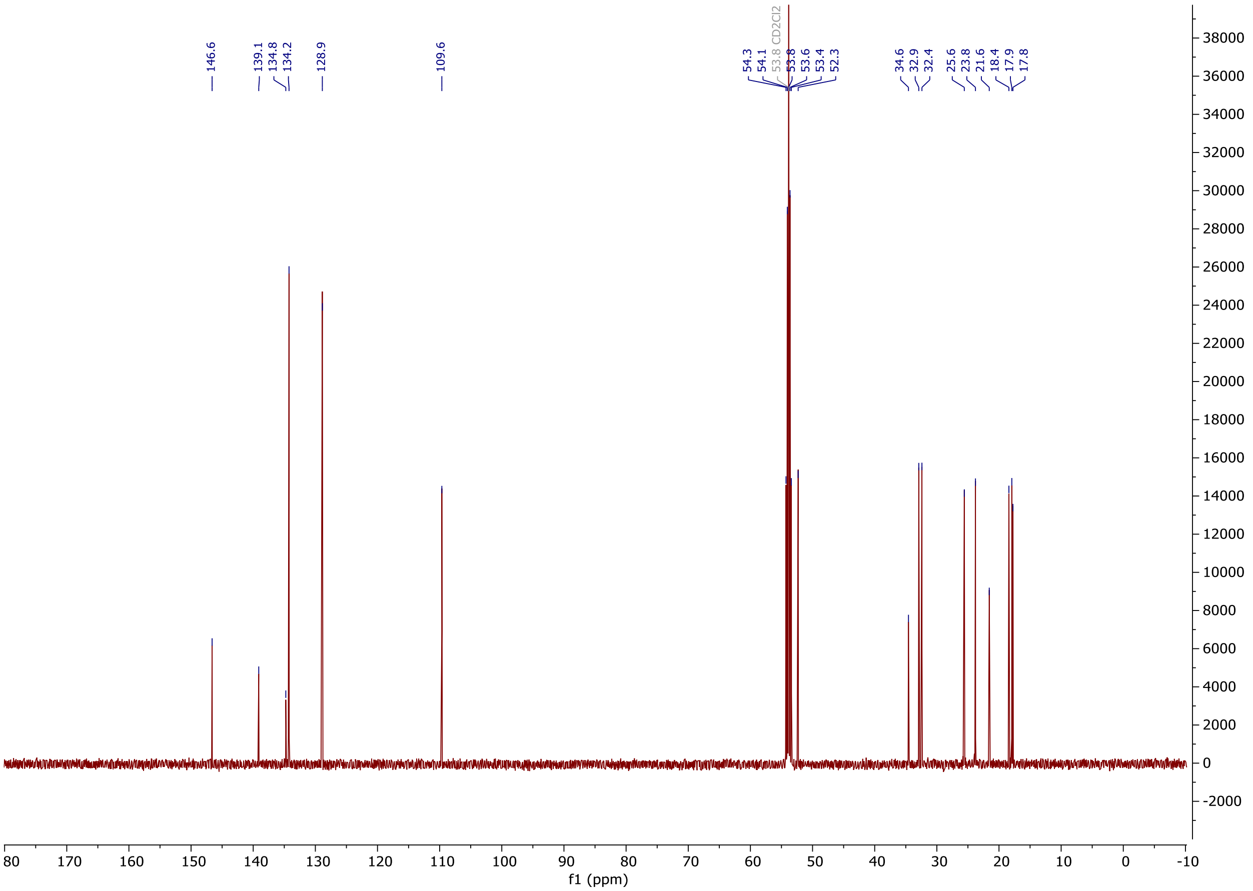

(*S*)-1-(4-methoxyphenyl)-3,3-dimethyl-5-methylene-1-propylsilinane 2l

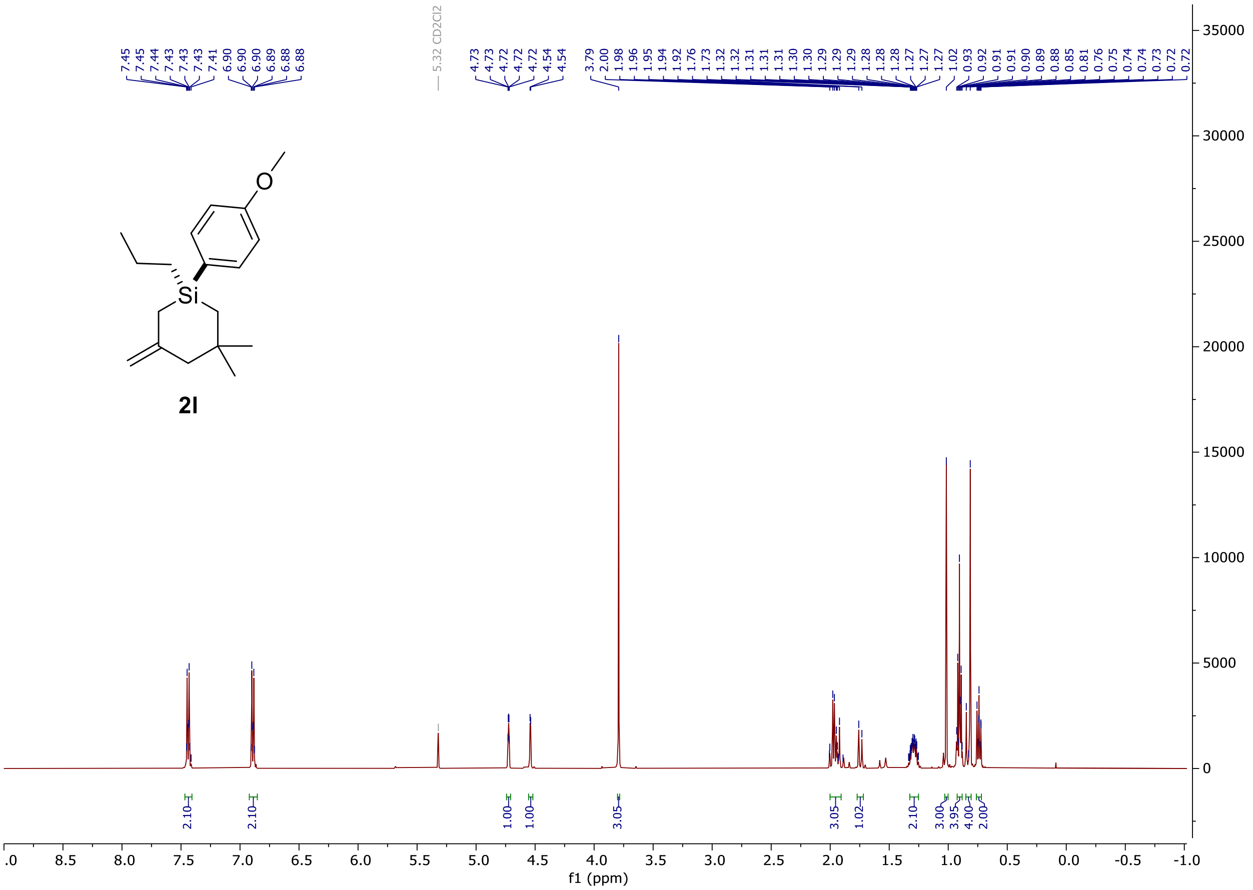

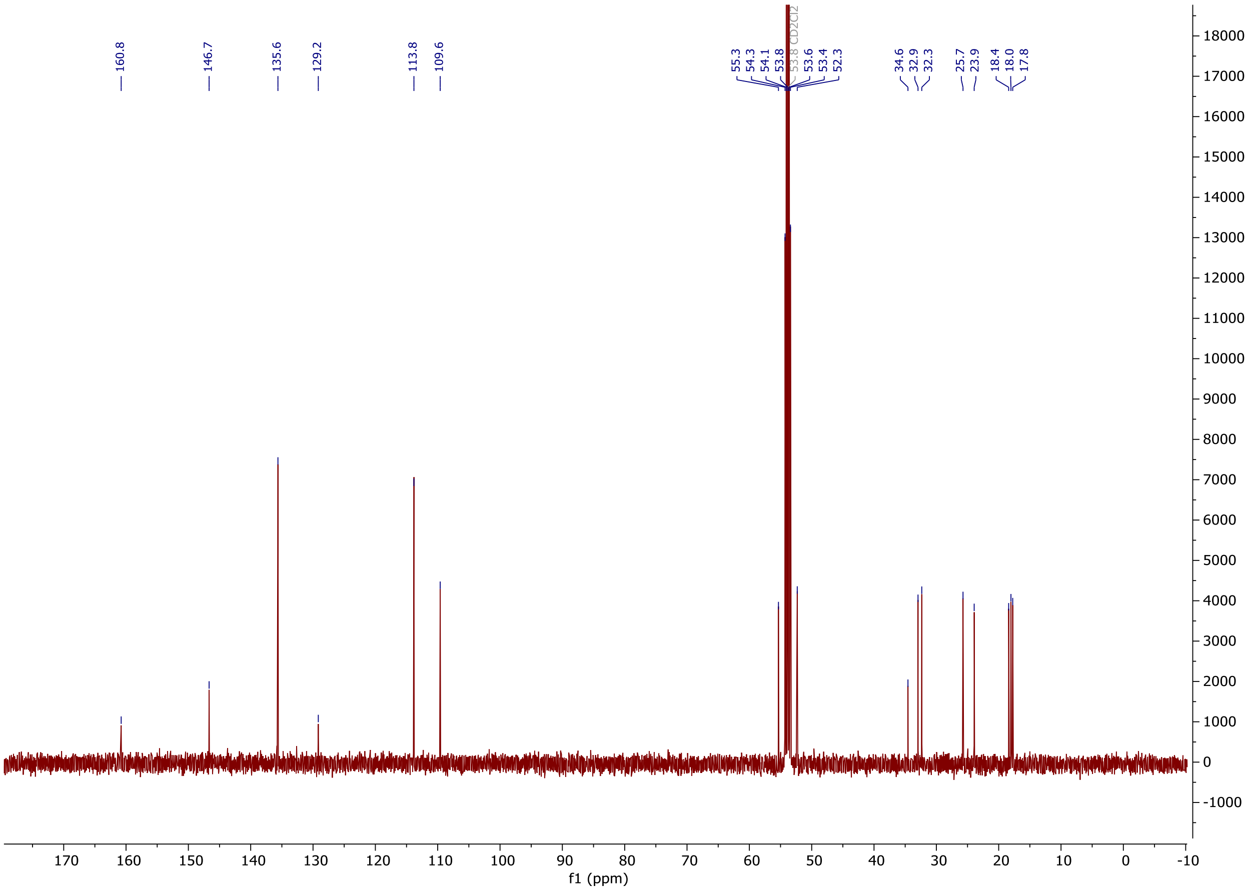

(*S*)-1-(4-fluorophenyl)-3,3-dimethyl-5-methylene-1-propylsilinane 2m

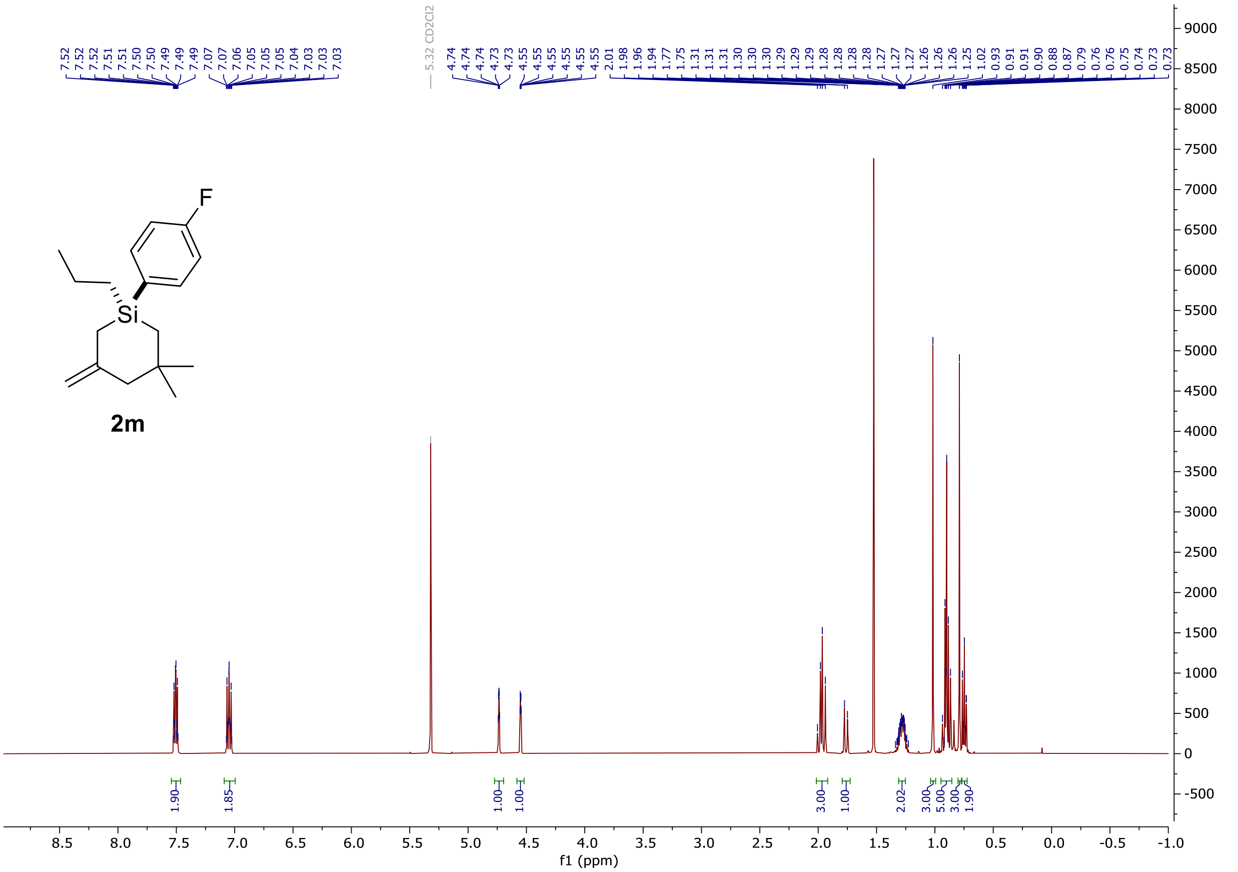

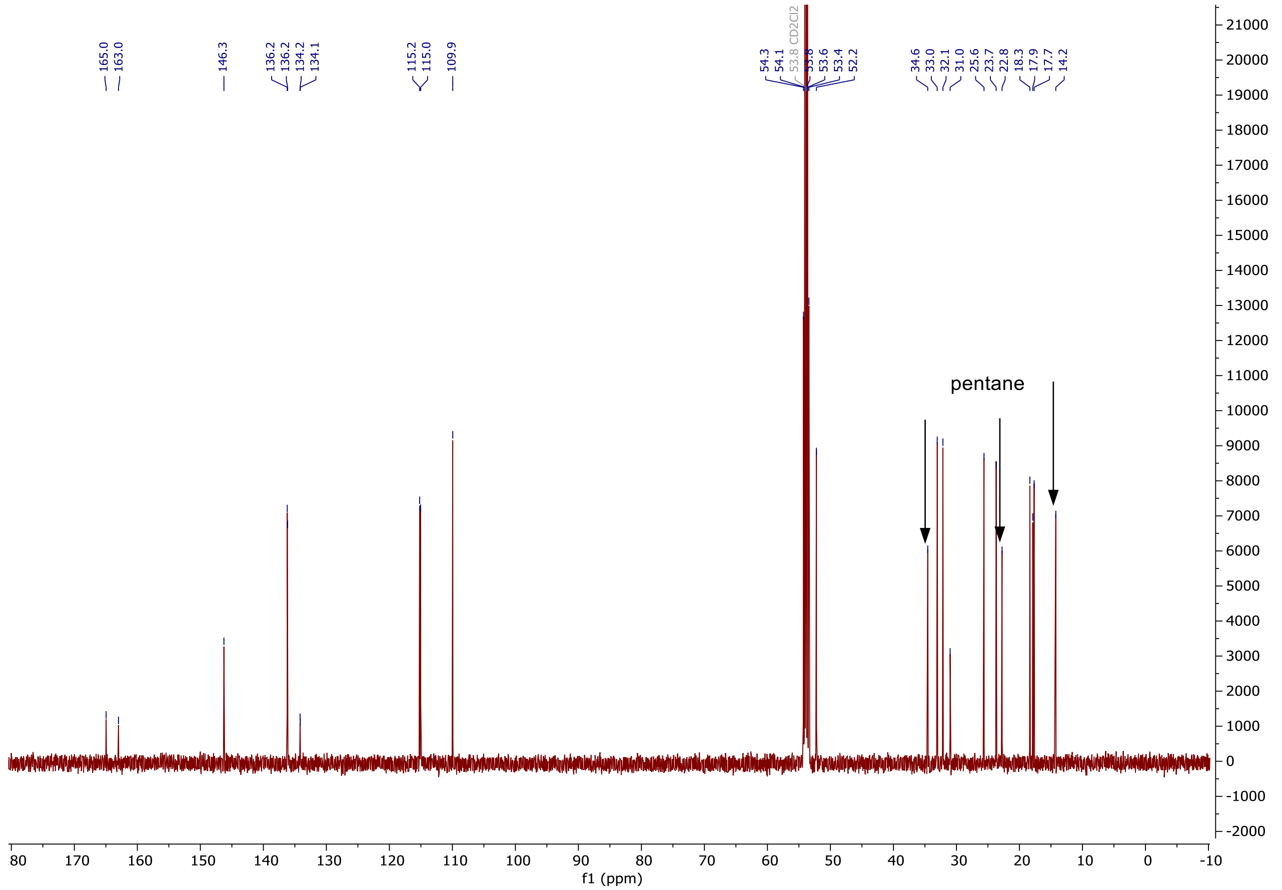

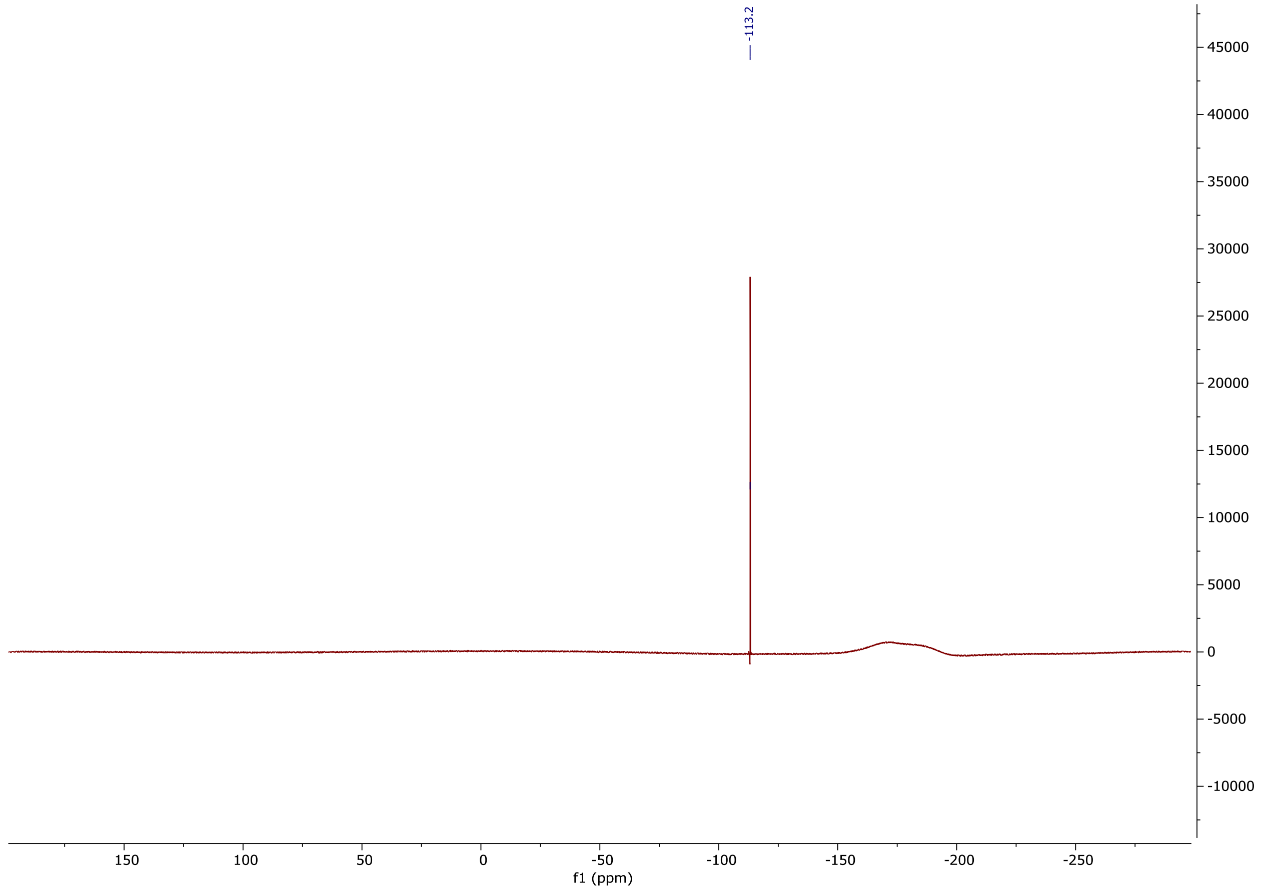

(*S*)-1-allyl-3,3-dimethyl-5-methylene-1-phenylsilinane 2n

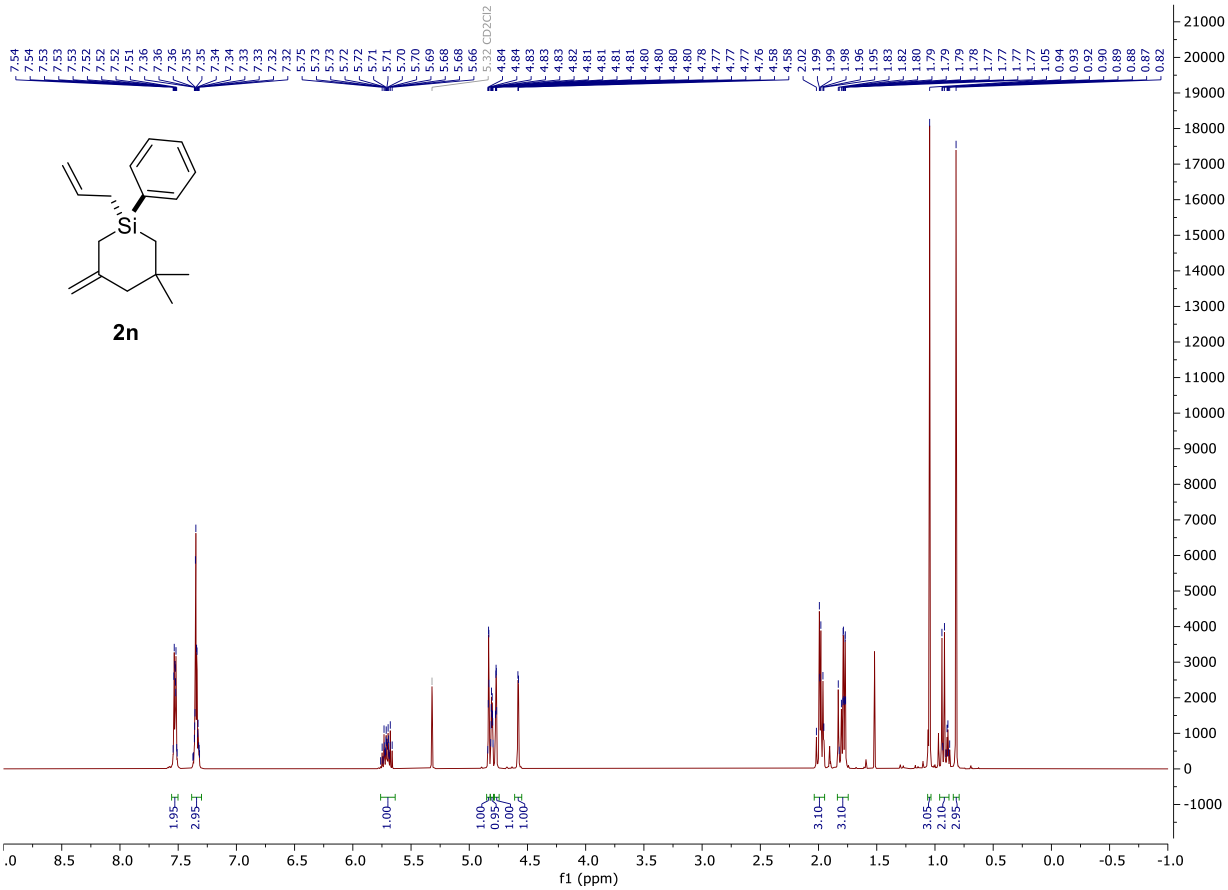

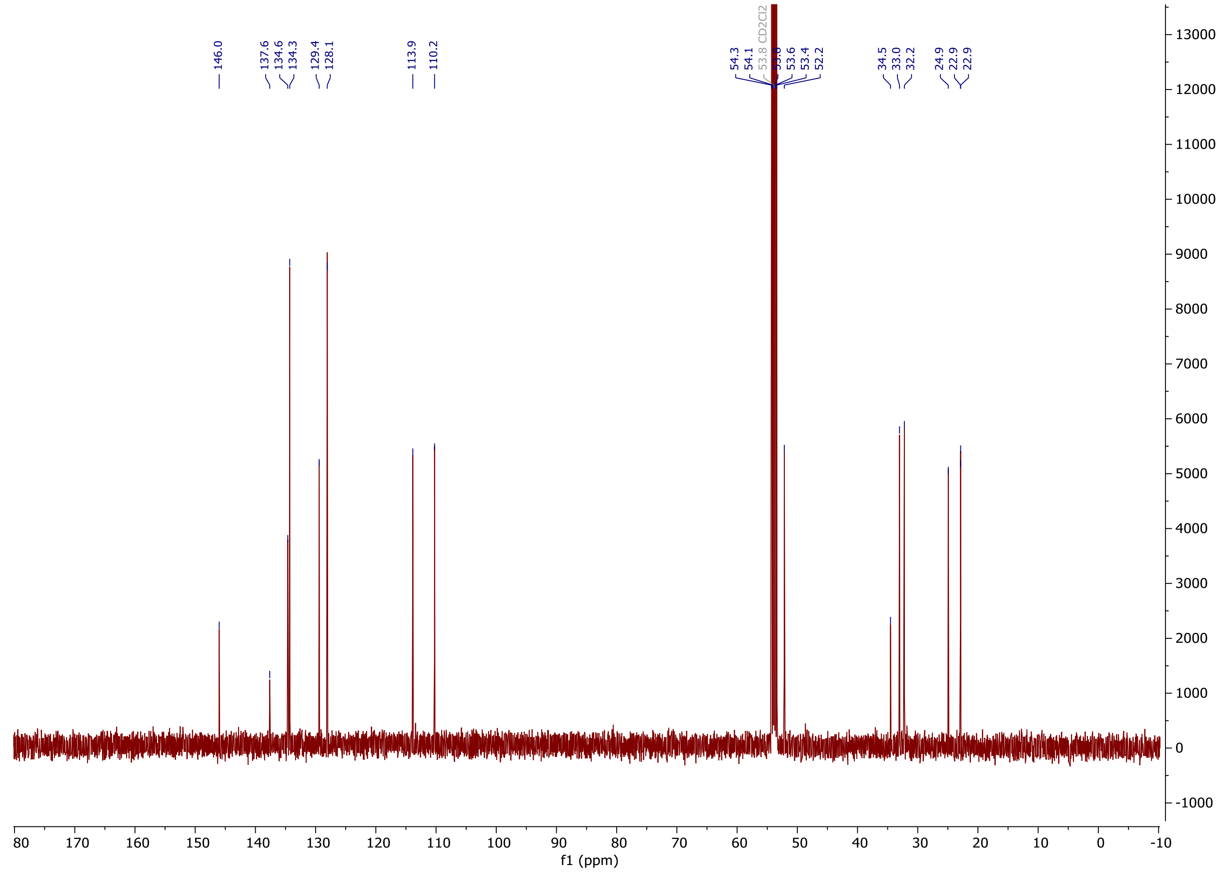

(*S*)-3,3-dimethyl-1-(2-methylallyl)-5-methylene-1-phenylsilinane 2o

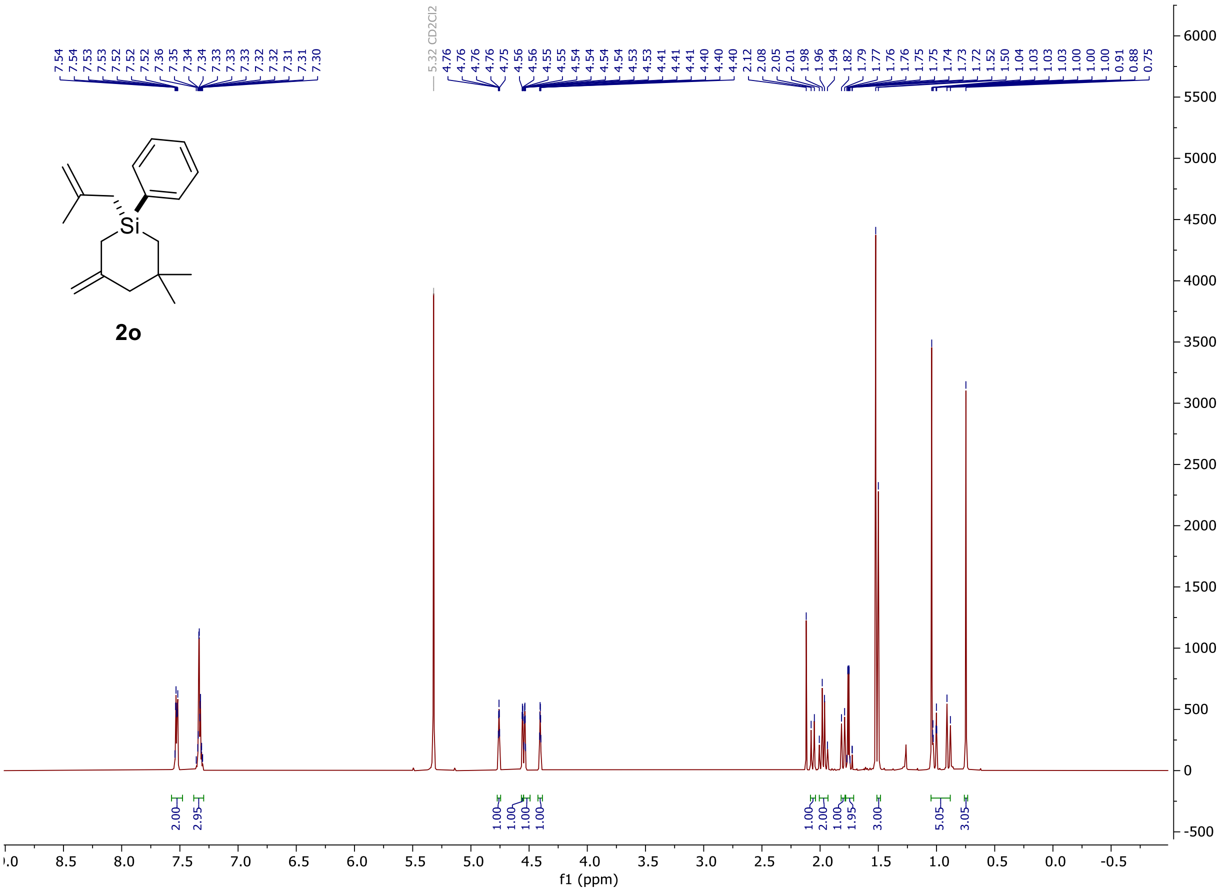

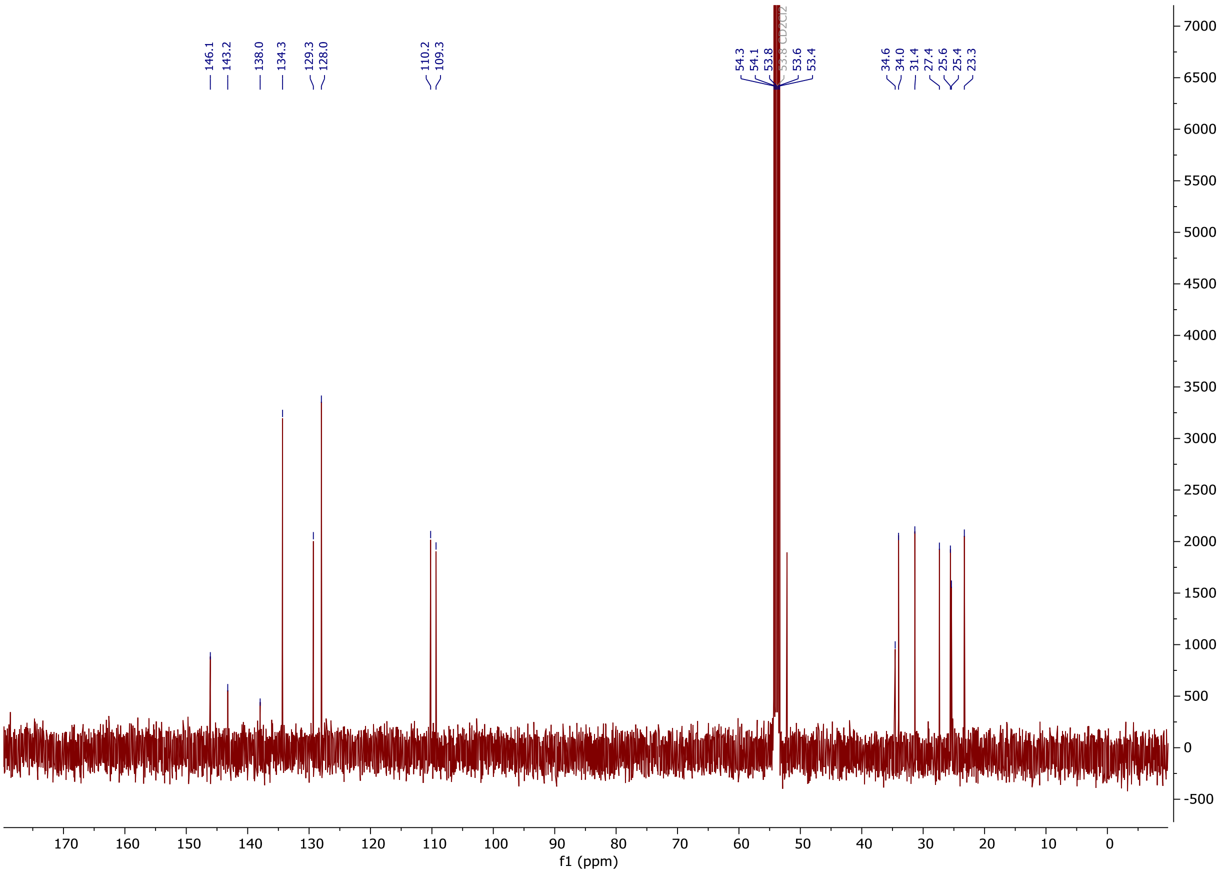

(*S*)-1-cyclohexyl-3,3-dimethyl-5-methylene-1-propylsilinane 2o

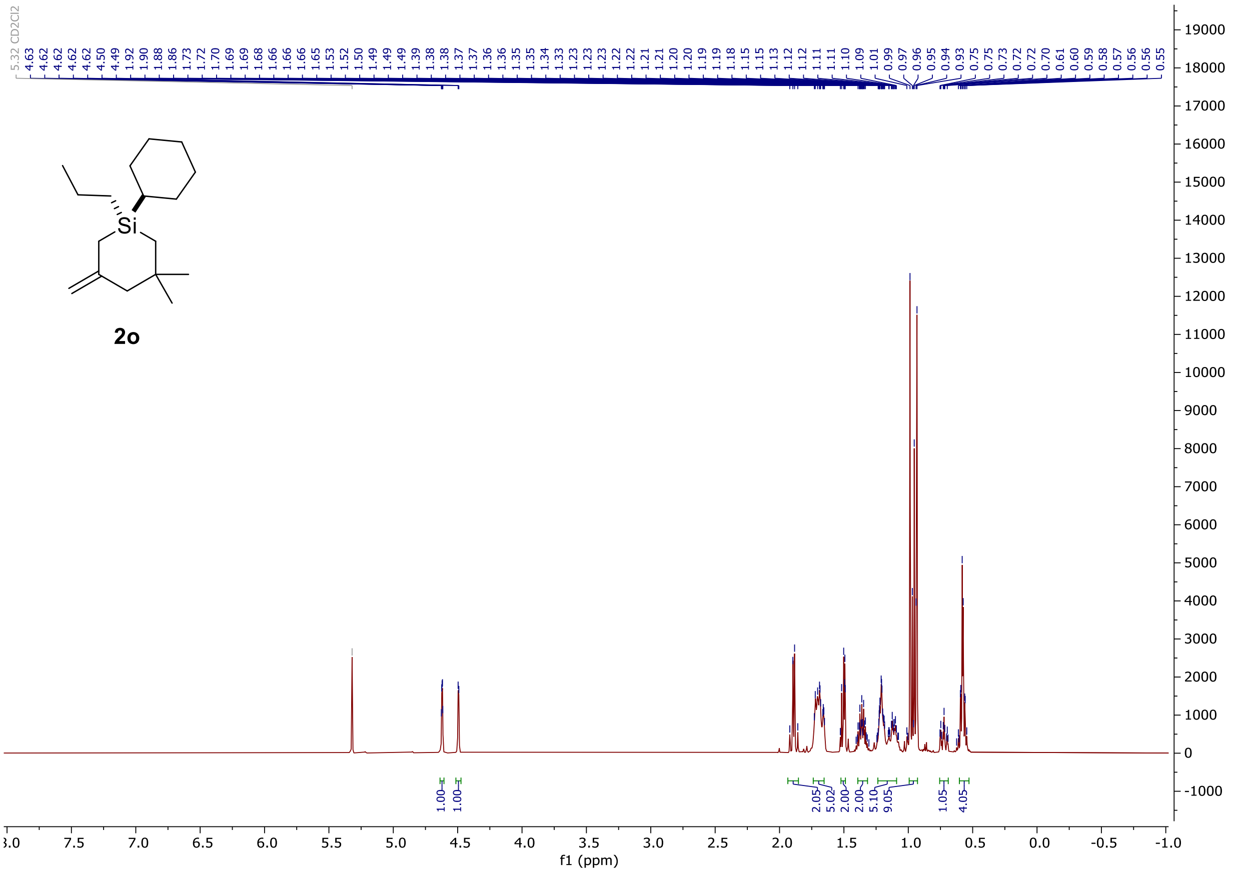

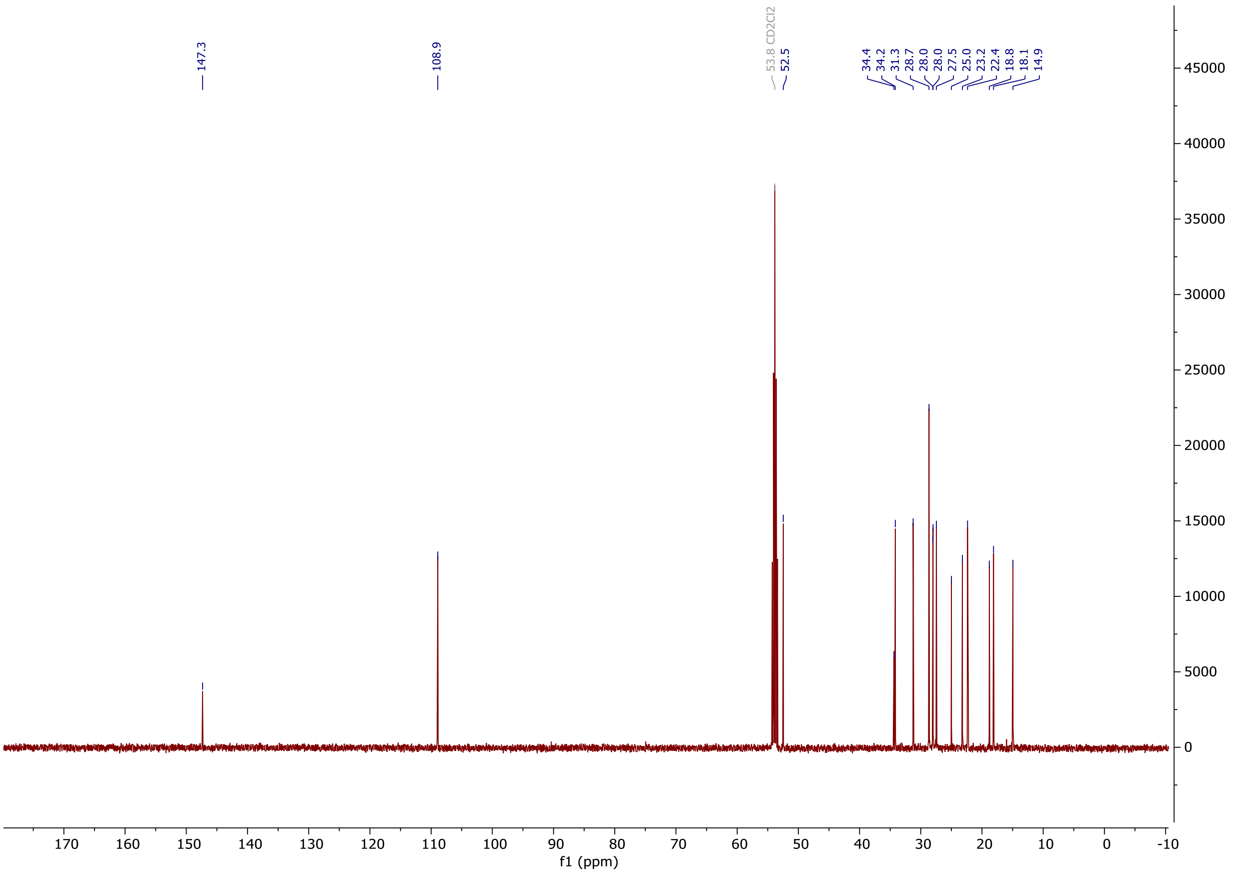

phenyl(propyl)(2,2,4-trimethylpent-4-en-1-yl)silanol 7’

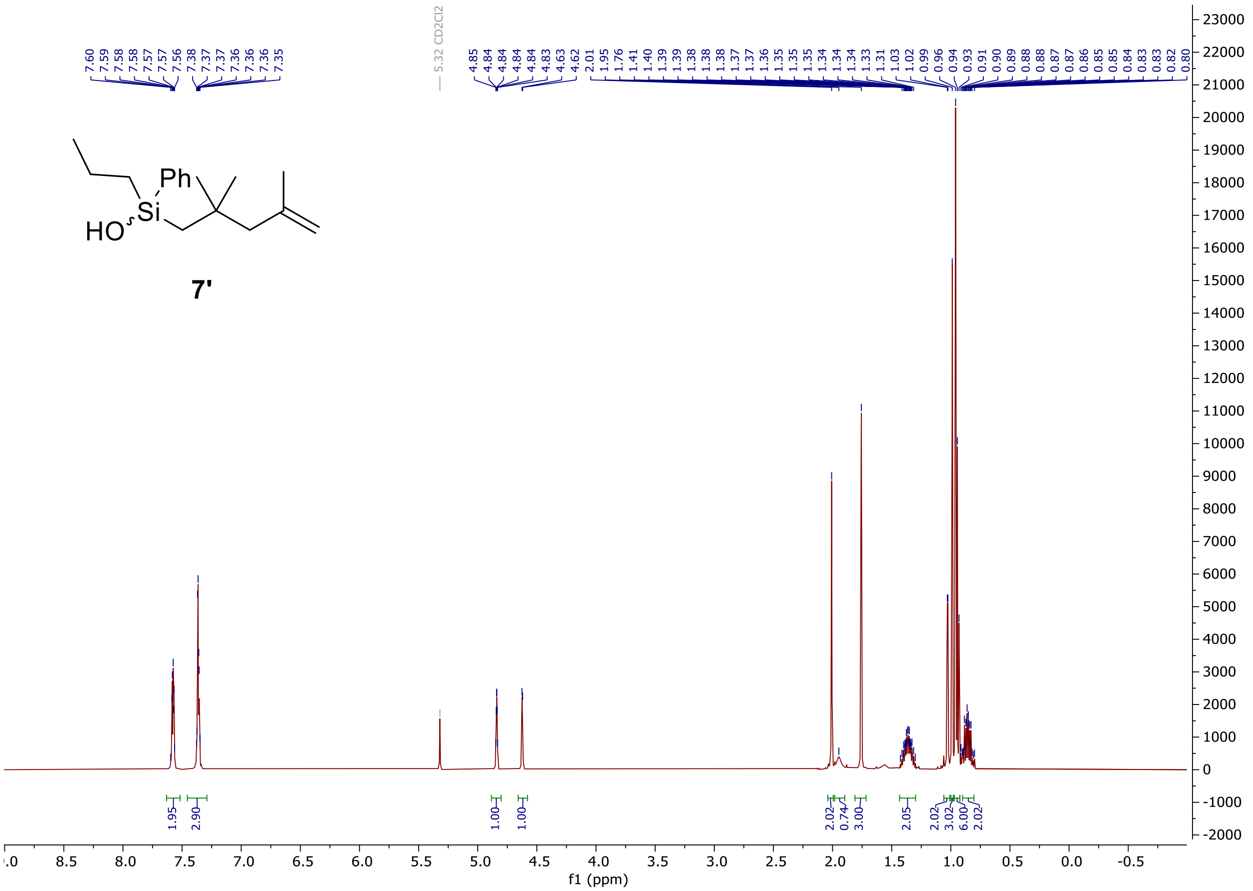

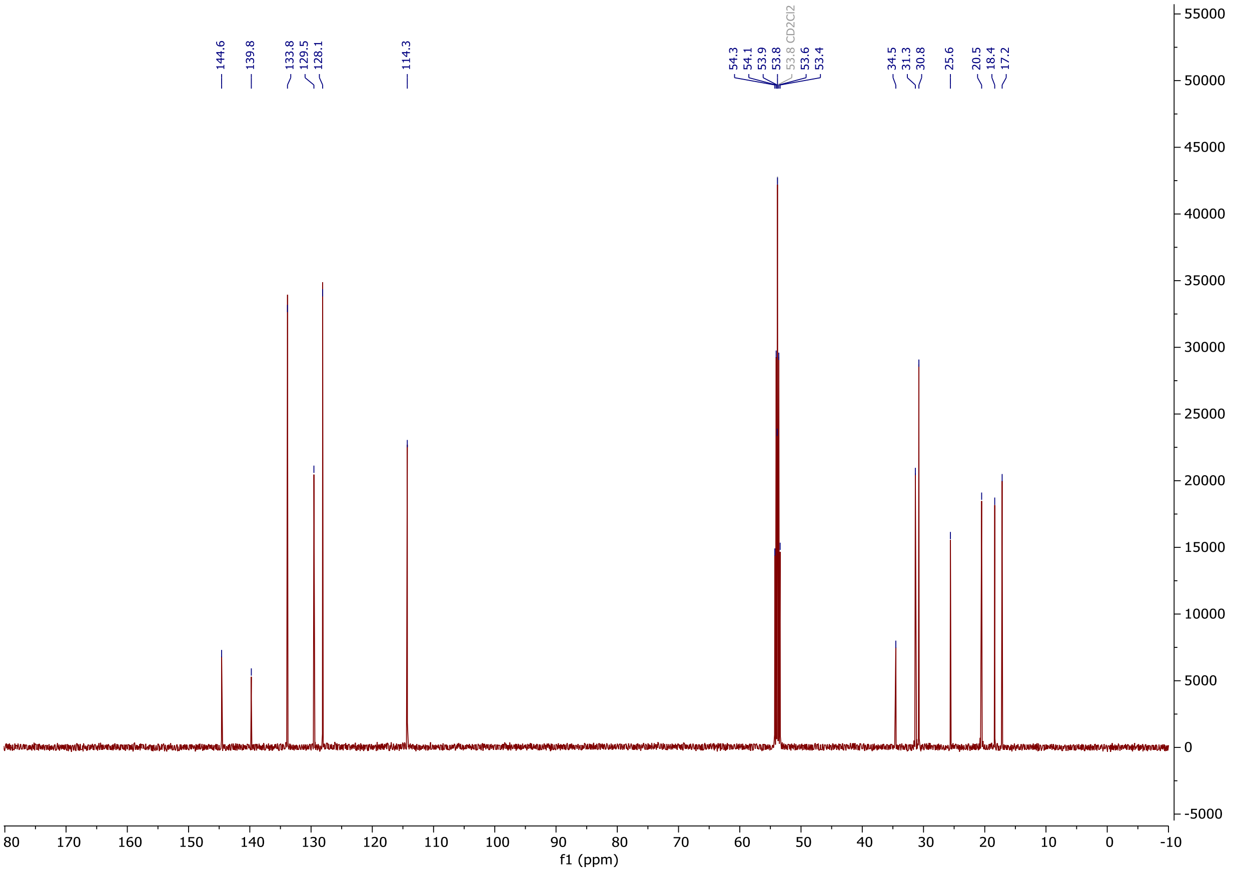

14. Copies of HPLC and GC traces

HPLC column: IG-3R, Acetonitrile: Water = 70:30, 1.0 mL/min, 298 K, 220 nm.

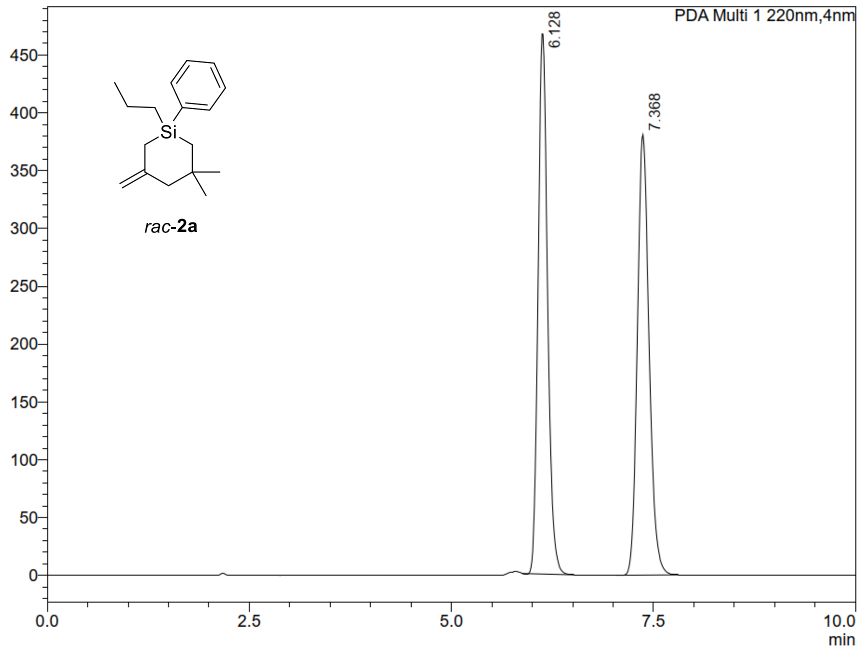

Peak #	t_R/min	% peak area
1	6.1	49.80
2	7.4	50.20
Total		100

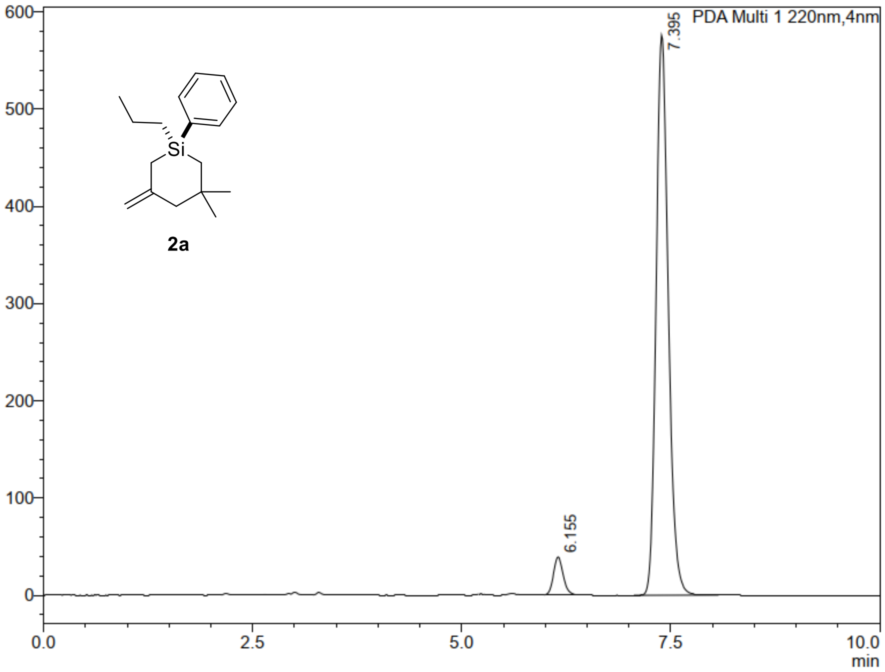

Peak #	t_R/min	% peak area
1	6.2	4.98
2	7.4	95.02
Total		100

HPLC column: IG-3R, Acetonitrile: Water = 70:30, 1.0 mL/min, 298 K, 220 nm.

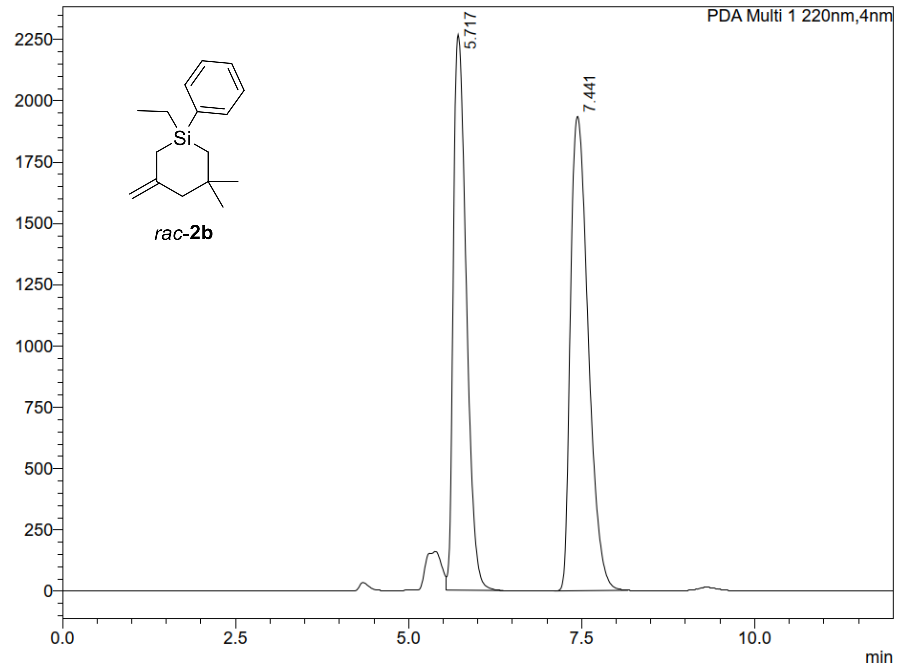

Peak #	t_R/min	% peak area
1	5.7	49.84
2	7.4	50.16
Total		100

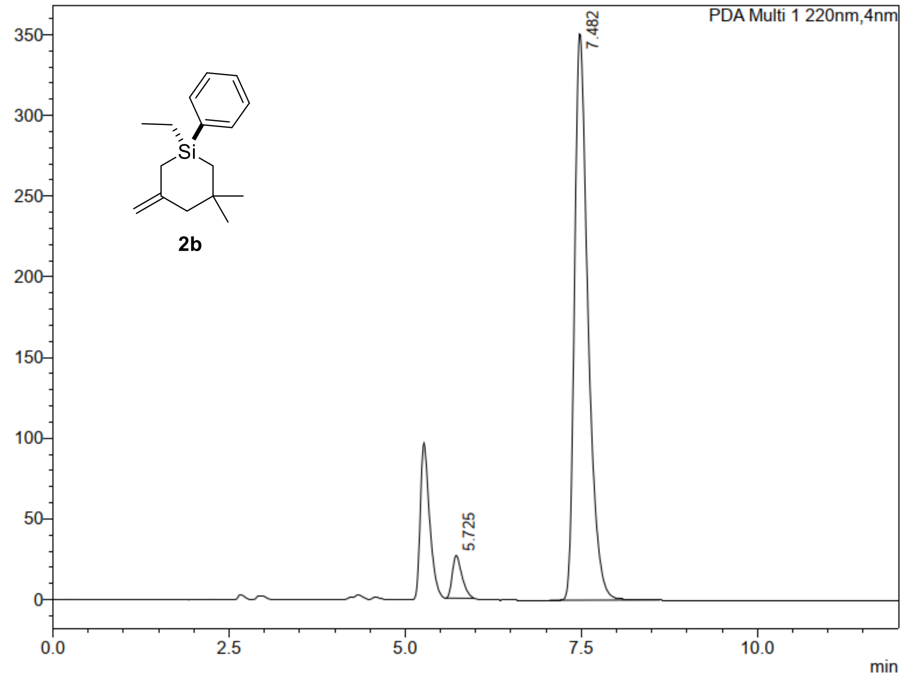

Peak #	t_R/min	% peak area
1	5.7	5.03
2	7.5	94.97
Total		100

HPLC column: OJ-3R, Acetonitrile: Water = 55:45, 1.0 mL/min, 298 K, 190 nm.

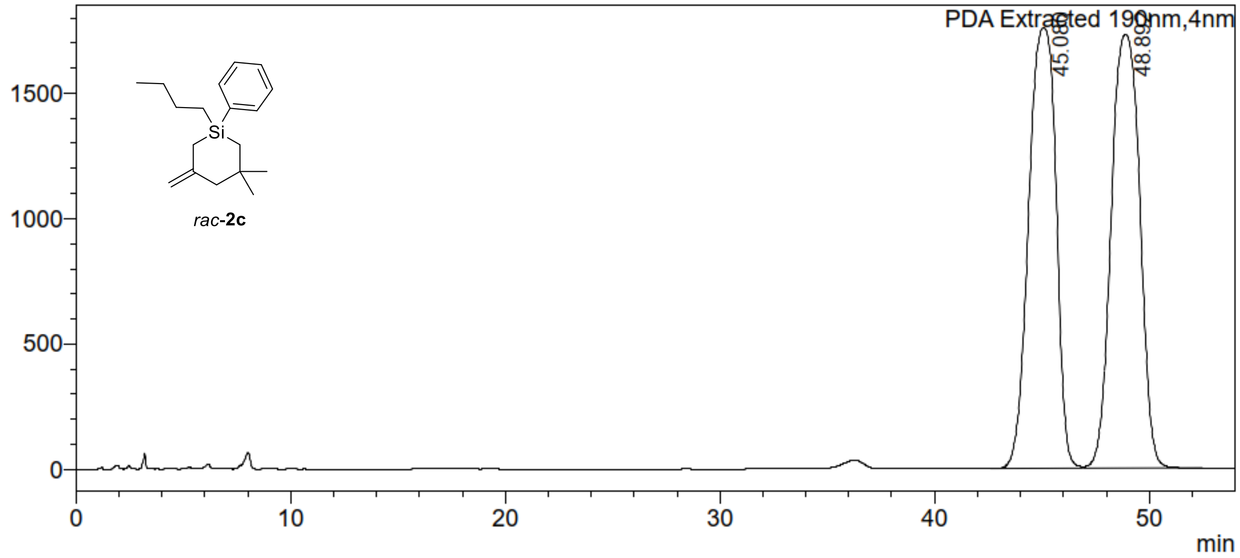

Peak #	t_R/min	% peak area
1	45.1	49.97
2	48.9	50.03
Total		100

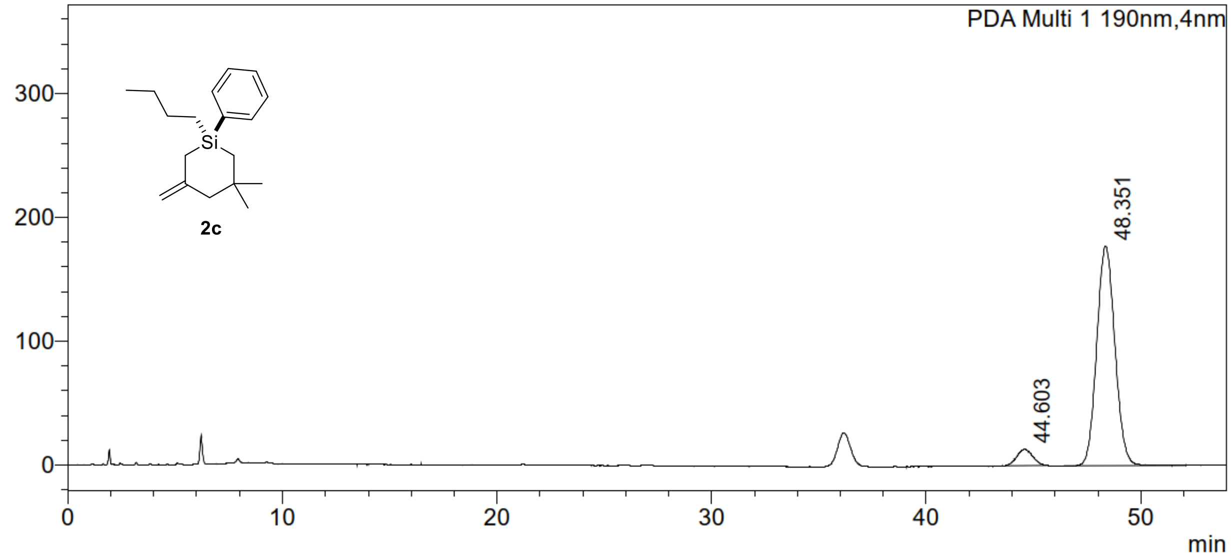

Peak #	t_R/min	% peak area
1	44.6	5.14
2	48.3	94.86
Total		100

HPLC column: IG-3R, Acetonitrile: Water = 70:30, 1.0 mL/min, 298 K, 220 nm.

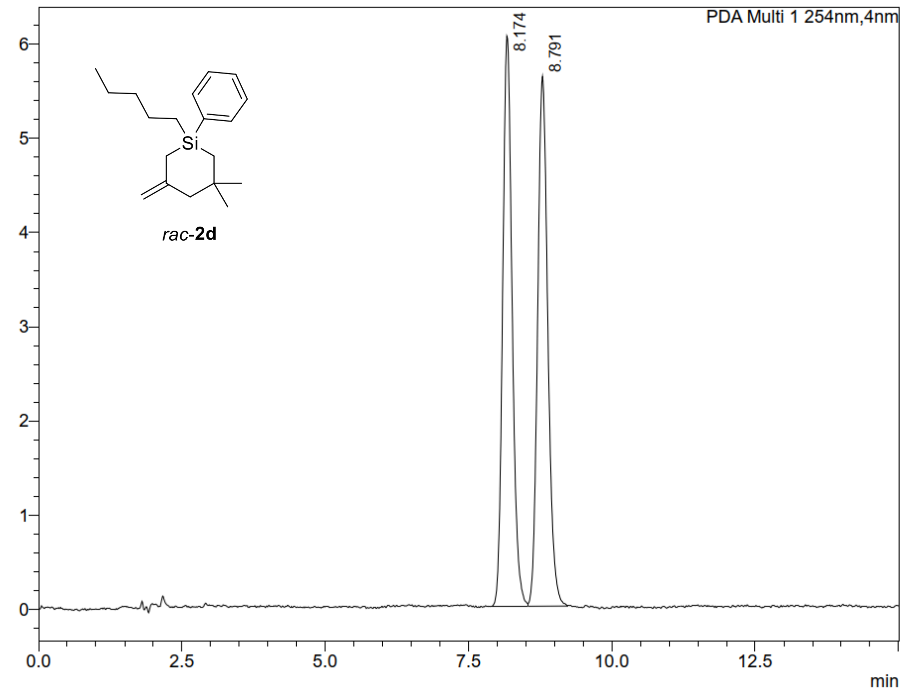

Peak #	t_R/min	% peak area
1	8.2	49.98
2	8.8	50.02
Total		100

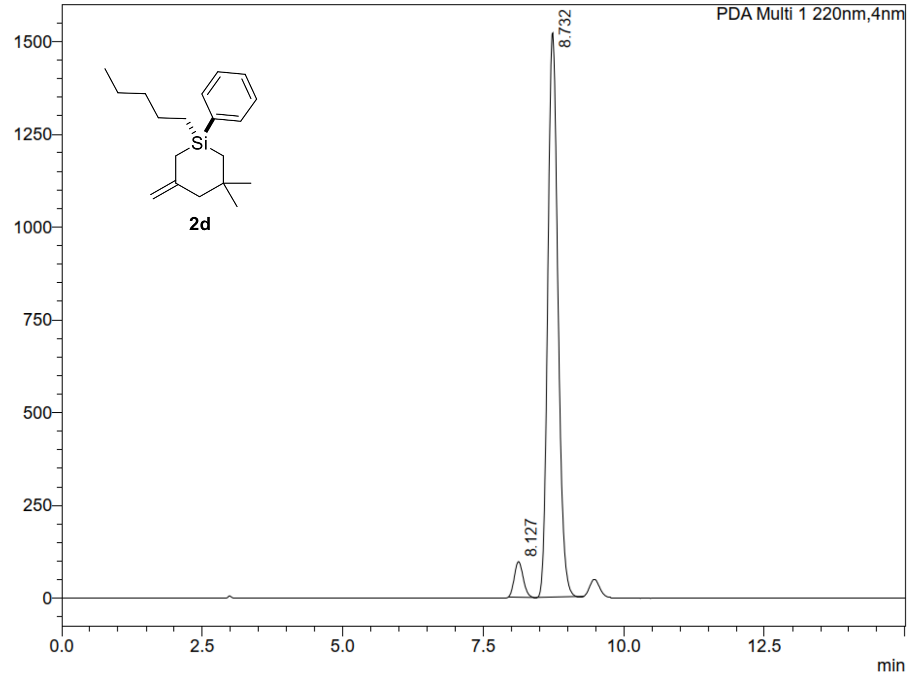

Peak #	t_R/min	% peak area
1	8.1	5.08
2	8.7	94.92
Total		100

HPLC column: IG-3R, Acetonitrile: Water = 60:40, 1.0 mL/min, 298 K, 220 nm.

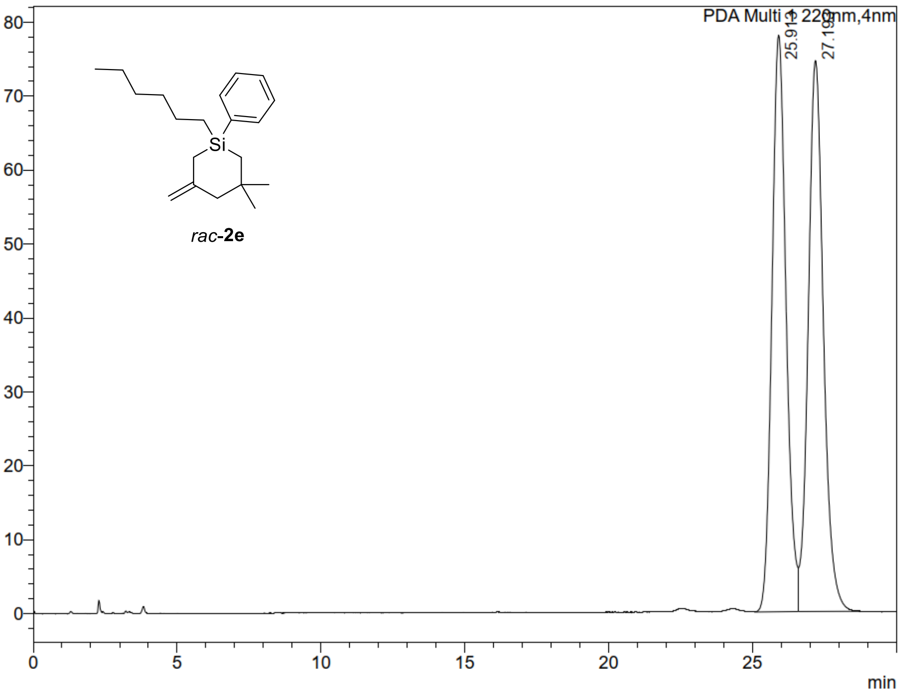

Peak #	t_R/min	% peak area
1	25.9	49.39
2	27.2	50.61
Total		100

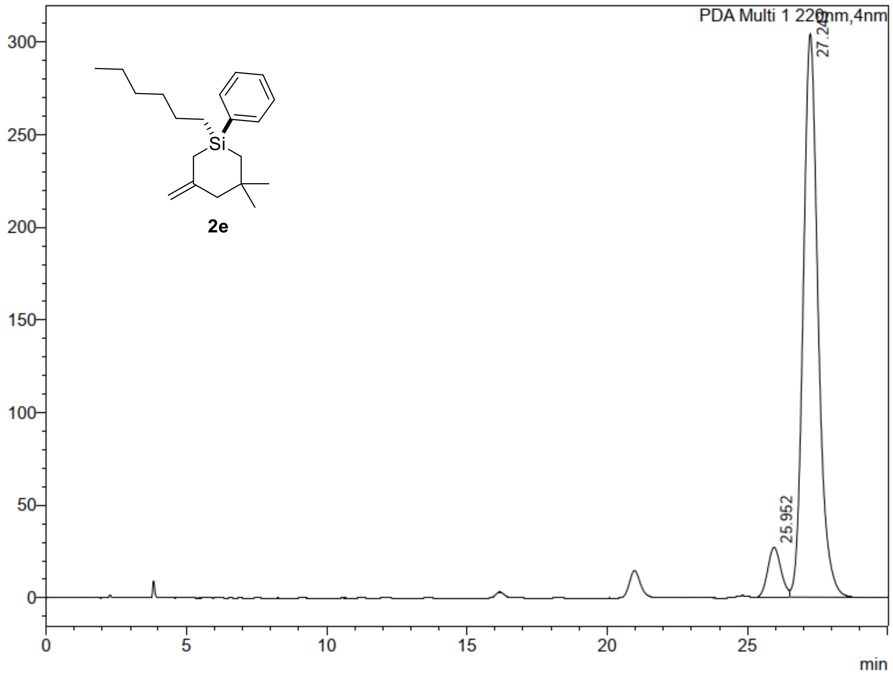

Peak #	t_R/min	% peak area
1	26.0	7.05
2	27.2	92.95
Total		100

HPLC column: IG-3R, Acetonitrile: Water = 60:40, 1.0 mL/min, 298 K, 220 nm.

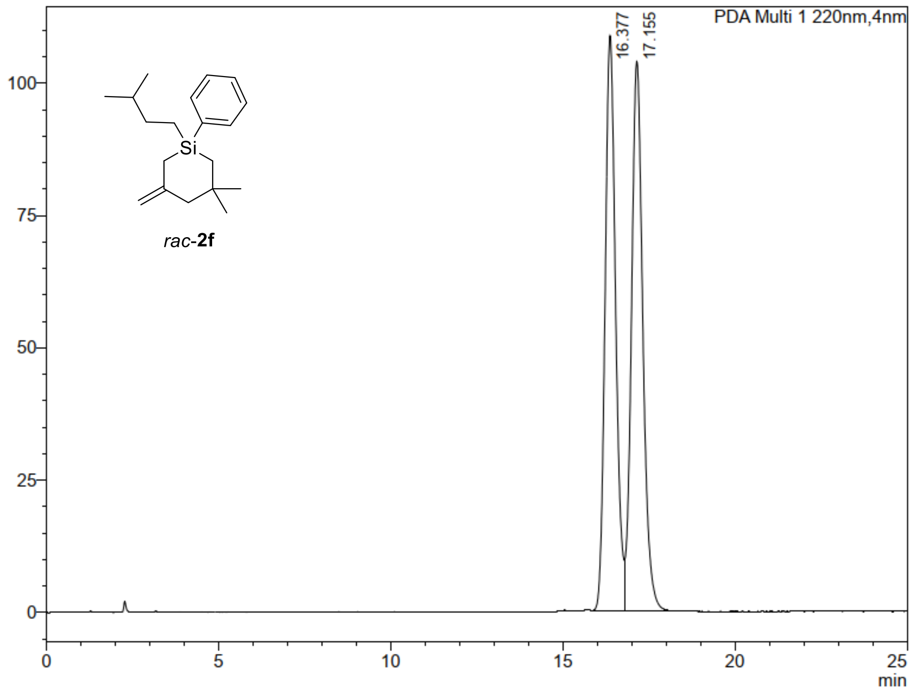

Peak #	t_R/min	% peak area
1	16.4	49.14
2	17.2	50.86
Total		100

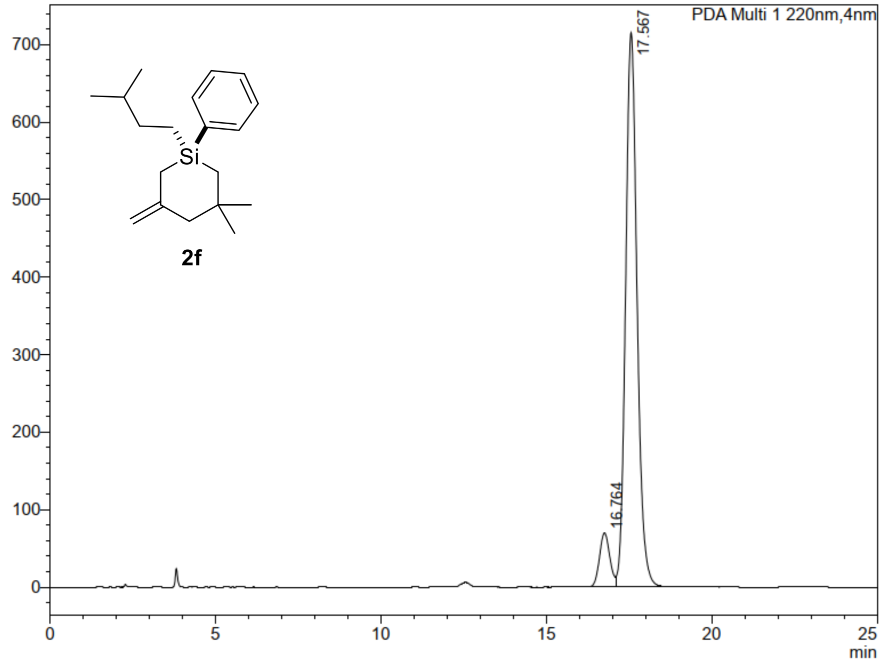

Peak #	t_R/min	% peak area
1	16.8	8.11
2	17.6	91.89
Total		100

HPLC column: IG-3R, Acetonitrile: Water = 70:30, 1.0 mL/min, 298 K, 220 nm.

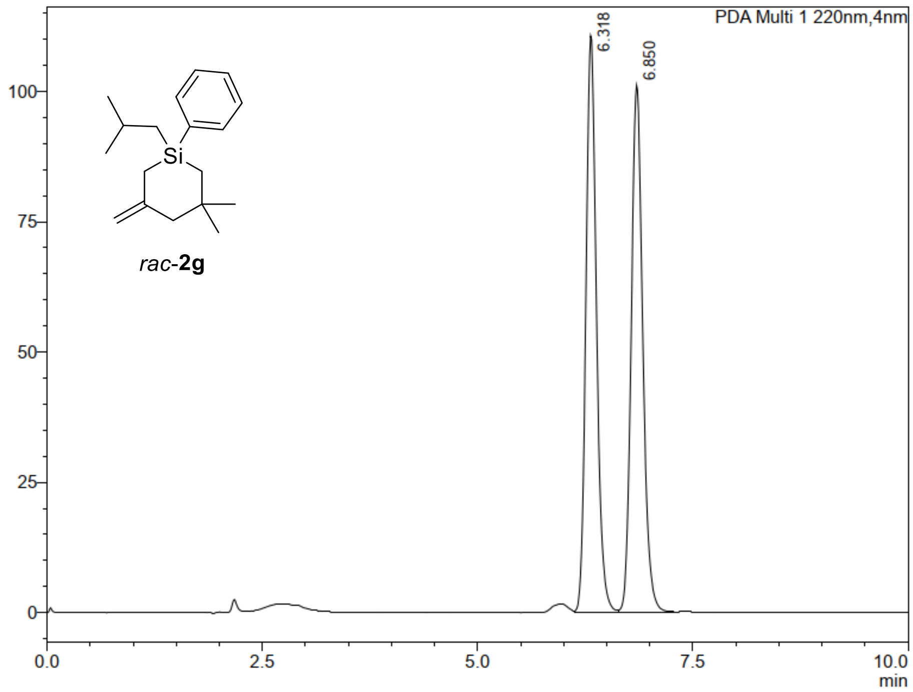

Peak #	t_R/min	% peak area
1	6.3	49.90
2	6.9	50.10
Total		100

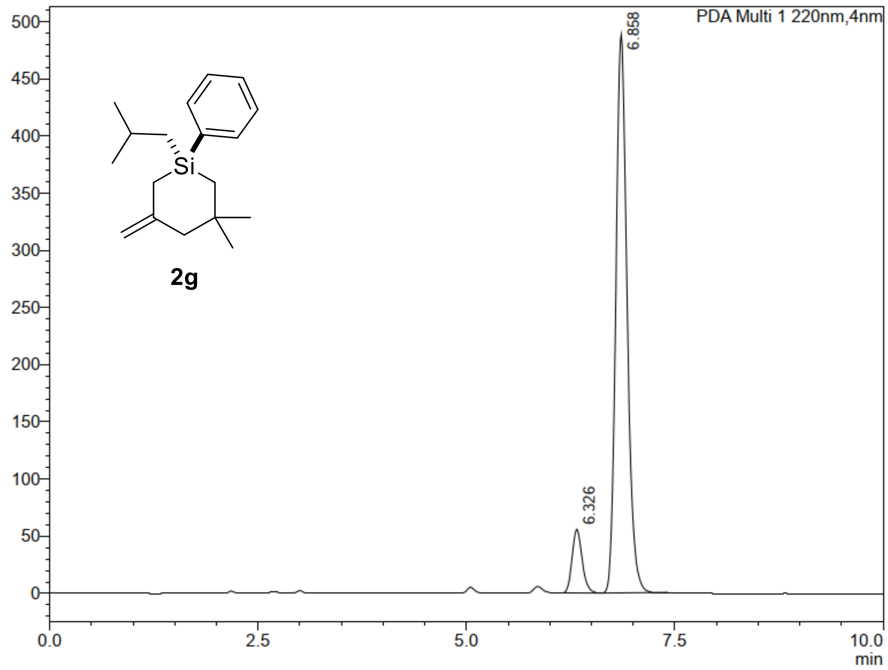

Peak #	t_R/min	% peak area
1	6.3	9.08
2	6.9	90.92
Total		100

HPLC column: OJ-3R, Acetonitrile: Water = 55:45, 1.0 mL/min, 298 K, 254 nm.

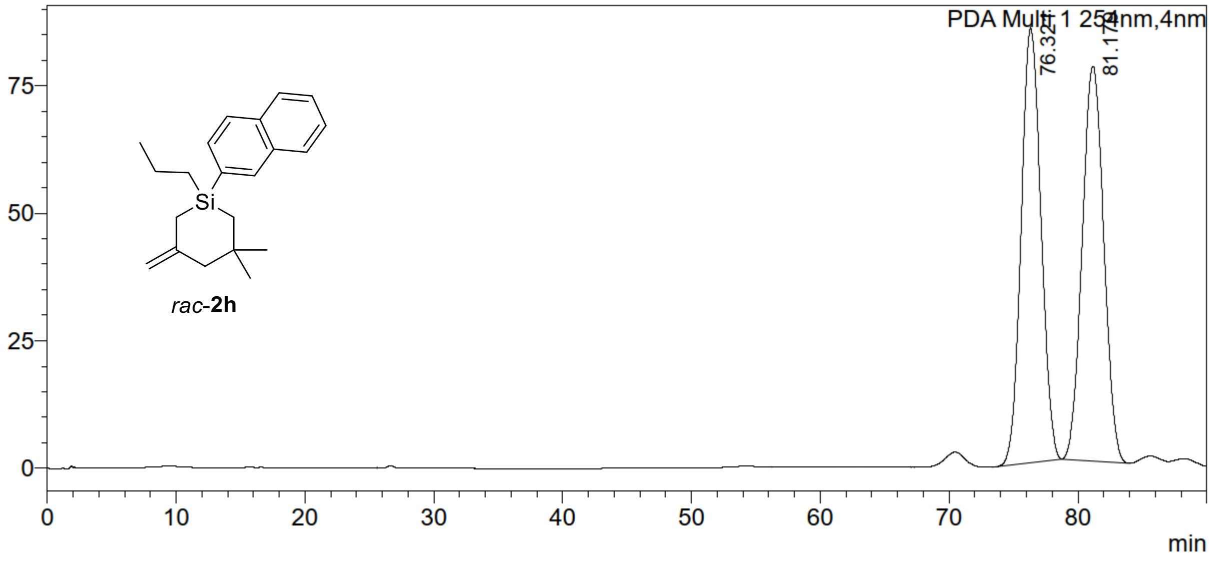

Peak #	t_R/min	% peak area
1	76.3	50.18
2	81.2	49.82
Total		100

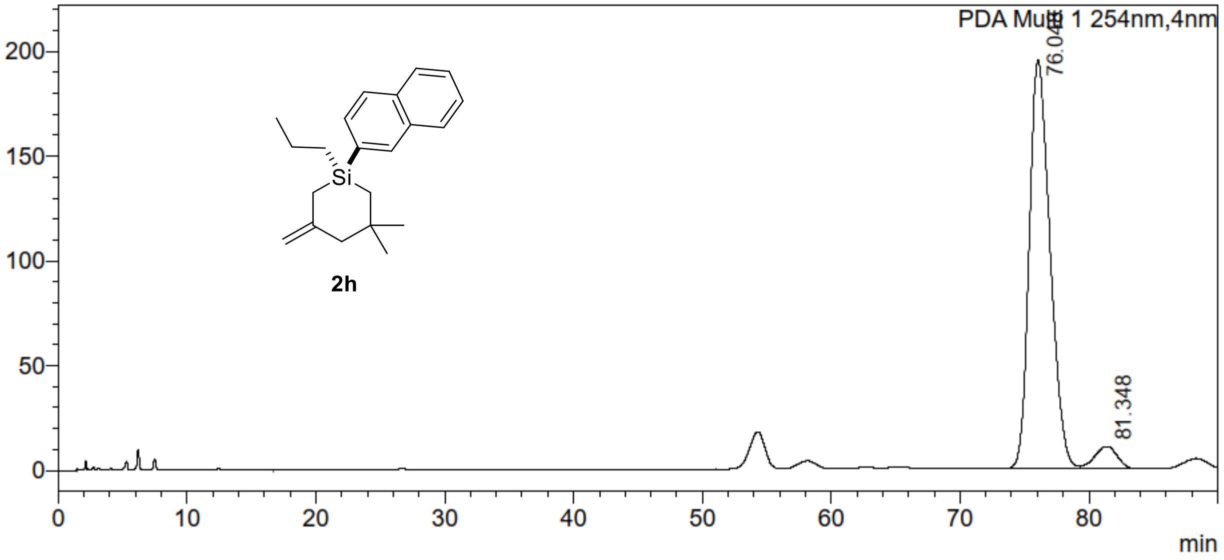

Peak #	t_R/min	% peak area
1	76.0	95.27
2	81.3	4.73
Total		100

HPLC column: OJ-3R, Acetonitrile: Water = 55:45, 1.0 mL/min, 298 K, 220 nm.

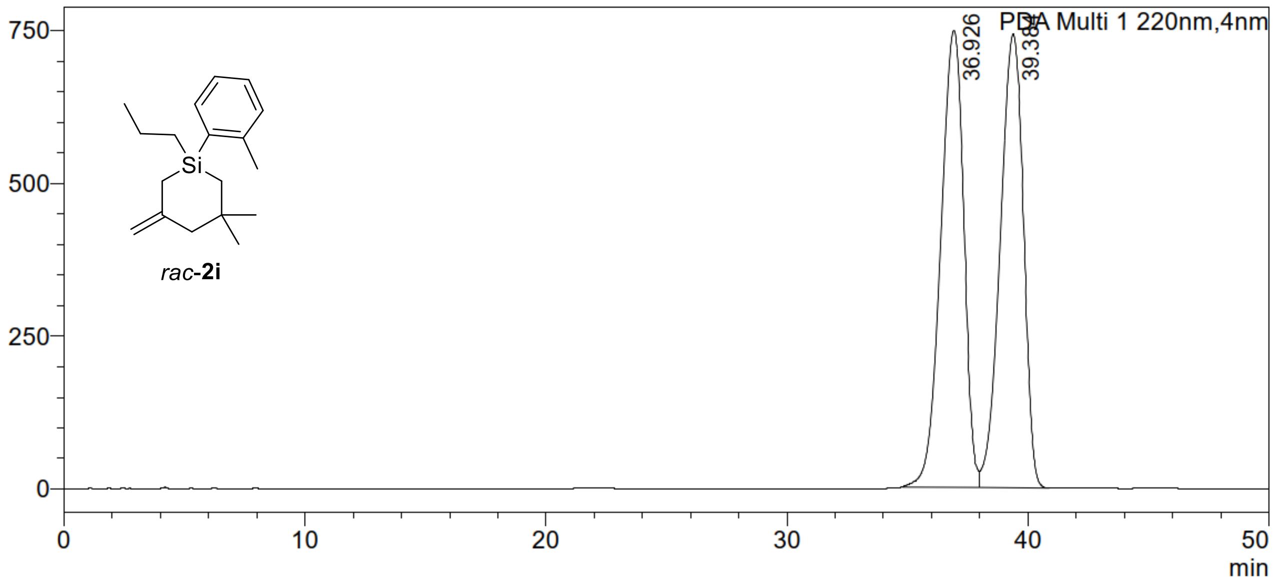

Peak #	t_R/min	% peak area
1	36.9	50.17
2	39.4	49.83
Total		100

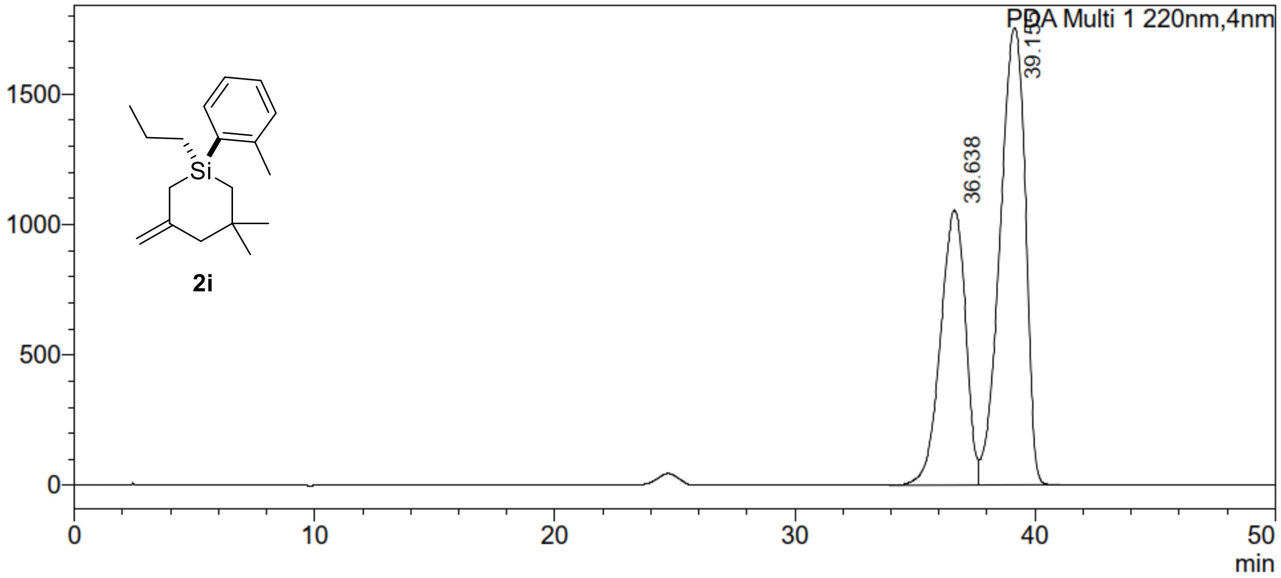

Peak #	t_R/min	% peak area
1	36.6	36.83
2	39.2	63.17
Total		100

HPLC column: IG-3R, Acetonitrile: Water = 70:30, 1.0 mL/min, 298 K, 220 nm.

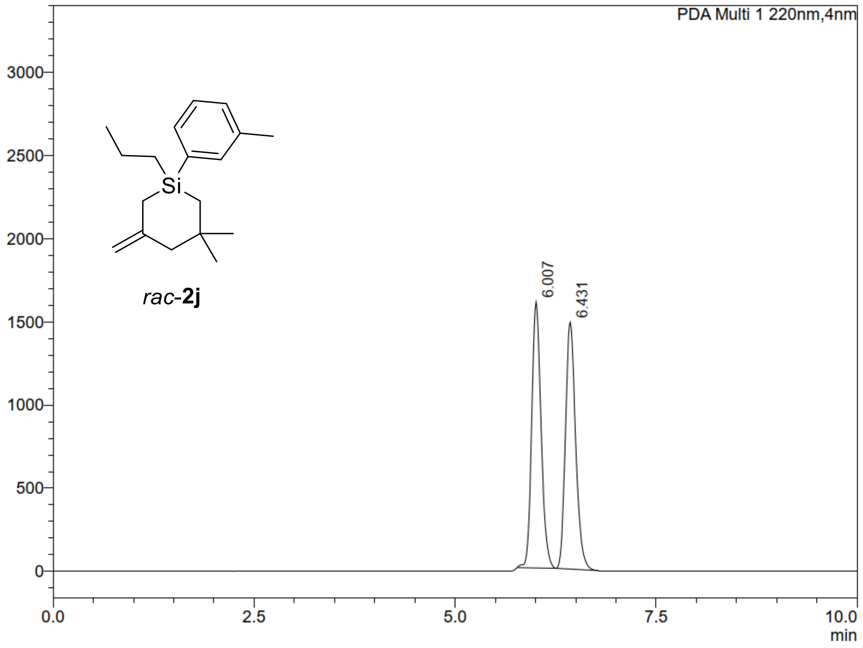

Peak #	t_R/min	% peak area
1	6.0	49.79
2	6.4	50.21
Total		100

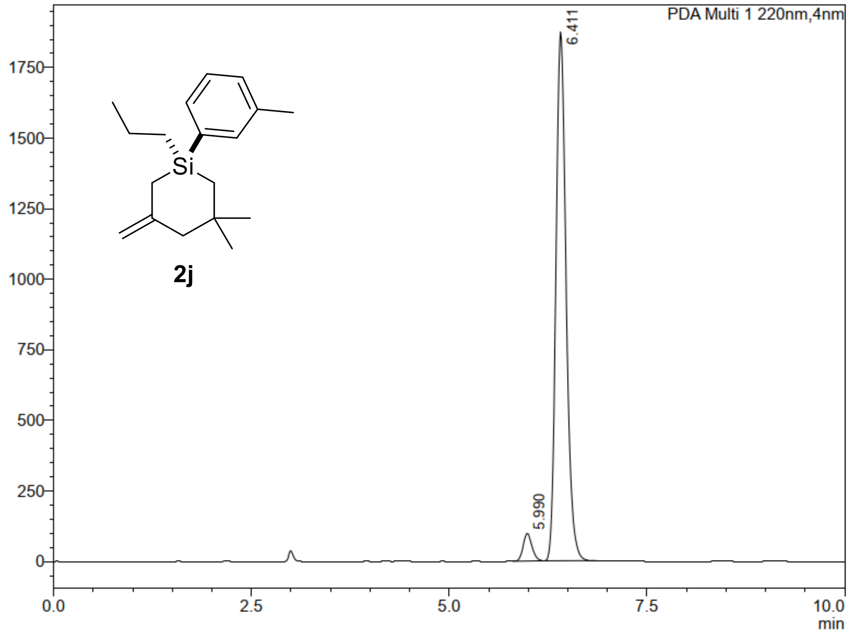

Peak #	t_R/min	% peak area
1	6.0	3.97
2	6.4	96.03
Total		100

HPLC column: IG-3R, Acetonitrile: Water = 70:30, 1.0 mL/min, 298 K, 220 nm.

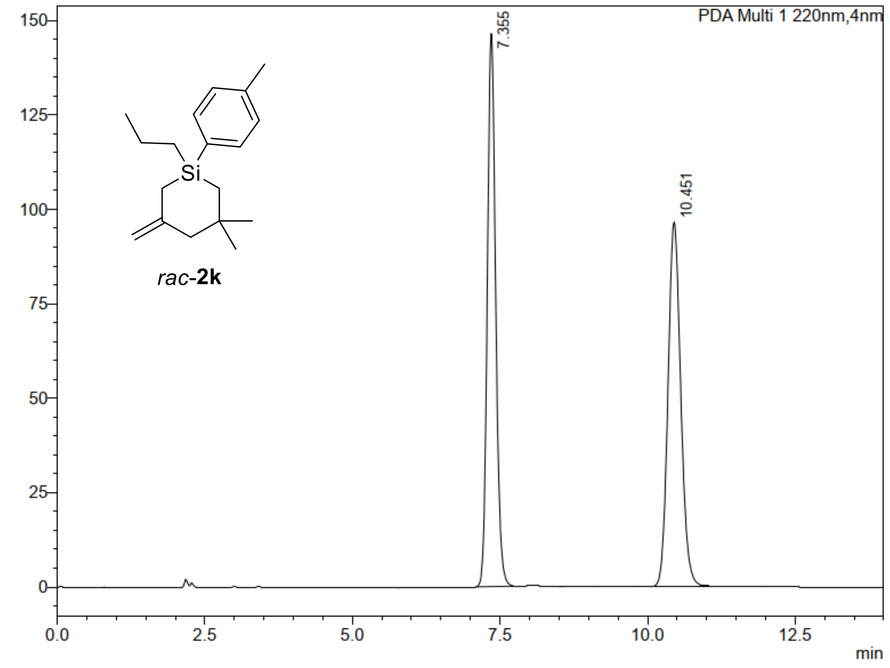

Peak #	t_R/min	% peak area
1	7.4	50.05
2	10.5	49.95
Total		100

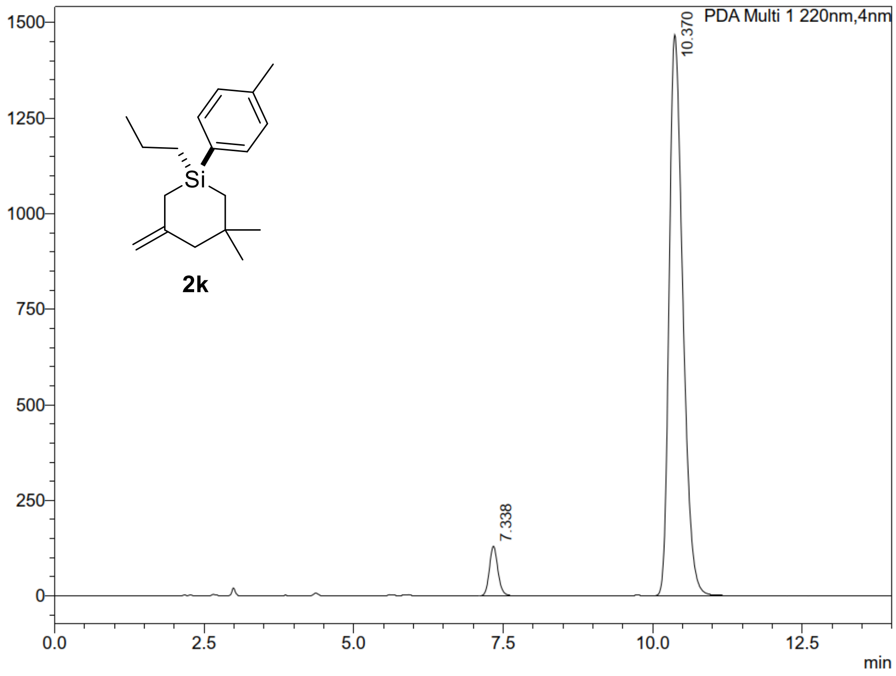

Peak #	t_R/min	% peak area
1	7.3	5.10
2	10.4	94.90
Total		100

HPLC column: IG-3R, Acetonitrile: Water = 60:40, 1.0 mL/min, 298 K, 220 nm.

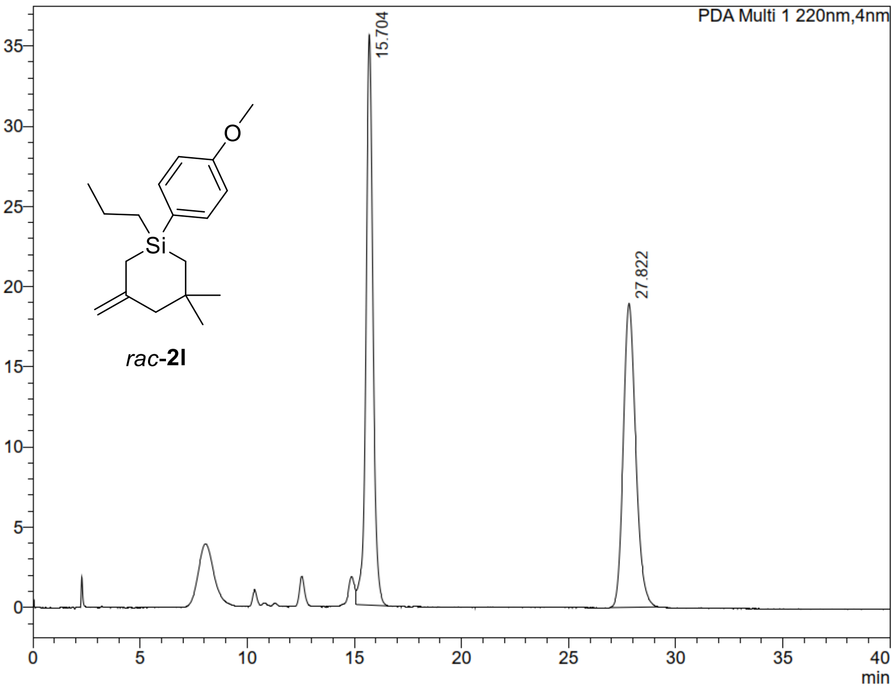

Peak #	t_R/min	% peak area
1	15.7	50.33
2	27.8	49.67
Total		100

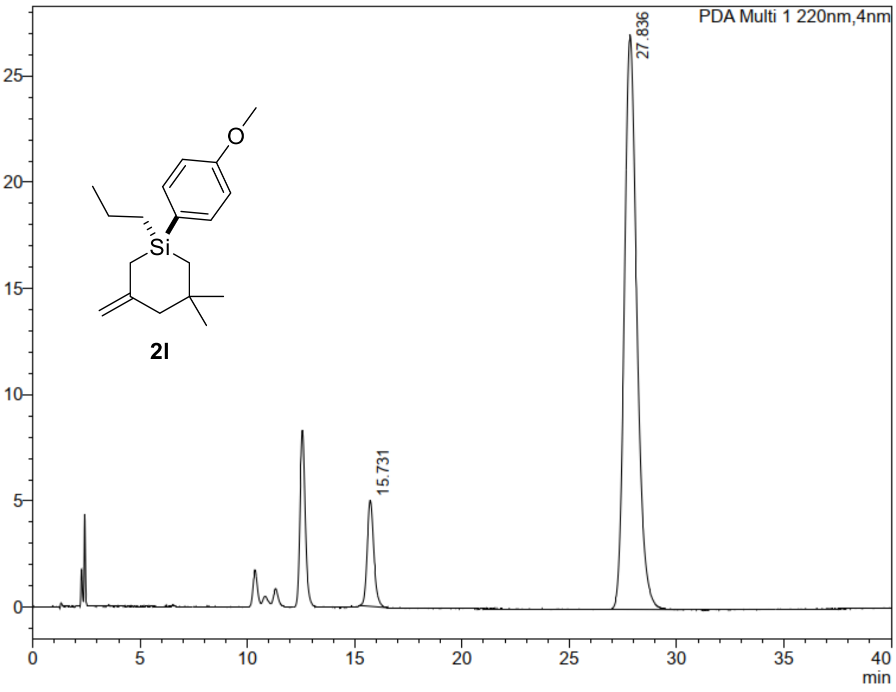

Peak #	t_R/min	% peak area
1	15.7	8.92
2	27.8	91.08
Total		100

HPLC column: IG-3R, Acetonitrile: Water = 70:30, 1.0 mL/min, 298 K, 220 nm.

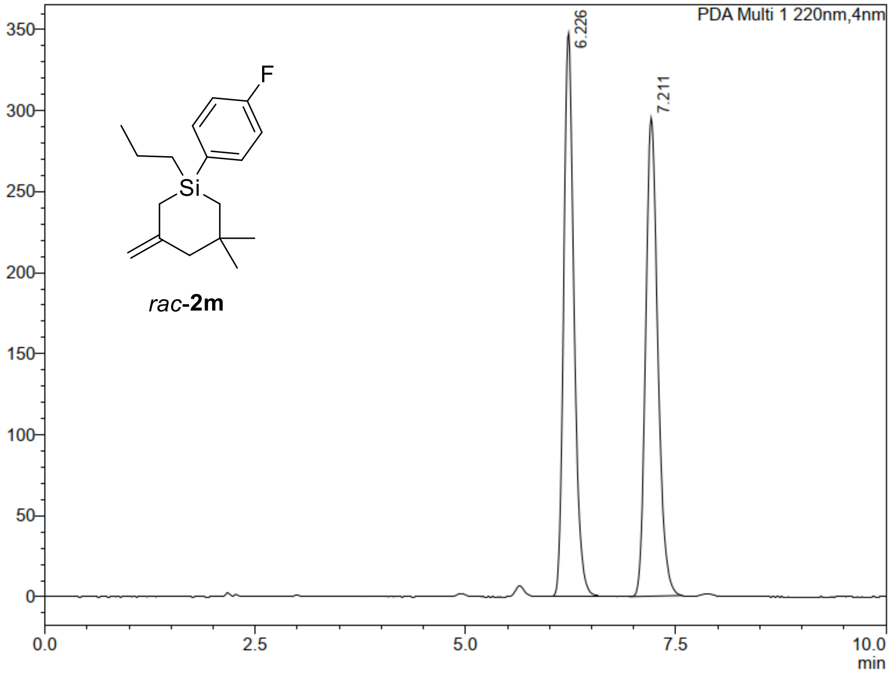

Peak #	t_R/min	% peak area
1	6.2	50.05
2	7.2	49.95
Total		100

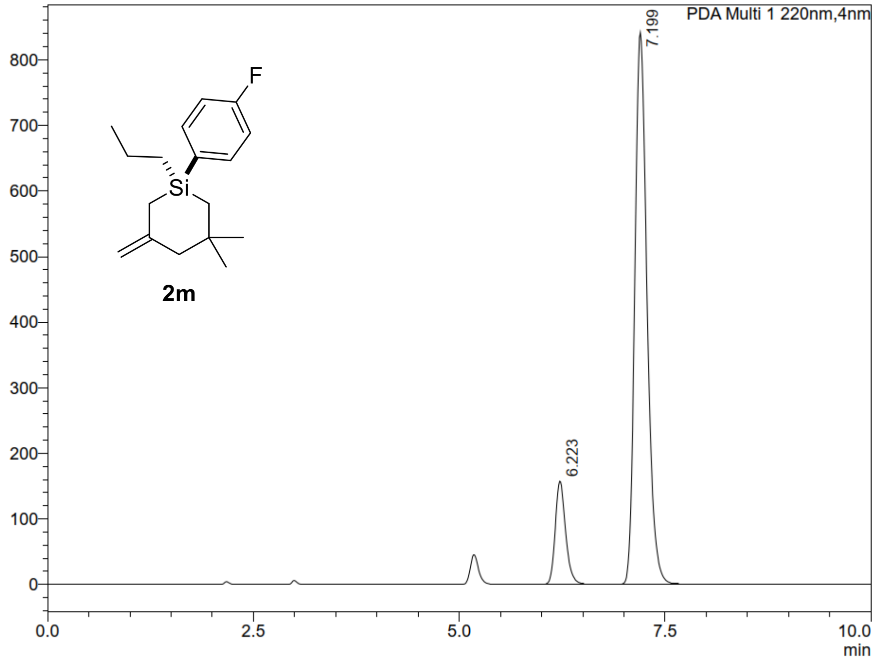

Peak #	t_R/min	% peak area
1	6.2	13.52
2	7.2	86.48
Total		100

HPLC column: IG-3R, Acetonitrile:Water = 60:40, 1.0 mL/min, 298 K, 220 nm.

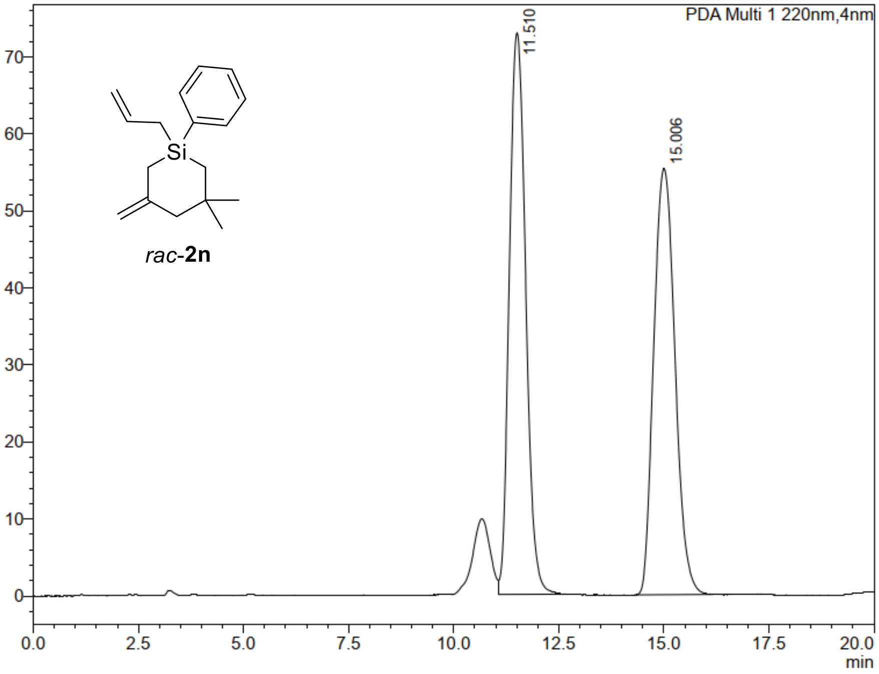

Peak #	t_R/min	% peak area
1	11.5	
2	14.0	
Total		100

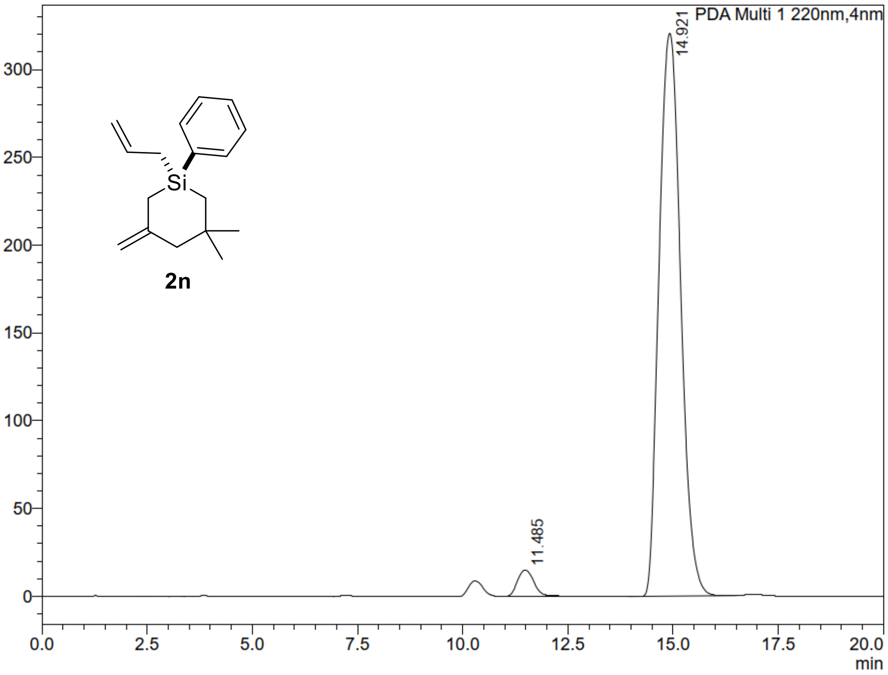

Peak #	t_R/min	% peak area
1	11.5	3.51
2	14.9	96.49
Total		100

HPLC column: IG-3R, Acetonitrile:Water = 70:30, 1.0 mL/min, 298 K, 220 nm.

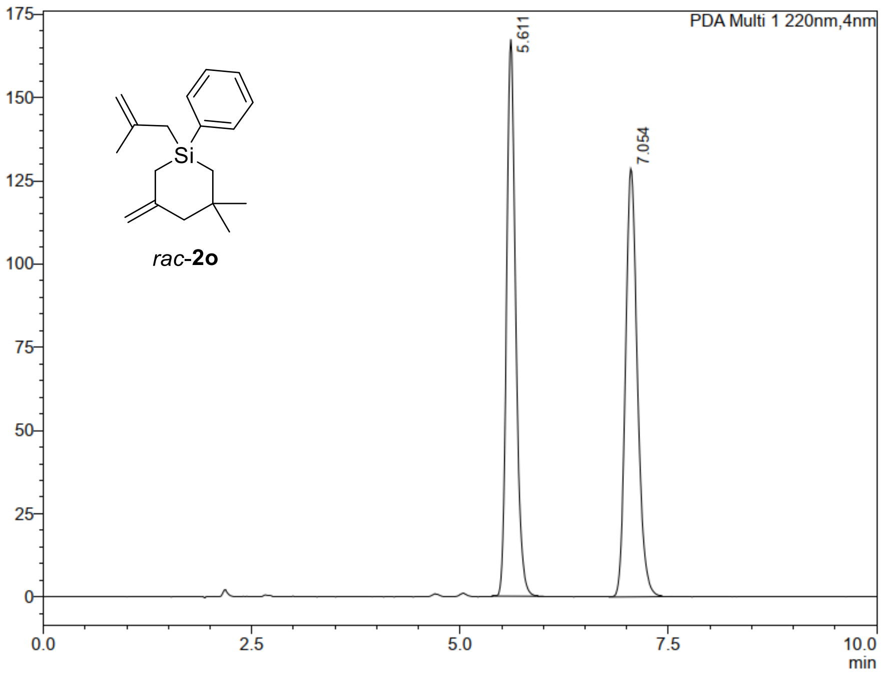

Peak #	t_R/min	% peak area
1	5.6	49.95
2	7.1	50.05
Total		100

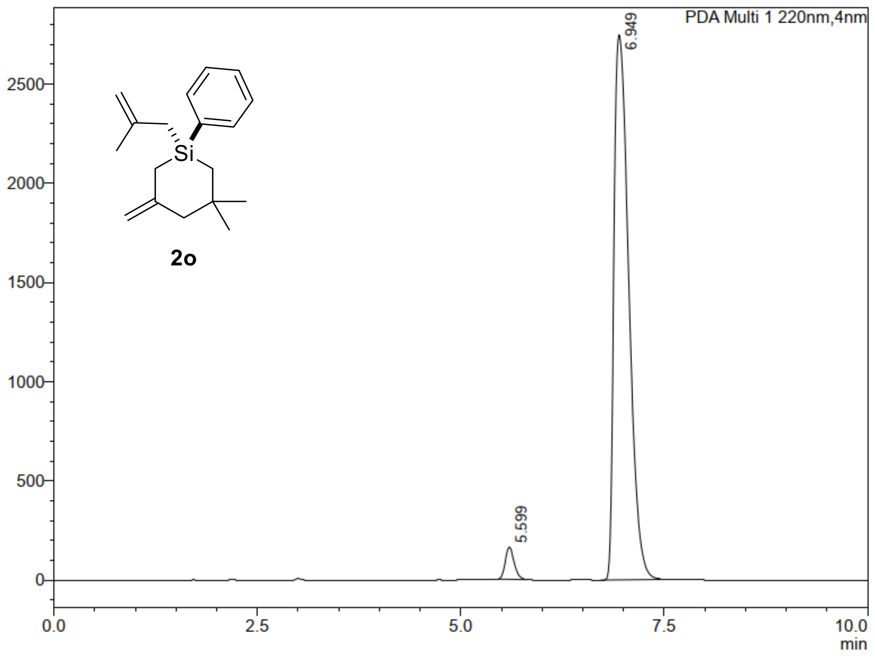

Peak #	t_R/min	% peak area
1	5.6	3.02
2	6.9	96.98
Total		100

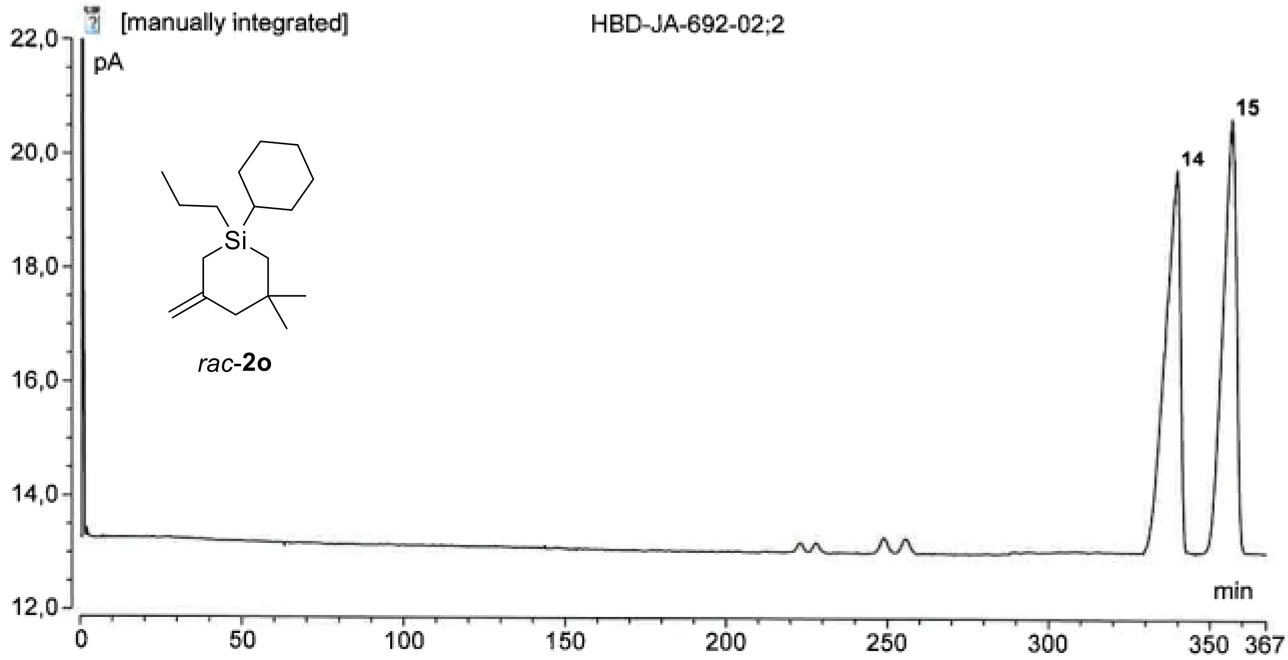

Sample:	**HBD-JA-692-02;2**	Instrument:	**GC_521**
Sequenz:	**8859 HBD-JA SE**	Measured:	**07.04.22 06:28**
Sequenz date:	**06.04.22**	Processing M.:	HBD
		Report-File:	Verhältnis 692-02

Racemat
Verhältnis der Enantiomere
Zuordnung achiral nach GCMS E36498 HBD-JA-692-02 22/8859

No.	Ret.Time min	Rel.Area %	Peak Name
14	340,03	50,01	MG:264
15	357,07	49,99	MG:264

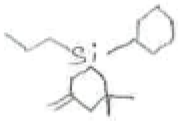

Instrument parameters:

Column:	24,0 m	Cyclodextrin-H 0,25/0,125df G/632
Temperature:	220/70 iso/350	
Gas:	0,50 bar	Hydrogen
Sample size:	0,2 µL	

Peak #	t_R/min	% peak area
1	340.0	50.01
2	357.1	49.99
Total		100

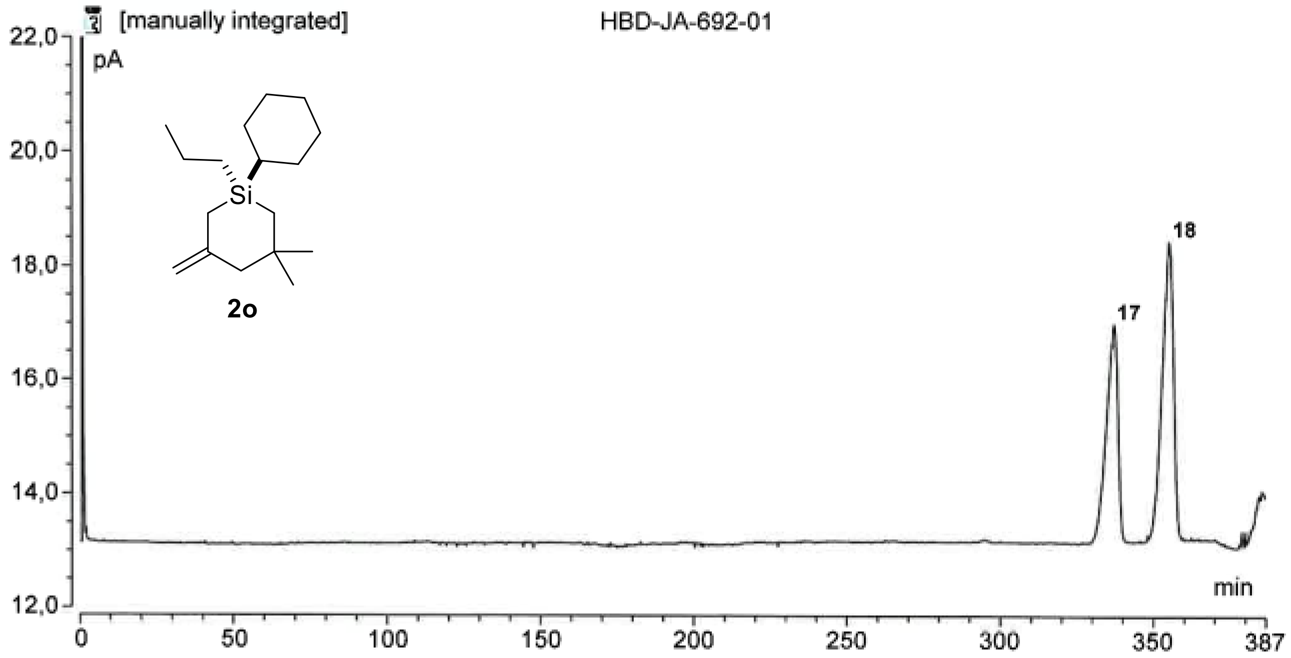

Sample:	**HBD-JA-692-01**	Instrument:	**GC_521**
Sequenz:	**8859 HBD-JA SE**	Measured:	**07.04.22 13:19**
Sequenz date:	**06.04.22**	Processing M.:	HBD
		Report-File:	Verhältnis 692-01

Verhältnis der Enantiomere
Zuordnung achiral nach GCMS E36498 HBD-JA-692-02 22/8859

No.	Ret.Time min	Rel.Area %	Peak Name
17	337,18	42,35	MG:264
18	355,27	57,65	MG:264

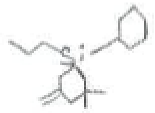

Instrument parameters:

Column:	24,0 m	Cyclodextrin-H 0,25/0,125df G/632
Temperature:	220/70 370iso 8/min 180 3min iso/350	
Gas:	0,50 bar	Hydrogen
Sample size:	0,2 µL	

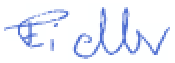

Peak #	t_R/min	% peak area
1	337.2	42.35
2	355.3	57.65
Total		100

HPLC column: IA-3, *n*-Heptane:Isopropanol = 98:2, 0.5 mL/min, 298 K, 220 nm.

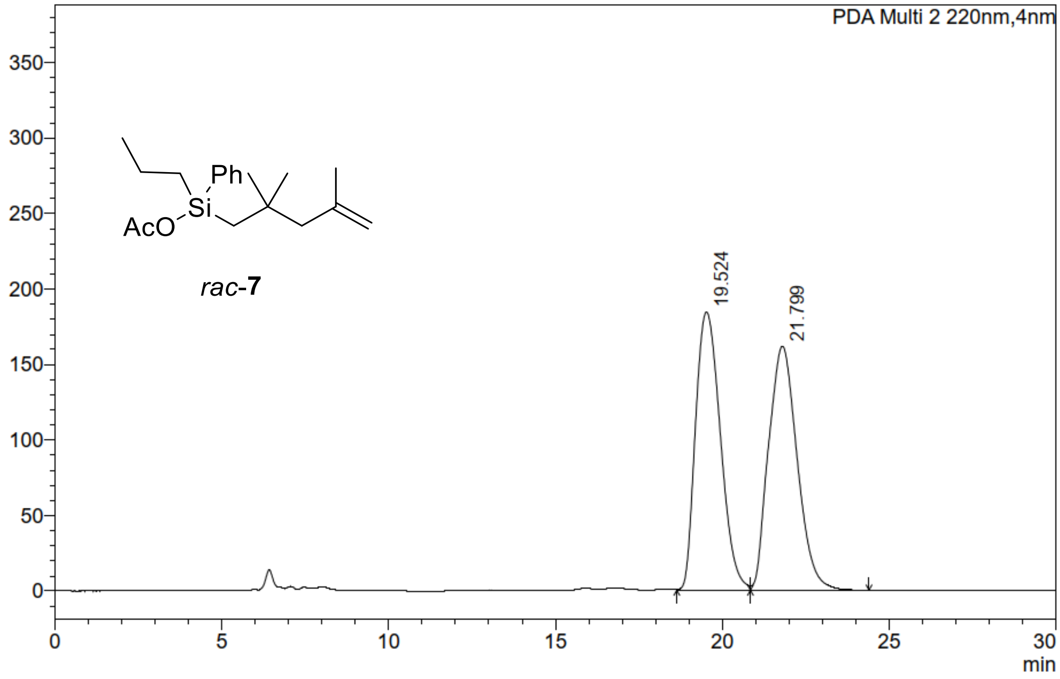

Peak #	t_R/min	% peak area
1	19.5	49.78
2	21.8	50.22
Total		100

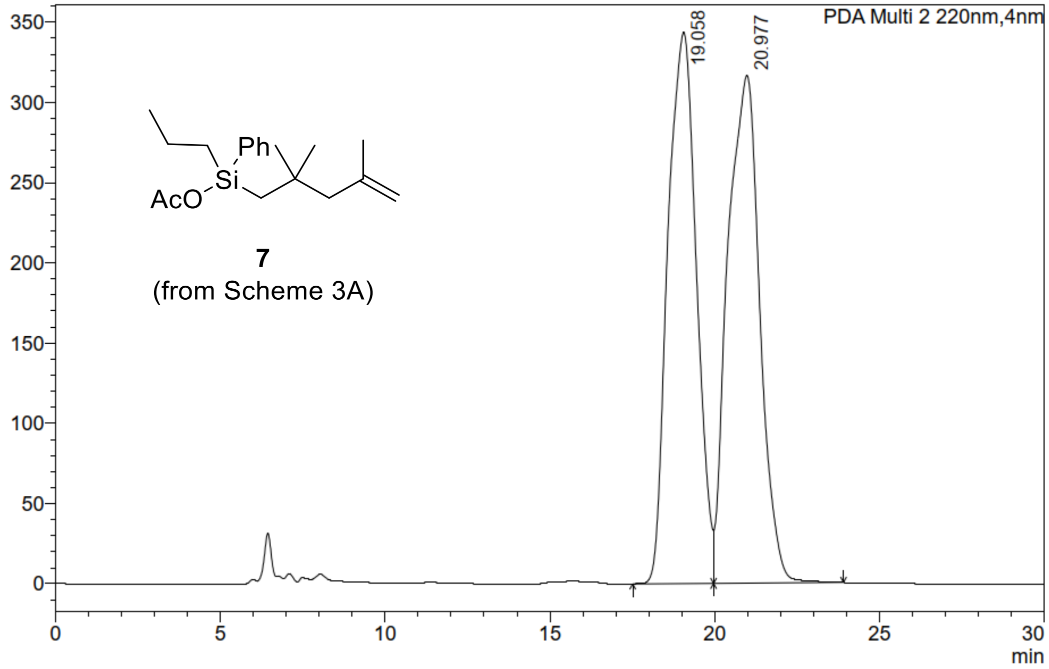

Peak #	t_R/min	% peak area
1	19.1	49.74
2	21.0	50.26
Total		100

HPLC column: IA-3, *n*-Heptane:Isopropanol = 98:2, 0.5 mL/min, 298 K, 220 nm.

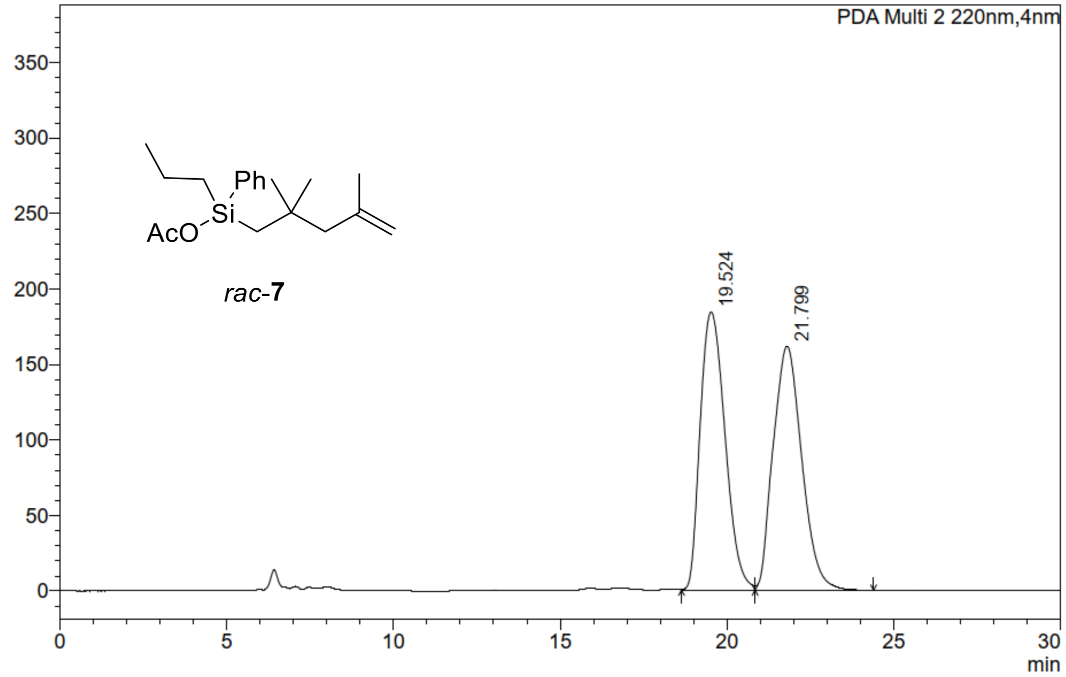

Peak #	t_R/min	% peak area
1	19.5	49.78
2	21.8	50.22
Total		100

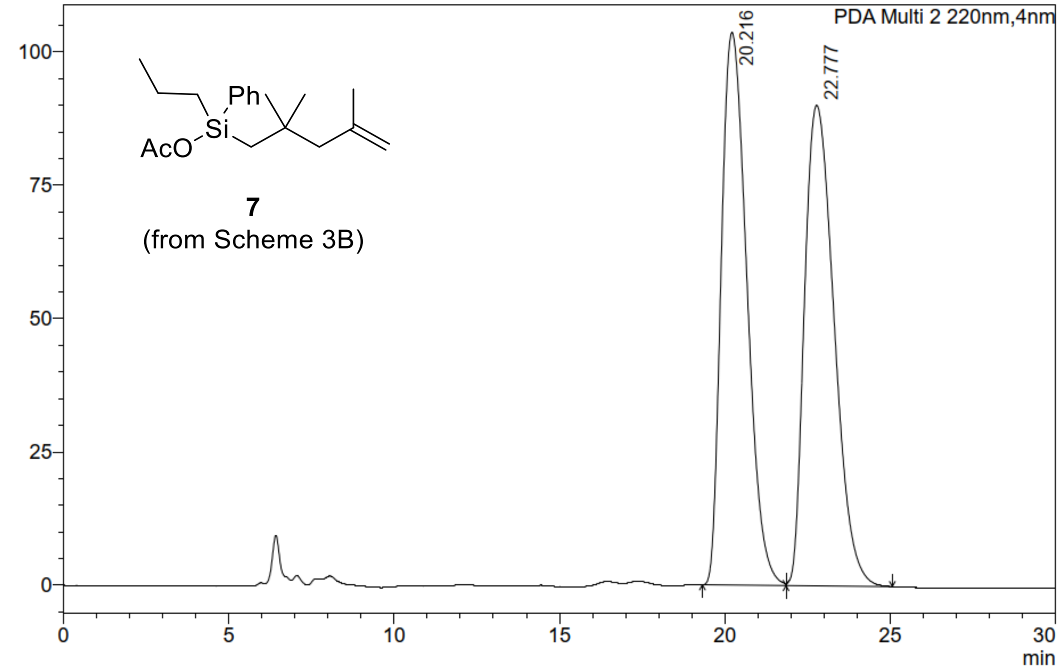

Peak #	t_R/min	% peak area
1	20.2	49.80
2	22.8	50.20
Total		100

14. References

1. Zhang, G., Li, Y., Wang, Y., Zhang, Q., Xiong, T. & Zhang, Q. *Angew. Chem. Int. Ed.* **59**, 11927–11931 (2020).

2. Zhou, H., Properzi, R., Leutzsch, M., Belanzoni, P., Bistoni, G., Tsuji, N., Han, J. T., Zhu, C. & List, B. *J. Am. Chem. Soc.* **145**, 4994–5000 (2023).

3. Kaib, P. S. J., Schreyer, L., Lee, S., Properzi, R. & List, B. *Angew. Chem. Int. Ed.* **55**, 13200–13203 (2016).

4. Lee, S., Bae, H. Y. & List, B. *Angew. Chem. Int. Ed.* **57**, 12162–12166 (2018).

5. Zhou, H., Han, J. T., Nöthling, N., Lindner, M. M., Jenniches, J., Kühn, C., Tsuji, N., Zhang, L. & List, B. *J. Am. Chem. Soc.* **145**, 4994–5000 (2022).

6. Neese F. *WIREs Comput. Mol. Sci.* **2**, 73–78 (2012).

7. Becke, A. D. *J. Chem. Phys*. **98**, 5648–5652 (1993).

8. Lee, C., Yang, W. & Parr, R. G. *Phys. Rev. B* **37**, 785–789 (1988).

9. Grimme, S., Antony, J., Ehrlich, S. & Krieg, H. *J. Chem. Phys.* **132**, 154104 (2010).

10. Grimme, S., Ehrlich, S. & Goerigk, L. *J. Comput. Chem.* **32**, 1456–1465 (2011).

11. Weigend, F. *Phys. Chem. Chem. Phys.* **8**, 1057–1065 (2006).

12. Weigend, F. & Ahlrichs, R. *Phys. Chem. Chem. Phys.* **7**, 3297–3305 (2005).

13. Valeev, E. F., Libint: A library for the evaluation of molecular integrals of many-body operators over Gaussian functions, *http://libint.valeyev.net/*

14. Miertuš, S., Scrocco, E. & Tomasi, J. *Chem. Phys.* **55**, 117–129 (1981).

15. Lu, T. & Chen, F. *J. Comput. Chem.* **33**, 580–592 (2012).

16. Xtb, Version 6.3; University Bonn: 2020; please refer to xtb@thch.uni-bonn.de.

17. Lu, T. *molclus program*, *http://www.keinsci.com/research/molclus.html*.

18. Maeda, S., Harabuchi, Y., Takagi, M., Taketsugu, T. & Morokuma, K. *Chem. Rec.* **16**, 2232–2248 (2016).

19. Maeda, S., Ohno, K. & Morokuma, K. *Phys. Chem. Chem. Phys.* **15**, 3683–3701 (2013).

20. Bannwarth, C., Ehlert, S. & Grimme, S. *J. Chem. Theory Comput.* **15**, 1652–1671 (2019).

21. Neese, F. *WIREs Comput Mol Sci* **2**, 73–78 (2011).

22. Becke, A. D. *J. Chem. Phys.* **98**, 5648–5652 (1993).

23. Frisch, M. J., Trucks, G. W., Schlegel, H. B., Scuseria, G. E., Robb, M. A., Cheeseman, J. R., Scalmani, G., Barone, V., Petersson, G. A., Nakatsuji, H., Li, X., Caricato, M., Marenich, A. V., Bloino, J., Janesko, B. G., Gomperts, R., Mennucci, B., Hratchian, H. P., Ortiz, J. V., Izmaylov, A. F., Sonnenberg, J. L., Williams-Young, D., Ding, F., Lipparini, F., Egidi, F., Goings, J., Peng, B., Petrone, A., Henderson, T., Ranasinghe, D., Zakrzewski, V. G., Gao, J., Rega, N., Zheng, G., Liang, W., Hada, M., Ehara, M., Toyota, K., Fukuda, R., Hasegawa, J., Ishida, M., Nakajima, T., Honda, Y., Kitao, O., Nakai, H., Vreven, T., Throssell, K., Montgomery Jr., J. A., Peralta, J. E., Ogliaro, F., Bearpark, M. J., Heyd, J. J., Brothers, E. N., Kudin, K. N., Staroverov, V. N., Keith, T. A., Kobayashi, R., Normand, J., Raghavachari, K., Rendell, A. P., Burant, J. C., Iyengar, S. S., Tomasi, J., Cossi, M., Millam, J. M., Klene, M., Adamo, C., Cammi, R., Ochterski, J. W., Martin, R. L., Morokuma, K., Farkas, O., Foresman, J. B., Fox, D. J. Gaussian, Inc. Wallingford CT (2016).

24. Grimme, S., Hansen, A., Ehlert, S. & Mewes, J.-M. *J. Chem. Phys.* **154**, 064103 (2021).

25. Neese, F. *WIREs Comput Mol Sci.* **12**, e1606 (2022).

26. Cossi, M., Rega, N., Scalmani, G. & Barone, V. *J. Comput. Chem.* **24**, 669–681 (2003).

27. Mardirossian, N. & Head-Gordon, M. J. *Chem. Phys.* **144**, 214110 (2016).

28. Zheng, J., Xu, X. & Truhlar, D. G. *Theor. Chem. Acc.* **128**, 295–305 (2011).

29. Weigend, F. & Ahlrichs, R. *Phys. Chem. Chem. Phys.* **7**, 3297–3305 (2005).

30. Pettersen, E. F., Goddard, T. D., Huang, C. C., Couch, G. S., Greenblatt, D. M., Meng, E. C. & Ferrin, T. E. *J. Comput. Chem.* **25**, 1605–1612 (2004).

31. Peng, Q., Duarte, F. & Paton, R. S. *Chem. Soc. Rev.* **45**, 6093–6107 (2016).

32. Humphrey, W., Dalke, A. & Schulten, K. *J. Mol. Graphics* **14**, 33–38 (1996).

33. Contreras-García, J., Johnson, E. R., Keinan, S., Chaudret, R., Piquemal, J.-P., Beratan, D. N. & Yang, W. *J. Chem. Theory Comput.* **7**, 625–632 (2011).
